# Evolution of combinatorial diversity in *trans*-acyltransferase polyketide synthase assembly lines across bacteria

**DOI:** 10.1038/s41467-021-21163-x

**Published:** 2021-03-03

**Authors:** Eric J. N. Helfrich, Reiko Ueoka, Marc G. Chevrette, Franziska Hemmerling, Xiaowen Lu, Stefan Leopold-Messer, Hannah A. Minas, Adrien Y. Burch, Steven E. Lindow, Jörn Piel, Marnix H. Medema

**Affiliations:** 1 Institute of Microbiology, Eidgenössische Technische Hochschule (ETH) Zürich, Zürich, Switzerland; 2 Institute for Molecular Bio Science, Goethe University Frankfurt, Frankfurt am Main, Germany; 3 Wisconsin Institute for Discovery, Department of Plant Pathology, University of Wisconsin-Madison, Madison, WI USA; 4 Bioinformatics Group, Wageningen University, Wageningen, The Netherlands; 5 Department of Plant and Microbial Biology, University of California at Berkeley, Berkeley, CA USA

**Keywords:** Natural product synthesis, Computational biology and bioinformatics, Phylogeny, Biosynthesis

## Abstract

*Trans*-acyltransferase polyketide synthases (*trans*-AT PKSs) are bacterial multimodular enzymes that biosynthesize diverse pharmaceutically and ecologically important polyketides. A notable feature of this natural product class is the existence of chemical hybrids that combine core moieties from different polyketide structures. To understand the prevalence, biosynthetic basis, and evolutionary patterns of this phenomenon, we developed *trans*PACT, a phylogenomic algorithm to automate global classification of *trans*-AT PKS modules across bacteria and applied it to 1782 *trans*-AT PKS gene clusters. These analyses reveal widespread exchange patterns suggesting recombination of extended PKS module series as an important mechanism for metabolic diversification in this natural product class. For three plant-associated bacteria, i.e., the root colonizer *Gynuella sunshinyii* and the pathogens *Xanthomonas cannabis* and *Pseudomonas syringae*, we demonstrate the utility of this computational approach for uncovering cryptic relationships between polyketides, accelerating polyketide mining from fragmented genome sequences, and discovering polyketide variants with conserved moieties of interest. As natural combinatorial hybrids are rare among the more commonly studied *cis*-AT PKSs, this study paves the way towards evolutionarily informed, rational PKS engineering to produce chimeric *trans*-AT PKS-derived polyketides.

## Abstract (web summary)

*Trans*-acyltransferase polyketide synthases are multimodular enzymes that synthesise diverse polyketides. Here, the authors present an algorithm for the global study of their diversity, showing exchange of conserved consecutive modules as a driver of diversification, and guiding the discovery of polyketides.

## Introduction

*Trans*-acyltransferase polyketide synthases (*trans*-AT PKSs) are giant bacterial enzymes that assemble highly diverse and complex polyketide natural products. Some of these, such as mupirocin and virginiamycin, are used as antibiotics in human and veterinary medicine^1,2^, and many others show promising drug-like bioactivities^3–6^. In addition, *trans*-AT PKS-derived polyketides are of ecological relevance as they have been linked to symbiotic interactions^7–9^ and pathogenicity^10–12^. *Trans*-AT PKSs are broadly distributed across the bacterial tree of life, including many chemically underexplored lineages^13–15^. Like the well-studied *cis*-AT PKSs, they form multifunctional assembly lines composed of multiple enzymatic modules, each of which is responsible for the incorporation and facultative processing of an acyl-CoA-derived building block. In the *trans*-AT systems, these units are usually malonyl residues that are attached by one or few free-standing acyltransferase (AT) enzymes to an acyl carrier protein (ACP) domain present in each module. A ketosynthase (KS) domain in the downstream module then uses this building block to elongate the presented intermediate, resulting in a β-keto thioester. Additional optional domains, either located within a module or also acting in *trans*, can further modify this thioester in the α-to-γ region to generate a product that is passed on to the next module. When the polyketide backbone is fully assembled, it is released from the PKS, typically by a thioesterase (TE) domain. Additional post-PKS enzymes may modify the polyketide backbone to produce the mature polyketide^13,16^. The diversity of module architectures (>150 known variants)^8^ and enzymatic domains found in *trans*-AT PKSs is remarkable, and far exceeds the architectural diversity of *cis*-AT PKSs^13,17^. In many cases, PKSs form hybrids with nonribosomal peptide synthetases (NRPSs) to produce hybrid polyketide–peptide backbones^13^.

Phylogenetic studies suggest that *trans*-AT PKSs evolve in a distinct fashion compared to textbook *cis*-AT PKSs. In *cis*-AT PKS systems, in which an AT domain is present in every module, pathways appear to largely arise from module duplication and subsequent divergence^18,19^, probably in combination with gene conversion^20^. This evolutionary scenario is reflected in the phylogeny of KS domains, which are usually monophyletic within the same *cis*-AT PKS family^21^. In contrast, the phylogeny of KS domains from *trans*-AT PKSs tightly correlates with the α-to-γ structural region of their thioester substrates^17,22,23^, which is at least in part a consequence of their substrate selectivity^22,24^. Accordingly, KS domains in the same assembly line that do not recognize the same substrate type are usually more remotely related and do not group together in phylogenetic trees. This correspondence between KS sequences and the structure of their accepted polyketide intermediates, referred to as the *trans*-AT PKS correlation rule^17^, permits detailed predictions of polyketide structures from *trans*-AT PKS biosynthetic gene clusters (BGCs)^17,22,23,25–30^.

Previous studies suggested for pederin-type compounds^31^ and a family of actin-binding macrolides^29^, respectively, that natural product structures within each of these compound groups have diversified by horizontal gene transfer and combinatorial reorganization of large *trans*-AT PKS gene fragments encompassing multiple PKS modules, here termed module blocks. At the chemical level, this mechanism creates complex chimeric polyketide families that share extended structural moieties, reminiscent of compounds generated by mix-and-match combinatorial chemistry^29,31^. In contrast, such hybrid assembly lines and their associated chimeric metabolites are not typically observed for textbook *cis*-AT PKSs^23^. In actin-binding macrolides, PKS chimerization enables extensive structural diversification, as well as preservation of pharmacophore moieties in otherwise structurally unrelated compounds.

We wanted to explore whether the ability to form hybrid assembly lines is a general feature of *trans*-AT PKSs. If so, this property might enable an evolution-guided genome mining strategy to discover polyketides with conserved substructures. Moreover, combinatorial plasticity would constitute a desirable property for the biosynthetic engineering of *trans*-AT PKS megasynthases. Hence, lessons learned from natural recombination and pathway evolution could inform how PKS fragments can be successfully recombined to generate nonnatural PKS assembly lines.

In this work, we present the *trans*-AT PKS Annotation and Comparison Tool (*trans*PACT), an algorithm that automatically assigns *trans*-AT PKS-derived KS domains to functional clades and identifies contiguous series of PKS modules, i.e., module blocks, shared between different PKS assembly lines. We describe the application of *trans*PACT in a global analysis of 1782 *trans*-AT PKS BGCs in publicly available genomes, and show how combinatorial novelty evolved across a wide range of *trans*-AT PKS families from different phyla. Our data reveal cryptic evolutionary relationships between seemingly non-related pathways and demonstrate that, in contrast to *cis*-AT PKS systems, combinatorial assembly is a widespread and general phenomenon in *trans*-AT PKSs. We apply these insights to understand natural product biosynthesis in three plant-associated bacteria and show the utility of *trans*PACT to uncover cryptic evolutionary relationships between distinct polyketide families and to conduct targeted, moiety-based polyketide mining even from fragmented draft genome assemblies. This study provides evidence for the origin of chemical innovation in *trans*-AT PKSs and paves the way toward rational, evolution-inspired engineering of nonnatural hybrid PKSs.

## Results

### Identification of conserved module blocks in annotated BGCs using *trans*PACT

**Fig. 1:**
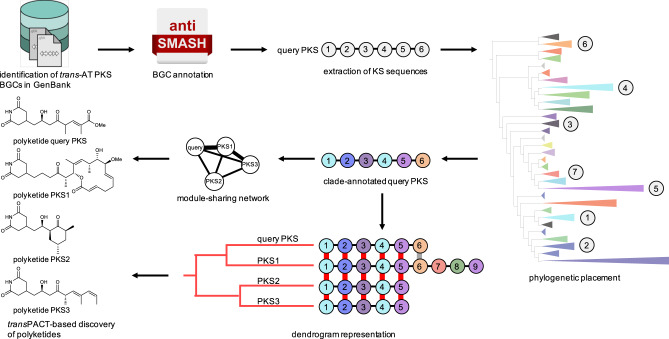
*trans*PACT Workflow. One thousand seven hundred eighty two *trans-*AT PKS BGCs were retrieved from GenBank and subjected to antiSMASH analysis^38^. *Trans*-AT PKS KS sequences were extracted, followed by phylogenetic placement to annotate query KS sequences that correlate with substrate specificity. Module sharing networks and dendrogram representations were computed and used for, e.g., subsequent genome mining studies resulting in the isolation of novel members of *trans*-AT PKS families, the identification of novel *trans*-AT PKS scaffolds, and culturable representatives of uncultured producers of polyketides and evolutionary insights.

To identify signatures of mosaic-like evolution across *trans*-AT PKSs, we developed the computational tool *trans*PACT (Fig. 1). As input for a search algorithm, we generated a maximum-likelihood phylogenetic tree of 647 KS domains (SI Fig. 1) from *trans*-AT PKSs with known products, in which isofunctional clades consisting of KS sequences with shared substrate types (for example, KSs accepting thioesters with β-hydroxyl groups) were manually annotated based on knowledge on the biosynthetic pathways or proposed biosynthetic models^13,16^. *trans*PACT then uses this tree as a reference for pplacer, a maximum-likelihood phylogenetic placement algorithm^32,33^, to assign query sequences to functional clades. Benchmarking on a set of 12 recently published *trans*-AT PKS assembly lines that were not part of the training set showed an accuracy of 81.2%, with regard to the chemistry associated with these functional clades (SI Dataset 1), which is on par with the results of the state-of-the-art algorithm for *trans*-AT PKS-derived polyketide structure prediction TransATor^17^ (82.5%). Thus, transPACT annotates PKS assembly lines with functional clade assignments for each module, which can be used to identify blocks of KS types that recur across different PKSs.

**Fig. 2:**
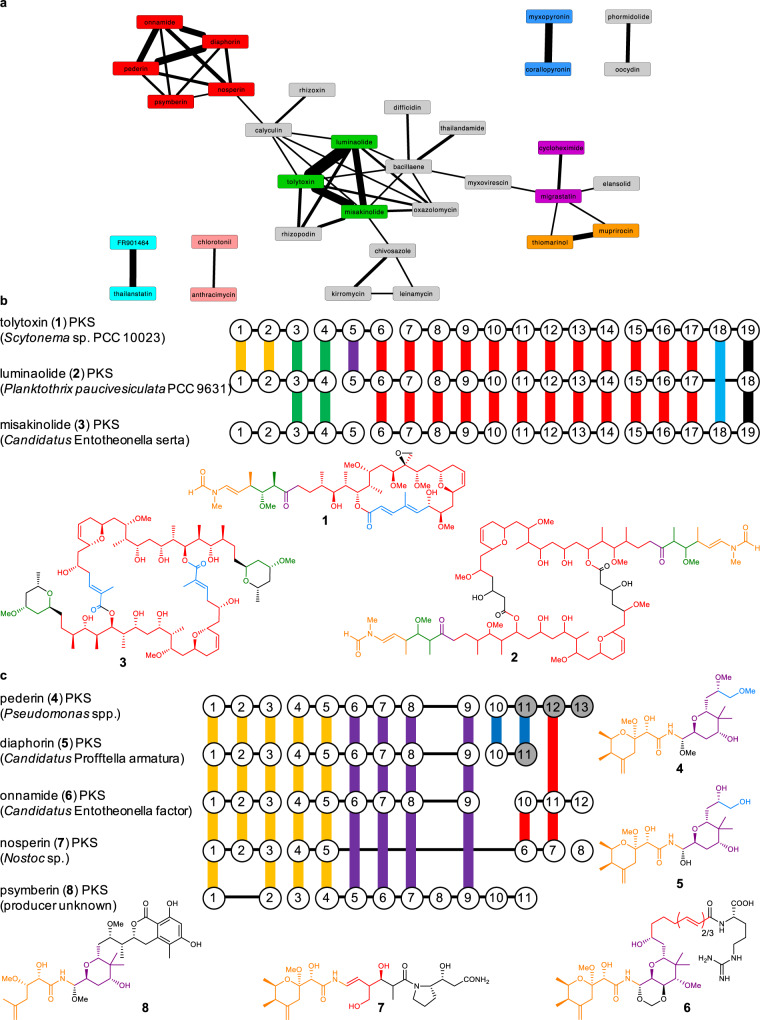
Conserved PKS module blocks (≥2 conserved consecutive modules) identified across all characterized *trans*-AT PKSs and within the misakinolide, and pederin-type PKS families. **a** PKS network of all characterized PKSs that share motifs with other characterized PKSs. The edge line width indicates the number of shared modules. PKS families are color-coded as follows: red: pederin-like PKSs, green: misakinolide-like PKSs, light blue: spliceostatin PKSs, violet: glutarimide PKSs, orange: mupirocin/thiomarinol PKSs, blue: corallopyronin/myxopyronin PKSs, rose: chlorotonil/anthracimycin PKSs, and gray: PKSs that do not belong to a characterized *trans*-AT PKS subfamily. **b** Evolutionary relationship between misakinolide-like PKSs. Conserved PKS module blocks and the corresponding structural moieties of polyketides are shown in the same colors. Vertical lines represent KSs that cluster together, horizontal lines connect domains of proteins. **c** Evolutionary relationship between pederin-type PKSs. Gray circles indicate KSs downstream of putative oxygen insertion events. Oxygen insertion, followed by hydrolysis of the formed ester results in a shortened polyketide. An editable Cytoscape network file is available as source data.

To test the capability of *trans*PACT to identify module blocks that reflect shared chemical substructures in corresponding polyketide products, we initially used all 49 characterized *trans-*AT PKSs reported by Helfrich and Piel^13^ that were included in the reference tree. We found that 46 (94%) out of the 49 known *trans*-AT PKSs shared at least one contiguous pair of modules with another assembly line, and 34 (69%) shared at least two pairs; network analysis based on these data led to the identification of patterns of module sharing between various families of PKSs (Fig. 2a). Analysis of the polyketide structures confirmed that PKSs with shared module blocks generate natural products with common substructures (SI Fig. 2 and SI Table 1). Further validating the *trans*PACT analysis, one of the familial groupings within the network contains the three PKSs for the combinatorial actin-binding macrolides tolytoxin (**1**), luminaolide (**2**), and misakinolide (**3**) (Fig. 2a, b)^29^. Indeed, the detected conserved motifs are in perfect agreement with our previous study showing that large parts of the BGCs have recombined to generate these macrolides^29^. In addition to these reported observations, this analysis also showed that the predicted substrates of KSs present in modules 3 and 4, as well as 6–17 are surprisingly conserved among all three PKSs, indicating that, in the misakinolide PKS, modules 1 and 2, as well as 5 might have been exchanged with other modules during evolution, while modules 3 and 4, as well as 6–17, were retained (Fig. 2b, and SI Figs. 1 and 2).

The BGCs associated with the production of pederin-like compounds are believed to have arisen from a remarkably extensive inter-phylum gene transfer and divergent evolution to yield pederin (**4**) (Gammaproteobacteria)^34^, nosperin (**7**) (Cyanobacteria)^26^, onnamide (**6**) (“Tectomicrobia”)^35,36^, diaphorin (**5**) (Betaproteobacteria)^27^, and psymberin (**8**) (unknown producer)^9,31^. Conservation of module sharing across the pederin-type PKSs indicated three distinct structural motifs, which are reflected in the PKS module architecture (Fig. 2c and SI Fig. 2). The first five modules of the pederin-like PKS family are highly conserved with the exception of the psymberin PKS, which lacks the second module. This conservation on the PKS level is reflected in the highly similar Western parts of pederin-like compounds. The four modules following the first conserved module block in the onnamide PKS have either been excised in the nosperin PKS or have been inserted into a nosperin-like precursor PKS to form the onnamide PKS. In addition, two of the terminal three modules of the onnamide PKS likely fused to the N-terminal part of the nosperin PKS or vice versa. Pederin (**4**) and diaphorin (**5**) are likely biosynthesized via oxygen insertion into the polyketide backbone^27,34^, followed by the hydrolysis of the resulting ester^37^. At the PKS level, a module block stretching the two modules 10 and 11 is present in the pederin and diaphorin PKSs for this oxidative modification^37^ and a subsequent elongation. In the case of the pederin PKS, this bimodule is neighbored by the large module block that is conserved between all pederin-like PKSs on the upstream side and the downstream block of the onnamide PKS that is shared between the pederin, nosperin and onnamide PKSs. The C-terminal modules of the psymberin PKS, however, do not contain KSs shared with any other PKS in this family, suggesting a different origin. The verification of the previously reported evolutionary relationship of the actin inhibitor and pederin-type PKSs by our algorithm indicated that *trans*PACT can detect as-yet undetected combinatorial recombination events in other assembly lines. Moreover, *trans*PACT uncovered cryptic evolutionary relationships between the calyculin and rhizoxin PKSs (Fig. 2 and SI Fig. 2), particularly in a stretch of conserved modules responsible for the biosynthesis of the polyene region of both associated polyketides. Similar cryptic evolutionary relationships were observed between the bacillaene and thailandamide PKSs (likewise the polyene section; Fig. 2 and SI Fig. 2), indicating that *trans*PACT is able to identify previously undiscovered evolutionary relationships even in well-characterized PKSs.

### Widespread transfer of module blocks across *trans*-AT PKSs

To identify combinatorial patterns at a global scale, we compiled all 1782 *trans*-AT PKS assembly lines from genomes publicly available in GenBank (based on antiSMASH 3.0 predictions^38^) and used *trans*PACT to interrogate *trans*-AT PKS evolution across the whole enzyme class (Fig. 1). We first carried out a multistep quality control on the predicted BGCs to make sure that (i) the BGCs contained at least two KS domains (in total, 1509 out of 1782 BGCs were selected for further analysis), and (ii) for each BGC, we excluded *cis*-AT PKS modules containing a KS and an AT domain. For all assembly lines and KS domains that passed these criteria, the pplacer maximum-likelihood phylogenetic placement algorithm^32,33^ implemented into the *trans*PACT workflow was used to place each sequence onto the reference tree to assign them to functional clades. If a KS domain was monophyletic with an annotated isofunctional clade, the annotation was transferred; if not, the KS domain was annotated as a domain of unknown function. Artificially shortened PKS BGCs due to the location of a PKS on a contig border were flagged by measuring the BGC-border distances: in cases where the contig borders were >5 kb away from the nearest KS-encoding region, we assumed that the PKS BGC was complete.

**Fig. 3:**
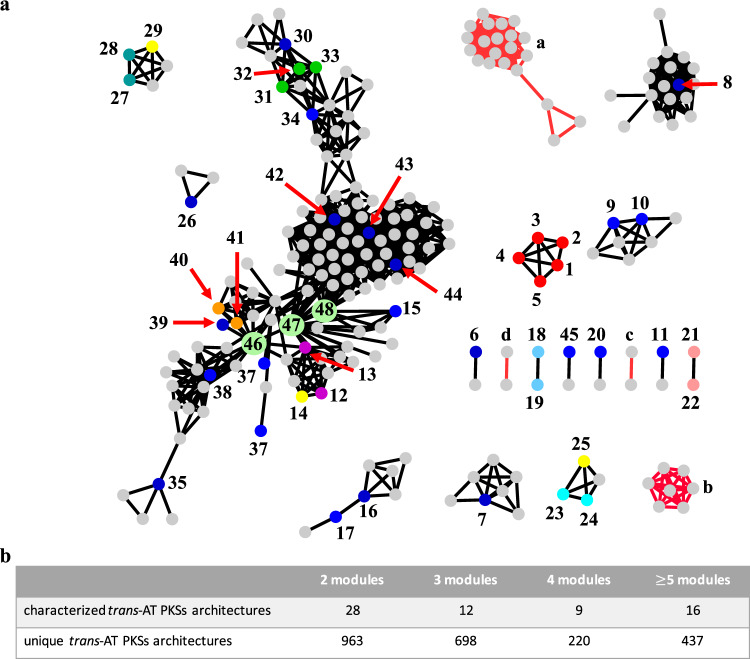
Conserved PKS motifs across all 1782 *trans*-AT PKS BGCs analyzed in this study. **a** Network representation (≥3 conserved consecutive modules). Individual PKSs are represented as nodes and the interconnecting edges indicate module pairs shared by two PKSs. 1: diaphorin; 2: nosperin; 3: onnamide; 4: pederin; 5: psymberin; 6: griseoviridin/virginiamycin, 7: leinamycin; 8: macrolactin; 9: kirromycin; 10: chivosazole; 11: basiliskamide; 12: 9-methylstreptimidone/cycloheximide; 13: migrastatin/dorrigocin; 14: secimide; 15: myxovirescin; 16: rhizoxin; 17: calyculin; 18: myxopyronin; 19: corallopyronin; 20: bryostatin; 21: chlorotonil; 22: anthracimycin; 23: FR901464; 24: thailanstatin; 25: spliceostatin; 26: enacyloxin; 27: oocydin; 28: phormidolide; 29: gynuellalide; 30: rhizopodin; 31: luminaolide; 32: misakinolide; 33: tolytoxin; 34: oxazolomycin; 35: etnangien; 36: batumin/kalimantacin; 37: sorangicin; 38: SIA7248; 39: elansolid; 40: thiomarinol; 41: mupirocin; 42: thailandamide; 43: bacillaene; 44: difficidin; 45: patellazole; 46: *P. durus*|CP009288|c3; 47: *B. gladioli*|CP009322|c8; 48: *G. sunshinyii* YC6258|CP007142|lacunalide. Colored nodes: characterized PKSs as depicted in Fig. 2a; gray: orphan PKSs; yellow nodes: PKSs for which the corresponding polyketide was identified in this study; light green nodes: nodes with a high transitivity (i.e., connected to several other nodes representing different PKS families); red edges: novel module blocks conserved in biosynthetic pathways. a: mainly *Burholderia* spp.; b: mainly *Paenibacillus* spp.; c: *Candidatus* Magnetomorum; d: *Pseudomonas* spp. Note: individual nodes represent unique *trans*-AT PKS architectures, can be composed of multiple GenBank files and might be responsible for the biosynthesis of distinct polyketides (e.g., cycloheximide and 9-methylstreptimidone). **b** Number of shared modules between all characterized PKSs and all 1782 *trans*-AT PKS BGCs analyzed in this study. An editable Cytoscape network file is available as source data.

**Fig. 4:**
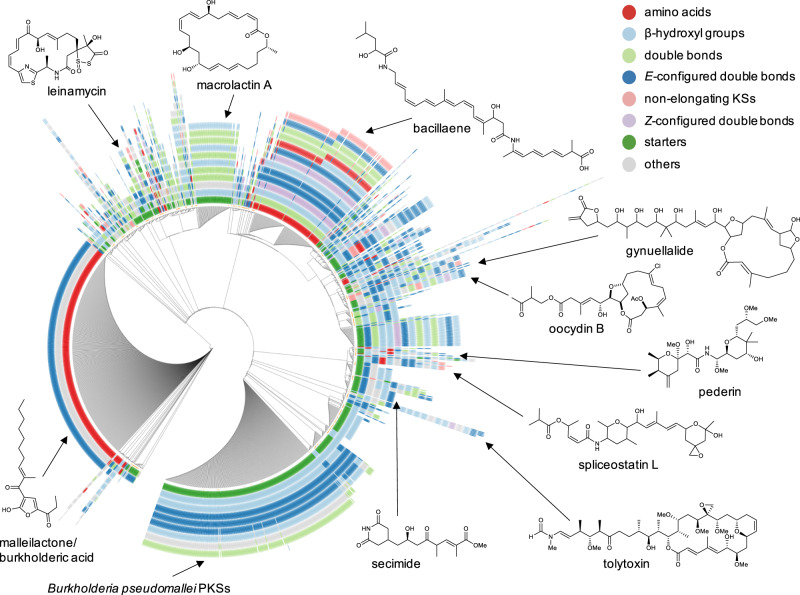
Dendrogram representation of conserved PKS motifs across all 1782 *trans*-AT PKSs analyzed in this study. Colors represent classes of KS clades that correlate with polyketide motifs. Selected polyketides are shown that are either associated with PKSs characterized in this study or belong to large PKS families. An interactive representation of the dendrogram can be accessed here: https://itol.embl.de/tree/474115015487031585082885#.

We then merged this dataset with the reference data and made the dataset nonredundant (removing duplicates of assembly lines that contain KSs grouping with the same clades in the same order). A network visualization of conserved module pair sharing between BGCs (Fig. 3) showed that multiple module blocks are shared by otherwise unrelated assembly lines, indicating widespread evolutionary modularity of *trans*-AT PKSs arising from recombination. We also visualized the architectural relationships in a dendrogram (Fig. 4 and SI Dataset 2), using a distance based on a combination of the Jaccard index of shared KS clades, their copy number differences, and synteny conservation. The color-coding in the dendrogram, which corresponds to the succession of KS types and thus to moieties present in a polyketide, reveals architectural and evolutive patterns across the bacterial kingdom. Together, the network and dendrogram permit rapid exploration of *trans*-AT PKS biosynthetic diversity by accelerating the detection of novel assembly line variants, conserved polyketide motifs, groups of bacteria harboring particularly diverse PKSs, and chemically neglected taxa.

The network analysis identified several patterns that corroborate recent findings in literature, which provided a first confirmation of the correctness of the approach: for example, *trans*PACT identified a significant expansion of the leinamycin family of PKSs with additional variants, which is in agreement with two recent studies describing new members of this PKS family (Fig. 3)^39,40^. Likewise, many other PKS families were expanded by *trans*PACT. In many cases, identical *trans*-AT PKS architectures, each represented by one node in the network visualization (Fig. 3), were identified that occur in multiple members of the same species, related species, or even different genera, indicating the widespread production of the same polyketide scaffold. These PKSs frequently cluster together with similar PKSs to form families that only differ by gene order or by one or few modules, thus giving rise to putatively highly similar polyketide scaffolds (SI Figs. 3 and 4). Abundant *trans*-AT PKS families include those responsible for the biosynthesis of (congeners of) the pathogenicity factor mallailactone/burkholderic acid (>300 PKS assembly lines), macrolactin (54 PKS assembly lines), difficidin (62 PKS assembly lines), and bacillaene (134 PKS assembly lines) (SI Fig. 3). Interestingly, most of these PKS families were identified in individual genera with notable exceptions. Difficidin and bacillaene-like PKSs are almost exclusively encoded in *Bacillus* genomes with the exception of a few *Staphylococcus pneumoniae* genomes, indicating horizontal gene transfer between different families of Firmicutes. While the *Bacillus* species harboring the bacillaene or difficidin PKSs are considered plant-protective^41^, *S. pneumoniae* is a human pathogen. Finally, the analysis suggests that the plant diseases caused by *Ralstonia solanacearum*, which infects a broad range of different agricultural crops^42^, might involve the same pathogenicity factor as in rice seedling blight, i.e., rhizoxins^43^. In accordance with recent studies, *trans*PACT also identified BGCs that are almost identical to the rhizoxin BGC in the genomes of the human pathogen *Chromobacterium violaceum*^44^ and *Ralstonia syzygii* R229^45^. Common features of PKS familiar groupings include the shortening of PKSs (bacillaene, oocydin, thailandamide, glutarimide, and difficidin-type assembly lines), module duplications, or module swaps as the only differences between otherwise identical PKS architectures (e.g., spliceostatin PKSs; SI Figs. 3 and 4). It should be noted though, that such cases should be treated with caution, as misassemblies cannot be fully ruled out, and the original raw reads for the genomes are frequently not publicly available to confirm the correctness of the assemblies easily.

In addition to permitting the rapid identification of conserved polyketide families, the network and dendrogram visualizations suggested extensive transfer of module blocks and formation of chimeric PKSs. For blocks of 2, 3, 4, and 5 or more modules, hundreds of conserved motifs were identified in the 1782 assembly lines within each category (Fig. 3b). Highly conserved module blocks corresponding to polyketide moieties that are particularly common might correlate with evolutionary success, as would be expected for molecule parts imparting bioactivity. One family of *trans*-AT PKSs, responsible for the biosynthesis of glutarimide-containing polyketides was greatly expanded in the dendrogram (Fig. 4a and SI Dataset 2) and the network representation (Fig. 3). Glutarimides are a diverse family of >50 polyketides that harbor the characteristic glutarimide residue^13^. Many of these, such as cycloheximide^46^, are cytotoxins, and some members (i.e., lactimidomycin, iso-migrastatin, and migrastatin)^47^ show promising anticancer bioactivities and serve as drug leads^13,47,48^. In total, our analysis revealed 43 PKSs or PKS fragments as candidates for the production of glutarimide-containing polyketides. While, to our knowledge, such compounds have almost exclusively been reported from *Streptomyces* strains, we found matching glutarimide module blocks in various gammaproteobacterial pathogens, including *Burkholderia gladioli* and *Pseudomonas* spp. (SI Fig. 3). Further predicted glutarimide PKSs were identified in *Magnetospirillum* and *Geobacter*, two genera with poorly understood chemistry (Fig. 4).

We also observed several nodes with a high transitivity (i.e., connected to multiple other network nodes from different PKS families), which suggested the formation of either larger assembly lines from smaller ones or vice versa. Notable examples include the recently characterized lacunalide^49^ PKS in *Gynuella sunshinyii* YC6258 (CP007142) and the metabolically unassigned cluster 3 in *Paenibacillus durus* (CP009288) and cluster 8 in *B. gladioli* (CP009322; Fig. 3, light green nodes).

The network representation also revealed potential outliers, i.e., *trans*-AT PKS assembly lines with products unrelated to known molecules, and therefore with high potential for the discovery of uncharacterized polyketide scaffolds in future genome mining efforts (Fig. 3, red edges). We identified several familial groupings of PKSs (between 2 and 20 distinct PKSs) not containing any reported representative. These PKS families were predominantly observed in *Burkholderia*, *Paenibacillus*, *Pseudomonas*, *Azospirillum, Streptococcus*, and *Bacillus* spp. genomes. It remains to be determined whether these genera are particularly rich sources of undescribed *trans*-AT PKS-derived polyketide scaffolds, or whether the observed numbers are a result of a bias originating from the overrepresentation of the sequenced genomes in GenBank. The identification of shared module blocks in PKSs encoded in these producers shows that *trans*PACT cannot only identify unknown producers of known polyketides, producers of variants of the same scaffold, and evolutionarily successful substructures, but can also be used to prioritize polyketide scaffolds for future genome mining studies (SI Datset 2 and SI Fig. 3).

### *trans*PACT-guided isolation of a new glutarimide-containing polyketide

**Fig. 5:**
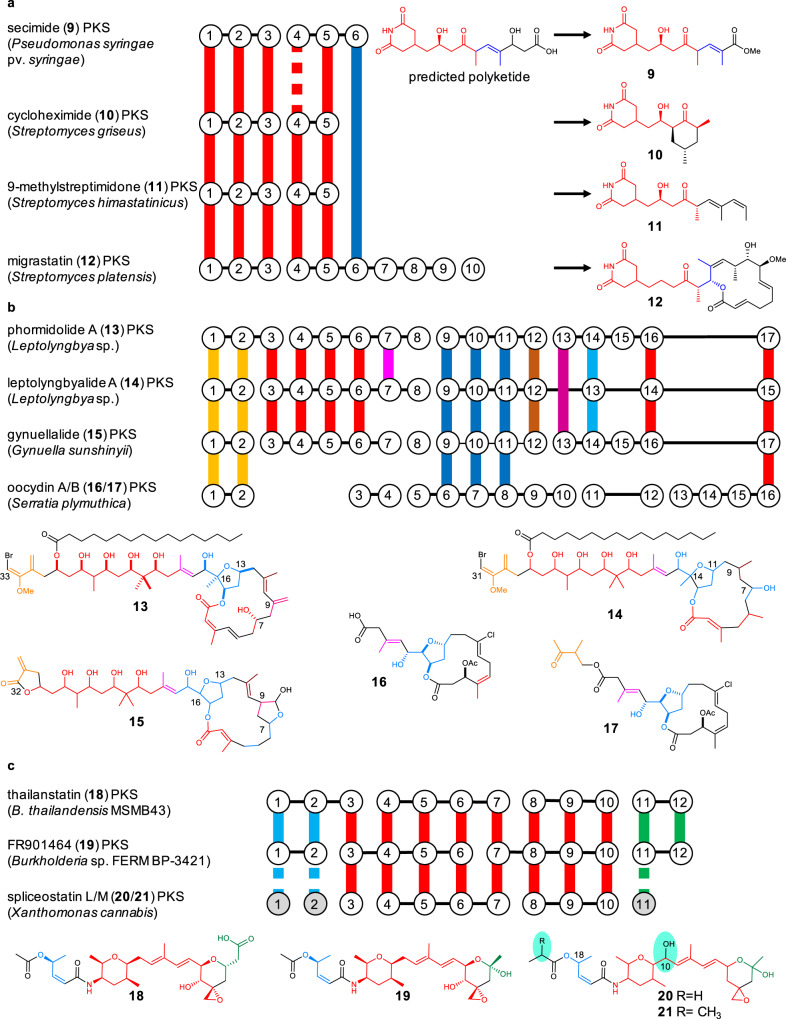
Conserved motifs identified in PKSs for glutarimides, phormidolides, oocydins, and spliceostatins and their corresponding metabolites. **a** Selected glutarimide-type PKSs. **b** Phormidolide-type and oocydin PKSs. **c** Spliceostatin-like PKSs. Conserved PKS module blocks and corresponding polyketide moieties are shown in the same colors. Vertical lines in PKS representations: KSs that cluster together in a phylogenetic tree; black horizontal lines: PKS protein scaffold; gray circles: PKS modules that harbor matching KS domains and domain architecture, but were identified on other, disconnected contigs in the genomic data; discontinuous vertical lines: manually assigned phylogenetic match, green highlights: structural differences compared to previously described spliceostatins.

To test whether our computational approach can lead to functional insights into the chemical diversity of bacteria, we selected a BGC that *trans*PACT predicted to belong to an unreported glutarimide-containing polyketide in the plant pathogen *Pseudomonas syringae* pv. *syringae*, which causes leaf spot diseases in various agricultural crops^50^. Like other PKSs responsible for the biosynthesis of glutarimide-containing polyketides, the selected PKS in this bacterium shows a strong conservation in its first five modules (Figs. 4 and 5a, red), with the exception of module 4 which could not be predicted with high confidence by *trans*PACT. The presence of the glutarimide moiety was corroborated by structural predictions based on KS phylogeny (SI Fig. 4) and TransATor analysis^17^. TransATor is a recently reported genome mining tool that complements *trans*PACT and that can be used for the structural prediction of *trans*-AT PKS-derived polyketides from PKS sequences. Downstream of the fifth module, the glutarimide-type PKSs (and corresponding metabolites) vary extensively, giving rise to dozens of different glutarimide-containing polyketides, of which cycloheximide (**10**)^46^, 9-methylstreptimidone (**11**)^46^, and migrastatin (**12)**^47^ are representatives (Fig. 5a). Our analysis (Fig. 5a) suggested the conserved PKS module block corresponding to the glutarimide residue in the *P. syringae trans*-AT PKS BGC (termed *sec*) is followed by a module that is homologous to migrastatin-like PKSs suggesting that the core structure of the corresponding polyketide likely resembles a truncated version of migrastatin. To test whether the *sec* PKS is indeed responsible for the production of a glutarimide-containing metabolite, *P. syringae* pv. *syringae* was cultivated for chemical analysis.

In initial trials, we were unable to identify a candidate for the predicted structure using various media and culturing conditions. We therefore generated a nonproducing mutant by disruption of the PKS gene *secD* (SI Fig. 5 and SI Table 2). Ultrahigh-performance liquid chromatography–high-resolution electrospray ionization–mass-spectrometry-based (UHPLC-HRESI-MS) comparative metabolomics of the wild-type (WT) and the knockout strains did not reveal differences under standard culturing conditions. However, changing the medium composition to mimic a natural plant environment by addition of fructose triggered the biosynthesis of a compound uniquely present in the WT extracts (SI Fig. 6). Isolation by repetitive rounds of reverse-phase HPLC and structure elucidation by high-resolution MS, 1D and 2D NMR experiments yielded a new polyketide that we termed secimide (**9**) (Fig. 5 and SI Figs. 7–12). The absolute configuration at C8 was determined by Mosher’s ester analysis (SI Figs. 13–23 and SI Table 3). The core structure of secimide resembles a truncated migrastatin-like or an extended cycloheximide-type polyketide as predicted. Notably, however, it is one elongation (C_2_) unit shorter than the prediction, suggesting that either the final module is skipped during biosynthesis (SI Fig. 5, route I), or the polyketide is shortened in a tailoring reaction (SI Fig. 5, route II), perhaps catalyzed by the oxygenase SecA (SI Table 4).

### Unraveling evolutionary relationships between phormidolide- and oocydin-type polyketides

Another subcluster in the network of shared PKS module blocks (Fig. 2) includes the two architecturally related cyanobacterial PKSs for the biosynthesis of phormidolide (**13**) (*phor* PKS) from *Leptolyngbya* sp. ISBN3-Nov-94-8^51^ and leptolyngbyalide (**14**) (*lept* PKS) from *Leptolyngbya* sp. PCC 7375^17^. Surprisingly, the network also indicated the existence of a phormidolide-like PKS in the taxonomically distant halophile *G. sunshinyii* YC6258, a halophilic Oceanospirillales bacterium associated with *Carex* plant roots (Fig. 3b, SI Fig. 24 and SI Table 4)^49,52^. Its PKS (termed the *gyn* PKS) shows a high degree of conservation with the *phor* and *lept* PKSs, sharing 14 out of 17 modules. Results obtained from the *trans*PACT analysis were in good agreement with manual analysis by phylogenetic inference (SI Fig. 4) and TransATor analysis^17^.

To identify the suggested *gyn* product, a 10 L *G. sunshinyii* culture was extracted and analyzed. Using the structure obtained from KS substrate predictions (TransATor analysis^17^) as guidance, a matching candidate metabolite was identified by UHPLC-HRESI-MS. Its molecular formula (C_40_H_62_O_12_), as suggested from the exact mass (*m*/*z* 757.4147 [M + Na]^+^, calc. *m/z* 757.4139 [M + Na]^+^), was close to the prediction (C_41_H_64_O_11_), and hence the corresponding metabolite was selected for purification (SI Fig. 25). The purified compound was characterized by NMR as a new variant of the phormidolide scaffold that we named gynuellalide (**15)** (Fig. 3, SI Figs. 26–31, and SI Table 5). In comparison to the phormidolides, gynuellalide features a hemiacetal moiety at C7–C9, and lacks both the characteristic fatty acid side chain and the methyl group attached to the tetrahydrofuran ring of the phormidolides. Although the core skeleton of phormidolides and gynuellalide differ in the starter moiety around C33/C32, respectively, the remaining regions as well as the KS assignments are remarkably similar, suggesting that the differences between **13** and **15** are the result of post-PKS modifications (Fig. 5b).

We were intrigued that the network node topology suggested the oocydin (*ooc*) PKS from *Serratia plymuthica* to also cluster with these PKSs, with which it shares three conserved PKS motifs spanning multiple modules (Fig. 3b). The *ooc* PKS generates the cytotoxin oocydin A (**16**) as the main product, which we recently found to be a cleaved version of the fully elongated PKS product oocydin B (**17**) retaining a cryptic starter moiety^37^. Oocydins and the cyanobacterial phormidolide-like compounds all share a core structure containing in succession: (i) an exomethylene β-branch, (ii) a tetrahydrofuran-containing moiety, and (iii) a methyl β-branch close to or at the terminus. These are interspersed with variable moieties, depending on the compound. One module block swap has resulted in the replacement by an oxygen insertion module in the *ooc* PKS, generating the unique internal ester moiety of **17**, whereas the first module block in phormidolide-like PKSs is followed by five conserved modules that introduce a series of β-hydroxyl groups. Another large replacement of the C-terminal PKS modules accounts for the distinct macrocyclic portion of the oocydins and phormidolides.

The network and module block data suggest that these shared moieties result from a common evolutionary origin of seven modules. In the case of the cyanobacterial *phor* and *lept* PKSs, the KSs are homologous over the first 12 modules (modules 8 were assigned manually). The remaining five PKS modules in both PKSs differ in modules 13 and 15. Identification of these relationships between oocydins and the three phormidolide-type polyketides illustrates the utility of *trans*PACT to aid not only natural product discovery, but also to identify unusual biosynthetic features. This example of mutually related patchwork-like hybrid polyketides lends further support to a general mechanism of major structural diversification by exchange of module blocks, virtually unknown for *cis*-AT PKSs.

### Identification of spliceostatin variants from a fragmented BGC in the pathogen *Xanthomonas cannabis*

As a final application of *trans*PACT, we mined the highly fragmented genome of another plant-associated bacterium for the production of a *trans*-AT PKS-derived polyketide based on one identified conserved module block. The pathogen *X. cannabis* causes bacterial leaf spot disease in a variety of agricultural crops, characterized by necrotic lesions at the infection sites^53–55^. As it lacks the Hrp type III secretion system and known effectors used by many other plant-invading xanthomonads, its mechanism of pathogenicity remains unknown^55^. Although its draft genome is fragmented into 260 contigs, *trans*PACT analysis rapidly identified a *trans*-AT PKS section clustering with characterized spliceostatin-type PKSs (Figs. 3 and 5a). The congeners thailanstatin A (**18**) and FR901464 (**19**) from the same polyketide family are promising spliceosome-inhibiting anticancer drug candidates^56^ previously isolated from different *Burkholderia* species and *Pseudomonas* sp. 2663, respectively^6,57^. The bioinformatic data suggested *X. cannabis* as an additional producer of spliceostatin-like polyketides. In comparison to the other spliceostatin PKSs, the *X. cannabis* PKS appeared to be incomplete, since only 8 out of the 13 spliceostatin modules were represented (Fig. 5c, SI Fig. 32, and SI Table 6). Manual analysis of contigs harboring *trans*-AT PKS genes enabled us to identify the core PKS BGC putatively responsible for the biosynthesis of spliceostatins (Fig. 5c). Since spliceostatins were reported to be highly potent cytotoxins^6,57^, we speculated that the identified PKS encodes a pathogenicity factor of *X. cannabis*.

UHPLC ESI data-dependent tandem MS in combination with molecular network analysis^58^ of extracts from liquid cultures of *X. cannabis* suggested the presence of more than a dozen spliceostatin analogs (SI Fig. 33). We purified two congeners (spliceostatin L (**20**) and spliceostatin M (**21**)) and characterized them by NMR (Fig. 5c, SI Figs. 34–47, and SI Tables 6 and 7), confirming their identity as additional members of this cytotoxin family. These differ from the known compounds in an α-hydroxylation at the C10 position, as well as in the presence of two distinct acyl ester moieties at the C18 position. **20** was tested for its ability to cause the characteristic leaf lesions using *Arabidopsis thaliana* as a model system. In these leaf assays, **20** caused the formation of lesions resembling the disease phenotype caused by *X. cannabis* (SI Fig. 48). Additional cytotoxicity assays with HeLa cells revealed an IC_50_ value of 5.9 nM for **20** and of 1.4 nM for **21** (SI Fig. 49). These results support a role as a pathogenicity factor for spliceostatin-type compounds in *X. cannabis*.

### Diversity of *trans*-AT PKS assembly lines in metagenome-assembled genomes

The results above were largely obtained through the analysis of genomes of cultivated bacteria. To determine whether further novelty can be found in metagenomes, we studied the functional diversity of a set of 56 *trans*-AT PKS BGCs that we recently identified in 20,584 metagenome-assembled genomes (MAGs)^59^ in the context of the diversity of known *trans*-AT PKS assembly lines (SI Fig. 50). Some of these assembly lines appeared to have a (virtually) identical clade composition to those responsible for the biosynthesis of known polyketides. Interestingly, these results suggest the presence of a BGC for the polyene antibiotic bacillaene, as well as an oocydin-like BGC in MAGs from the human gut, indicating that these molecules might play a role in molecular interactions in the gut microbiome. In addition, the set of MAG-derived BGCs contained several *trans*-AT PKS assembly lines with unprecedented functional clade compositions. Among others, these include two related complex assembly lines of 13/14 modules in length that, according to GTDB-Tk^60^ taxonomy assignments of the associated MAGs, originate from *Ruminococcus* strains. Several assembly line architectures distinct from characterized PKSs were also identified in MAGs originating from other fastidious anaerobic human gut microbes, such as *Blautia producta*, *Anaerocolumna* sp., and a Lachnospiraceae bacterium to which no more detailed taxonomy could be assigned. All in all, these results show the potential of metagenome analysis to identify known *trans*-AT PKS systems in microbiomes of interest, as well as a way to provide new leads for discovery.

## Discussion

A hallmark of polyketides produced by *trans*-AT PKSs is their remarkable structural diversity. In part, this diversity stems from the use of a wide array of enzymatic domains and modules that are rare or undescribed in *cis*-AT PKSs^13,16^. Here, we provide broad evidence of extensive and widespread diversification in *trans*-AT PKSs that results from replacement, insertion, or excision of conserved PKS module blocks. In a global analysis of >1700 *trans*-AT PKS systems, our *trans*PACT analysis showed that the *trans-*AT PKS biosynthetic space consists of several superfamilies, within which extensive module sharing is observed. Many of these superfamilies are connected to each other by some members each sharing conserved consecutive module series with one another. Together, this strongly suggests that extensive combinatorialization and hybridization processes underlie the diversification of these polyketides. Strikingly, little evidence exists for similar mechanisms in *cis*-AT PKS evolution^19^.

Through detection of conserved module blocks and identification of cryptic relationships, *trans*PACT aids natural product discovery in several ways. In three case studies, we demonstrate its utility as a mining tool for natural products using plant-associated bacteria as examples. In the case of gynuellalide, cryptic evolutionary relationships between phormidolide- and oocydin-type polyketides were identified. Interestingly, and despite the fact that the gynuellalide PKS is almost identical to the phormidolide PKS, the corresponding polyketide shows remarkable differences, which are likely due to extensive post-PKS modifications.

In addition to identifying cryptic evolutionary relationships between PKS families, we show that the algorithm also allows sequence-based predictions of chemical features in highly fragmented genomes. In particular for large PKS and NRPS BGCs, insufficient sequence coverage and the presence of repetitive regions in multimodular enzyme-coding genes severely compromise efforts to assemble them contiguously, which makes it difficult to predict their functions^61^. The automated comparative genomic analysis provided by *trans*PACT can address this challenge. In this context, the prediction of spliceostatins from the fragmented *X. cannabis* genome yielded two potent analogs for this group of drug candidates. Their activity in leaf necrosis assays suggests an ecological function as pathogenicity factors. Other spliceosome inhibitors like herboxidiene and pladienolide were shown to exhibit herbicidal or plant growth-retardation activities^62,63^. However, since the target of the new polyketides has not been experimentally verified, further studies are needed to investigate their role as spliceosome inhibitors in the context of plant pathogenesis.

Moreover, we used *trans*PACT for the targeted expansion of the glutarimide-containing family of polyketides. The *sec* locus is conserved in multiple plant pathogenic bacteria and is responsible for the biosynthesis of secimide, a glutarimide-containing polyketide that resembles a truncated version of migrastatin-type polyketides. Its corresponding PKS is very similar to the migrastatin PKS over the first six modules. In comparison to the migrastatin PKS, the final four modules are missing. This observation indicates that a PKS family can be expanded through the addition or deletion of module blocks from preexisting, functional PKSs and the resulting PKSs remain functional.

The identification of conserved PKS module blocks that correspond to shared polyketide moieties enhances our understanding of natural product evolution, and suggests implications for drug discovery and PKS engineering. Why is module block swapping prevalent in *trans*-AT PKSs? Exchange of larger structural moieties provides a strong force of polyketide innovation compared to module duplication as known for *cis*-AT PKSs, thus permitting polyketide evolution “in quantum leaps”. Such polyketide moieties might be maintained and combinatorially used for structural reasons, such as permitting closure of (macrocyclic) rings, mediating recognition of the metabolite by exporters, or conferring pharmacological activity. Support for pharmacophores as selection forces for module blocks is provided by actin-binding macrolides, which use an exocyclic tail moiety for target binding^64^ that recurs as a combinatorial-like unit in several otherwise unrelated congeners^29^. In the diverse family of pederin-type polyketides, a large shared moiety confers cytotoxic activity, while swapped parts further modulate the bioactivity profile, including potent dermatotoxicity only in congeners with lipophilic termini^65^. It would be worthwhile to test whether the conserved tetrahydrofuran portion in the cytotoxic oocydins and phormidolides are likewise determinants of bioactivity. Extrapolated to natural product mining, the use of *trans*PACT to target uncharacterized but widely conserved module blocks could be an interesting rationale to discover evolutionarily successful bioactive principles in nature. We have made *trans*PACT available as an open-source software, which scientists can apply on their own custom (meta)genomic datasets^66^.

In addition to keeping a pharmacophore intact, preserved module blocks could be important at the biochemical level as the successful recombination of module blocks might frequently result in functional chimeric PKSs. *trans*PACT revealed dozens of such putative successful recombination events. The extent of module block recombination is remarkable, as putatively hybridized PKS assembly lines are observed across phylum borders in numerous instances. However, we cannot exclude that recombination occurred between ancestral clusters present in the same source taxon, followed by transfer of whole clusters to other phyla. Previous studies by one of our labs has located recombination sites in *trans*-AT systems behind KS domains^29,31^, supporting the redefinition of a biosynthetic module as also proposed by others for *cis*-AT PKSs^67,68^. Classically, the KS and ACP domains have been defined as module boundaries. From an evolutionary and biochemical perspective, however, a module would more logically start downstream of an (in *trans*-AT PKSs often substrate-specific) KS domain and end with the next KS, which phylogenetically and biochemically matches the modification introduced by this module^68^. Recombination between different *trans*-AT PKSs behind specific KSs would be expected to more likely result in functional PKSs than for traditional module boundaries. Identifying the exact recombination loci as well as focusing on evolutionarily successful module blocks will provide important clues for rationally engineering functional hybrid *trans*-AT PKSs by module block fusion. Moreover, rational excisions and insertions could be designed once the exact locations of functional recombination events have been defined.

In summary, our analysis groups *trans*-AT PKSs into three major categories. The first category consists of PKSs that are conserved in a variety of different producers and putatively responsible for the biosynthesis of identical or closely related polyketides. While this result shows the potential of *trans*PACT to conduct in silico dereplication studies even for fragmented (meta)genomes, it also offers the possibility to identify cultured sources for polyketides initially isolated from uncultured bacteria (i.e., luminaolide)^26,29^. This strategy could be relevant for biotechnological production, since many bioactive *trans*-AT PKS products have been isolated from uncultured sources^8,13^. The second PKS category comprises enzymes that harbor a conserved module block corresponding to a shared polyketide substructure. Targeting these polyketides might lead to the identification of polyketides with pharmacophores within otherwise highly dissimilar scaffolds, such as in the case of the glutarimide polyketides studied here. Thirdly, the existence of common motifs present only in orphan PKSs (unlabeled node groups in Fig. 2) point to a large potential to identify biosynthetic diversity in future genome mining studies.

## Methods

### *Trans*-AT PKS assembly line identification, annotation, and phylogenetic analysis

The set of characterized *trans*-AT PKS assembly lines^16,17^ was manually gathered from MIBiG^69^ and literature; the set of predicted *trans*-AT PKS assembly lines was gathered from antiSMASH^38^ results against the entirety of GenBank (version of December 2015). KS domains in all assembly lines were predicted using the SMART^70^-derived PKS_KS.hmm domain, using HMMer v2 instead of v3 to avoid undue gaps inside the hits. Domains from *cis*-AT modules were filtered out by checking for hits with the PKS_AT.hmm model. Domains were aligned using Muscle in MEGA v6 and a phylogenetic tree was constructed with RAxML, under a GTRGAMMA substitution model. Functional clades in the reference tree were assigned manually, and phylogenetic placement of predicted KS domains was performed using pplacer v1.1.6^33^.

### Prediction of the order of KS domains across PKS assembly lines

For KS domains that are exclusively encoded on the positive or negative strand, the KS domains were ordered according to their genomic location (1228 BGCs are encoded exclusively on the same strand orientation). For KS domains that are encoded on both positive (+) and negative strands (–), we separated them into two situations: partial mixture and complete mixture. Partial mixture describes the situation where all domains encoded on positive strands have a genomic location larger (or smaller) than the largest (or smallest) genomic location of those on the negative strands (−−−+++ or +++−−−). Complete mixture is the situation where at least one domain on the positive strand is located in between the ones on the negative strand or vice versa (−−+−++). For partial mixture, if a TE domain, which is considered to terminate biosynthesis, is identified and the TE domain is located next to the terminal KS domain on one strand, the biosynthesis is considered to start from the KS domains on the other strand (times of correction: 91 TEs on negative strand, 96 TEs on positive strand). If no TE domain is identified, the KS domains on the two strands are indexed separately. For complete mixture, which only happened three times, we did not index the KS domains. TE domains are identified with Pfam TC trusted threshold cutoffs in HMMER v2.

### Organisms and culture conditions

Seed cultures of *Xanthomonas campestris* pv. *cannabis* (*X. cannabis*) were prepared by inoculating 10 mL peptone–sucrose (PS) medium in baffled Erlenmeyer flasks from cryo-stocks of *X. cannabis*. After cultivation at 30 °C overnight at 200 rounds per minute (r.p.m.), 1 L baffled Erlenmeyer flasks (17×) containing 200 mL PS medium were inoculated with 400 µL of seed culture. Bacteria were incubated at 30 °C for 40 h at 200 r.p.m. *Pseudomonas syringae* B728a was cultured in SR-minimal medium supplemented with 0.1% fructose and 100 µM arbutin in baffled Erlenmeyer flasks at 20 °C for 4 days. *Gynuella sunshinyii* was cultured in marine broth 2216 in 1 L ultrahigh yield flasks for 3 days at 30 °C at 120 r.p.m.

### Construction of a secimide production deficient mutant

An unmarked deletion mutant of Psyr_4314 was constructed as described previously^71^. Briefly, the 5′ and 3′ regions flanking Psyr_4314 were amplified in a first round of PCR reactions, in addition to a kanamycin resistance cassette flanked with FRT sites from pKD13.8 The primers 4314-F1 and 4314-R1 were used to amplify the region upstream of Psyr_4314, while 4314-F2 and 4314-R2 were used to amplify the region downstream of Psyr_4314, and FRT-KM-F and FRT-KM-R were used to amplify the FRT-flanked kanamycin resistance cassette (Supplementary Table 9). In a subsequent PCR, all three fragments were combined and amplified for 15 cycles without added primers, followed by addition of 4314-F1 and 4314-R2 for 20 more PCR cycles to amplify the combined fragment. The resulting PCR product was cloned into the suicide vector pTOK2T9. The obtained construct was subsequently transferred into *P. syringae* by tri-parental mating^10^ and transformants selected on KB plates containing rifampin, kanamycin, and tetracycline. Double crossover mutants as indicated by kanamycin resistance, but tetracycline sensitivity were subsequently identified and the *kan* cassette excised. Excising was conducted by introducing plasmid pFLP211 in which the omega fragment conferring spectinomycin resistance had been added. Replica plating was used to cure the Δ4314 strain of pFLP2-omega. Successful markerless deletions were confirmed by PCR.

### Detection of secimide

A total of 200 mL cultures of *P. syringae* B728a WT and a *sec* knockout strain were harvested by centrifugation. The supernatants of both cultures were extracted with EtOAc, and the organic layers concentrated in vacuo. The pellet was extracted with acetone, and also concentrated in vacuo. All extracts were dissolved in MeOH, and subjected to HR-LCMS analysis (5% MeCN for 5 min, 5–100% MeCN gradient for 30 min, washed with 100% MeCN, column: Phenomenex Kinetex 2.6 µm XB-C18 100 Å, 4.6 × 150 mm, UV detection at λ = 220 nm, column oven: 27 °C). The most significant difference between WT and knockout in the LCMS chromatogram was attributed to one compound with the *m*/*z* of 326.16 eluting after 16–18 min that only appeared in the WT supernatant.

### Isolation and structure elucidation of secimide

A 1 L culture of *P. syringae* B728a was centrifuged, and the supernatant was extracted with EtOAc. The organic layer was concentrated in vacuo, and separated by reversed-phase HPLC (Phenomenex Luna 5 µm C18, 10 × 250 mm, UV detection at *l* = 220 nm, room temperature (r.t.)) eluted with 5% MeCN for 5 min, then a gradient from 5% MeCN to 100% MeCN for 30 min, and 100% MeCN for 25 min. The fraction containing secimide was further separated by reversed-phase HPLC (Phenomenex Luna 5 µm Phenylhexyl, 10 × 250 mm, UV detection at *λ* = 220 nm, r.t.) eluted with 20% MeCN to afford 0.4 mg of secimide.

Secimide has a molecular formula of C_16_H_23_NO_6_, which was suggested by HR-LC-ESIMS (*m/z* 326.1597, [M + H]^+^, Δ −0.1 m.m.u.). Analysis of the ^1^H NMR spectrum in conjunction with the HSQC data revealed the presence of one protonated sp^2^ carbon, one methoxymethyl, one oxygenated methine, one doublet methyl, one vinyl methyl, two methines and four methylenes. Unit **I** and unit **II** were determined by COSY analysis. The HMBC correlations from H7, H10, and H11 to C8 suggested that unit **I** was connected to the methyl ester moiety. The HMBC correlations from H6 to C9, and from H9 to C5 and C6 suggested that the vinyl methyl group was connected to unit **I**. Unit **II** and this vinyl methyl group were connected via a ketone carbon (C4) based on the HMBC correlations from H3, H9, and H6 to C4. The piperidine-2,6-dione moiety was determined based on the HMBC correlations from H2′ to C3′and C6′, from H6′ to C2′ and C5′, as well as the molecular formula.

The 5*E* geometry of secimide was determined by NOESY correlations between H7 and H9. The absolute stereochemistry of C2 was determined as *R* by applying the modified Mosher’s method.

### Preparation of secimide MTPA esters

A total of 1 mg of secimide was added to *S*-(+)- or *R*-(−)-MTPACl (5 µL) in 100 µL of CH_2_Cl_2_ containing 1 mg of DMAP for 2 h at 40 °C. The mixture was partitioned between 0.1 M NaHCO_3_ and CHCl_3_. The organic layer was washed with 0.1 M HCl and H_2_O, and then concentrated in vacuo. The extract was separated by reversed-phase HPLC (Phenomenex Luna 5 µm C18, 10 × 250 mm, UV detection at λ = 220 nm, r.t.) eluted with 5% MeCN for 5 min, then a gradient from 5% MeCN to 100% MeCN for 30 min, and 100% MeCN for 25 min to afford *R*-(+)- or *S*-(−)-MTPA esters, respectively.

### Isolation and structure elucidation of gynuellalide

A 10 L culture of *G. sunshinyii* was centrifuged and the supernatant extracted with EtOAc. The extract was concentrated in vacuo, and the crude extract was separated by reversed-phase HPLC (Phenomenex Luna 5 µm C18, 20 × 250 mm, UV detection at *l* = 200 nm, r.t.), using the following gradient: 5% MeCN for 5 min, then a gradient from 5% MeCN to 100% MeCN for 30 min, and 100% MeCN for 25 min. The fractions containing gynuellalide were further separated by reversed-phase HPLC (Phenomenex Synergi Hydro-RP 5 µm C18, 10 × 250 mm, UV detection at λ = 200 nm, r.t.), using an isocratic solvent composition of 30% MeCN. Purification was achieved by reversed-phase HPLC (Phenomenex Luna 5 µm Phenylhexyl, 10 × 250 mm, UV detection at *λ* = 200 nm, r.t.) using an isocratic elution profile with 35% MeCN + 0.1% FA to afford 0.3 mg of gynuellalide.

Gynuellalide had a molecular formula of C_16_H_23_NO_6_, which was suggested by HR-LC-ESIMS (*m/z* 757.4147 [M + Na]^+^, Δ +1.3 m.m.u.). Analysis of the ^1^H NMR spectrum in conjunction with the HSQC data revealed the presence of one exomethylene, two singlet aliphatic methyls, one doublet aliphatic methyl, three vinylic methyls, eight oxygenated methines, one dioxygenated methine, and three protonated sp2 carbons. COSY spectrum deduced the partial structures **I**–**VII**. Unit **I** and unit **II** was connected by HMBC correlations from H39 to C25 and from OH25 to C26. Unit **II** was connected to unit **III** via a dimethyl quaternary carbon, which was determined by HMBC correlations from OH21 to C22, from H23 to C21, from OH23 to C22, from H37 to C21, C22, C23 and C38, and from H38 to C21, C22, C23, and C37. Unit **III** and unit **IV** was connected by HMBC correlations from H18 to C20, from H20 to C18, C19, and C20, and from H36 to C20. The ether ring in unit **IV** was determined by a weak HMBC correlation from H13 to C16. Unit **IV** and unit **V** were connected by HMBC correlations from H10 to C12, from H12 to C11 and C35, and from H35 to C12. A hemiacetal moiety formed by unit **V** and unit **VI** was supported by HMBC correlations from H8 to C34, from H9 to C34, from H34 to C7, C8, C9, and C10, and from OH34 to C9. Unit **V** was connected to unit **VII** by HMBC correlations from H2 to C4, from H4 to C2, C3, and C33, from H5 to C3, and from H33 to C4. The macrolide was formed between unit **IV** and unit **VII** by HMBC correlations from H2 and H15 to C1. Finally, the five-membered lactone moiety with the exomethylene group was attached to the terminus of unit **I**, as determined by the molecular formula and HMBC correlations from H30 to C31, C32, and C40, and from H40 to C30 and C32.

The 2*E*,10*E*,18*E* geometry was determined by ROESY correlations between H2 and H4, between H9 and H35, between H10 and H12, and between H17 and H36. The relative stereochemistry of C7 and C9 was determined as 7 *R*′,9*R*′ by ROESY correlations between H6 and H7a, between H7a and H10, and between H6 and H10. The relative stereochemistry of the ether ring was determined as 13 *S*′,15 *S*′,16 *R*′ by ROESY correlations between H12 and H15, between H12 and H17, between H13 and H16, and between H15 and H17.

### Isolation of spliceostatins

*Xanthomonas cannabis* cultures were incubated at 30 °C for 40 h at 200 r.p.m. After centrifugation, the highly viscous supernatant was extracted once with 80% (v/v) EtOAc/water, then with 40% (v/v) EtOAc/water. The organic layers were combined and the solvent removed under reduced pressure to yield 250 mg of crude extract. The residue was dissolved in 2 mL of acetonitrile and stored at −20 °C. The precipitate was removed once a day for 5 days. The clear solution was purified on an Agilent 1260 Infinity HPLC using a Kinetex C18 100 Å column (mobile phase A water, mobile phase B acetonitrile; flow rate 2.0 mL/min; gradient, 5–35% B for 8 min, 35–95% B for 32 min, 95% B for 8 min, 95–5% B for 3 min, 5% B for 1 min). The collected fractions were analyzed on a Thermo Orbitrap UPLC-HESI-MS. A total of 0.2 mg of **20** and 0.2 mg of **21** were obtained and the structures characterized by 1D and 2D NMR experiments.

### Structure elucidation of spliceostatin M (21)

The molecular formula of C_29_H_45_NO_8_ was predicted based on HR-ESIMS spectroscopy. The HMBC correlations from H2 and H17 to the chemical shift of 97.2 p.p.m. characteristic for an acetal, together with the HMBC correlations from the alcohol at 3.71 to C1, C2, and C17, were used to determine the hydroxylation of C1. Based on HMBC correlations from H2 to C3 and H18 to C2, C3, and C4 the epoxide was connected. The coupling constant of H6 and H7 (3JH-6,H-7 = 15.8 Hz) suggests *trans*-configuration. The NOESY spectra shows cross peaks between H9 and H7, as well as H10 and H19 suggesting *trans*-configuration for the double bond between C8 and C9. The COSY correlation of the alcohol at 2.95 to H10 and the HMBC correlation of it to C9, C10, and C11 suggest hydroxylation of C10. C12 and C13 were connected by HMBC correlations of their respective protons. HMBC correlations of NH toward C1′ and C14 connect the two parts of the molecule through a peptide bond. The coupling constant of H2′ and H3′ (3JH-2′,H-3′ = 11.7 Hz) suggests *cis*-configuration. HMBC correlations of H4′ to C1″ connect the isobutyrate group to the molecule. The relative stereochemistry of the Western pyran ring was determined by analysis of the NOESY spectrum. The axial protons H11 and H15 show cross peaks, as well as NH with C16 and C20. The relative stereochemistry of the Eastern pyran ring could not be resolved due to a lack of correlation signals between H2 and H4.

### Structure elucidation of spliceostatin L (20)

The molecular formula of C_28_H_43_NO_8_ was predicted based on HR-ESIMS spectroscopy. The HMBC correlations from H2 and H17 to the for an acetal characteristic chemical shift of 97.2 ppm together with the HMBC correlations from the alcohol at 3.72 to C1, C2, and C17 were used to determine the hydroxylation of C1. Based on HMBC correlations from H2 to C3, and H18 to C2, C3, and C4, the epoxide was connected. The coupling constant of H6 and H7 (3JH-6,H-7 = 15.8 Hz) suggests *trans*-configuration. The NOESY spectra shows cross peaks between H9 and H7 as well as H10 and H19 suggesting *trans*-configuration for the double bond between C8 and C9. The COSY correlation of the alcohol at 2.95 to H10 and the HMBC correlation of it to C9, C10, and C11 suggest hydroxylation of C10. C12 and C13 were connected by HMBC correlations of their respective protons. HMBC correlations of NH toward C1′ and C14 connect the two parts of the molecule through a peptide bond. The coupling constant of H2′ and H3′ (3JH-2′,H-3′ = 11.7 Hz) suggests *cis*-configuration. HMBC correlations of H4′ to C1″ connect the propionate group to the molecule. The relative stereochemistry of the Western pyran ring was determined by analysis of the NOESY spectrum. The axial protons H11 and H15 show cross peaks, as well as NH with C16 and C20. The relative stereochemistry of the Eastern pyran ring could not be resolved due to a lack of correlation signals between H2 and H4.

### Instrumentation and software

NMR spectra were recorded on a Bruker Avance III spectrometer equipped with a cold probe at 500 or 600 MHz for ^1^H NMR, and 125 or 150 MHz for ^13^C NMR. Chemical shifts were referenced to the solvent peak at δ_H_ 7.27 and δ_C_ 77.23 for chloroform-*d*, δ_H_ 2.50 and δ_C_ 39.51 for DMSO-*d6*, and δ_H_ 1.94 and δ_C_ 1.39 for acetonitrile-*d3*. UHPLC-HRESI-MS was performed on a Thermo Scientific Q Exactive mass spectrometer. Software versions used for data analysis are Xcalibur 4.1 (Thermo) and Chromeleon Xpress version 7.2 (Thermo) for MS, Open Lab CDS version 2.2 (Agilent) for HPLC, and TopSpin version 3.5.7 (Bruker) for NMR.

### Cultivation of *A. thaliana*

*Arabidopsis thaliana* Col-0 seeds were prepared as described by Innerebner et al.^72^. Plants were cultivated in full-gas 24-well plates on Murashige Skoog-medium9 (MS-medium) at 22 °C in a climate chamber. After 1 week under long-day conditions (16 h photoperiod) the photoperiod was reduced to short-day conditions (9 h photoperiod).

### Inoculation of *A. thaliana* with *X. cannabis* and spliceostatins

*Xanthomonas campestris* pv. *cannabis* was grown in PS medium overnight. Cells were harvested and the pellet was washed once with water, resuspended in water, and diluted to an optical density of OD_600_ of 0.5. **20** was dissolved in 10% acetonitrile in water to obtain a concentration of 1.8 mM. Solutions with the following concentrations were prepared 180, 18, 1.8, and 0.18 µM by dilution with water. In 24-well plates, 1 µL of each solution was spotted onto one leaf of three plants, respectively, water was chosen as a negative control, *X. cannabis* suspension as a positive control. The experiment was performed on three independent 24-well plates. After inoculation with bacteria and treatment with **20**, the plants were cultivated under short-day conditions (9 h photoperiod). Pictures were taken directly after inoculation (0 d.p.i.) and 10 days post inoculation (10 d.p.i.).

### HeLa cell cytotoxicity assay

HeLa cells were cultivated at 37 °C, 5% (v/v) CO_2_ for 3–4 days. HeLa cells were washed twice with PBS buffer (Sigma D8537), 2 mL of 0.05% trypsin-EDTA solution (Thermo 25300-054) were added, and the plate was incubated for 5 min at 37 °C. The cells were resuspended in 10 mL medium (DMEM-GlutaMAX) supplemented with 10% FCS (Eurobio CVFSVF00-01), 1% nonessential amino acids (Thermo 11140-035), and 50 µg/mL gentamicin and centrifuged for 5 min, 110 r.c.f. at r.t. The supernatant was discarded and the pellet resuspended in 10 mL medium. Cells were counted by analyzing 10 µL of the suspension under a ZEISS Axiovert 25 microscope using a Neubauer hemocytometer. A 5000 cells/mL suspension was prepared and 200 µL were transferred into each well of a 96-well plate. After 3 days of cultivation, 2 µL sample were added to the lanes B2-11. DMSO was used as a negative control, doxorubicin (*c* = 1 mg/mL in DMSO) as a positive control. Both were measured in triplicates. Isolated spliceostatin L (**20**) and spliceostatin M (**21**) were used at a concentration of *c* = 3 µM in DMSO in quadruplicates. A total of 50 µL medium were added to the wells in row B, mixed by pipetting and transferred to the next row. This procedure was repeated until row G, thereby diluting the concentration of the samples five times. After 3 days of cultivation, 50 µL of 3-(4,5-dimethylthiazol-2-yl)-2,5-diphenyltetrazolium bromide (1 mg/mL in sterile H_2_O) were added and the cells incubated for 3 h at 37 °C. The supernatant was discarded and 150 µL of DMSO were added to the wells. The absorbance at 570 nm was measured on a spectraMAXplus spectrometer (Molecular Devices LLC). A 96-well plate with DMSO served as a blank, all measurements were performed in technical triplicates. Relative and absolute IC_50_ values were calculated by fitting a four-parameter logistic model to the mean of the biological replicates in GraphPad Prism 7. For determination of the absolute IC_50_, data were normalized by averaging the absorbance values measured at highest doxorubicin concentrations and setting it as the bottom of the curve (bottom constrained to 0.0), and the three negative control values containing the least DMSO and setting it as the top of the curve (top constrained to 1.0).

### Reporting summary

Further information on research design is available in the Nature Research Reporting Summary linked to this article.

## Supplementary information

Supplementary Information

Description of Additional Supplementary Files

Supplementary Dataset 1

Supplementary Dataset 2

Reporting Summary

## Data Availability

GenBank: the spliceostatin gene cluster from *X. cannabis* was deposited in GenBank under accession number BK010647. MIBiG: the spliceostatin, gynuellalide, and secimide BGCs were deposited in MIBiG under accession numbers BGC0002059, BGC0001835, and BGC0002060. All other raw data are available from the corresponding authors upon request. Source data are provided with this paper.

## Source data

Source Data

## Code availability

The transPACT source code is available freely under an open-source AGPL-3.0 license from https://github.com/chevrm/transPACT (10.5281/zenodo.4148258).
