# Supplementary material for: Evolution of combinatorial diversity in trans-acyltransferase polyketide synthase assembly lines across bacteria
Source: Nat Commun. 2021 Mar 3;12:1422. doi: 10.1038/s41467-021-21163-x (PMC7930024; doi:10.1038/s41467-021-21163-x)
Supplement: Supplementary file 1 — Supplementary Information [file 41467_2021_21163_MOESM1_ESM.pdf]

Supplementary Information

Evolution of Combinatorial Diversity in *Trans*-Acyltransferase Polyketide Synthase Assembly Lines Across Bacteria

Eric J. N. Helfrich,[1,2#] Reiko Ueoka,[1#] Marc G. Chevrette,[3#] Franziska Hemmerling,[1] Xiaowen Lu,[4] Stefan Leopold-Messer,[1] Hannah A. Minas,[1] Adrien Y. Burch,[5] Steven E. Lindow,[5] Jörn Piel,[1]* and Marnix H. Medema[4]*

[1]Institute of Microbiology, Eidgenössische Technische Hochschule (ETH) Zurich, Vladimir-Prelog-Weg 4, 8093 Zurich, Switzerland.

[2] Institute for Molecular Bioscience, Goethe University Frankfurt, Max-von-Laue-Straße 9, 60438 Frankfurt am Main, Germany.

[3]Wisconsin Institute for Discovery, Department of Plant Pathology, University of Wisconsin-Madison, 330 N. Orchard Street, Madison, WI 53715, USA.

[3]Bioinformatics Group, Wageningen University, Droevendaalsesteeg 1, 6708PB Wageningen, The Netherlands.

[4]Department of Plant and Microbial Biology, University of California at Berkeley, Berkeley, California 94720, USA.

# authors contributed equally to this work

* Correspondence should be addressed to JP (jpiel@ethz.ch) and MHM (marnix.medema@wur.nl)

Supplementary Figures

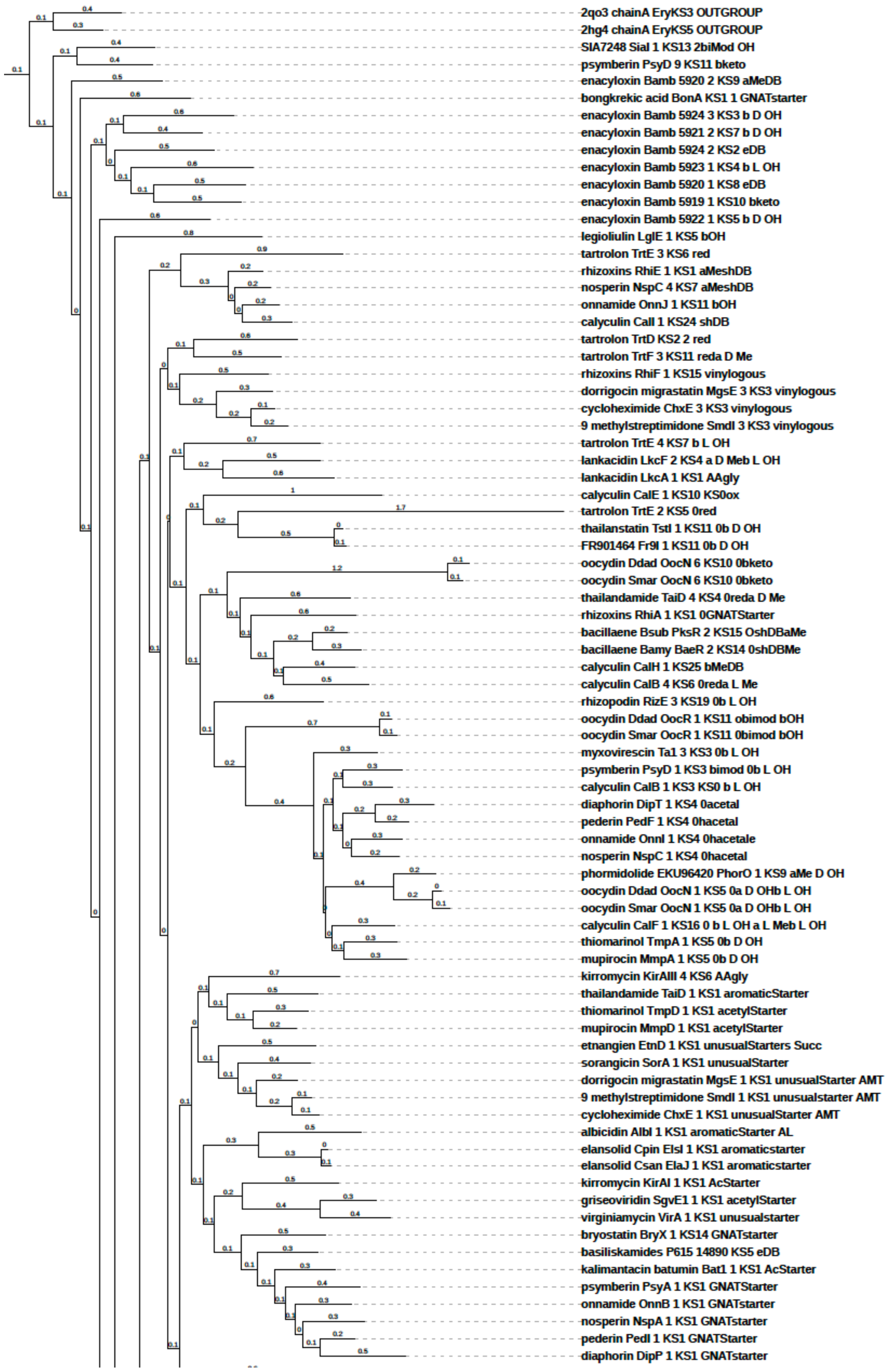

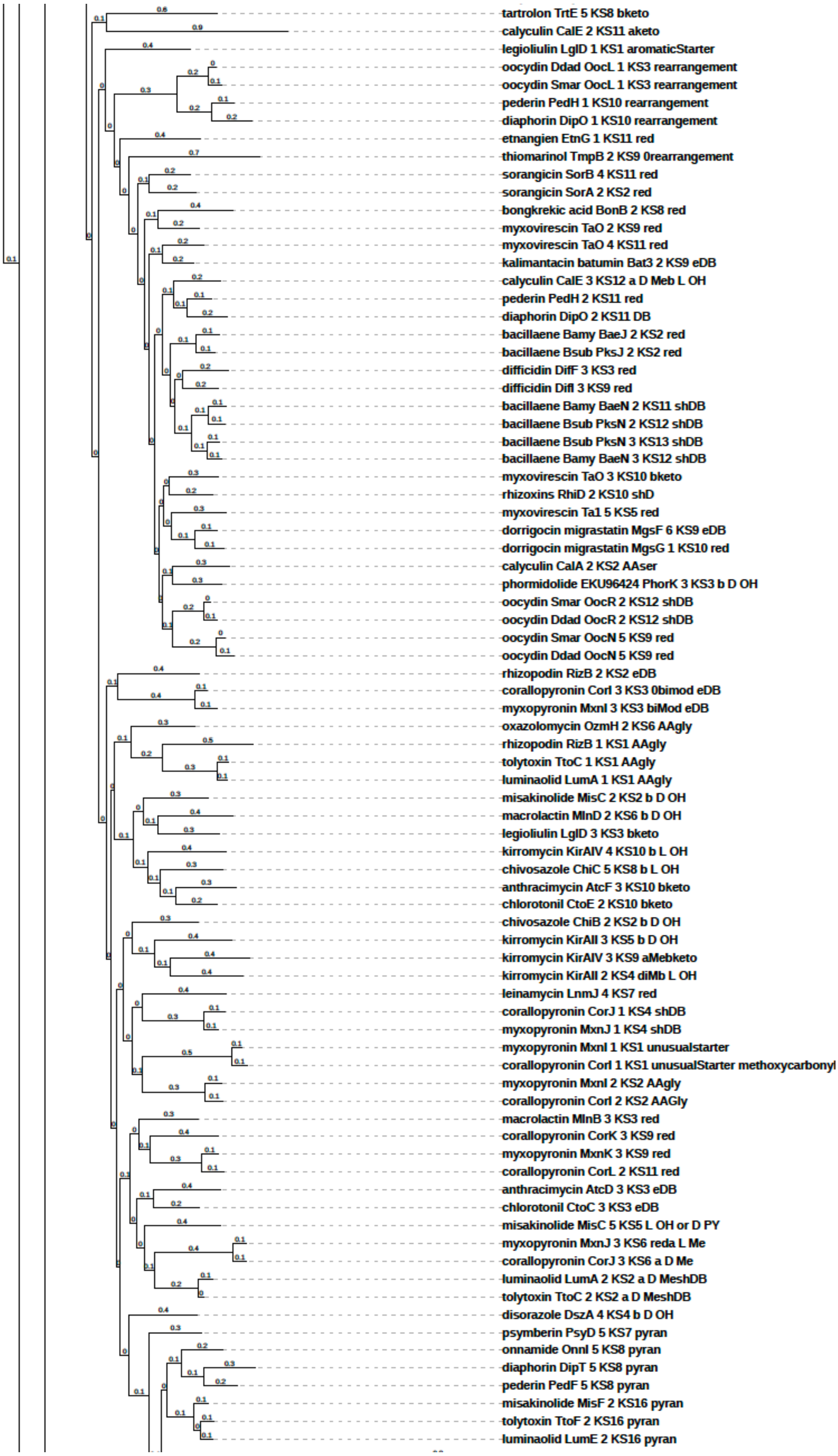
tartrolon TrtE 5 KS8 bketo
calyculin CalE 2 KS11 aketo
legioliulin LglD 1 KS1 aromaticStarter
oocydin Ddad OocL 1 KS3 rearrangement
oocydin Smar OocL 1 KS3 rearrangement
pederin PedH 1 KS10 rearrangement
diaphorin DipO 1 KS10 rearrangement
etnangien EtnG 1 KS11 red
thiomarinol TmpB 2 KS9 0rearrangement
sorangicin SorB 4 KS11 red
sorangicin SorA 2 KS2 red
bongkrekic acid BonB 2 KS8 red
myxovirescin TaO 2 KS9 red
myxovirescin TaO 4 KS11 red
kalimantacin batumin Bat3 2 KS9 eDB
calyculin CalE 3 KS12 a D Meb L OH
pederin PedH 2 KS11 red
diaphorin DipO 2 KS11 DB
bacillaene Bamy BaeJ 2 KS2 red
bacillaene Bsub PksJ 2 KS2 red
difficidin DifF 3 KS3 red
difficidin DifI 3 KS9 red
bacillaene Bamy BaeN 2 KS11 shDB
bacillaene Bsub PksN 2 KS12 shDB
bacillaene Bsub PksN 3 KS13 shDB
bacillaene Bamy BaeN 3 KS12 shDB
myxovirescin TaO 3 KS10 bketo
rhizoxins RhiD 2 KS10 shD
myxovirescin Ta1 5 KS5 red
dorrigocin migrastatin MgsF 6 KS9 eDB
dorrigocin migrastatin MgsG 1 KS10 red
calyculin CalA 2 KS2 AAser
phormidolide EKU96424 PhorK 3 KS3 b D OH
oocydin Smar OocR 2 KS12 shDB
oocydin Ddad OocR 2 KS12 shDB
oocydin Smar OocN 5 KS9 red
oocydin Ddad OocN 5 KS9 red
rhizopodin RizB 2 KS2 eDB
corallopyronin CorI 3 KS3 0bimod eDB
myxopyronin MxnI 3 KS3 biMod eDB
oxazolomycin OzmH 2 KS6 AAgly
rhizopodin RizB 1 KS1 AAgly
tolytoxin TtoC 1 KS1 AAgly
luminaolid LumA 1 KS1 AAgly
misakinolide MisC 2 KS2 b D OH
macrolactin MlnD 2 KS6 b D OH
legioliulin LglD 3 KS3 bketo
kirromycin KirAIV 4 KS10 b L OH
chivosazole ChiC 5 KS8 b L OH
anthracimycin AtcF 3 KS10 bketo
chlorotonil CtoE 2 KS10 bketo
chivosazole ChiB 2 KS2 b D OH
kirromycin KirAII 3 KS5 b D OH
kirromycin KirAIV 3 KS9 aMebketo
kirromycin KirAII 2 KS4 diMb L OH
leinamycin LnmJ 4 KS7 red
corallopyronin CorJ 1 KS4 shDB
myxopyronin MxnJ 1 KS4 shDB
myxopyronin MxnI 1 KS1 unusualstarter
corallopyronin CorI 1 KS1 unusualStarter methoxycarbonyl
myxopyronin MxnI 2 KS2 AAgly
corallopyronin CorI 2 KS2 AAGly
macrolactin MlnB 3 KS3 red
corallopyronin CorK 3 KS9 red
myxopyronin MxnK 3 KS9 red
corallopyronin CorL 2 KS11 red
anthracimycin AtcD 3 KS3 eDB
chlorotonil CtoC 3 KS3 eDB
misakinolide MisC 5 KS5 L OH or D PY
myxopyronin MxnJ 3 KS6 reda L Me
corallopyronin CorJ 3 KS6 a D Me
luminaolid LumA 2 KS2 a D MeshDB
tolytoxin TtoC 2 KS2 a D MeshDB
disorazole DszA 4 KS4 b D OH
psymberin PsyD 5 KS7 pyran
onnamide OnnI 5 KS8 pyran
diaphorin DipT 5 KS8 pyran
pederin PedF 5 KS8 pyran
misakinolide MisF 2 KS16 pyran
tolytoxin TtoF 2 KS16 pyran
luminaolid LumE 2 KS16 pyran

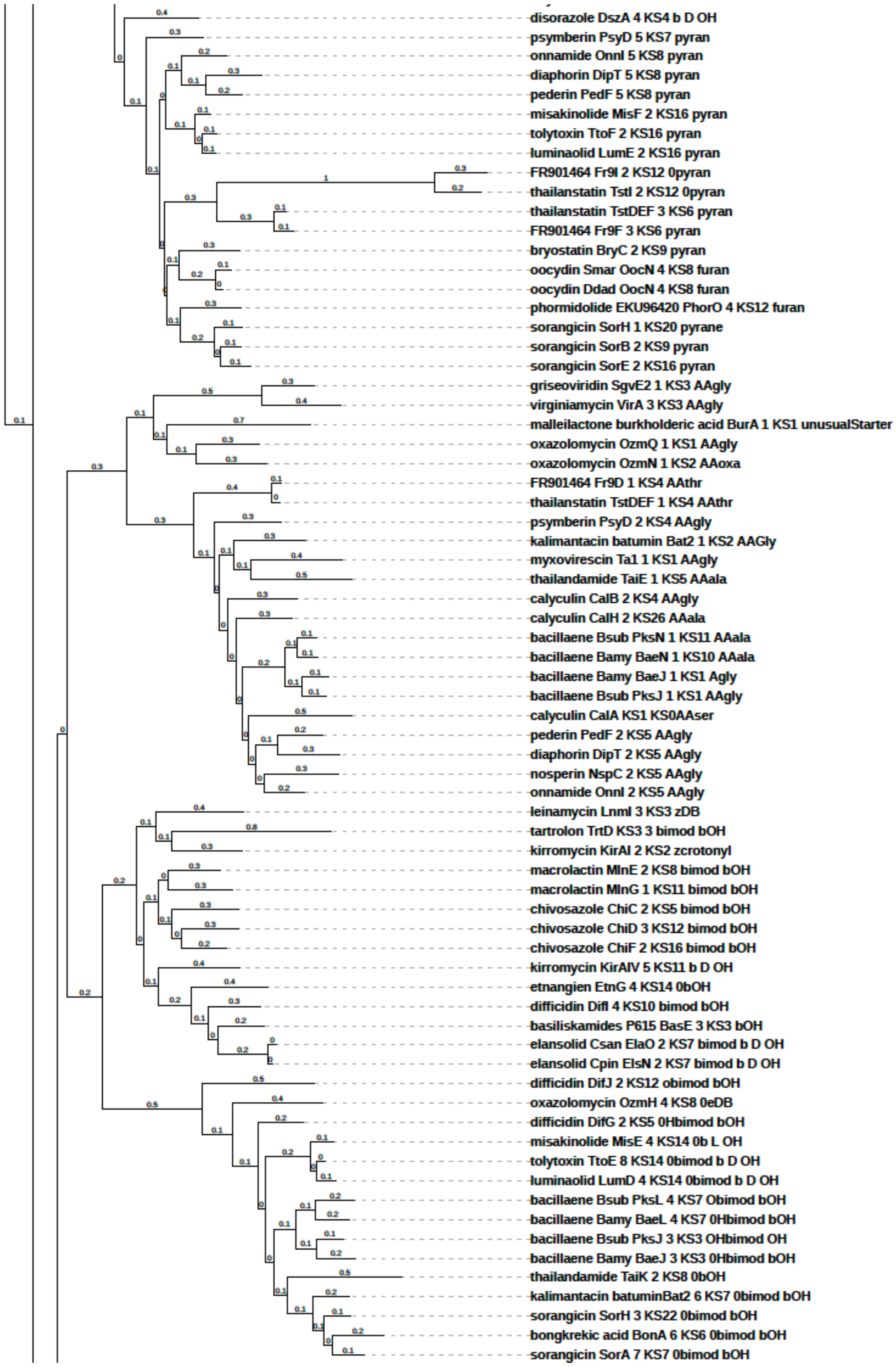
disorazole DszA 4 KS4 b D OH
psymberin PsyD 5 KS7 pyran
onnamide OnnI 5 KS8 pyran
diaphorin DipT 5 KS8 pyran
pederin PedF 5 KS8 pyran
misakinolide MisF 2 KS16 pyran
tolytoxin TtoF 2 KS16 pyran
luminaolid LumE 2 KS16 pyran
FR901464 Fr9I 2 KS12 0pyran
thailanstatin TstI 2 KS12 0pyran
thailanstatin TstDEF 3 KS6 pyran
FR901464 Fr9F 3 KS6 pyran
bryostatin BryC 2 KS9 pyran
oocydin Smar OocN 4 KS8 furan
oocydin Ddad OocN 4 KS8 furan
phormidolide EKU96420 PhorO 4 KS12 furan
sorangicin SorH 1 KS20 pyrane
sorangicin SorB 2 KS9 pyran
sorangicin SorE 2 KS16 pyran
griseoviridin SgvE2 1 KS3 AAgly
virginiamycin VirA 3 KS3 AAgly
malleilactone burkholderic acid BurA 1 KS1 unusualStarter
oxazolomycin OzmQ 1 KS1 AAgly
oxazolomycin OzmN 1 KS2 AAoxa
FR901464 Fr9D 1 KS4 AAthr
thailanstatin TstDEF 1 KS4 AAthr
psymberin PsyD 2 KS4 AAgly
kalimantacin batumin Bat2 1 KS2 AAGly
myxovirescin Ta1 1 KS1 AAgly
thailandamide TaiE 1 KS5 AAala
calyculin CalB 2 KS4 AAgly
calyculin CalH 2 KS26 AAala
bacillaene Bsub PksN 1 KS11 AAala
bacillaene Bamy BaeN 1 KS10 AAala
bacillaene Bamy BaeJ 1 KS1 Agly
bacillaene Bsub PksJ 1 KS1 AAgly
calyculin CalA KS1 KS0AAser
pederin PedF 2 KS5 AAgly
diaphorin DipT 2 KS5 AAgly
nosperin NspC 2 KS5 AAgly
onnamide OnnI 2 KS5 AAgly
leinamycin LnmI 3 KS3 zDB
tartrolon TrtD KS3 3 bimod bOH
kirromycin KirAI 2 KS2 zcrotonyl
macrolactin MlnE 2 KS8 bimod bOH
macrolactin MlnG 1 KS11 bimod bOH
chivosazole ChiC 2 KS5 bimod bOH
chivosazole ChiD 3 KS12 bimod bOH
chivosazole ChiF 2 KS16 bimod bOH
kirromycin KirAIV 5 KS11 b D OH
etnangien EtnG 4 KS14 0bOH
difficidin DifI 4 KS10 bimod bOH
basiliskamides P615 BasE 3 KS3 bOH
elansolid Csan ElaO 2 KS7 bimod b D OH
elansolid Cpin ElsN 2 KS7 bimod b D OH
difficidin DifJ 2 KS12 obimod bOH
oxazolomycin OzmH 4 KS8 0eDB
difficidin DifG 2 KS5 0Hbimod bOH
misakinolide MisE 4 KS14 0b L OH
tolytoxin TtoE 8 KS14 0bimod b D OH
luminaolid LumD 4 KS14 0bimod b D OH
bacillaene Bsub PksL 4 KS7 Obimod bOH
bacillaene Bamy BaeL 4 KS7 0Hbimod bOH
bacillaene Bsub PksJ 3 KS3 OHbimod OH
bacillaene Bamy BaeJ 3 KS3 0Hbimod bOH
thailandamide TaiK 2 KS8 0bOH
kalimantacin batuminBat2 6 KS7 0bimod bOH
sorangicin SorH 3 KS22 0bimod bOH
bongkrekic acid BonA 6 KS6 0bimod bOH
sorangicin SorA 7 KS7 0bimod bOH

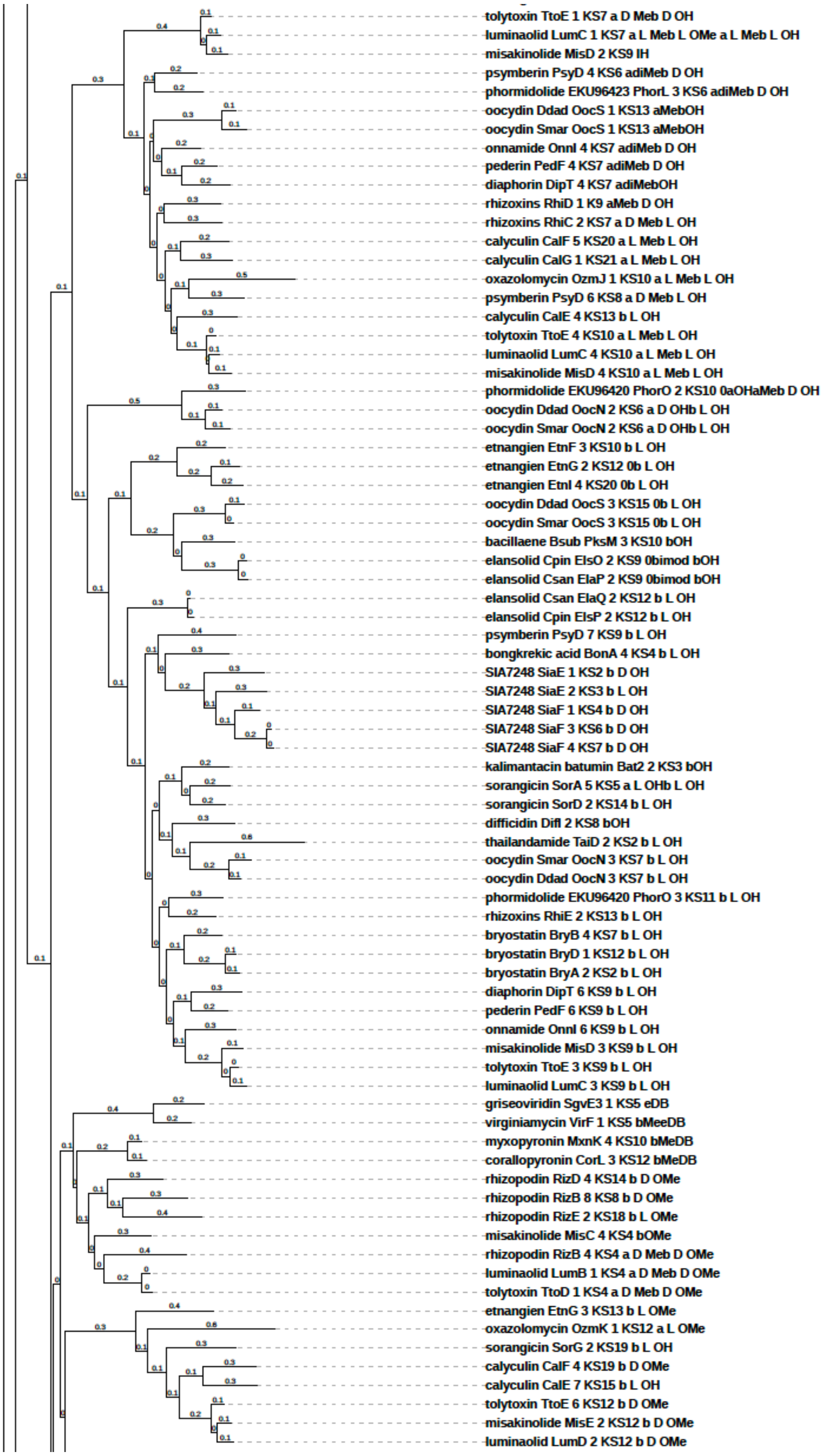
tolytoxin TtoE 1 KS7 a D Meb D OH
luminaolid LumC 1 KS7 a L Meb L OMe a L Meb L OH
misakinolide MisD 2 KS9 IH
psymberin PsyD 4 KS6 adiMeb D OH
phormidolide EKU96423 PhorL 3 KS6 adiMeb D OH
oocydin Ddad OocS 1 KS13 aMebOH
oocydin Smar OocS 1 KS13 aMebOH
onnamide OnnI 4 KS7 adiMeb D OH
pederin PedF 4 KS7 adiMeb D OH
diaphorin DipT 4 KS7 adiMebOH
rhizoxins RhiD 1 K9 aMeb D OH
rhizoxins RhiC 2 KS7 a D Meb L OH
calyculin CalF 5 KS20 a L Meb L OH
calyculin CalG 1 KS21 a L Meb L OH
oxazolomycin OzmJ 1 KS10 a L Meb L OH
psymberin PsyD 6 KS8 a D Meb L OH
calyculin CalE 4 KS13 b L OH
tolytoxin TtoE 4 KS10 a L Meb L OH
luminaolid LumC 4 KS10 a L Meb L OH
misakinolide MisD 4 KS10 a L Meb L OH
phormidolide EKU96420 PhorO 2 KS10 0aOHaMeb D OH
oocydin Ddad OocN 2 KS6 a D OHb L OH
oocydin Smar OocN 2 KS6 a D OHb L OH
etnangien EtnF 3 KS10 b L OH
etnangien EtnG 2 KS12 0b L OH
etnangien EtnI 4 KS20 0b L OH
oocydin Ddad OocS 3 KS15 0b L OH
oocydin Smar OocS 3 KS15 0b L OH
bacillaene Bsub PksM 3 KS10 bOH
elansolid Cpin ElsO 2 KS9 0bimod bOH
elansolid Csan ElaP 2 KS9 0bimod bOH
elansolid Csan ElaQ 2 KS12 b L OH
elansolid Cpin ElsP 2 KS12 b L OH
psymberin PsyD 7 KS9 b L OH
bongkrekic acid BonA 4 KS4 b L OH
SIA7248 SiaE 1 KS2 b D OH
SIA7248 SiaE 2 KS3 b L OH
SIA7248 SiaF 1 KS4 b D OH
SIA7248 SiaF 3 KS6 b D OH
SIA7248 SiaF 4 KS7 b D OH
kalimantacin batumin Bat2 2 KS3 bOH
sorangicin SorA 5 KS5 a L OHb L OH
sorangicin SorD 2 KS14 b L OH
difficidin DifI 2 KS8 bOH
thailandamide TaiD 2 KS2 b L OH
oocydin Smar OocN 3 KS7 b L OH
oocydin Ddad OocN 3 KS7 b L OH
phormidolide EKU96420 PhorO 3 KS11 b L OH
rhizoxins RhiE 2 KS13 b L OH
bryostatin BryB 4 KS7 b L OH
bryostatin BryD 1 KS12 b L OH
bryostatin BryA 2 KS2 b L OH
diaphorin DipT 6 KS9 b L OH
pederin PedF 6 KS9 b L OH
onnamide OnnI 6 KS9 b L OH
misakinolide MisD 3 KS9 b L OH
tolytoxin TtoE 3 KS9 b L OH
luminaolid LumC 3 KS9 b L OH
griseoviridin SgvE3 1 KS5 eDB
virginiamycin VirF 1 KS5 bMeeDB
myxopyronin MxnK 4 KS10 bMeDB
corallopyronin CorL 3 KS12 bMeDB
rhizopodin RizD 4 KS14 b D OMe
rhizopodin RizB 8 KS8 b D OMe
rhizopodin RizE 2 KS18 b L OMe
misakinolide MisC 4 KS4 bOMe
rhizopodin RizB 4 KS4 a D Meb D OMe
luminaolid LumB 1 KS4 a D Meb D OMe
tolytoxin TtoD 1 KS4 a D Meb D OMe
etnangien EtnG 3 KS13 b L OMe
oxazolomycin OzmK 1 KS12 a L OMe
sorangicin SorG 2 KS19 b L OH
calyculin CalF 4 KS19 b D OMe
calyculin CalE 7 KS15 b L OH
tolytoxin TtoE 6 KS12 b D OMe
misakinolide MisE 2 KS12 b D OMe
luminaolid LumD 2 KS12 b D OMe

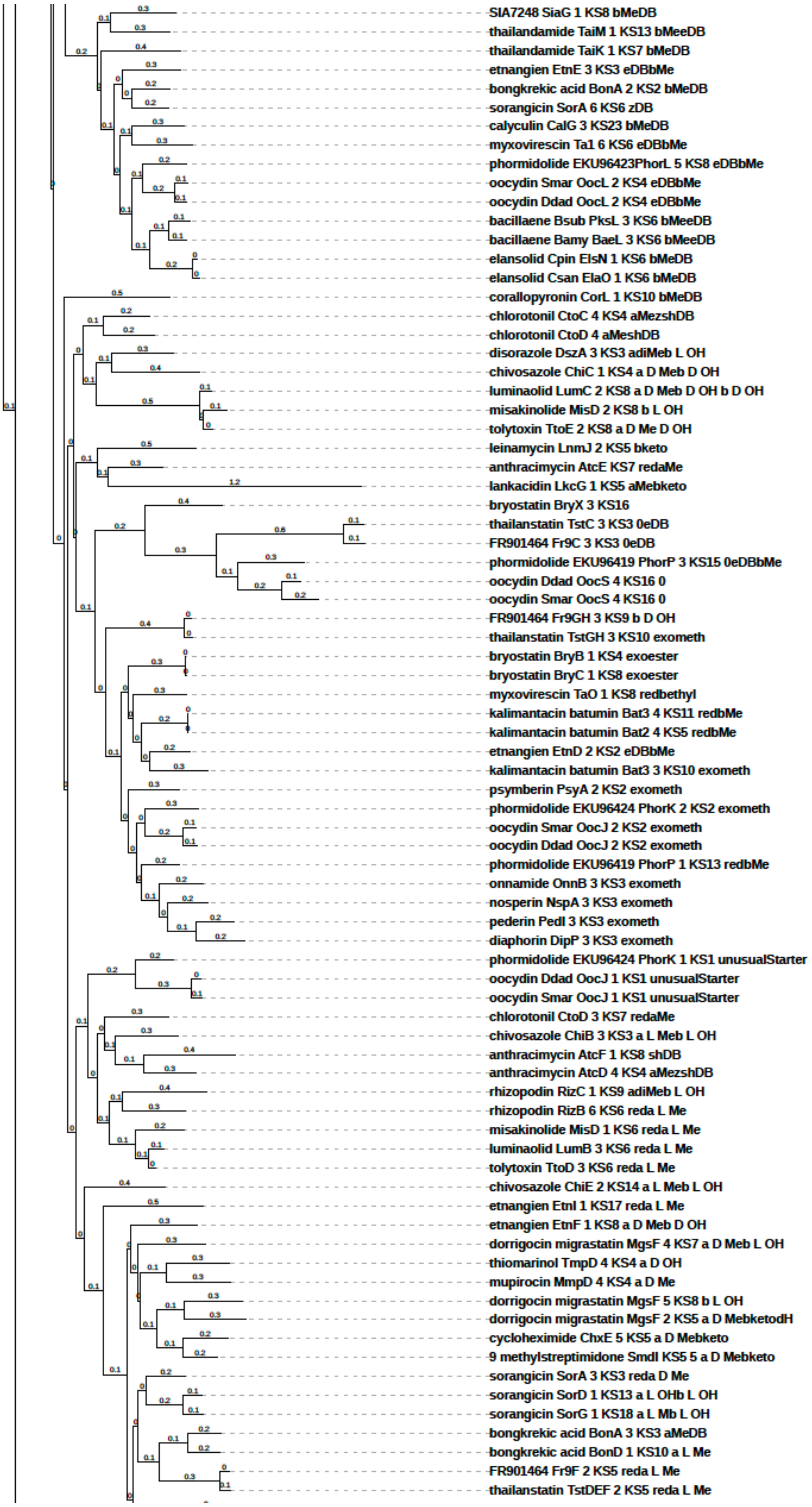

SIA7248 SiaG 1 KS8 bMeDB
thailandamide TaiM 1 KS13 bMeeDB
thailandamide TaiK 1 KS7 bMeDB
etnangien EtnE 3 KS3 eDBbMe
bongkrekic acid BonA 2 KS2 bMeDB
sorangicin SorA 6 KS6 zDB
calyculin CalG 3 KS23 bMeDB
myxovirescin Ta1 6 KS6 eDBbMe
phormidolide EKU96423PhorL 5 KS8 eDBbMe
oocydin Smar OocL 2 KS4 eDBbMe
oocydin Ddad OocL 2 KS4 eDBbMe
bacillaene Bsub PksL 3 KS6 bMeeDB
bacillaene Bamy BaeL 3 KS6 bMeeDB
elansolid Cpin ElsN 1 KS6 bMeDB
elansolid Csan ElaO 1 KS6 bMeDB
corallopyronin CorL 1 KS10 bMeDB
chlorotonil CtoC 4 KS4 aMezshDB
chlorotonil CtoD 4 aMeshDB
disorazole DszA 3 KS3 adiMeb L OH
chivosazole ChiC 1 KS4 a D Meb D OH
luminaolid LumC 2 KS8 a D Meb D OH b D OH
misakinolide MisD 2 KS8 b L OH
tolytoxin TtoE 2 KS8 a D Me D OH
leinamycin LnmJ 2 KS5 bketo
anthracimycin AtcE KS7 redaMe
lankacidin LkcG 1 KS5 aMebketo
bryostatin BryX 3 KS16
thailanstatin TstC 3 KS3 0eDB
FR901464 Fr9C 3 KS3 0eDB
phormidolide EKU96419 PhorP 3 KS15 0eDBbMe
oocydin Ddad OocS 4 KS16 0
oocydin Smar OocS 4 KS16 0
FR901464 Fr9GH 3 KS9 b D OH
thailanstatin TstGH 3 KS10 exometh
bryostatin BryB 1 KS4 exoester
bryostatin BryC 1 KS8 exoester
myxovirescin TaO 1 KS8 redbethyl
kalimantacin batumin Bat3 4 KS11 redbMe
kalimantacin batumin Bat2 4 KS5 redbMe
etnangien EtnD 2 KS2 eDBbMe
kalimantacin batumin Bat3 3 KS10 exometh
psymberin PsyA 2 KS2 exometh
phormidolide EKU96424 PhorK 2 KS2 exometh
oocydin Smar OocJ 2 KS2 exometh
oocydin Ddad OocJ 2 KS2 exometh
phormidolide EKU96419 PhorP 1 KS13 redbMe
onnamide OnnB 3 KS3 exometh
nosperin NspA 3 KS3 exometh
pederin PedI 3 KS3 exometh
diaphorin DipP 3 KS3 exometh
phormidolide EKU96424 PhorK 1 KS1 unusualStarter
oocydin Ddad OocJ 1 KS1 unusualStarter
oocydin Smar OocJ 1 KS1 unusualStarter
chlorotonil CtoD 3 KS7 redaMe
chivosazole ChiB 3 KS3 a L Meb L OH
anthracimycin AtcF 1 KS8 shDB
anthracimycin AtcD 4 KS4 aMezshDB
rhizopodin RizC 1 KS9 adiMeb L OH
rhizopodin RizB 6 KS6 reda L Me
misakinolide MisD 1 KS6 reda L Me
luminaolid LumB 3 KS6 reda L Me
tolytoxin TtoD 3 KS6 reda L Me
chivosazole ChiE 2 KS14 a L Meb L OH
etnangien EtnI 1 KS17 reda L Me
etnangien EtnF 1 KS8 a D Meb D OH
dorrigocin migrastatin MgsF 4 KS7 a D Meb L OH
thiomarinol TmpD 4 KS4 a D OH
mupirocin MmpD 4 KS4 a D Me
dorrigocin migrastatin MgsF 5 KS8 b L OH
dorrigocin migrastatin MgsF 2 KS5 a D MebketodH
cycloheximide ChxE 5 KS5 a D Mebketo
9 methylstreptimidone SmdI KS5 5 a D Mebketo
sorangicin SorA 3 KS3 reda D Me
sorangicin SorD 1 KS13 a L OHb L OH
sorangicin SorG 1 KS18 a L Mb L OH
bongkrekic acid BonA 3 KS3 aMeDB
bongkrekic acid BonD 1 KS10 a L Me
FR901464 Fr9F 2 KS5 reda L Me
thailanstatin TstDEF 2 KS5 reda L Me

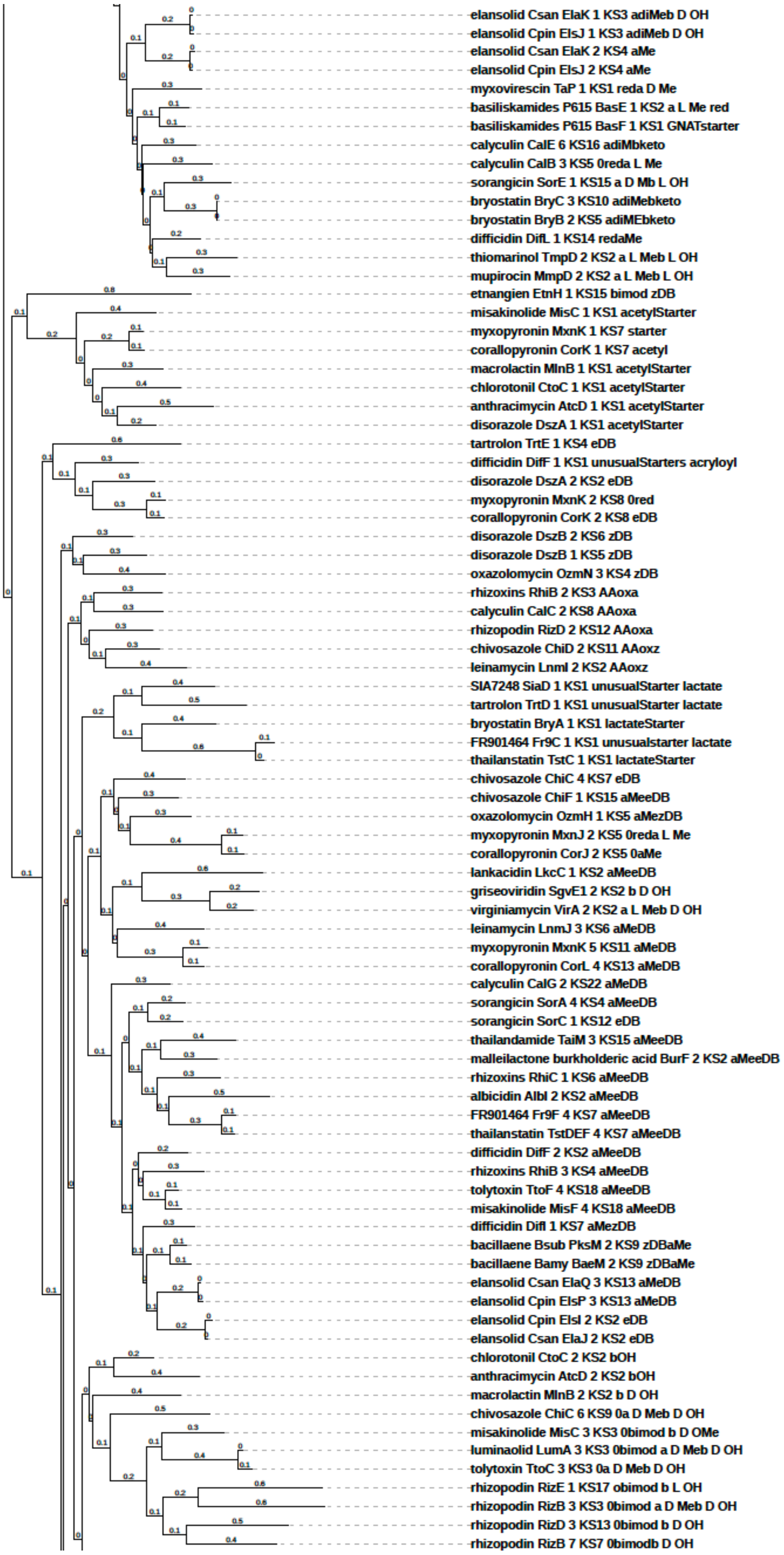
elansolid Csan ElaK 1 KS3 adiMeb D OH
elansolid Cpin ElsJ 1 KS3 adiMeb D OH
elansolid Csan ElaK 2 KS4 aMe
elansolid Cpin ElsJ 2 KS4 aMe
myxovirescin TaP 1 KS1 reda D Me
basiliskamides P615 BasE 1 KS2 a L Me red
basiliskamides P615 BasF 1 KS1 GNATstarter
calyculin CalE 6 KS16 adiMbketo
calyculin CalB 3 KS5 0reda L Me
sorangicin SorE 1 KS15 a D Mb L OH
bryostatin BryC 3 KS10 adiMebketo
bryostatin BryB 2 KS5 adiMEbketo
difficidin DifL 1 KS14 redaMe
thiomarinol TmpD 2 KS2 a L Meb L OH
mupirocin MmpD 2 KS2 a L Meb L OH
etnangien EtnH 1 KS15 bimod zDB
misakinolide MisC 1 KS1 acetylStarter
myxopyronin MxnK 1 KS7 starter
corallopyronin CorK 1 KS7 acetyl
macrolactin MlnB 1 KS1 acetylStarter
chlorotonil CtoC 1 KS1 acetylStarter
anthracimycin AtcD 1 KS1 acetylStarter
disorazole DszA 1 KS1 acetylStarter
tartrolon TrtE 1 KS4 eDB
difficidin DifF 1 KS1 unusualStarters acryloyl
disorazole DszA 2 KS2 eDB
myxopyronin MxnK 2 KS8 0red
corallopyronin CorK 2 KS8 eDB
disorazole DszB 2 KS6 zDB
disorazole DszB 1 KS5 zDB
oxazolomycin OzmN 3 KS4 zDB
rhizoxins RhiB 2 KS3 AAoxa
calyculin CalC 2 KS8 AAoxa
rhizopodin RizD 2 KS12 AAoxa
chivosazole ChiD 2 KS11 AAoxz
leinamycin LnmI 2 KS2 AAoxz
SIA7248 SiaD 1 KS1 unusualStarter lactate
tartrolon TrtD 1 KS1 unusualStarter lactate
bryostatin BryA 1 KS1 lactateStarter
FR901464 Fr9C 1 KS1 unusualstarter lactate
thailanstatin TstC 1 KS1 lactateStarter
chivosazole ChiC 4 KS7 eDB
chivosazole ChiF 1 KS15 aMeeDB
oxazolomycin OzmH 1 KS5 aMezDB
myxopyronin MxnJ 2 KS5 0reda L Me
corallopyronin CorJ 2 KS5 0aMe
lankacidin LkcC 1 KS2 aMeeDB
griseoviridin SgvE1 2 KS2 b D OH
virginiamycin VirA 2 KS2 a L Meb D OH
leinamycin LnmJ 3 KS6 aMeDB
myxopyronin MxnK 5 KS11 aMeDB
corallopyronin CorL 4 KS13 aMeDB
calyculin CalG 2 KS22 aMeDB
sorangicin SorA 4 KS4 aMeeDB
sorangicin SorC 1 KS12 eDB
thailandamide TaiM 3 KS15 aMeeDB
malleilactone burkholderic acid BurF 2 KS2 aMeeDB
rhizoxins RhiC 1 KS6 aMeeDB
albicidin AlbI 2 KS2 aMeeDB
FR901464 Fr9F 4 KS7 aMeeDB
thailanstatin TstDEF 4 KS7 aMeeDB
difficidin DifF 2 KS2 aMeeDB
rhizoxins RhiB 3 KS4 aMeeDB
tolytoxin TtoF 4 KS18 aMeeDB
misakinolide MisF 4 KS18 aMeeDB
difficidin DifI 1 KS7 aMezDB
bacillaene Bsub PksM 2 KS9 zDBaMe
bacillaene Bamy BaeM 2 KS9 zDBaMe
elansolid Csan ElaQ 3 KS13 aMeDB
elansolid Cpin ElsP 3 KS13 aMeDB
elansolid Cpin ElsI 2 KS2 eDB
elansolid Csan ElaJ 2 KS2 eDB
chlorotonil CtoC 2 KS2 bOH
anthracimycin AtcD 2 KS2 bOH
macrolactin MlnB 2 KS2 b D OH
chivosazole ChiC 6 KS9 0a D Meb D OH
misakinolide MisC 3 KS3 0bimod b D OMe
luminaolid LumA 3 KS3 0bimod a D Meb D OH
tolytoxin TtoC 3 KS3 0a D Meb D OH
rhizopodin RizE 1 KS17 obimod b L OH
rhizopodin RizB 3 KS3 0bimod a D Meb D OH
rhizopodin RizD 3 KS13 0bimod b D OH
rhizopodin RizB 7 KS7 0bimodb D OH

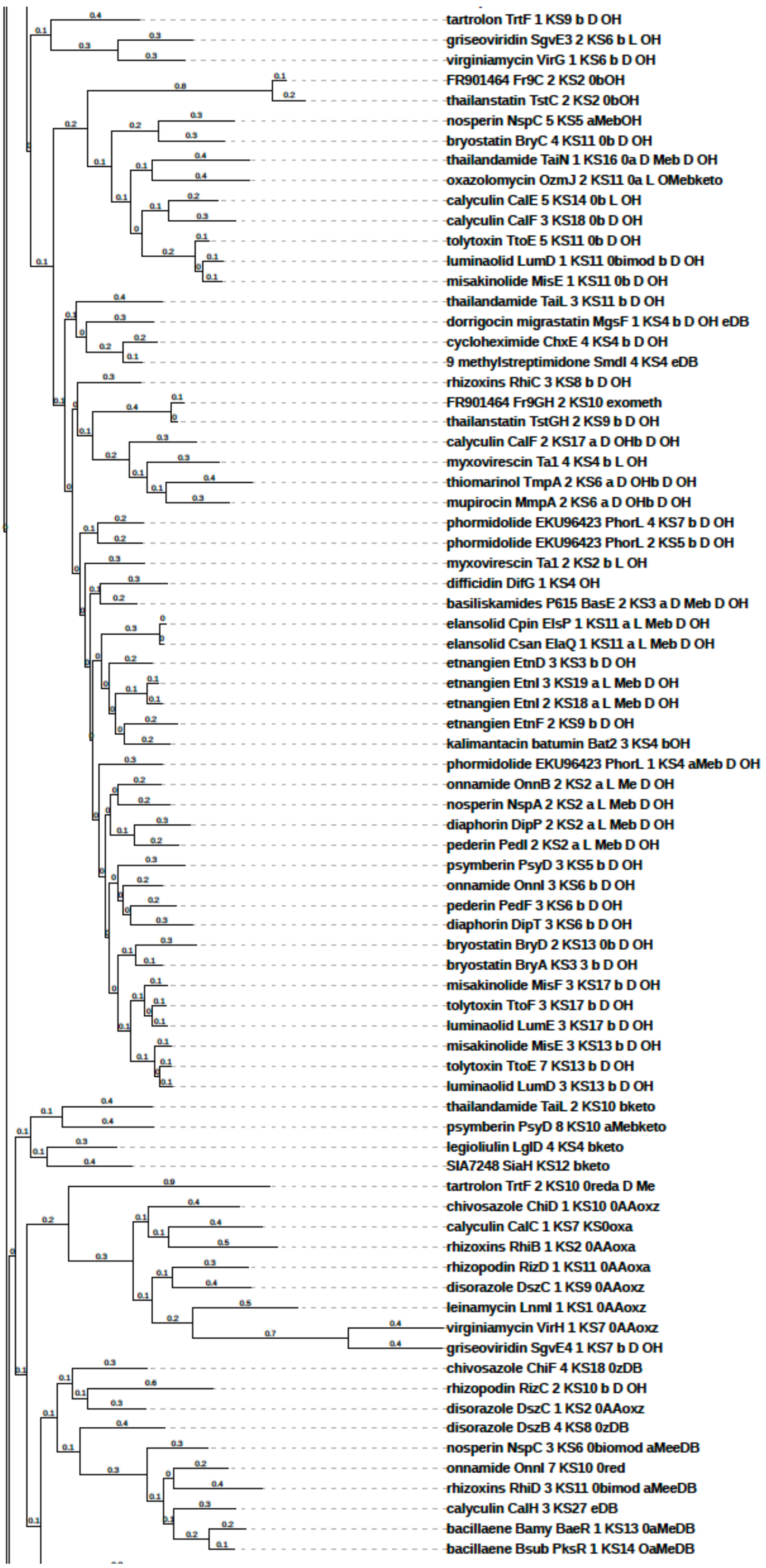

tartrolon TrtF 1 KS9 b D OH
griseoviridin SgvE3 2 KS6 b L OH
virginiamycin VirG 1 KS6 b D OH
FR901464 Fr9C 2 KS2 0bOH
thailanstatin TstC 2 KS2 0bOH
nosperin NspC 5 KS5 aMebOH
bryostatin BryC 4 KS11 0b D OH
thailandamide TaiN 1 KS16 0a D Meb D OH
oxazolomycin OzmJ 2 KS11 0a L OMebketo
calyculin CalE 5 KS14 0b L OH
calyculin CalF 3 KS18 0b D OH
tolytoxin TtoE 5 KS11 0b D OH
luminaolid LumD 1 KS11 0bimod b D OH
misakinolide MisE 1 KS11 0b D OH
thailandamide TaiL 3 KS11 b D OH
dorrigocin migrastatin MgsF 1 KS4 b D OH eDB
cycloheximide ChxE 4 KS4 b D OH
9 methylstreptimidone SmdI 4 KS4 eDB
rhizoxins RhiC 3 KS8 b D OH
FR901464 Fr9GH 2 KS10 exometh
thailanstatin TstGH 2 KS9 b D OH
calyculin CalF 2 KS17 a D OHb D OH
myxovirescin Ta1 4 KS4 b L OH
thiomarinol TmpA 2 KS6 a D OHb D OH
mupirocin MmpA 2 KS6 a D OHb D OH
phormidolide EKU96423 PhorL 4 KS7 b D OH
phormidolide EKU96423 PhorL 2 KS5 b D OH
myxovirescin Ta1 2 KS2 b L OH
difficidin DifG 1 KS4 OH
basiliskamides P615 BasE 2 KS3 a D Meb D OH
elansolid Cpin ElsP 1 KS11 a L Meb D OH
elansolid Csan ElaQ 1 KS11 a L Meb D OH
etnangien EtnD 3 KS3 b D OH
etnangien EtnI 3 KS19 a L Meb D OH
etnangien EtnI 2 KS18 a L Meb D OH
etnangien EtnF 2 KS9 b D OH
kalimantacin batumin Bat2 3 KS4 bOH
phormidolide EKU96423 PhorL 1 KS4 aMeb D OH
onnamide OnnB 2 KS2 a L Me D OH
nosperin NspA 2 KS2 a L Meb D OH
diaphorin DipP 2 KS2 a L Meb D OH
pederin PedI 2 KS2 a L Meb D OH
psymberin PsyD 3 KS5 b D OH
onnamide OnnI 3 KS6 b D OH
pederin PedF 3 KS6 b D OH
diaphorin DipT 3 KS6 b D OH
bryostatin BryD 2 KS13 0b D OH
bryostatin BryA KS3 3 b D OH
misakinolide MisF 3 KS17 b D OH
tolytoxin TtoF 3 KS17 b D OH
luminaolid LumE 3 KS17 b D OH
misakinolide MisE 3 KS13 b D OH
tolytoxin TtoE 7 KS13 b D OH
luminaolid LumD 3 KS13 b D OH
thailandamide TaiL 2 KS10 bketo
psymberin PsyD 8 KS10 aMebketo
legioliulin LgID 4 KS4 bketo
SIA7248 SiaH KS12 bketo
tartrolon TrtF 2 KS10 0reda D Me
chivosazole ChiD 1 KS10 0AAoxz
calyculin CalC 1 KS7 KS0oxa
rhizoxins RhiB 1 KS2 0AAoxa
rhizopodin RizD 1 KS11 0AAoxa
disorazole DszC 1 KS9 0AAoxz
leinamycin LnmI 1 KS1 0AAoxz
virginiamycin VirH 1 KS7 0AAoxz
griseoviridin SgvE4 1 KS7 b D OH
chivosazole ChiF 4 KS18 0zDB
rhizopodin RizC 2 KS10 b D OH
disorazole DszC 1 KS2 0AAoxz
disorazole DszB 4 KS8 0zDB
nosperin NspC 3 KS6 0biomod aMeeDB
onnamide OnnI 7 KS10 0red
rhizoxins RhiD 3 KS11 0bimod aMeeDB
calyculin CalH 3 KS27 eDB
bacillaene Bamy BaeR 1 KS13 0aMeDB
bacillaene Bsub PksR 1 KS14 OaMeDB

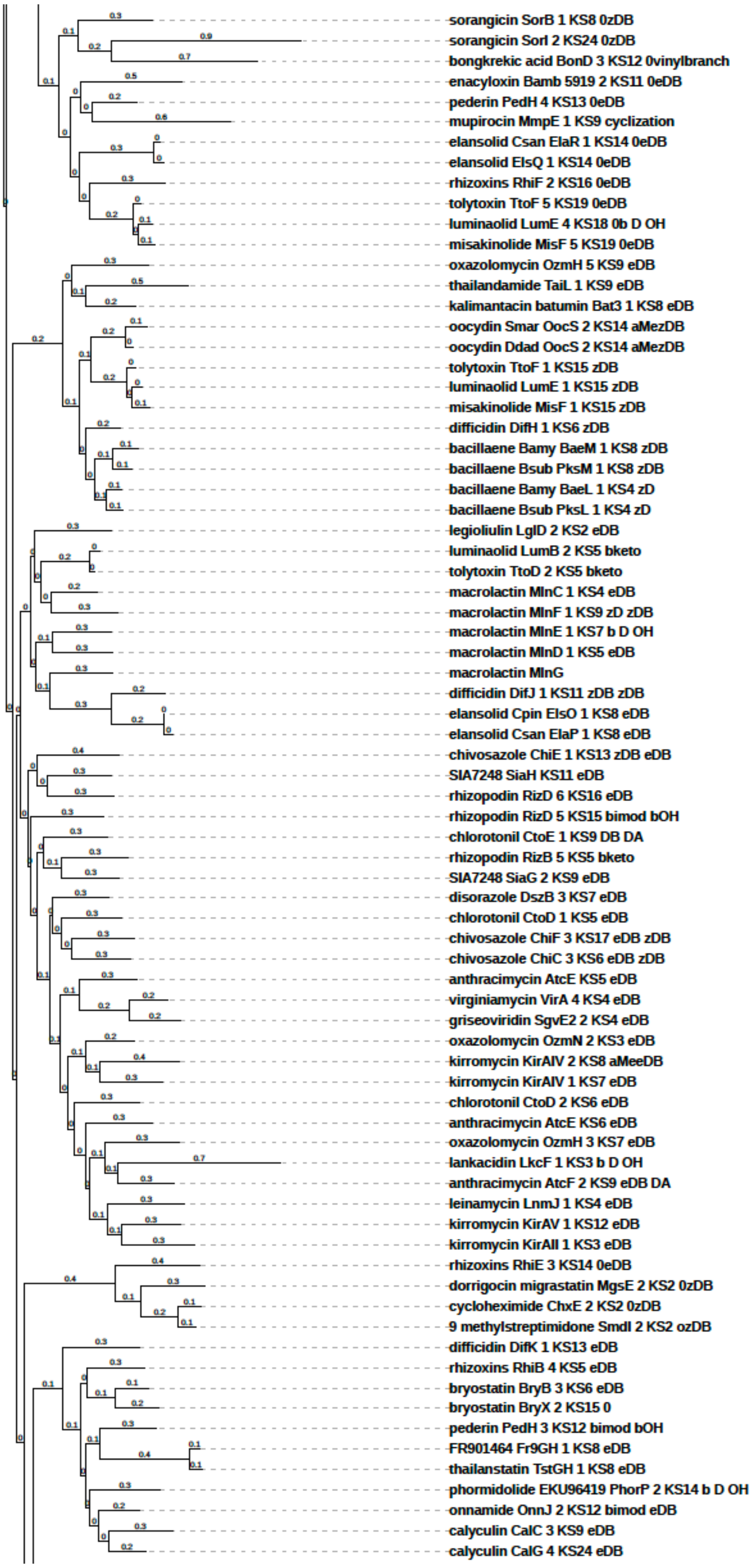
sorangicin SorB 1 KS8 0zDB
sorangicin SorI 2 KS24 0zDB
bongkrekic acid BonD 3 KS12 0vinylbranch
enacyloxin Bamb 5919 2 KS11 0eDB
pederin PedH 4 KS13 0eDB
mupirocin MmpE 1 KS9 cyclization
elansolid Csan ElaR 1 KS14 0eDB
elansolid ElsQ 1 KS14 0eDB
rhizoxins RhiF 2 KS16 0eDB
tolytoxin TtoF 5 KS19 0eDB
luminaolid LumE 4 KS18 0b D OH
misakinolide MisF 5 KS19 0eDB
oxazolomycin OzmH 5 KS9 eDB
thailandamide TaiL 1 KS9 eDB
kalimantacin batumin Bat3 1 KS8 eDB
oocydin Smar OocS 2 KS14 aMezDB
oocydin Ddad OocS 2 KS14 aMezDB
tolytoxin TtoF 1 KS15 zDB
luminaolid LumE 1 KS15 zDB
misakinolide MisF 1 KS15 zDB
difficidin DifH 1 KS6 zDB
bacillaene Bamy BaeM 1 KS8 zDB
bacillaene Bsub PksM 1 KS8 zDB
bacillaene Bamy BaeL 1 KS4 zD
bacillaene Bsub PksL 1 KS4 zD
legioliulin LglD 2 KS2 eDB
luminaolid LumB 2 KS5 bketo
tolytoxin TtoD 2 KS5 bketo
macrolactin MlnC 1 KS4 eDB
macrolactin MlnF 1 KS9 zD zDB
macrolactin MlnE 1 KS7 b D OH
macrolactin MlnD 1 KS5 eDB
macrolactin MlnG
difficidin DifJ 1 KS11 zDB zDB
elansolid Cpin ElsO 1 KS8 eDB
elansolid Csan ElaP 1 KS8 eDB
chivosazole ChiE 1 KS13 zDB eDB
SIA7248 SiaH KS11 eDB
rhizopodin RizD 6 KS16 eDB
rhizopodin RizD 5 KS15 bimod bOH
chlorotonil CtoE 1 KS9 DB DA
rhizopodin RizB 5 KS5 bketo
SIA7248 SiaG 2 KS9 eDB
disorazole DszB 3 KS7 eDB
chlorotonil CtoD 1 KS5 eDB
chivosazole ChiF 3 KS17 eDB zDB
chivosazole ChiC 3 KS6 eDB zDB
anthracimycin AtcE KS5 eDB
virginiamycin VirA 4 KS4 eDB
griseoviridin SgvE2 2 KS4 eDB
oxazolomycin OzmN 2 KS3 eDB
kirromycin KirAIV 2 KS8 aMeeDB
kirromycin KirAIV 1 KS7 eDB
chlorotonil CtoD 2 KS6 eDB
anthracimycin AtcE KS6 eDB
oxazolomycin OzmH 3 KS7 eDB
lankacidin LkcF 1 KS3 b D OH
anthracimycin AtcF 2 KS9 eDB DA
leinamycin LnmJ 1 KS4 eDB
kirromycin KirAV 1 KS12 eDB
kirromycin KirAII 1 KS3 eDB
rhizoxins RhiE 3 KS14 0eDB
dorrigocin migrastatin MgsE 2 KS2 0zDB
cycloheximide ChxE 2 KS2 0zDB
9 methylstreptimidone SmdI 2 KS2 ozDB
difficidin DifK 1 KS13 eDB
rhizoxins RhiB 4 KS5 eDB
bryostatin BryB 3 KS6 eDB
bryostatin BryX 2 KS15 0
pederin PedH 3 KS12 bimod bOH
FR901464 Fr9GH 1 KS8 eDB
thailanstatin TstGH 1 KS8 eDB
phormidolide EKU96419 PhorP 2 KS14 b D OH
onnamide OnnJ 2 KS12 bimod eDB
calyculin CalC 3 KS9 eDB
calyculin CalG 4 KS24 eDB

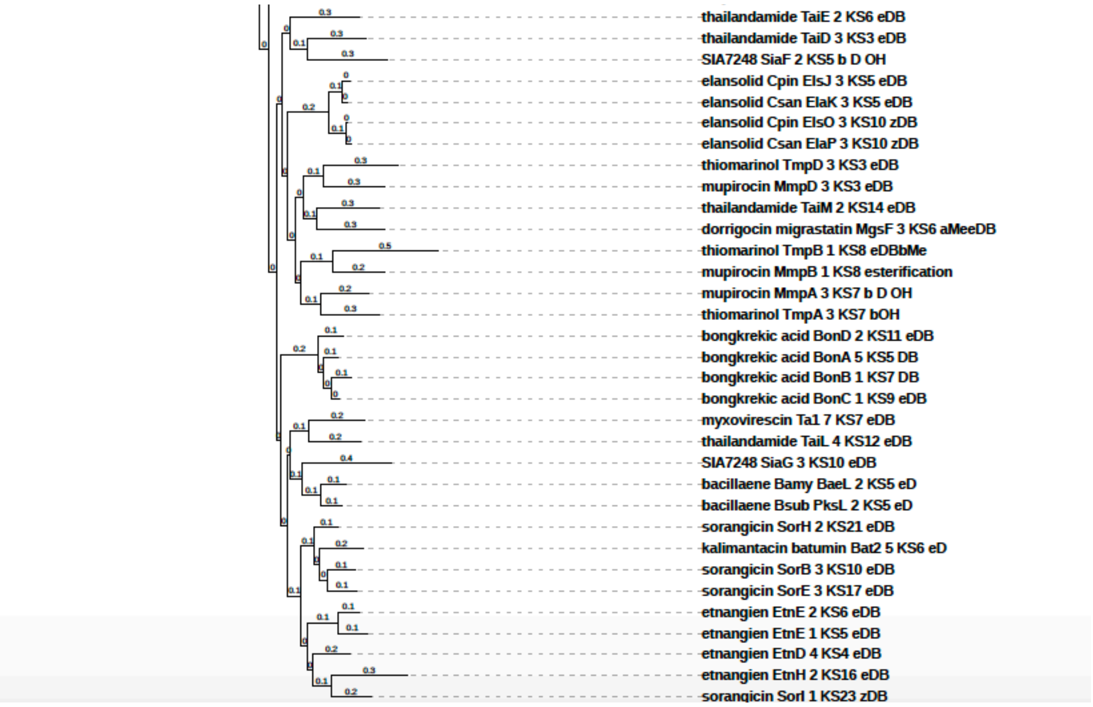

Supplementary Figure 1: **Random Axelerated Maximum likelihood (RAxML) phylogenetic tree calculated from a MUSCLE alignment of 647 KS sequences of all 49 characterized *trans*-AT PKS BGCs described in Helfrich and Piel.**[1] Legend: aMe: α-methyl, bOH: β-hydroxyl, aOH: α-hydroxyl, 0: non-elongating KS, hacetal: hemiacetal, DB: double bond, shDB: shifted double bond, red: completely reduced, vinylogous: vinylogous chain branching, Oxa/Oxz: oxazole, Thia: thiazole, eDB/eD: *E*-configured DB, zDB/zD: *Z*-configured DB, D_OH: D-configured hydroxyl group, L_OH: L-configured hydroxyl group, rearrangement: oxygen insertion, 2biMod: second KS in a bimodule, bimod: KS of a bimodule, 0bimod: non-elongating KS in a bimodule, ozDB: oxazole or double bond. Bimodule consist of two adjacent modules and jointly modify an intermediate. Branch nomenclature: polyketide name_protein name_number of KS on the respective protein_number of KS in the PKS_substrate specificity. Numbers on tree nodes indicate bootstrap values.

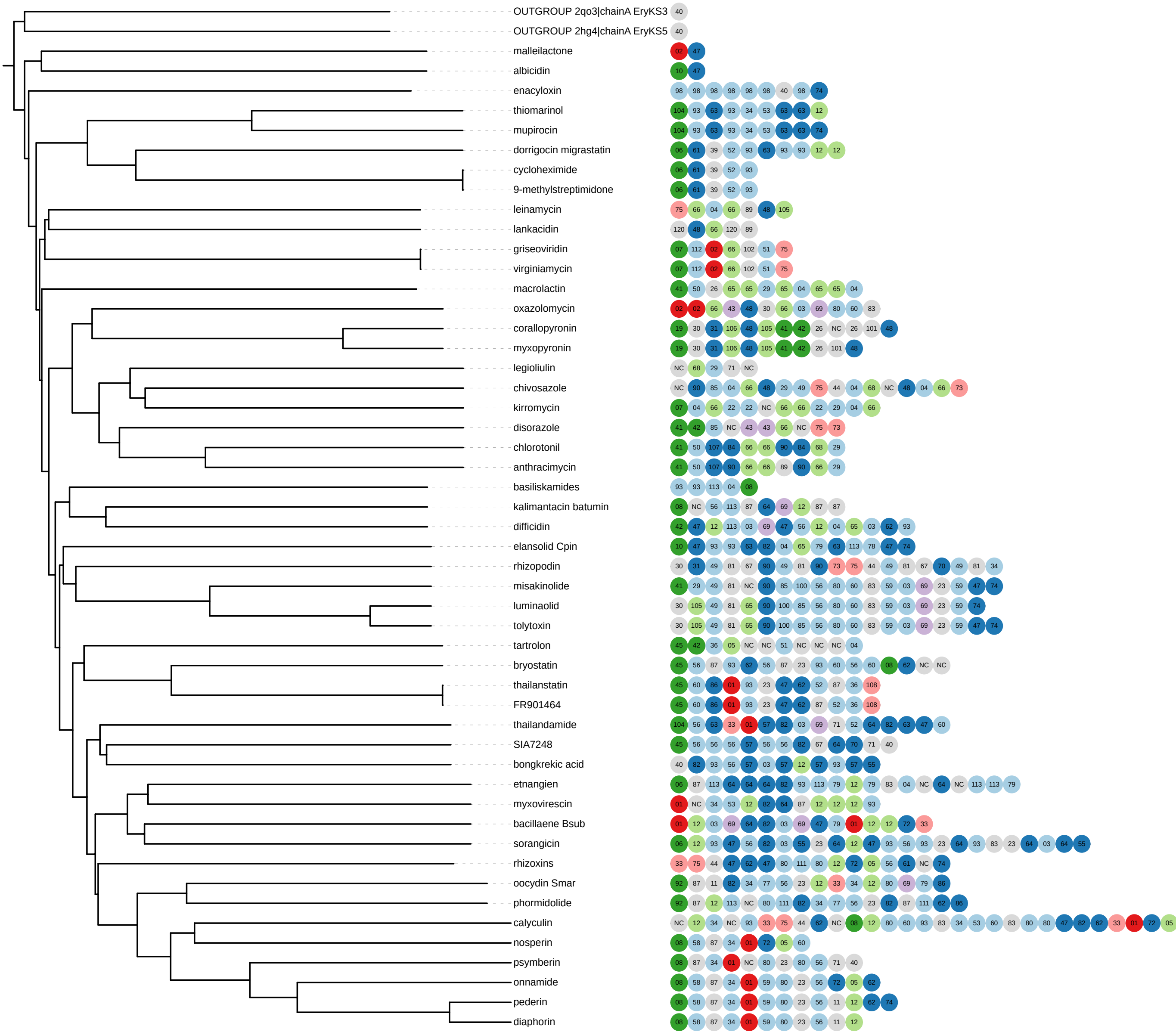

Supplementary Figure 2: Dendrogram representation of Figure 2A, showing conserved module blocks shared between characterized *trans*-AT PKS biosynthetic gene clusters. See Supplementary Table 2 for detailed number legend. Colors represent classes of phylogenetic clades. Red: amino acids; light blue: β-hydroxyl groups; light green: double bonds; dark blue: *E*-configured double bonds; light red: non-elongating KSs; dark green: starters; light purple: *Z*-configured double bonds; grey: others.

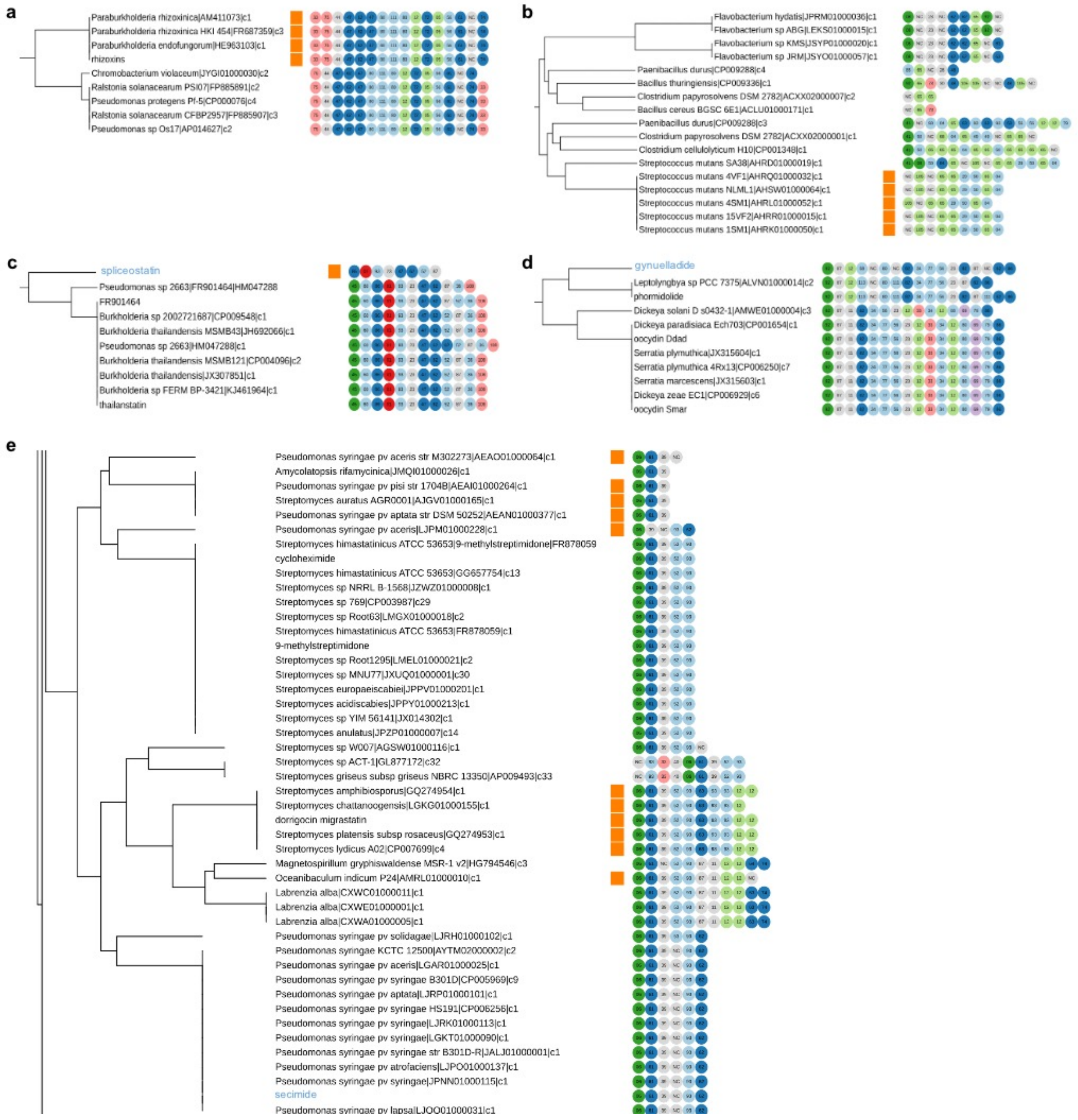

Supplementary Figure 3: Selection of novel PKS module blocks highlighted in the main text and families of PKS expanded through the isolation of novel polyketides in this study. (a) and (b) selection of novel PKS module blocks within the context of total assembly lines as depicted in the dendrogram. (c) spliceostatin-like PKSs. (d) phormidolide-like PKSs. (e) glutarimide PKSs. See Supplementary Table 2 for detailed number legend. Colors represent classes of phylogenetic clades. Red: amino acids; light blue: β-hydroxyl groups; light green: double bonds; dark blue: *E*-configured double bonds; light red: non-elongating KSs; dark green: starters; light purple: *Z*-configured double bonds; grey: others. Molecules linked to characterized biosynthetic gene clusters help identify families of *trans*-AT PKSs. Orange boxes indicate likely incomplete biosynthetic gene clusters as determined by their small distance to contig borders of less than 5 kb. An interactive representation of the dendrogram can be accesses here: https://itol.embl.de/tree/474115015487031585082885#.

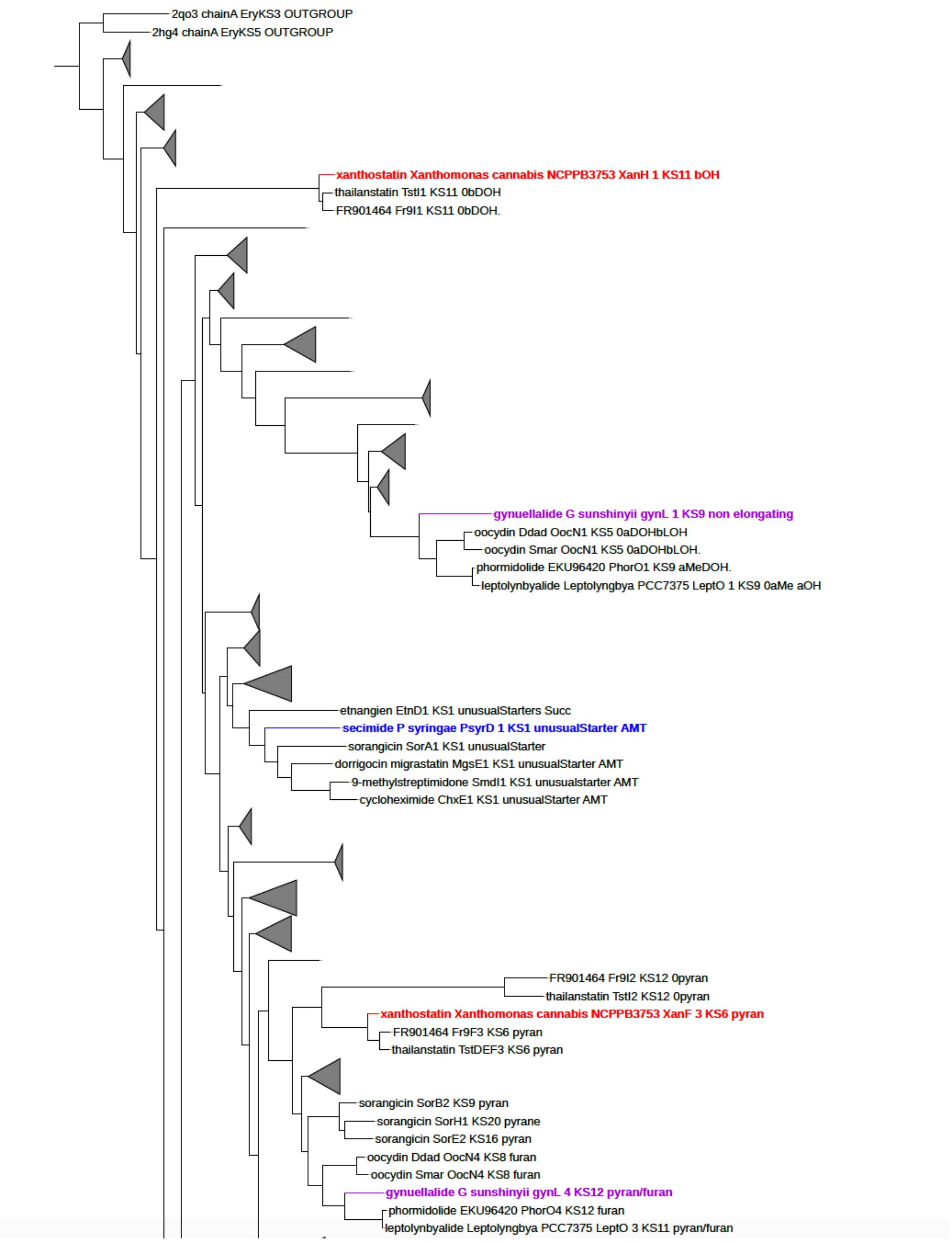
2qo3 chainA EryKS3 OUTGROUP
2hg4 chainA EryKS5 OUTGROUP
xanthostatin Xanthomonas cannabis NCPPB3753 XanH 1 KS11 bOH
thailanstatin TstI1 KS11 0bDOH
FR901464 Fr9I1 KS11 0bDOH.
gynuellalide G sunshinyii gynL 1 KS9 non elongating
oocydin Ddad OocN1 KS5 0aDOHbLOH
oocydin Smar OocN1 KS5 0aDOHbLOH.
phormidolide EKU96420 PhorO1 KS9 aMeDOH.
leptolynbyalide Leptolyngbya PCC7375 LeptO 1 KS9 0aMe aOH
etnangien EtnD1 KS1 unusualStarters Succ
secimide P syringae PsyrD 1 KS1 unusualStarter AMT
sorangicin SorA1 KS1 unusualStarter
dorrigocin migrastatin MgsE1 KS1 unusualStarter AMT
9-methylstreptimidone SmdI1 KS1 unusualstarter AMT
cycloheximide ChxE1 KS1 unusualStarter AMT
FR901464 Fr9I2 KS12 0pyran
thailanstatin TstI2 KS12 0pyran
xanthostatin Xanthomonas cannabis NCPPB3753 XanF 3 KS6 pyran
FR901464 Fr9F3 KS6 pyran
thailanstatin TstDEF3 KS6 pyran
sorangicin SorB2 KS9 pyran
sorangicin SorH1 KS20 pyrane
sorangicin SorE2 KS16 pyran
oocydin Ddad OocN4 KS8 furan
oocydin Smar OocN4 KS8 furan
gynuellalide G sunshinyii gynL 4 KS12 pyran/furan
phormidolide EKU96420 PhorO4 KS12 furan
leptolynbyalide Leptolyngbya PCC7375 LeptO 3 KS11 pyran/furan

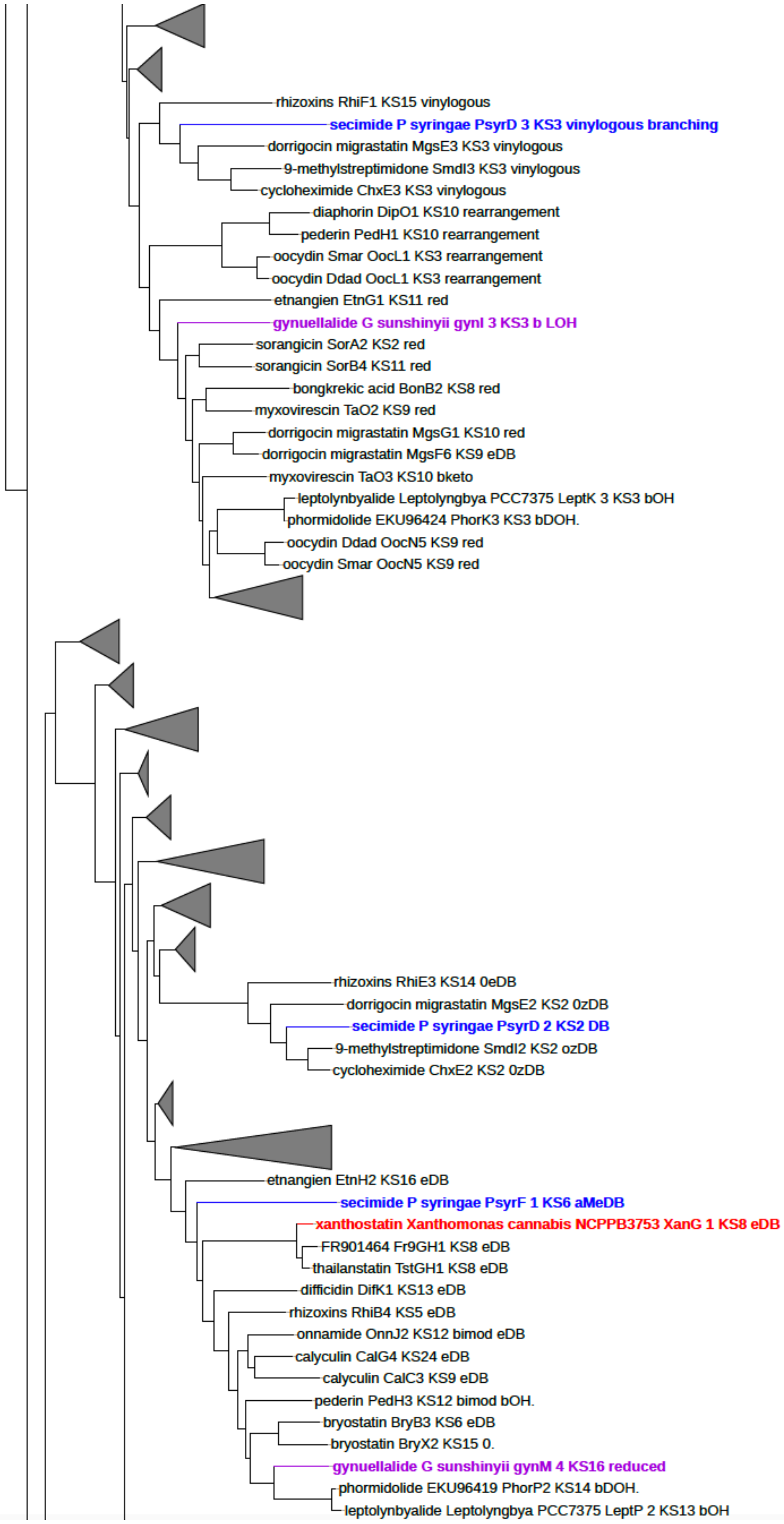
rhizoxins RhiF1 KS15 vinylogous
secimide P syringae PsyrD 3 KS3 vinylogous branching
dorrigocin migrastatin MgsE3 KS3 vinylogous
9-methylstreptimidone SmdI3 KS3 vinylogous
cycloheximide ChxE3 KS3 vinylogous
diaphorin DipO1 KS10 rearrangement
pederin PedH1 KS10 rearrangement
oocydin Smar OocL1 KS3 rearrangement
oocydin Ddad OocL1 KS3 rearrangement
etnangien EtnG1 KS11 red
gynuellalide G sunshinyii gynI 3 KS3 b LOH
sorangicin SorA2 KS2 red
sorangicin SorB4 KS11 red
bongkrekic acid BonB2 KS8 red
myxovirescin TaO2 KS9 red
dorrigocin migrastatin MgsG1 KS10 red
dorrigocin migrastatin MgsF6 KS9 eDB
myxovirescin TaO3 KS10 bketo
leptolynbyalide Leptolyngbya PCC7375 LeptK 3 KS3 bOH
phormidolide EKU96424 PhorK3 KS3 bDOH.
oocydin Ddad OocN5 KS9 red
oocydin Smar OocN5 KS9 red
rhizoxins RhiE3 KS14 0eDB
dorrigocin migrastatin MgsE2 KS2 0zDB
secimide P syringae PsyrD 2 KS2 DB
9-methylstreptimidone SmdI2 KS2 ozDB
cycloheximide ChxE2 KS2 0zDB
etnangien EtnH2 KS16 eDB
secimide P syringae PsyrF 1 KS6 aMeDB
xanthostatin Xanthomonas cannabis NCPPB3753 XanG 1 KS8 eDB
FR901464 Fr9GH1 KS8 eDB
thailanstatin TstGH1 KS8 eDB
difficidin DifK1 KS13 eDB
rhizoxins RhiB4 KS5 eDB
onnamide OnnJ2 KS12 bimod eDB
calyculin CalG4 KS24 eDB
calyculin CalC3 KS9 eDB
pederin PedH3 KS12 bimod bOH.
bryostatin BryB3 KS6 eDB
bryostatin BryX2 KS15 0.
gynuellalide G sunshinyii gynM 4 KS16 reduced
phormidolide EKU96419 PhorP2 KS14 bDOH.
leptolynbyalide Leptolyngbya PCC7375 LeptP 2 KS13 bOH

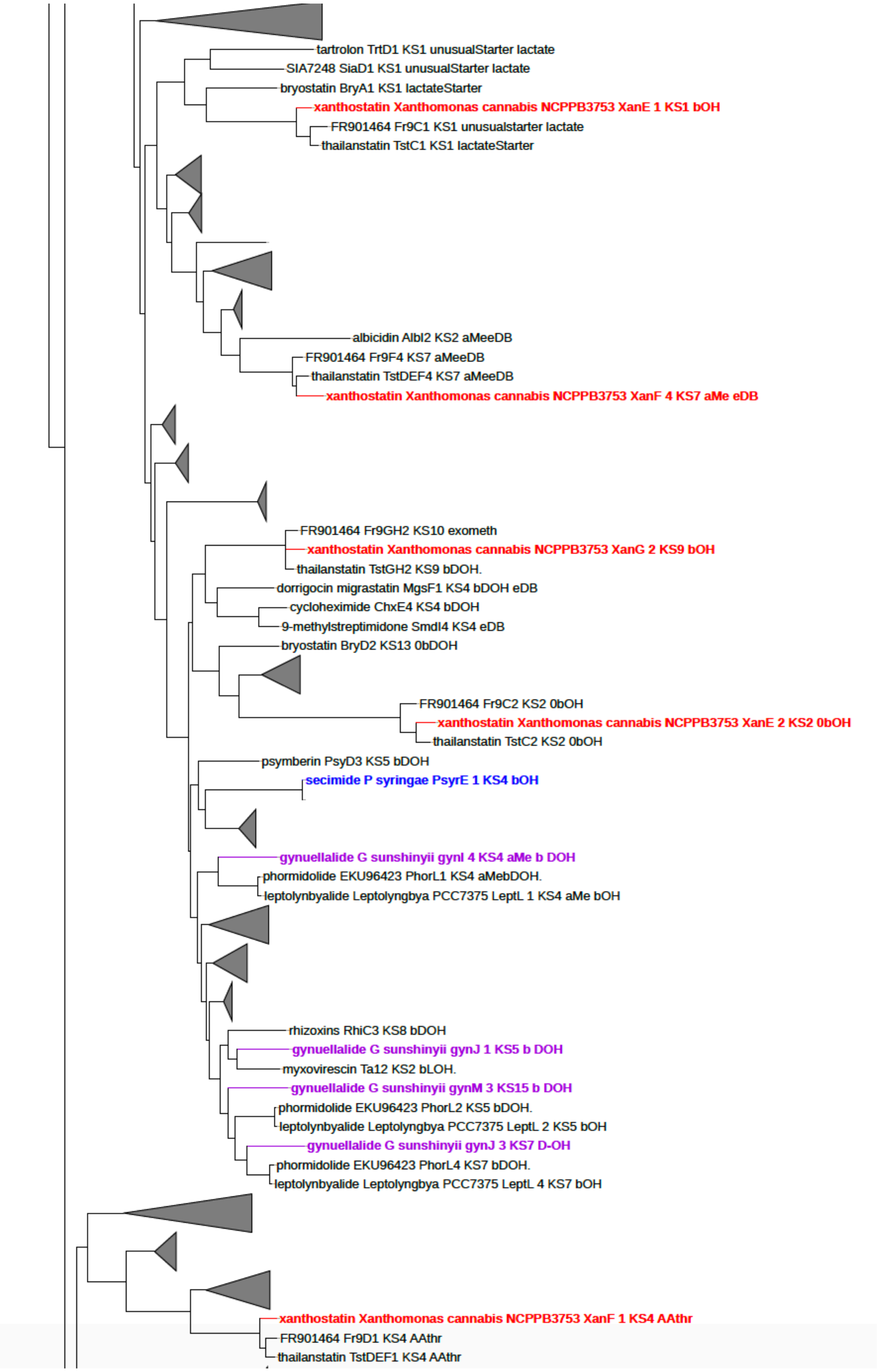
tartrolon TrtD1 KS1 unusualStarter lactate
SIA7248 SiaD1 KS1 unusualStarter lactate
bryostatin BryA1 KS1 lactateStarter
xanthostatin Xanthomonas cannabis NCPPB3753 XanE 1 KS1 bOH
FR901464 Fr9C1 KS1 unusualstarter lactate
thailanstatin TstC1 KS1 lactateStarter
albicidin AlbI2 KS2 aMeeDB
FR901464 Fr9F4 KS7 aMeeDB
thailanstatin TstDEF4 KS7 aMeeDB
xanthostatin Xanthomonas cannabis NCPPB3753 XanF 4 KS7 aMe eDB
FR901464 Fr9GH2 KS10 exometh
xanthostatin Xanthomonas cannabis NCPPB3753 XanG 2 KS9 bOH
thailanstatin TstGH2 KS9 bDOH.
dorrigocin migrastatin MgsF1 KS4 bDOH eDB
cycloheximide ChxE4 KS4 bDOH
9-methylstreptimidone SmdI4 KS4 eDB
bryostatin BryD2 KS13 0bDOH
FR901464 Fr9C2 KS2 0bOH
xanthostatin Xanthomonas cannabis NCPPB3753 XanE 2 KS2 0bOH
thailanstatin TstC2 KS2 0bOH
psymberin PsyD3 KS5 bDOH
secimide P syringae PsyrE 1 KS4 bOH
gynuellalide G sunshinyii gynI 4 KS4 aMe b DOH
phormidolide EKU96423 PhorL1 KS4 aMebDOH.
leptolynbyalide Leptolyngbya PCC7375 LeptL 1 KS4 aMe bOH
rhizoxins RhiC3 KS8 bDOH
gynuellalide G sunshinyii gynJ 1 KS5 b DOH
myxovirescin Ta12 KS2 bLOH.
gynuellalide G sunshinyii gynM 3 KS15 b DOH
phormidolide EKU96423 PhorL2 KS5 bDOH.
leptolynbyalide Leptolyngbya PCC7375 LeptL 2 KS5 bOH
gynuellalide G sunshinyii gynJ 3 KS7 D-OH
phormidolide EKU96423 PhorL4 KS7 bDOH.
leptolynbyalide Leptolyngbya PCC7375 LeptL 4 KS7 bOH
xanthostatin Xanthomonas cannabis NCPPB3753 XanF 1 KS4 AAthr
FR901464 Fr9D1 KS4 AAthr
thailanstatin TstDEF1 KS4 AAthr

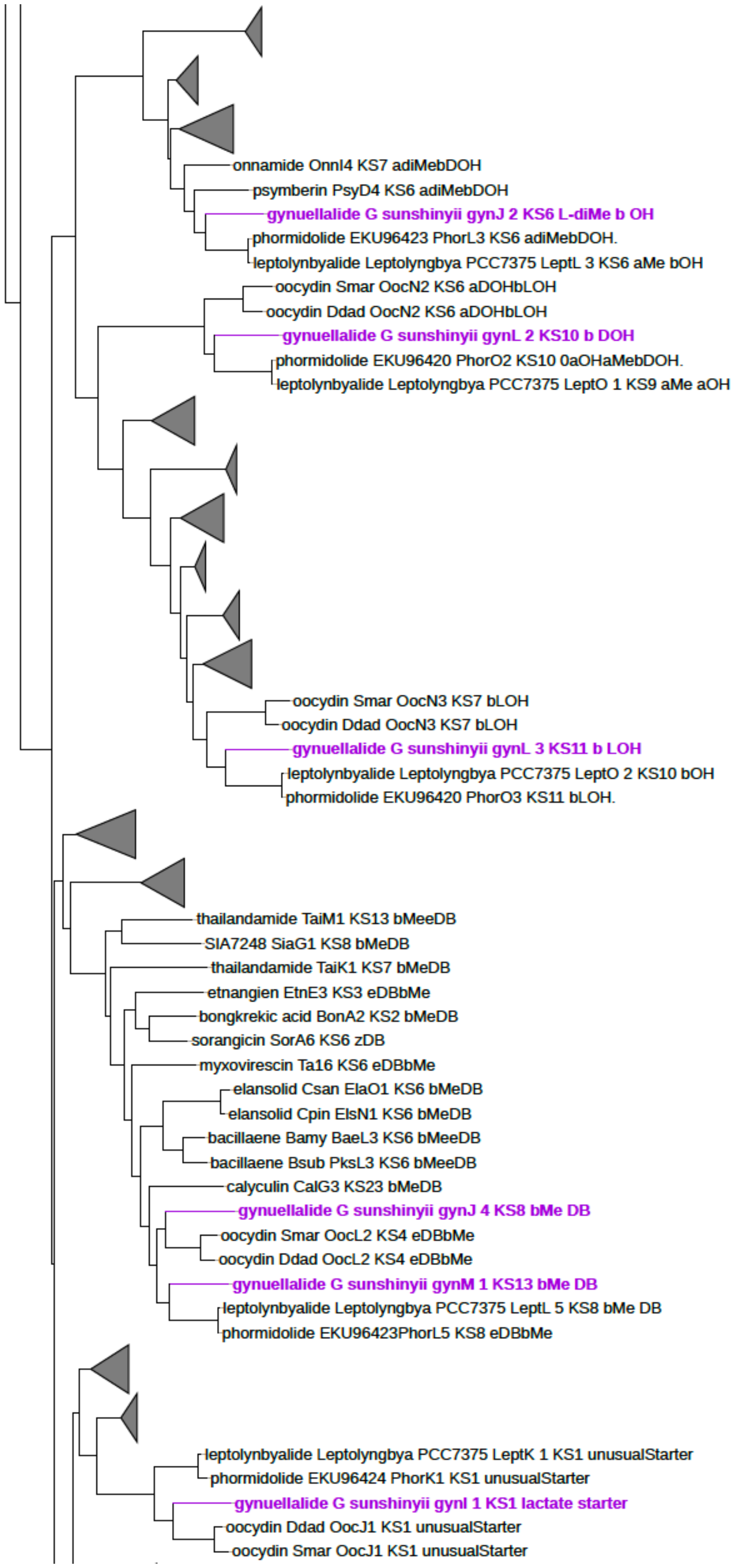

onnamide OnnI4 KS7 adiMebDOH
psymberin PsyD4 KS6 adiMebDOH
gynuellalide G sunshinyii gynJ 2 KS6 L-diMe b OH
phormidolide EKU96423 PhorL3 KS6 adiMebDOH.
leptolynbyalide Leptolyngbya PCC7375 LeptL 3 KS6 aMe bOH
oocydin Smar OocN2 KS6 aDOHbLOH
oocydin Ddad OocN2 KS6 aDOHbLOH
gynuellalide G sunshinyii gynL 2 KS10 b DOH
phormidolide EKU96420 PhorO2 KS10 0aOHaMebDOH.
leptolynbyalide Leptolyngbya PCC7375 LeptO 1 KS9 aMe aOH
oocydin Smar OocN3 KS7 bLOH
oocydin Ddad OocN3 KS7 bLOH
gynuellalide G sunshinyii gynL 3 KS11 b LOH
leptolynbyalide Leptolyngbya PCC7375 LeptO 2 KS10 bOH
phormidolide EKU96420 PhorO3 KS11 bLOH.
thailandamide TaiM1 KS13 bMeeDB
SIA7248 SiaG1 KS8 bMeDB
thailandamide TaiK1 KS7 bMeDB
etnangien EtnE3 KS3 eDBbMe
bongkrekic acid BonA2 KS2 bMeDB
sorangicin SorA6 KS6 zDB
myxovirescin Ta16 KS6 eDBbMe
elansolid Csan ElaO1 KS6 bMeDB
elansolid Cpin ElsN1 KS6 bMeDB
bacillaene Bamy BaeL3 KS6 bMeeDB
bacillaene Bsub PksL3 KS6 bMeeDB
calyculin CalG3 KS23 bMeDB
gynuellalide G sunshinyii gynJ 4 KS8 bMe DB
oocydin Smar OocL2 KS4 eDBbMe
oocydin Ddad OocL2 KS4 eDBbMe
gynuellalide G sunshinyii gynM 1 KS13 bMe DB
leptolynbyalide Leptolyngbya PCC7375 LeptL 5 KS8 bMe DB
phormidolide EKU96423PhorL5 KS8 eDBbMe
leptolynbyalide Leptolyngbya PCC7375 LeptK 1 KS1 unusualStarter
phormidolide EKU96424 PhorK1 KS1 unusualStarter
gynuellalide G sunshinyii gynI 1 KS1 lactate starter
oocydin Ddad OocJ1 KS1 unusualStarter
oocydin Smar OocJ1 KS1 unusualStarter

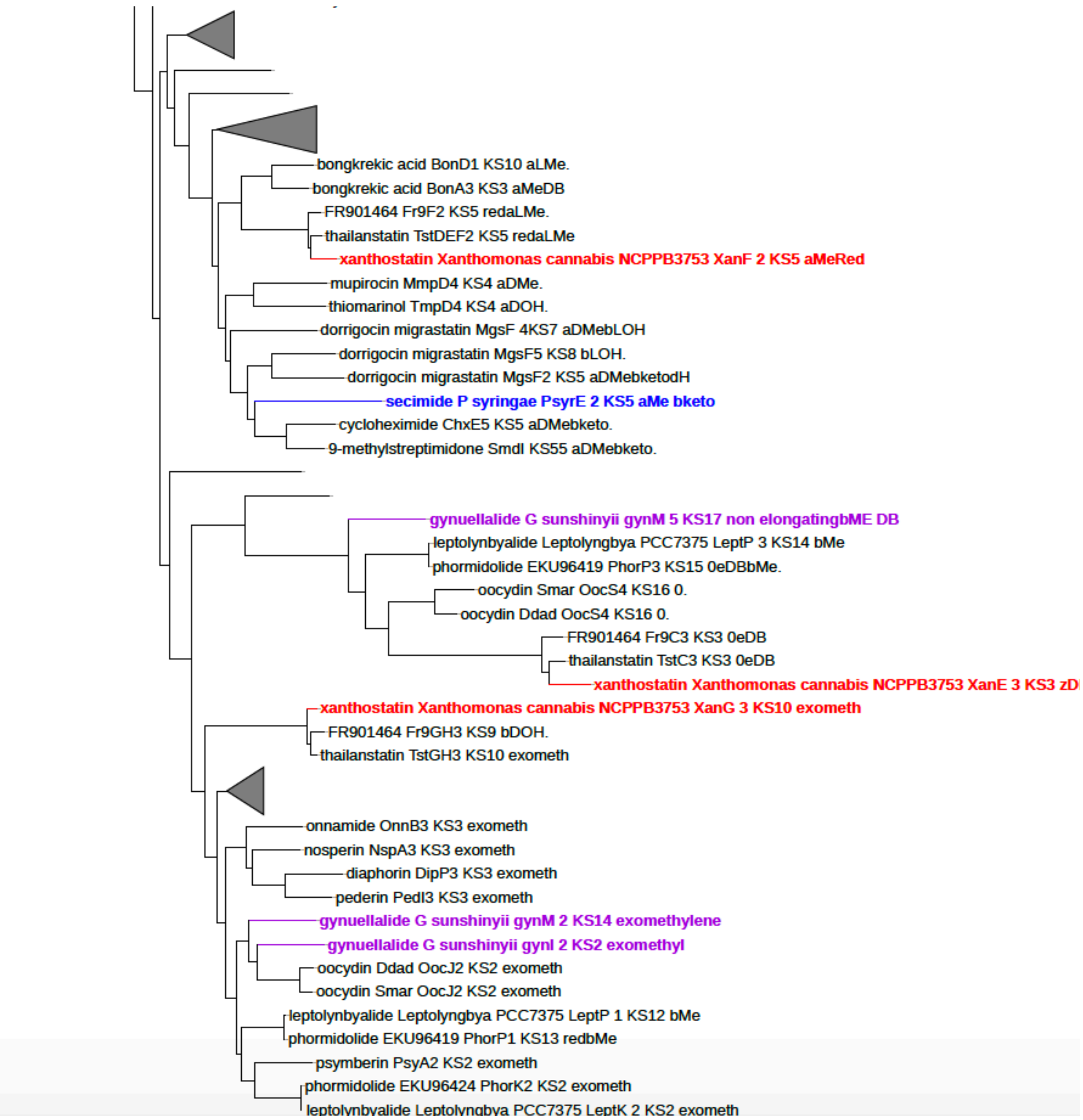

Supplementary Figure 4: Validation of the *trans*PACT workflow using the three biosynthetic gene clusters for the new polyketides described in this study as an example. Maximum likelihood phylogenetic tree computed from a MUSCLE alignment of all *trans*-AT PKS KS sequences from the 49 characterized *trans*-AT PKSs described by Helfrich and Piel,[1] plus all KSs extracted from the secimide, gynuellalide, and spliceostatin PKSs (denoted here as 'xanthostatin') characterized in this study. Phylogenetic placement of the KSs from the three example compounds is in good agreement with the transPACT results.

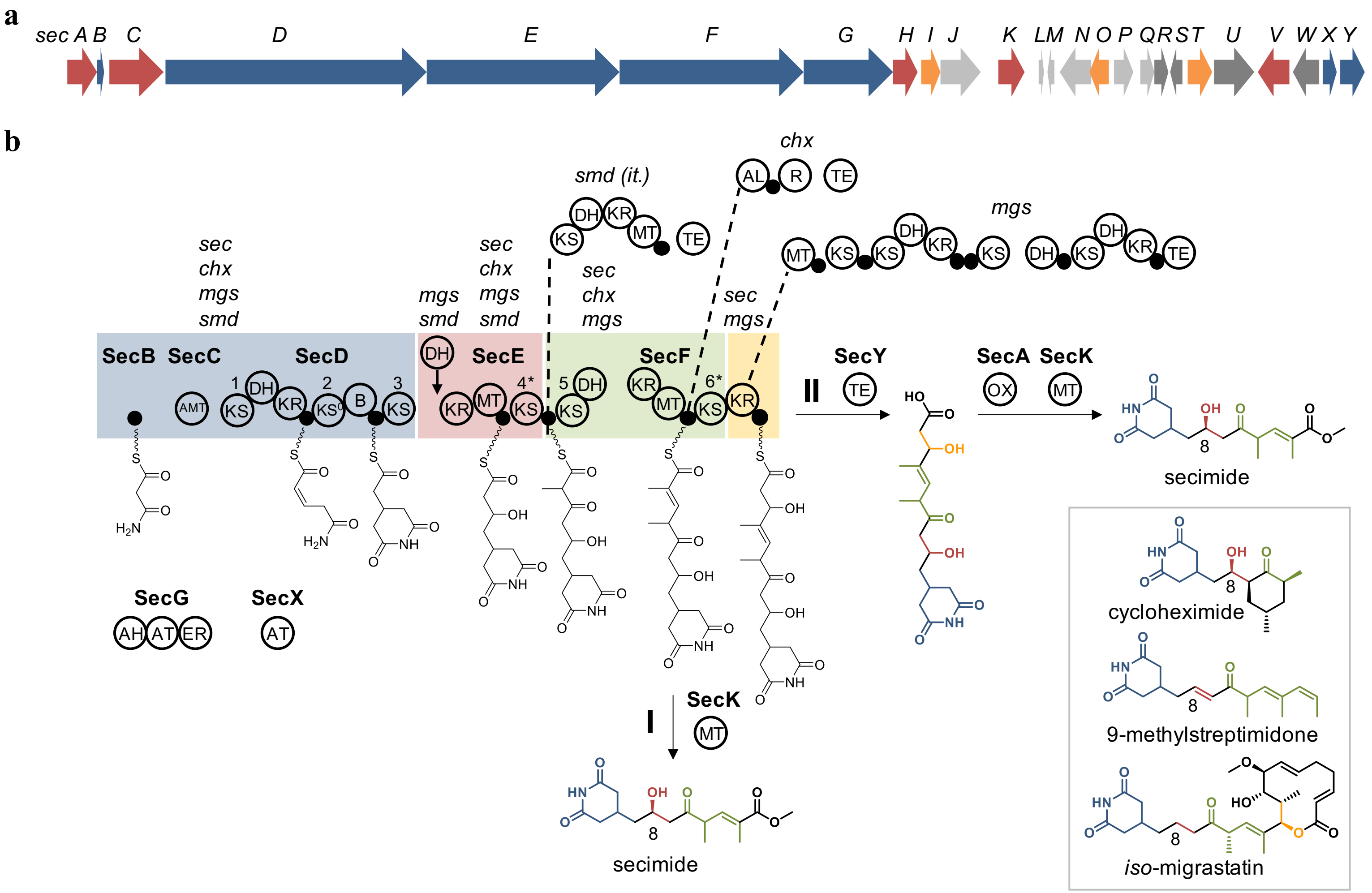

Supplementary Figure 5: Secimide biosynthetic model. (**a**) The *sec* BGC. blue: PKS genes, orange: transporter genes, light grey: hypothetical genes, red: precursor biosynthesis and tailoring genes, dark grey: genes involved in replication, DNA biosynthesis and repair. (**b**) The secimide PKS and alternative models for secimide biosynthesis. The *sec* proteins are shown with bold labels, other labels refer to genes encoding the core PKSs of the related cycloheximide (*chx*), migrastatin (*mgs*), and 9-methylstreptimidone (*smd*) pathways. Proteins with multiple labels have orthologs in the corresponding pathways. Alternative hypothesis for the final steps: I) the last module is skipped and the intermediate converted to the ester by SecK or II) the polyketide backbone is shortened after it is completely assembled putatively catalyzed by the dioxygenase SecA and then methylated by SecK. *It.*, iterative module. Colors represent corresponding substructures of the polyketides.

a

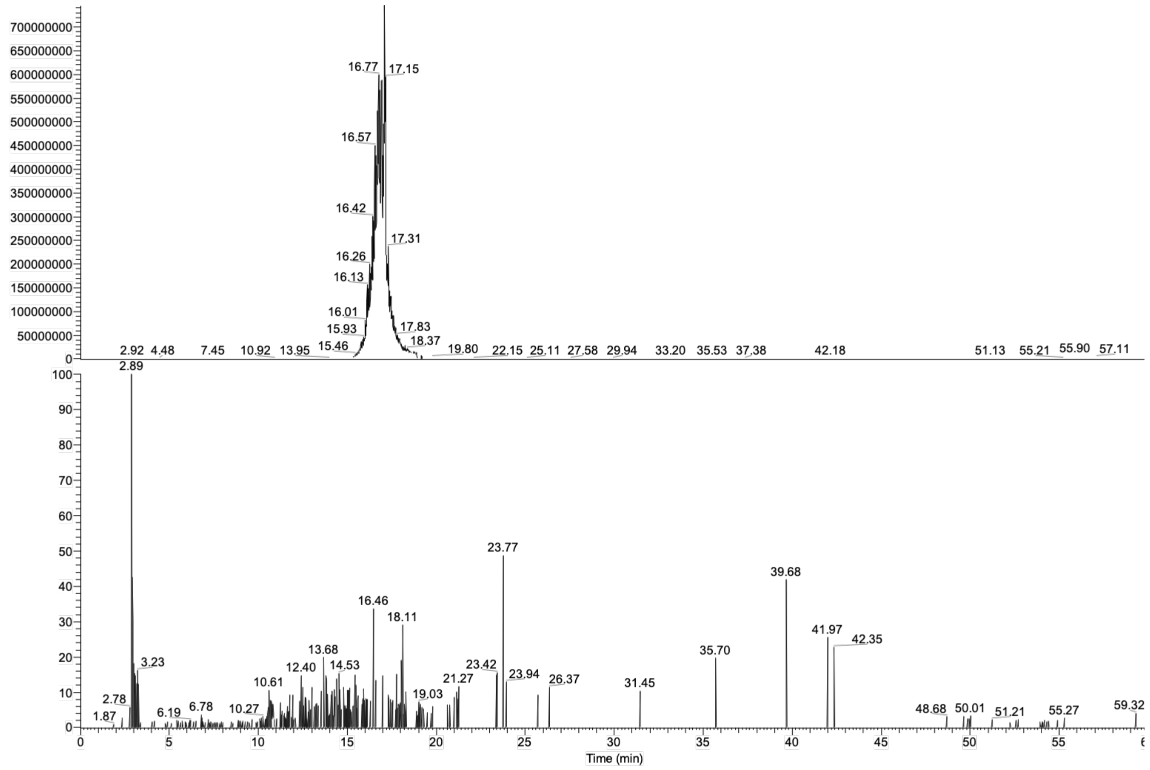

b

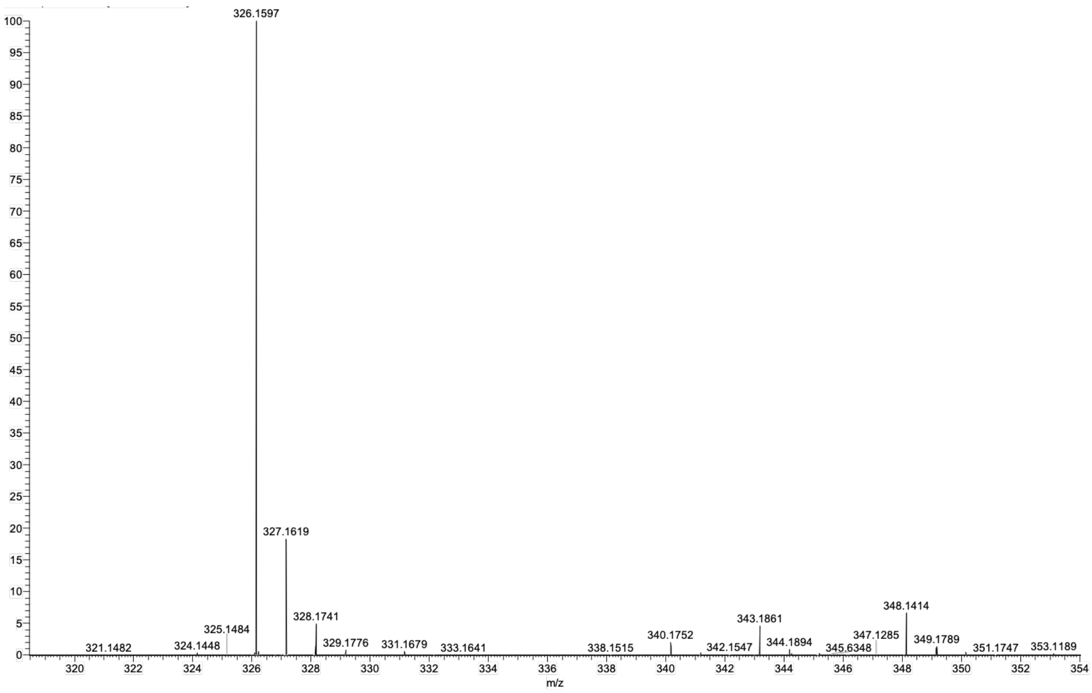

Supplementary Figure 6: HR-LCMS data of the extracts of the *P. syringae* wild type (WT) and PKS knockout strains. (**a**) Extracted ion chromatogram (*m/z* 326.15-326.16) of WT supernatant extract (upper) and PKS-knockout supernatant extract (lower); (**b**) Mass spectrum of secimide from *P. syringae* pv. *syringae*.

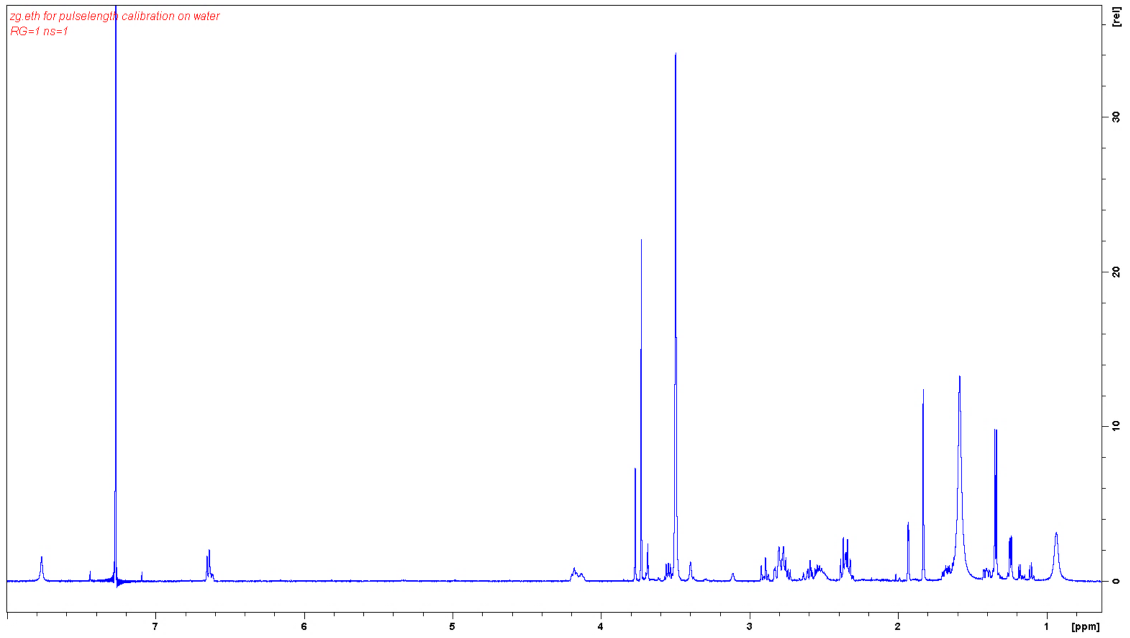

Supplementary Figure 7: ^{1}H NMR spectrum of secimide from *P. syringae* in $CDCl_3$.

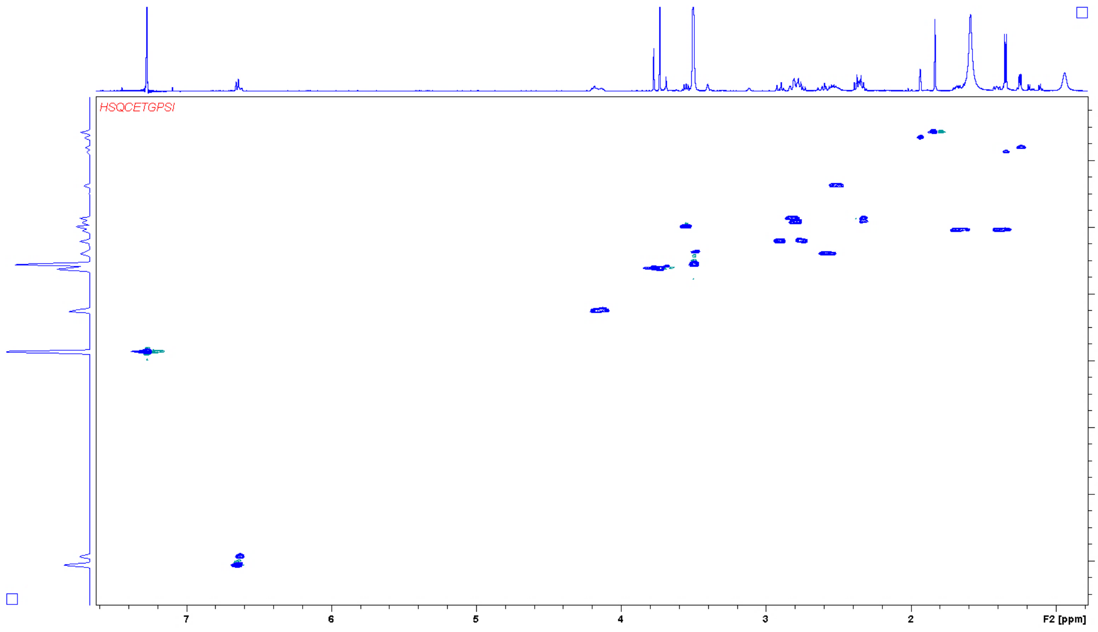

Supplementary Figure 8: HSQC spectrum of secimide from *P. syringae* in $CDCl_3$.

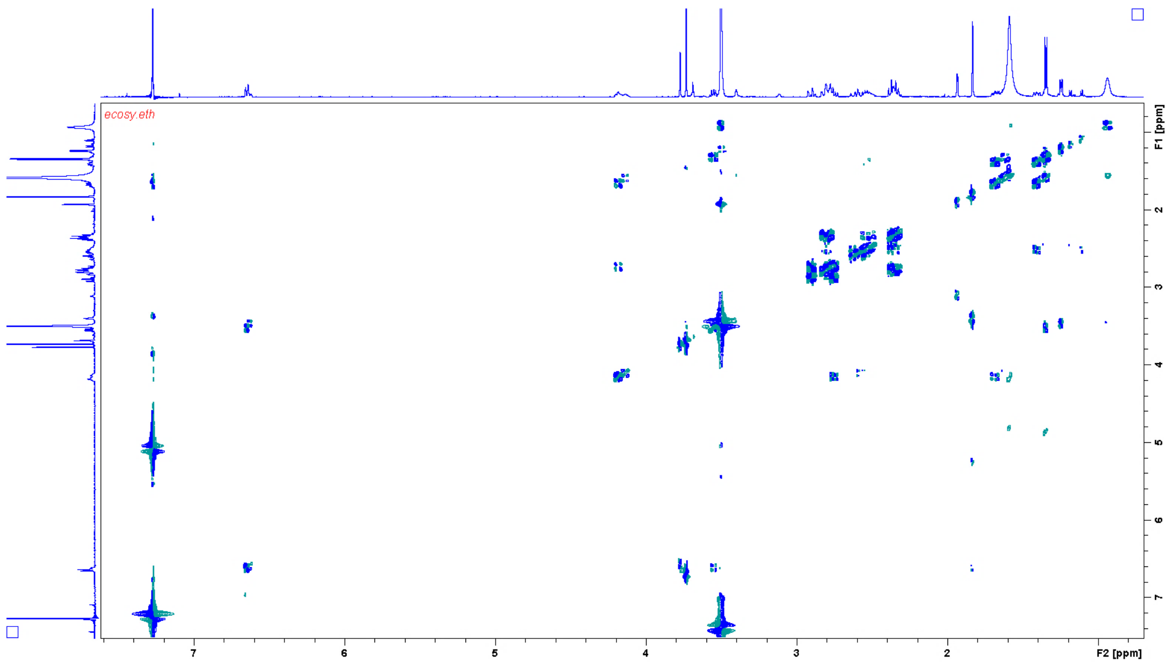

Supplementary Figure 9: COSY spectrum of secimide from *P. syringae* in $CDCl_3$.

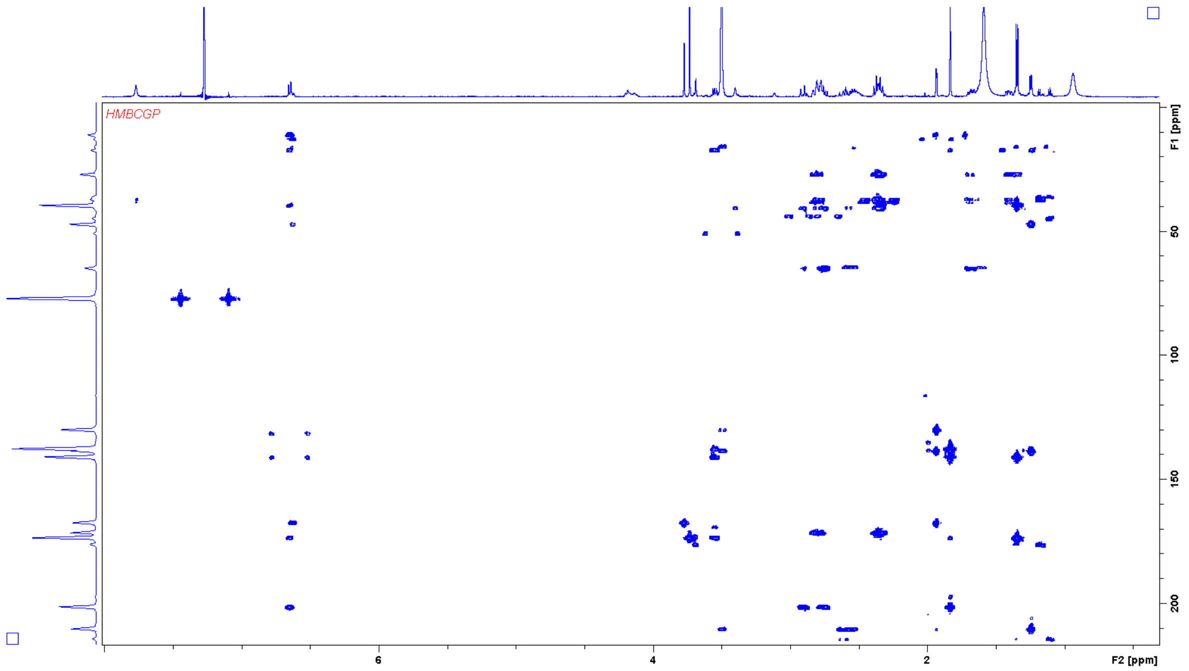

Supplementary Figure 10: HMBC spectrum of secimide from *P. syringae* in $CDCl_3$.

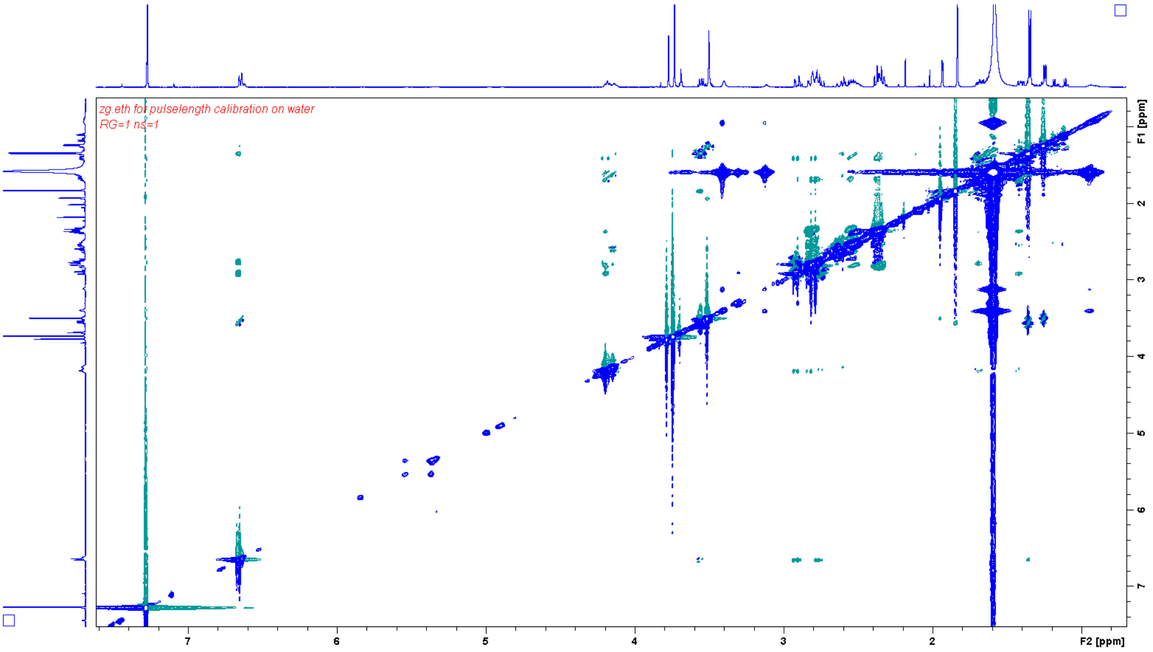

Supplementary Figure 11: NOESY spectrum of secimide from *P. syringae* in CDCl3.

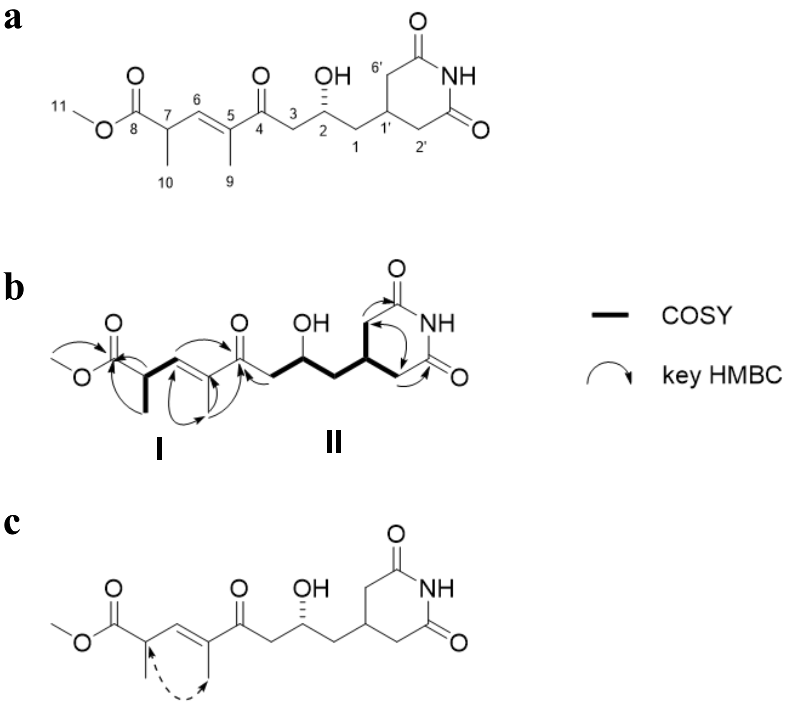

Supplementary Figure 12: Structure elucidation of secimide. (a) Structure of secimide. (**b**) COSY and key HMBC correlations of secimide. (**c**) Key NOESY correlation of secimide.

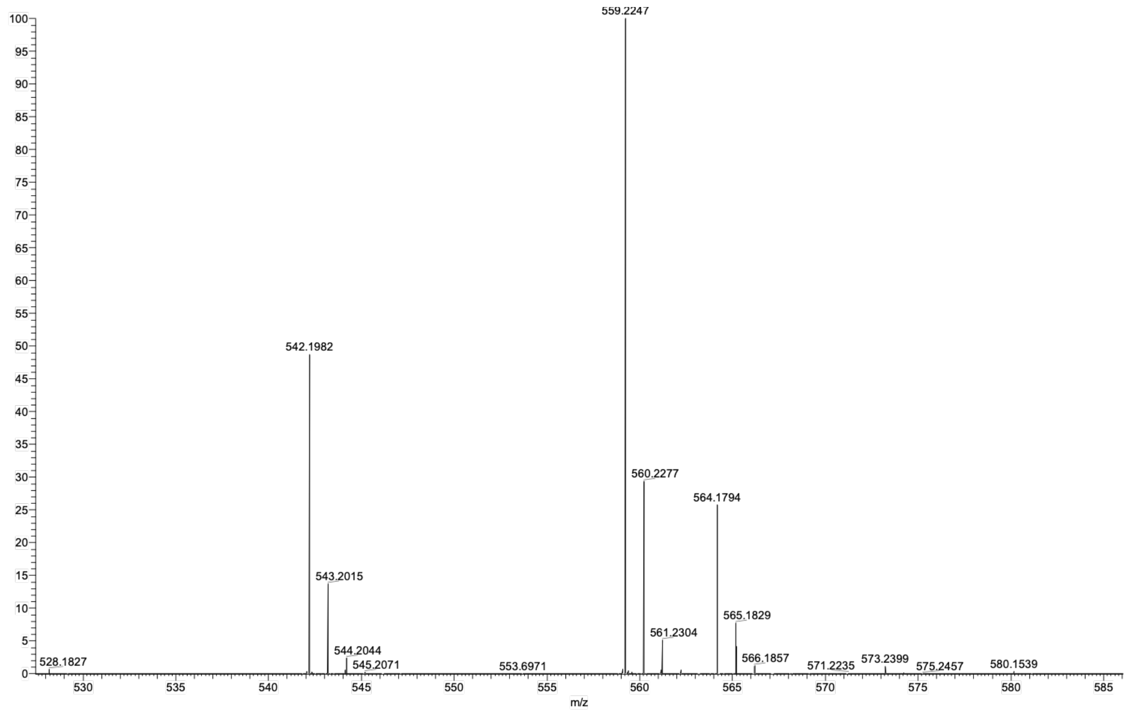

Supplementary Figure 13: HR-ESIMS data of secimide (*R*)-MTPA ester (*m*/*z* 542.1982 $[M+H]^+$, *m*/*z* 559.2247 $[M+NH_4]^+$, *m*/*z* 564.1794 $[M+Na]^+$).

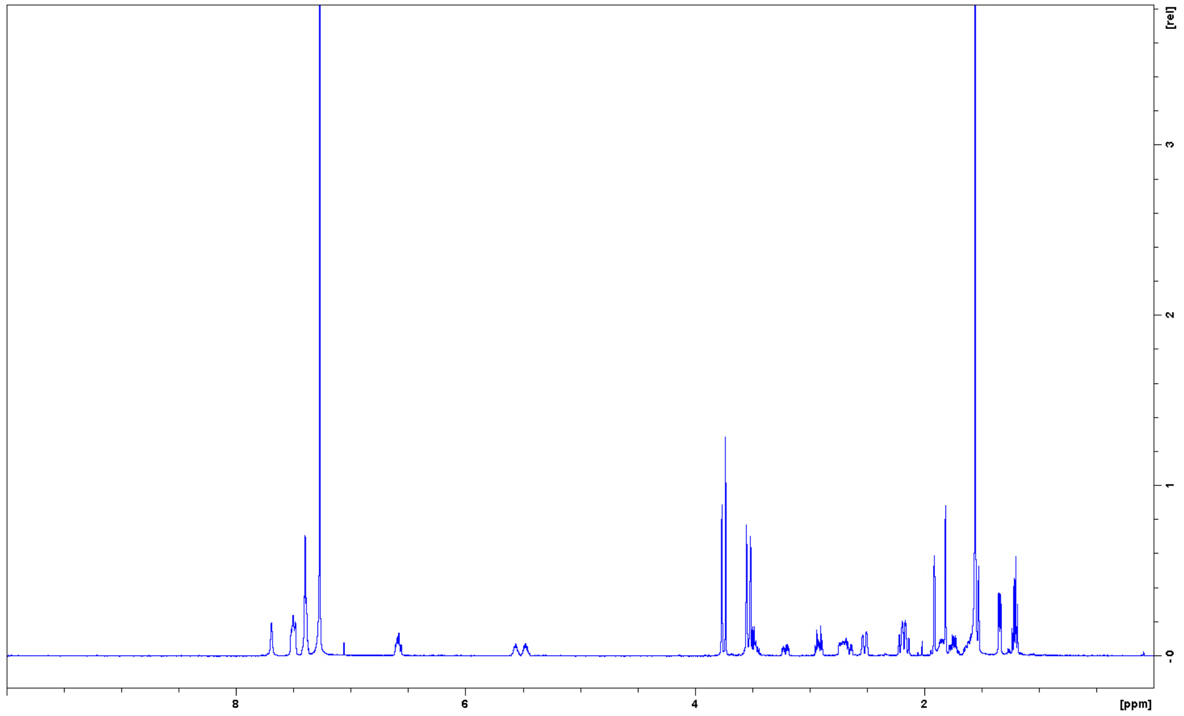

Supplementary Figure 14: ^1H NMR spectrum of secimide (*R*)-MTPA ester in $CDCl_3$.

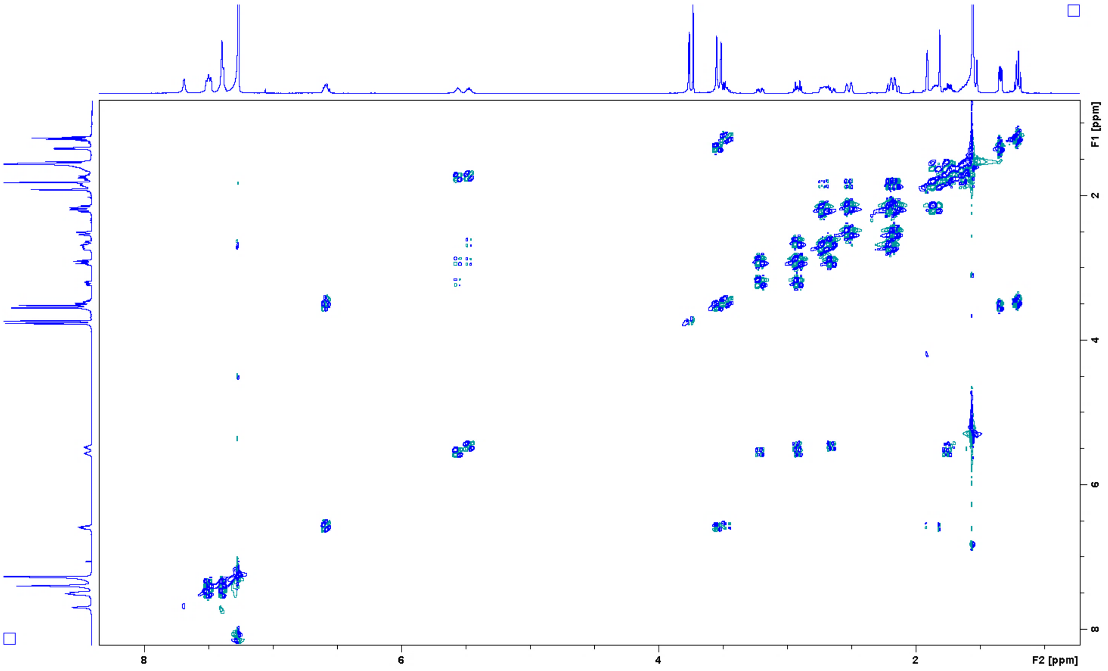

Supplementary Figure 15: COSY spectrum of secimide (*R*)-MTPA ester in $CDCl_3$.

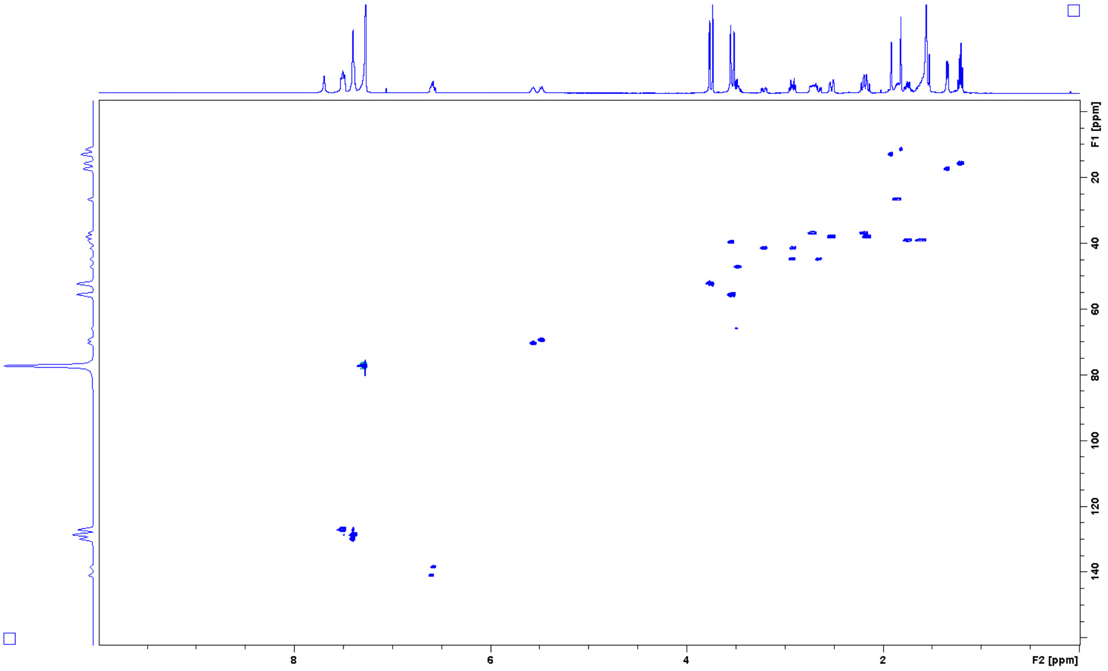

Supplementary Figure 16: HSQC spectrum of secimide (*R*)-MTPA ester in $CDCl_3$.

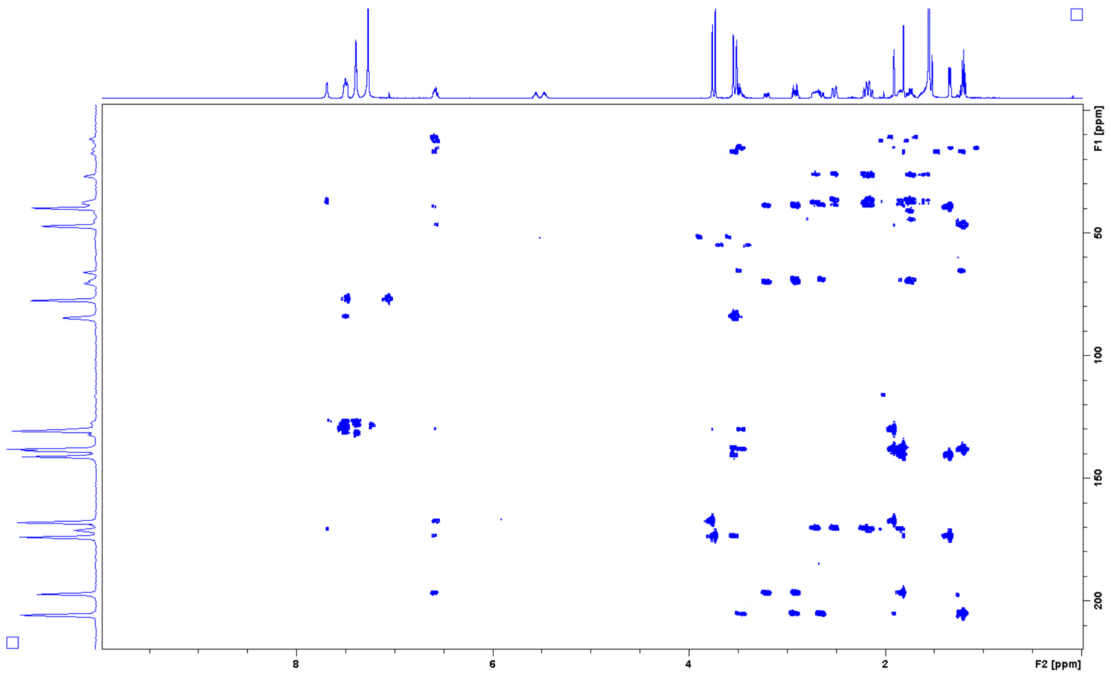

Supplementary Figure 17: HMBC spectrum of secimide (*R*)-MTPA ester in $CDCl_3$.

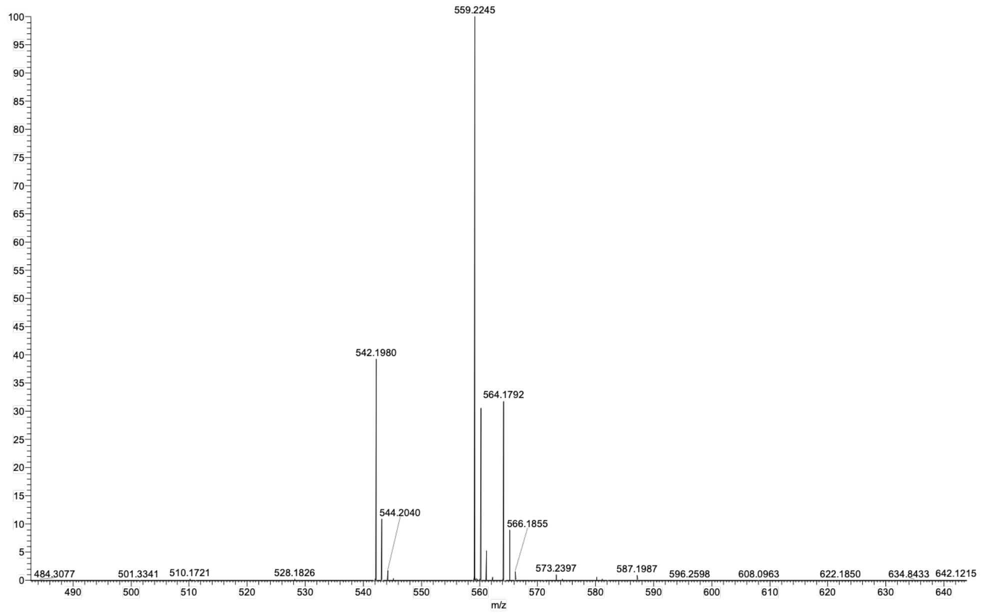

Supplementary Figure 18: HR-ESIMS data of secimide (*S*)-MTPA ester (*m*/*z* 542.1980 $[M+H]^+$, *m*/*z* 559.2245 $[M+NH_4]^+$, *m*/*z* 564.1792 $[M+Na]^+$).

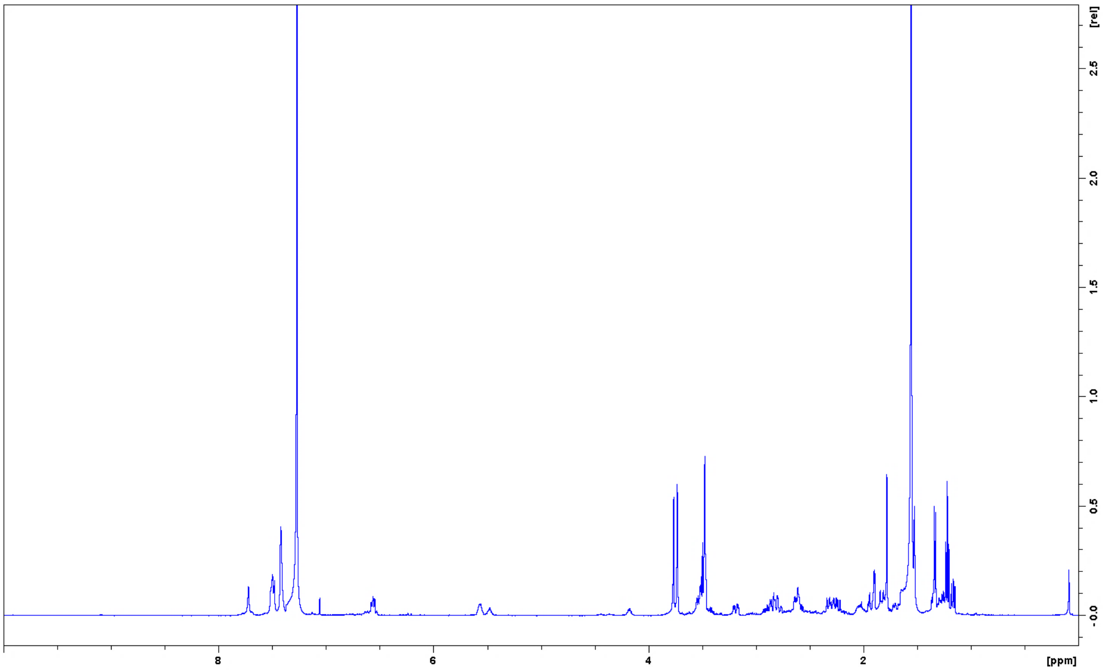

Supplementary Figure 19: ^{1}H NMR spectrum of secimide (*S*)-MTPA ester in CDCl$_3$.

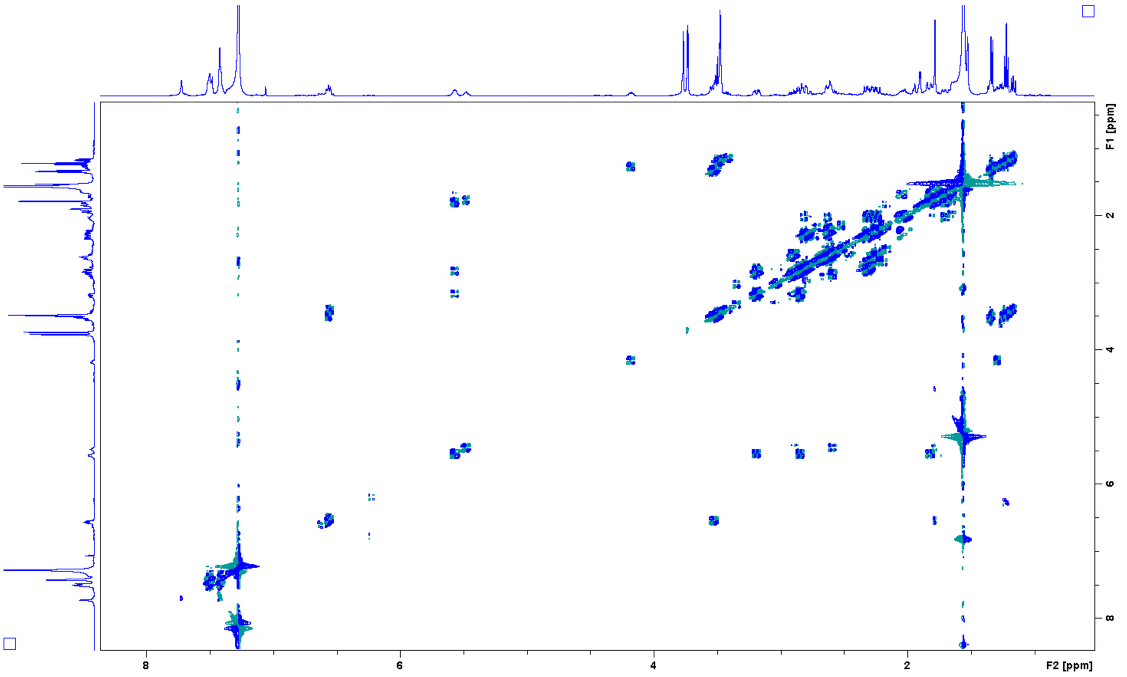

Supplementary Figure 20: COSY spectrum of secimide (*S*)-MTPA ester in CDCl$_3$.

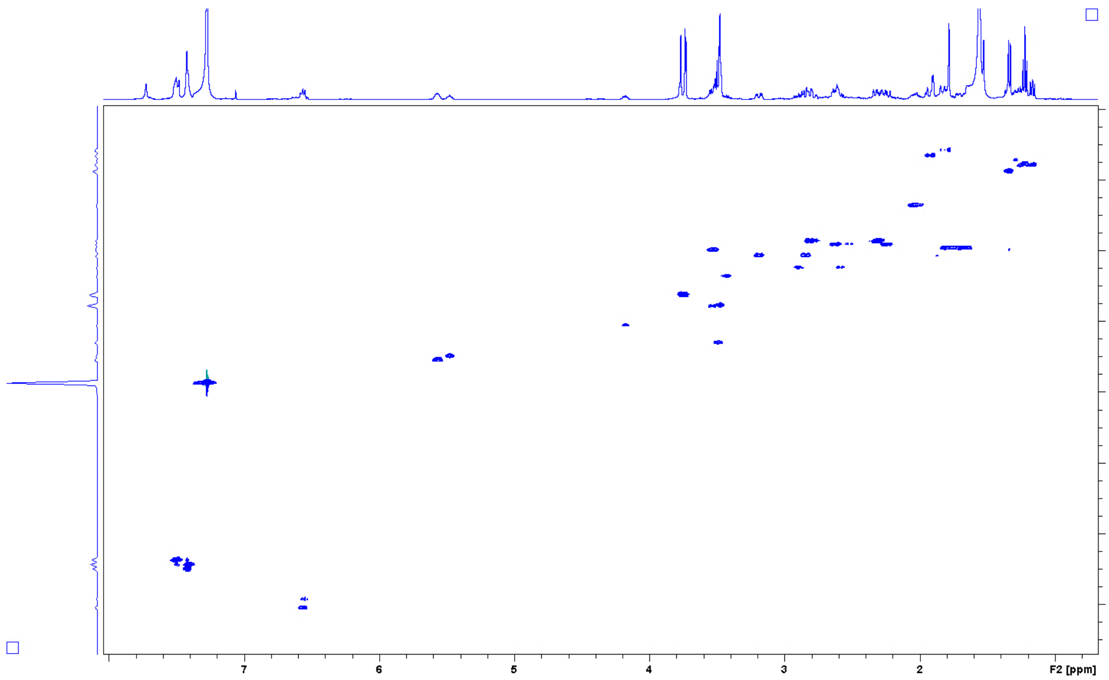

Supplementary Figure 21: HSQC spectrum of secimide (*S*)-MTPA ester in $CDCl_3$.

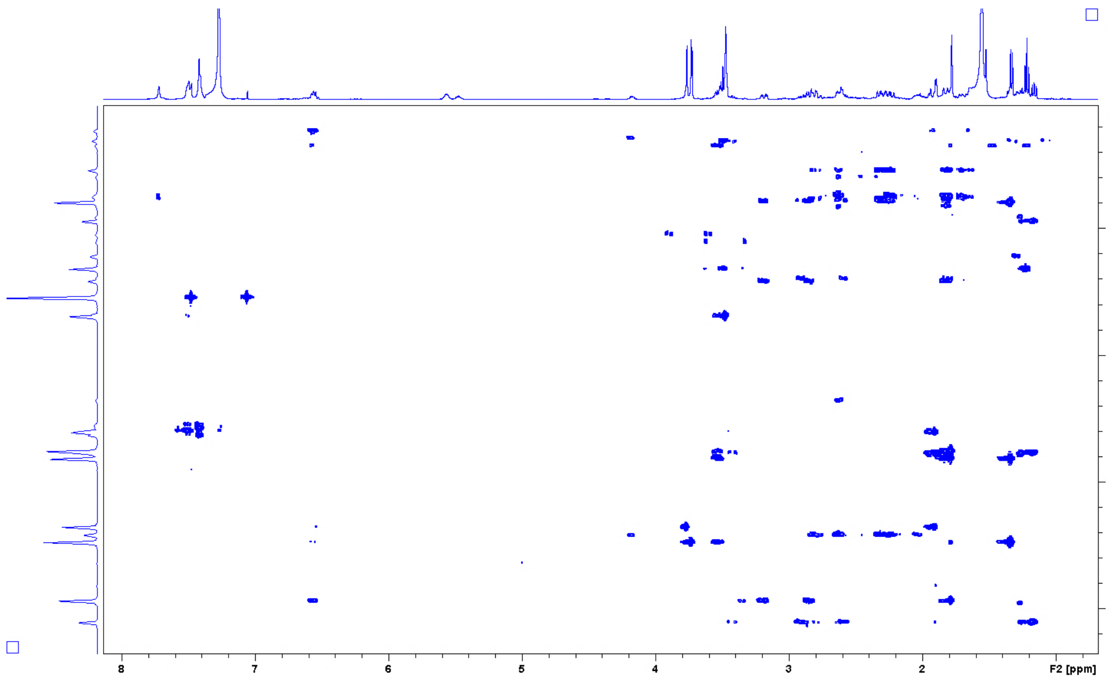

Supplementary Figure 22: HMBC spectrum of secimide (*S*)-MTPA ester in $CDCl_3$.

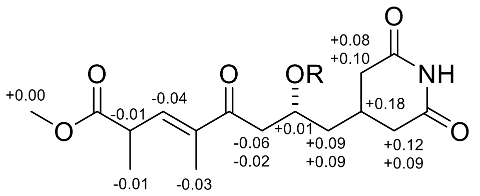

R = MTPA

Supplementary Figure 23: δ$_{S\text{-}R}$ values of secimide MTPA esters

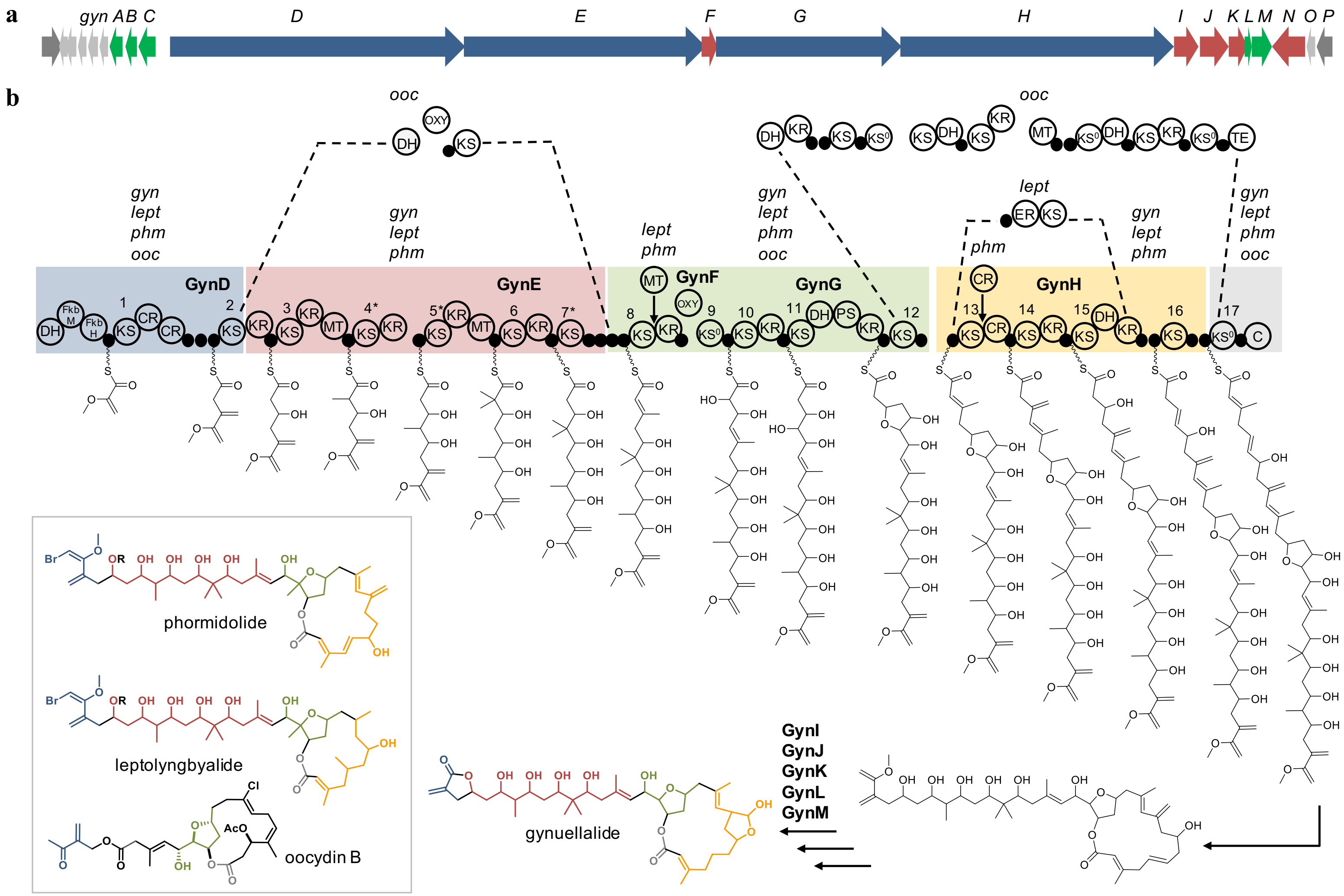

Supplementary Figure 24: Gynuellalide biosynthetic model. (**a**) **The** *gyn* BGC. blue: PKS core biosynthetic genes, light grey: hypothetical genes, light blue: regulatory gene, dark grey: regulatory genes, red: tailoring genes, green: β-branching cassette. (**b**) The gynuellalide PKS and biosynthetic model for gynuellalide biosynthesis. The *gyn* proteins are shown with bold labels, other labels refer to genes encoding the core PKSs of the related phormidolide (*phm*), leptolyngbyalide (*lept*), and oocydin (*ooc*) pathways. Proteins with multiple labels have orthologs in the corresponding pathways. Colors were used to highlight substructures of the associated polyketides. R: fatty acyl-chain found in phormidolides and leptolyngbyalides.

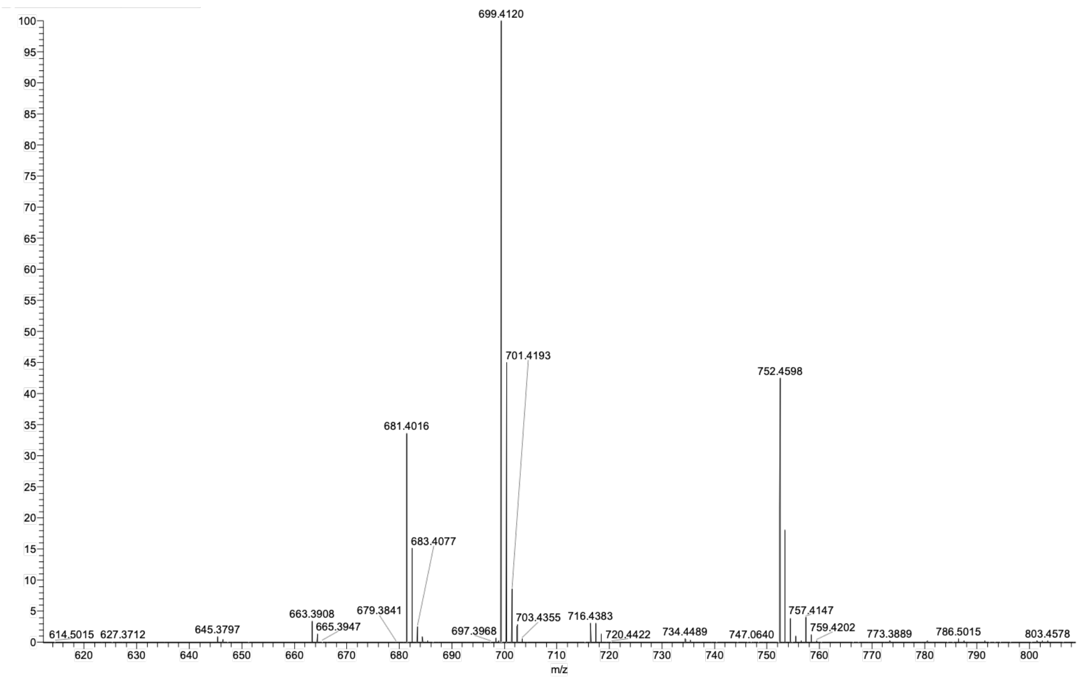

Supplementary Figure 25: HR-ESI-MS spectrum of gynuellalide (*m*/*z* 757.4147 $[M+Na]^+$, *m*/*z* 752.4598 $[M+NH_4]^+$, *m*/*z* 699.4120 $[M+H-H_2O]^+$, *m*/*z* 681.4016 $[M+H-2H_2O]^+$).

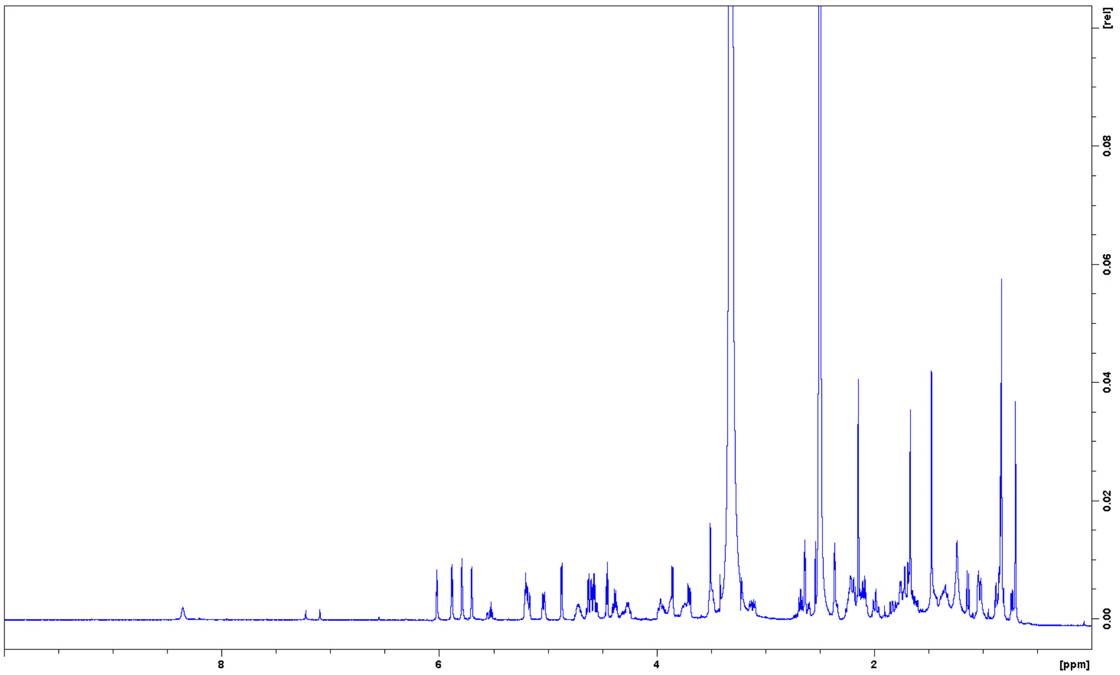

Supplementary Figure 26: ^{1}H NMR spectrum of gynuellalide in DMSO-d_6.

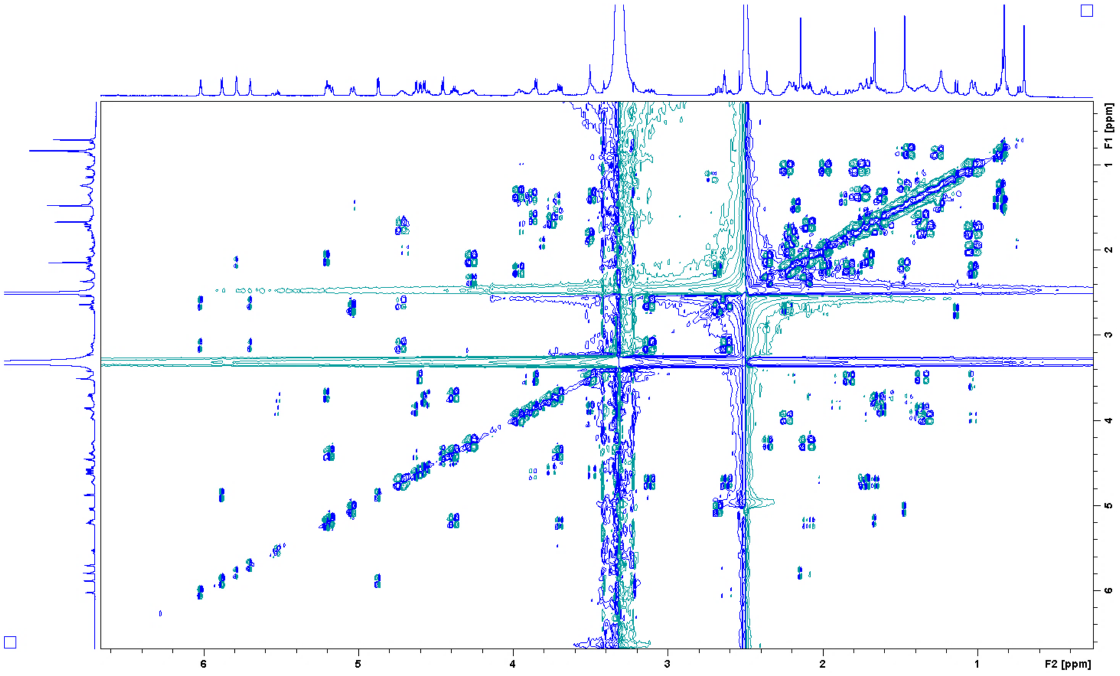

Supplementary Figure 27: COSY spectrum of gynuellalide in DMSO-*d*$_6$.

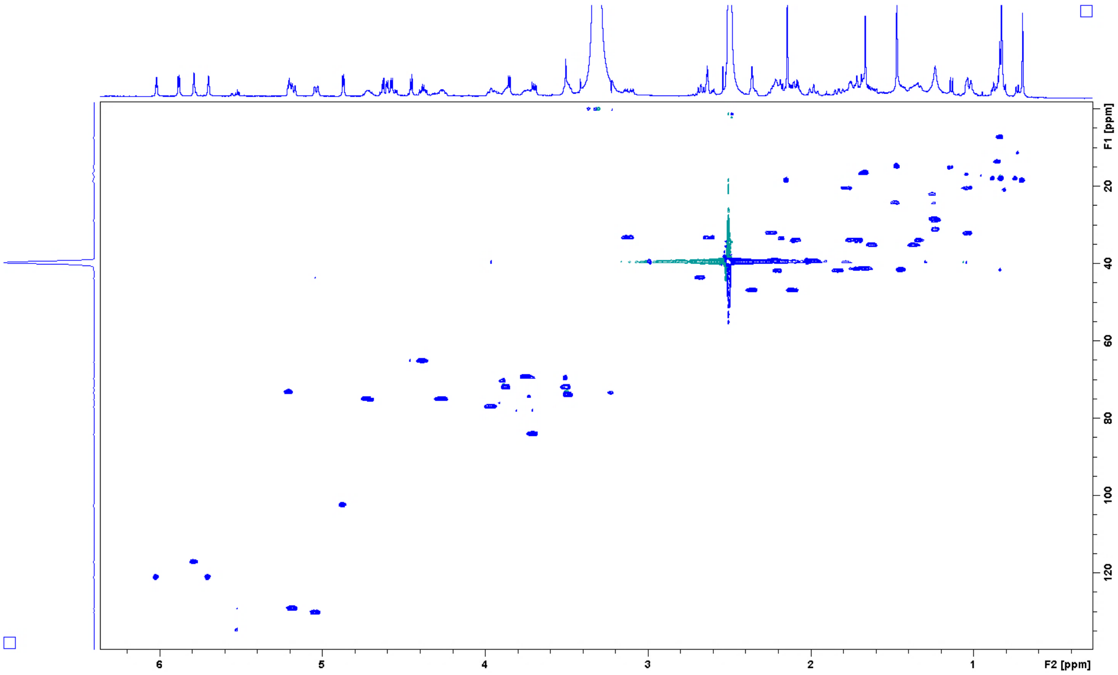

Supplementary Figure 28: HSQC spectrum of gynuellalide in DMSO-*d*$_6$.

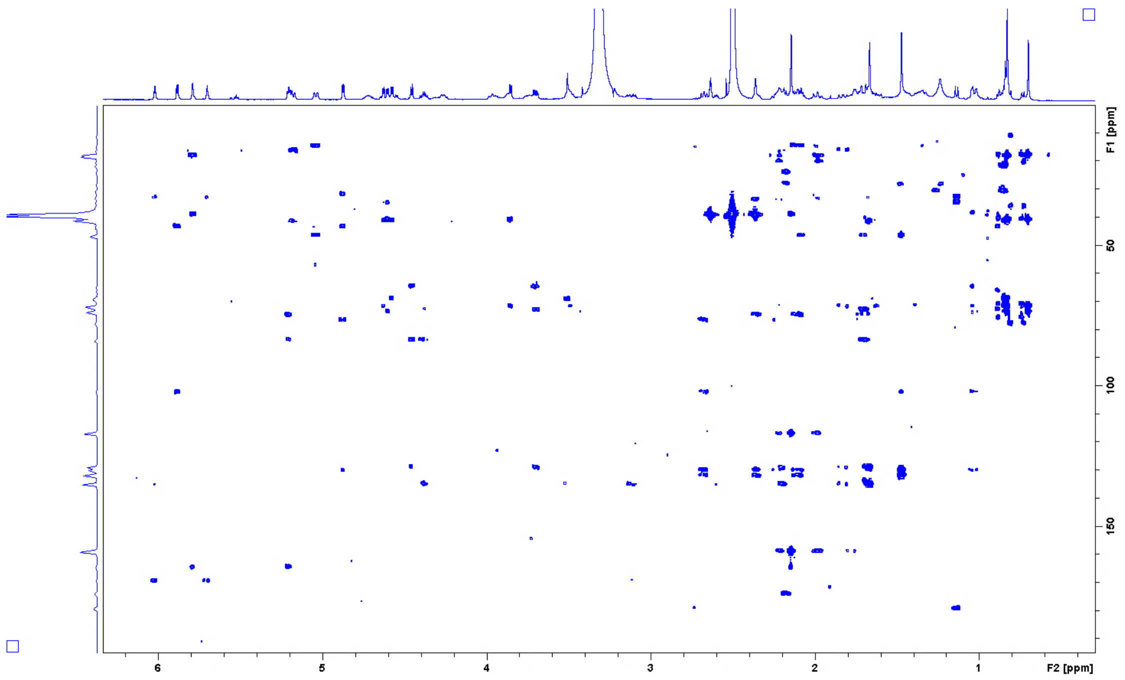

Supplementary Figure 29: HMBC spectrum of gynuellalide in DMSO-*d*6.

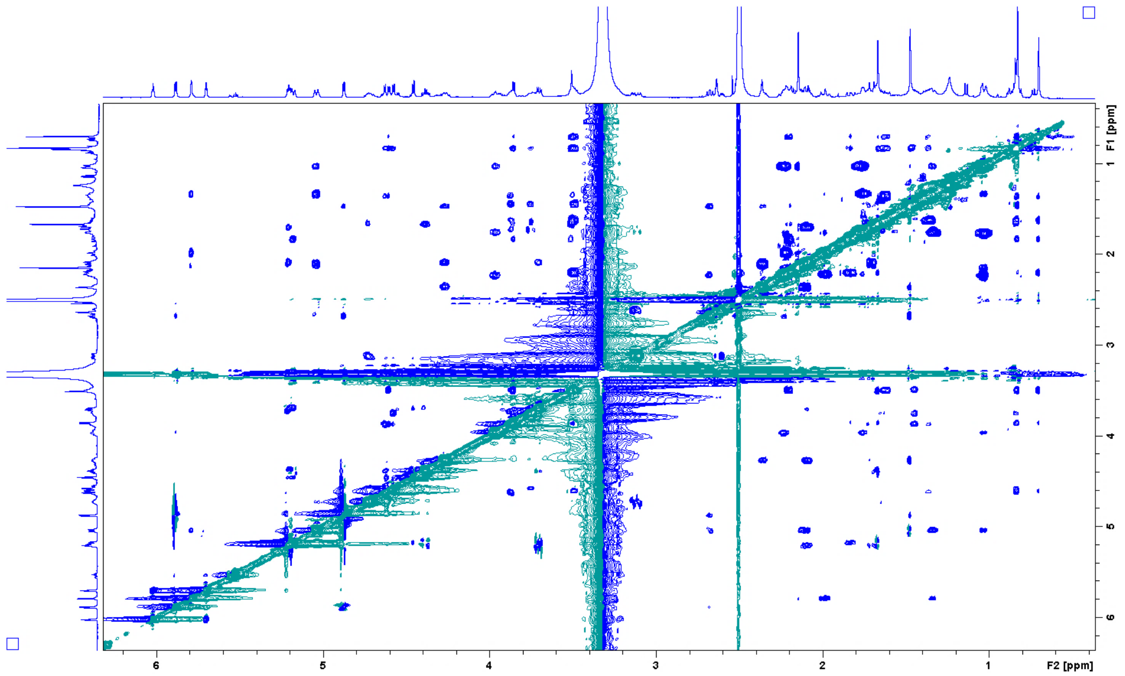

Supplementary Figure 30: ROESY spectrum of gynuellalide in DMSO-*d*6.

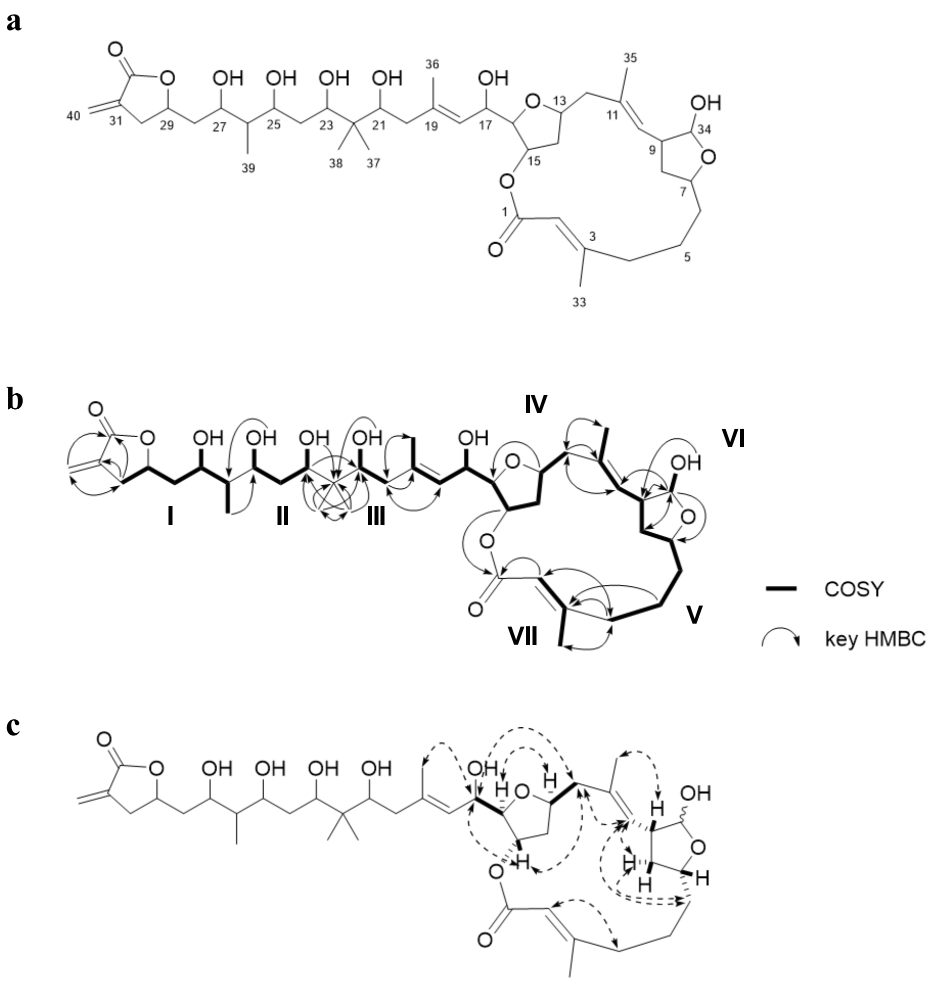

Supplementary Figure 31: Structure elucidation of gynuellalide. (**a**) Structure of gynuellalide. (**b**) COSY and key HMBC correlations of gynuellalide. (**c**) Key NOESY correlations of gynuellalide.

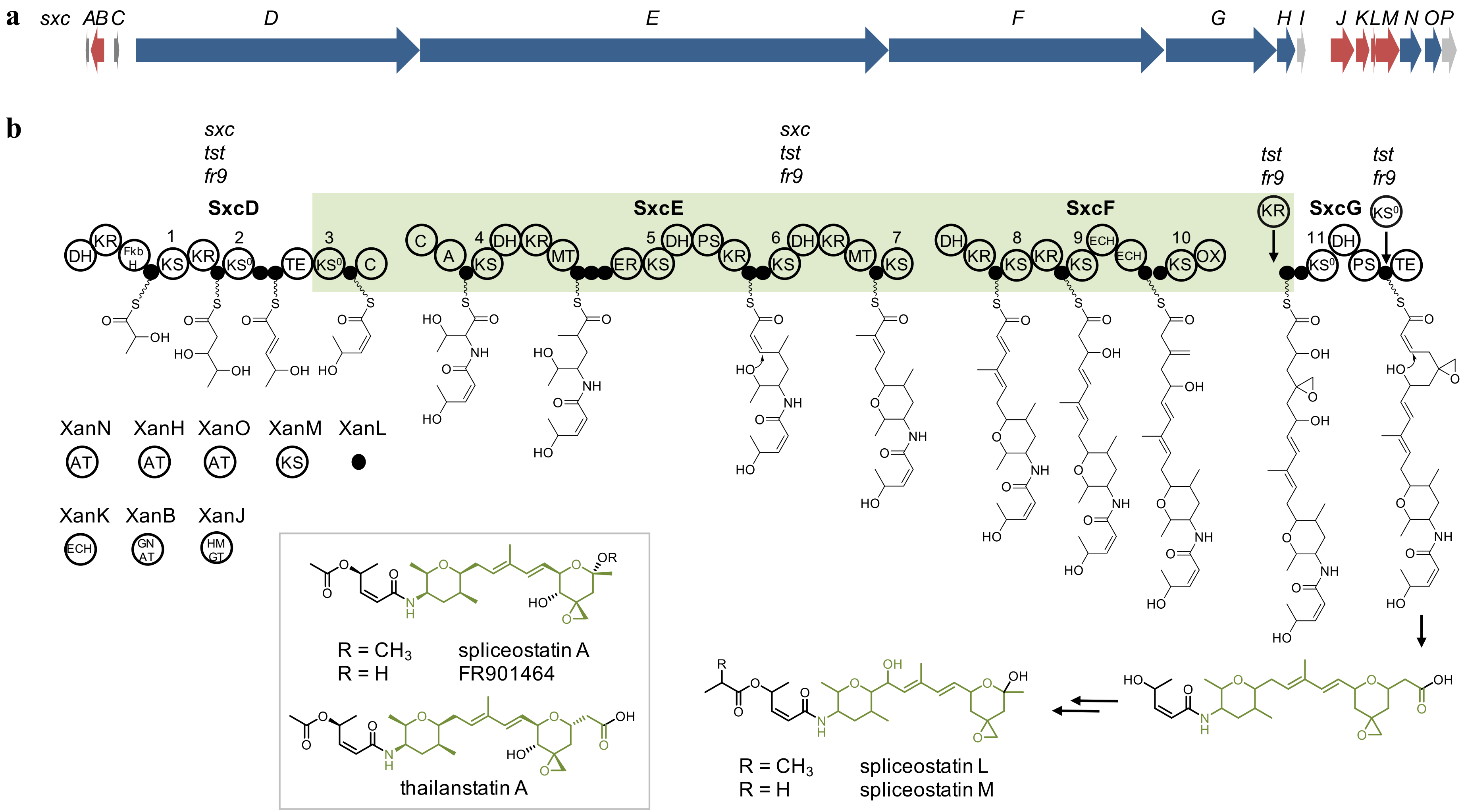

Supplementary Figure 32: Spliceostatin biosynthetic model. (**a**) The spliceostatin BGC. Blue: core PKS genes, red: β-branching cassette, grey: other genes. (**b**) The spliceostatin PKS and model for spliceostatin biosynthesis. The spliceostatin PKS proteins of *X. cannabis* (*sxc*) are shown with bold labels, other labels refer to genes encoding the core PKS of the related thailanstatin (*tst*) and FR901464 (fr9) pathways. Substructures associated with the biosynthesis of the PKS fragment detected by *trans*PACT are shown in green.

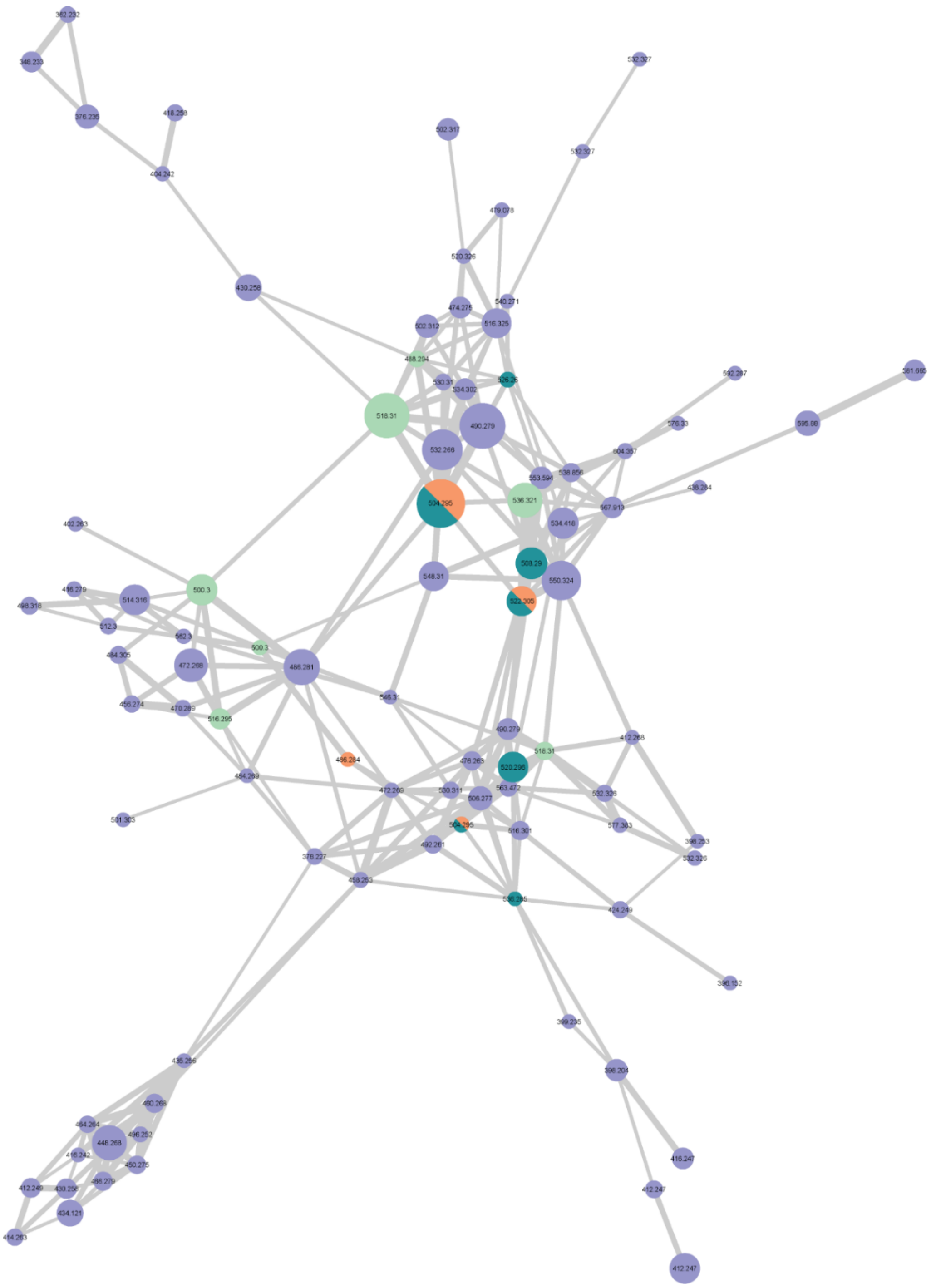

Supplementary Figure 33: Mass-spectral molecular network analysis of extracts from *X. cannabis*. Node size correlates with the number of MS^2 scans for a given ion and can be used as a proxy for the relative abundance. The edge line width indicates the relatedness of two significantly pairwise aligned spectra (cosine 0.65 or higher). Ions corresponding to the isolated compound spliceostatin L (**20**) are colored orange, those that fit adducts of spliceostatin M (**21**) light green. Masses that resemble adducts (proton or sodium) of compounds reported in the literature are colored turquois.

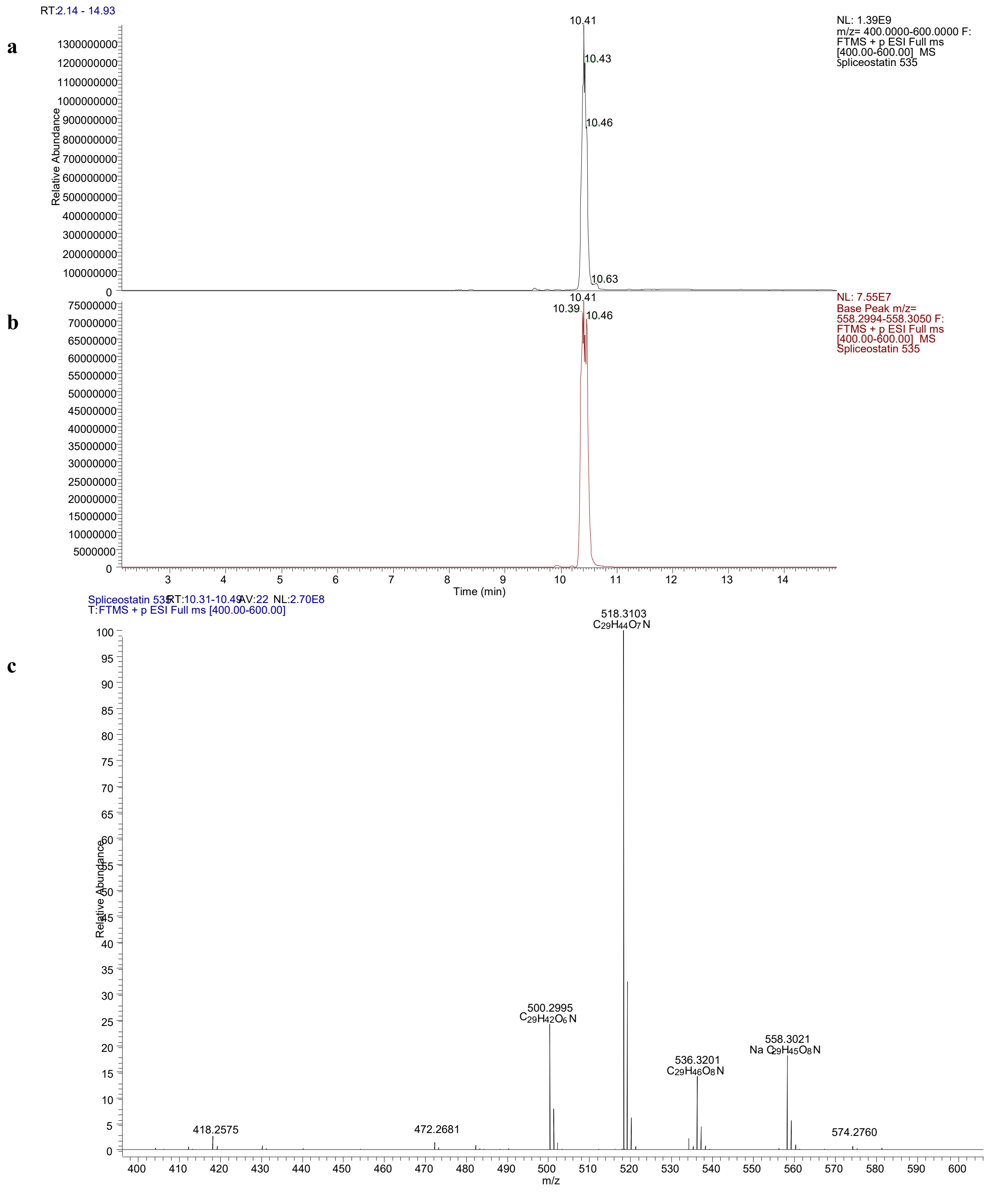

Supplementary Figure 34: UHPLC-HRMS data of purified spliceostatin L (20) from *Xanthomonas cannab*is. (**a**) Total ion chromatogram; (**b**) Extracted ion chromatogram (m/z 558.2994-558.3050); (**c**) Mass spectrum of the peak at 10.41 min (*m/z* 558.3021 $[M+Na]^+$, *m/z* 536.3201 $[M+H]^+$, *m/z* 518.3103 $[M+H-H_2O]^+$, *m/z* 500.2995 $[M+H-2H_2O]^+$).

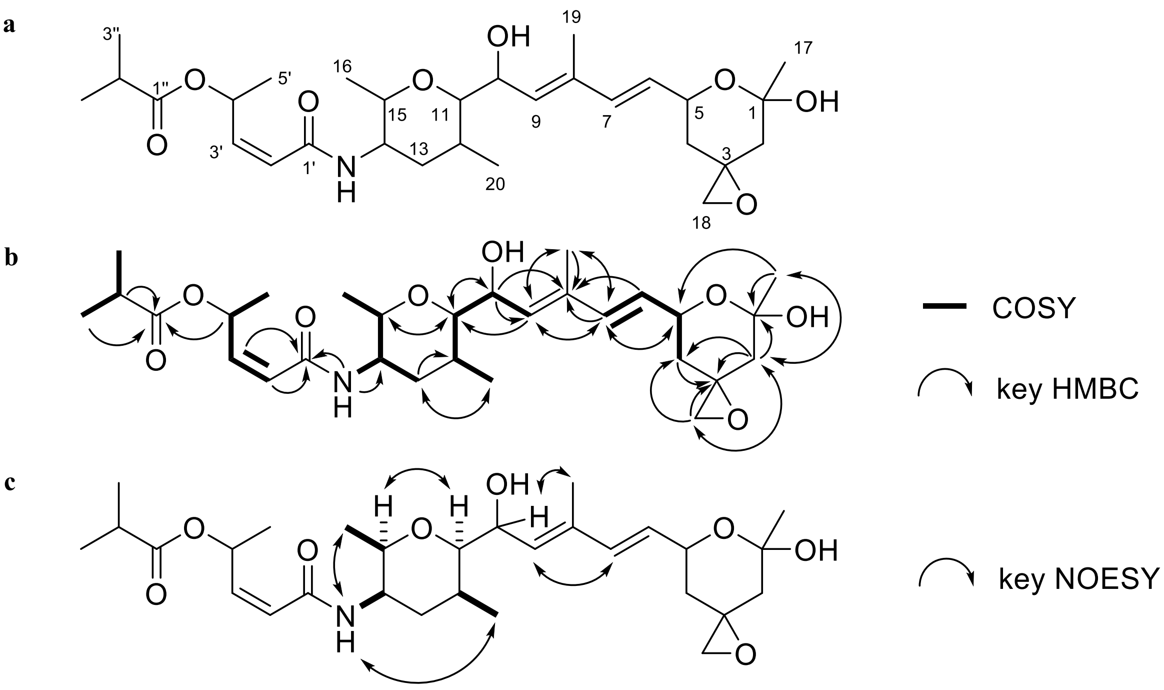

Supplementary Figure 35: Structure elucidation of spliceostatin L (20). (**a**) atom labeling according to Supplementary Table 7, (**b**) Key HMBC and COSY correlations of **20**, (**c**) key NOESY correlations of **20**.

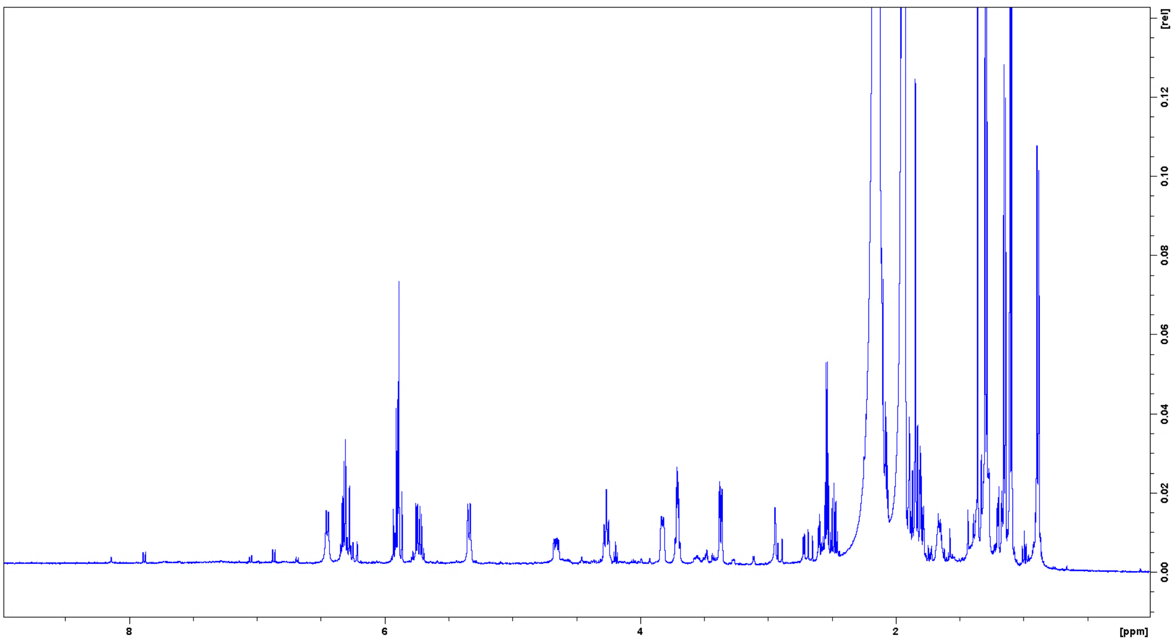

Supplementary Figure 36: ^{1}H NMR spectrum of spliceostatin L (20) from *Xanthomonas cannabis* in acetonitrile-*d*$_3$.

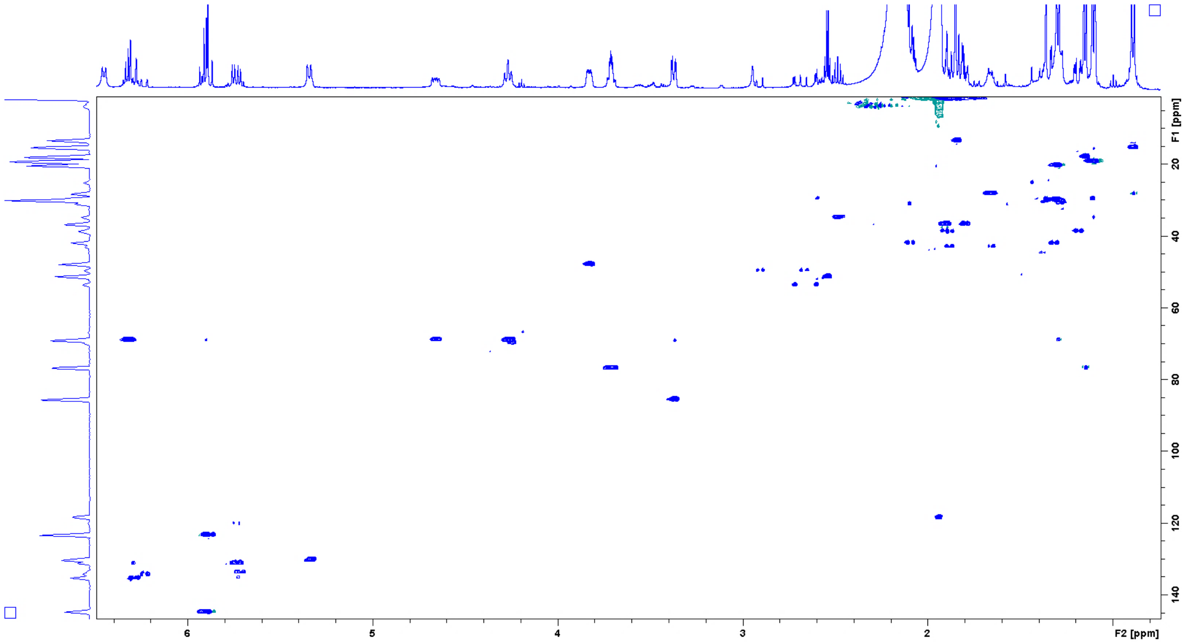

Supplementary Figure 37: HSQC spectrum of spliceostatin L (20) from *Xanthomonas cannabis* in acetonitrile-*d*3.

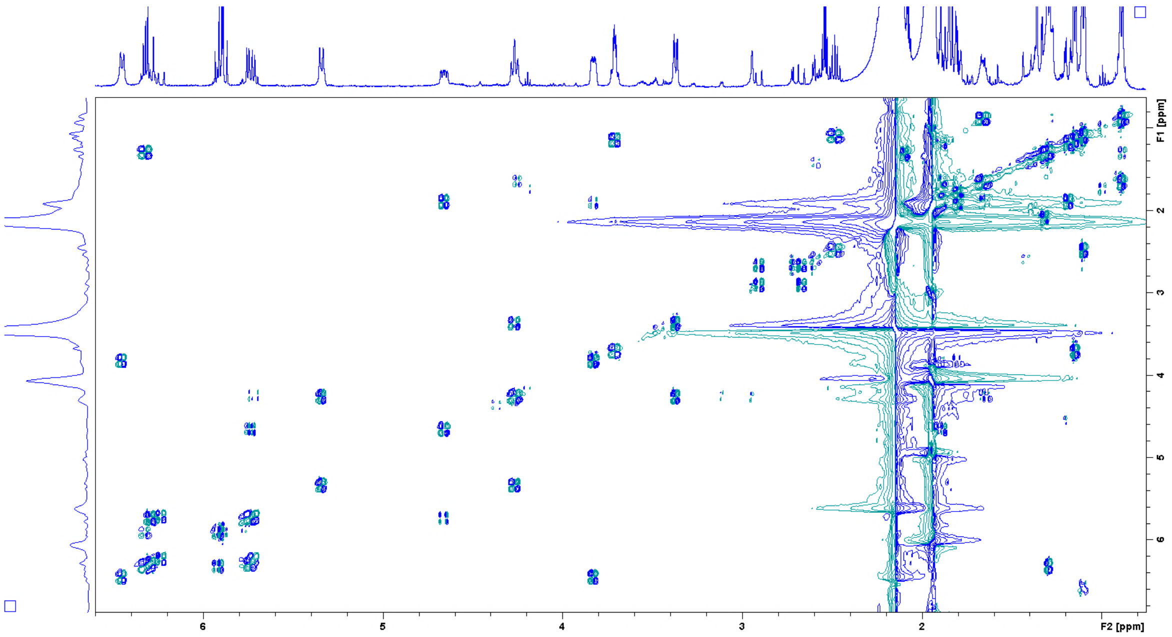

Supplementary Figure 38: COSY spectrum of spliceostatin L (20) from *Xanthomonas cannabis* in acetonitrile-*d*3.

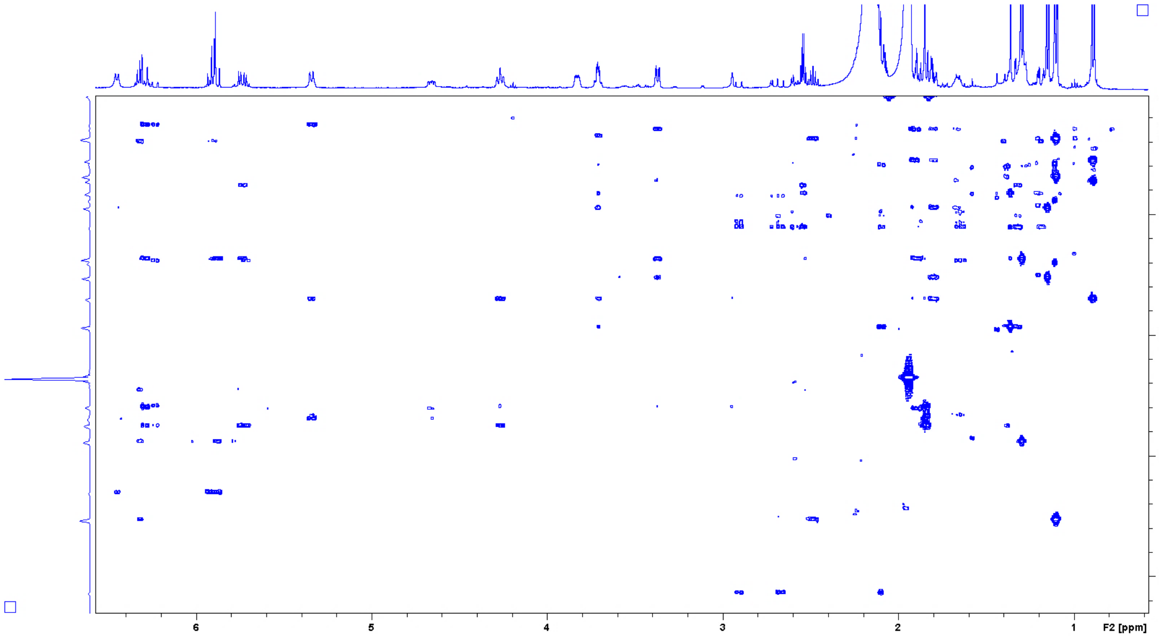

Supplementary Figure 39: HMBC spectrum of spliceostatin L (20) from *Xanthomonas cannabis* in acetonitrile-*d*$_3$.

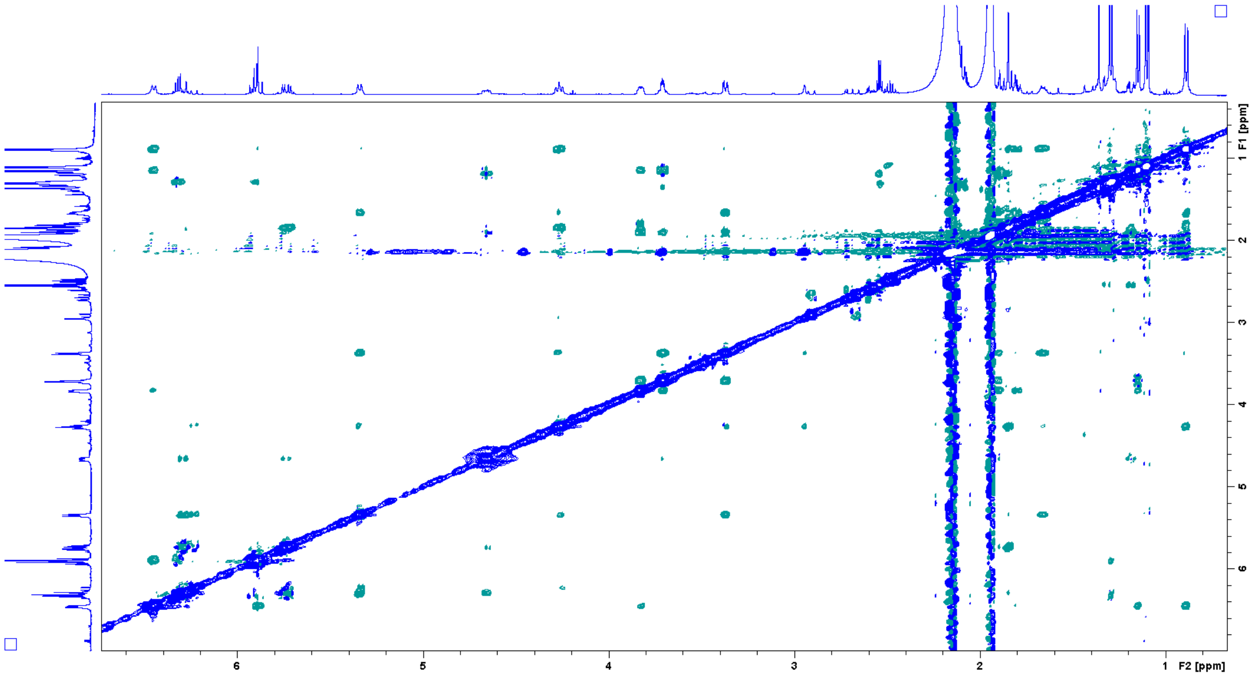

Supplementary Figure 40: NOESY spectrum of spliceostatin L (20) from *X. cannabis* in acetonitrile-*d*$_3$.

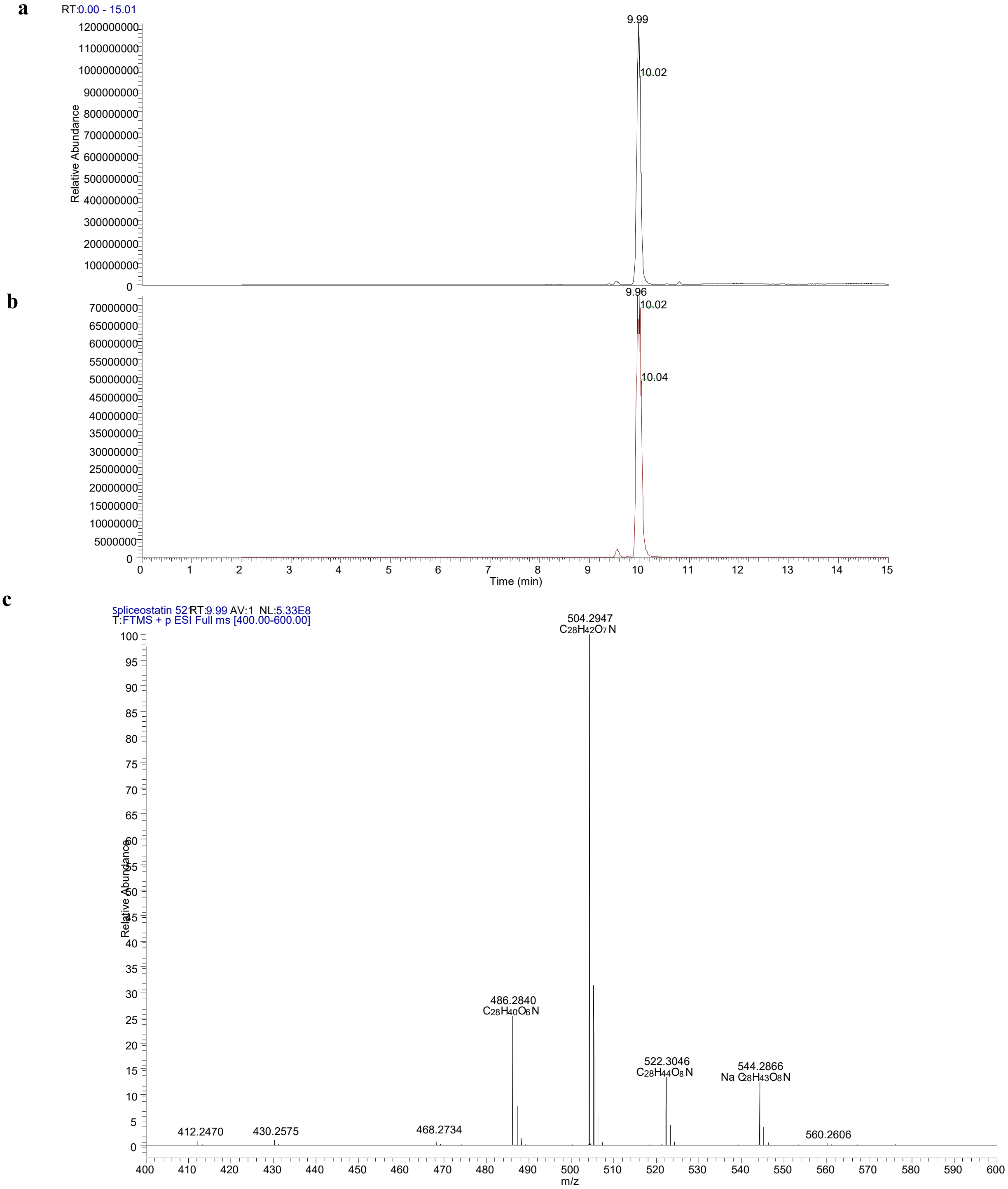

Supplementary Figure 41: UHPLC-HRMS data of purified spliceostatin M (21). (**a**) Total ion chromatogram; (**b**) Extracted ion chromatogram (*m/z* 544.2838-544.2892); (**c**) Mass spectrum of the peak at 9.99 min (*m/z* 544.2866 $[M+Na]^+$, *m/z* 522.3046 $[M+H]^+$, *m/z* 504.2947 $[M+H-H_2O]^+$, *m/z* 486.2840 $[M+H-2H_2O]^+$).

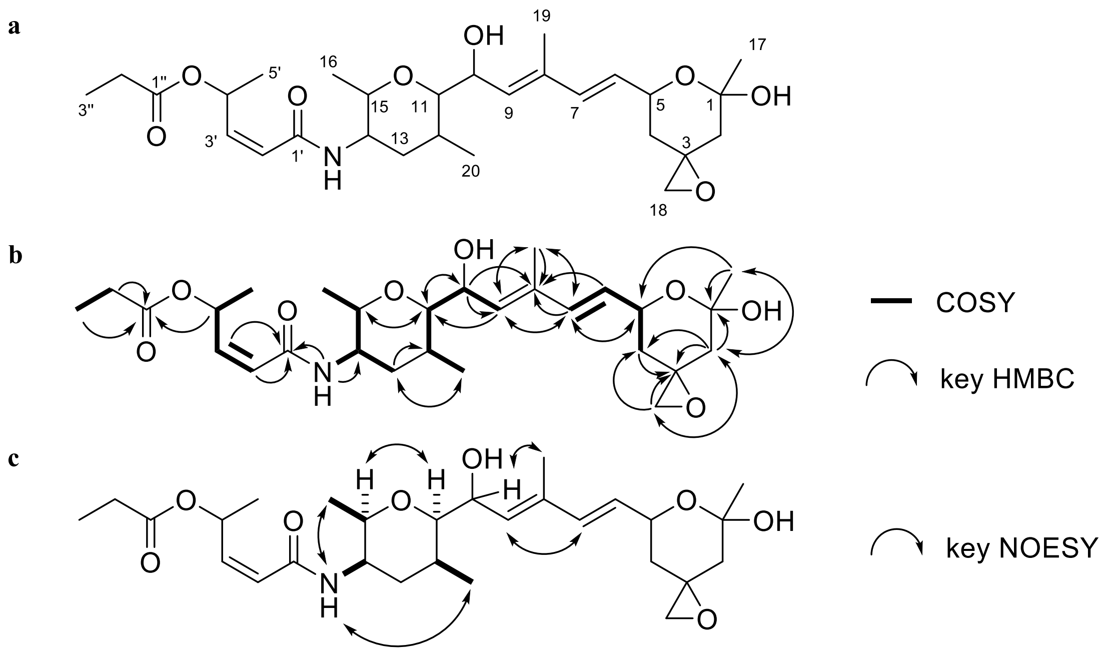

Supplementary Figure 42: Structure elucidation of spliceostatin M (21). (**a**) Atom labeling according to Supplementary Table 8, (**b**) HMBC and COSY key correlations of **21**, (**c**) NOESY key correlations of **21**.

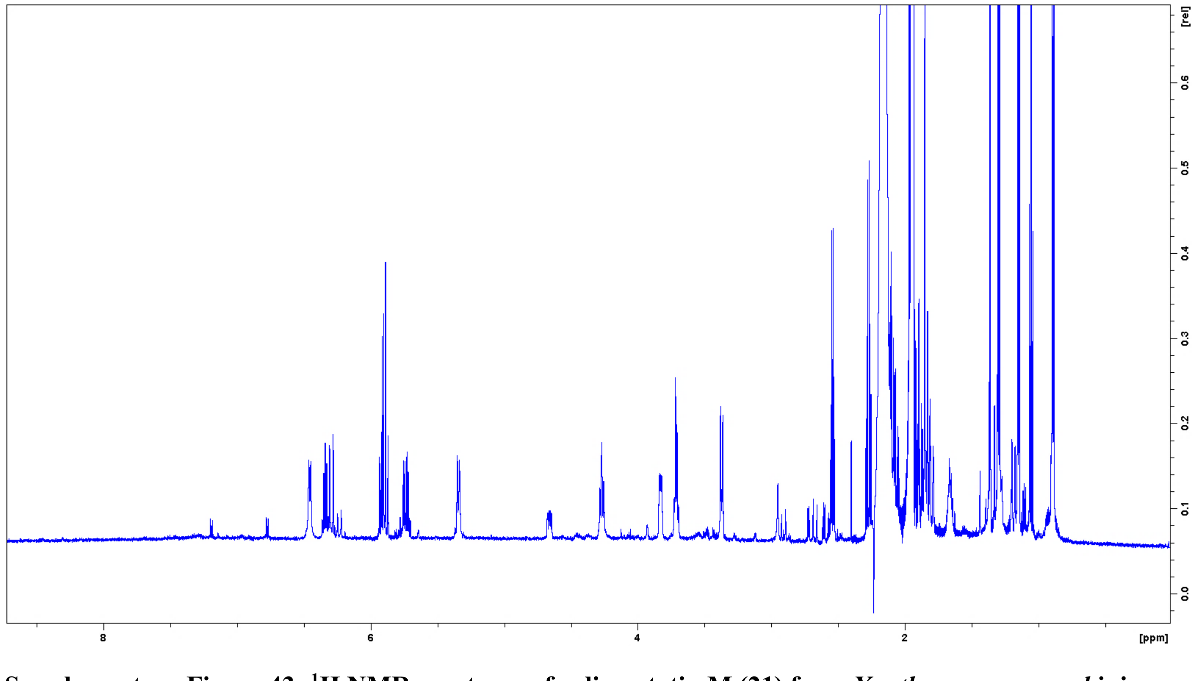

Supplementary Figure 43: ^{1}H NMR spectrum of spliceostatin M (21) from *Xanthomonas cannabis* in acetonitrile-*d*$_{3}$.

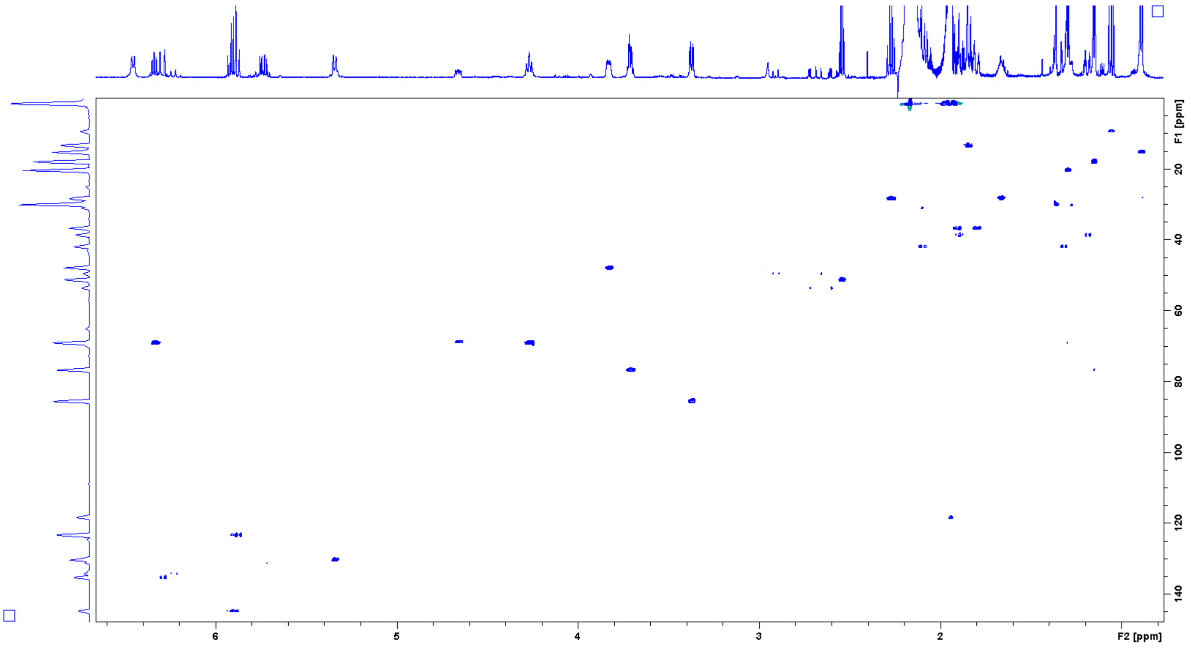

Supplementary Figure 44: HSQC spectrum of spliceostatin M (21) from *Xanthomonas cannabis* in acetonitrile-*d*3.

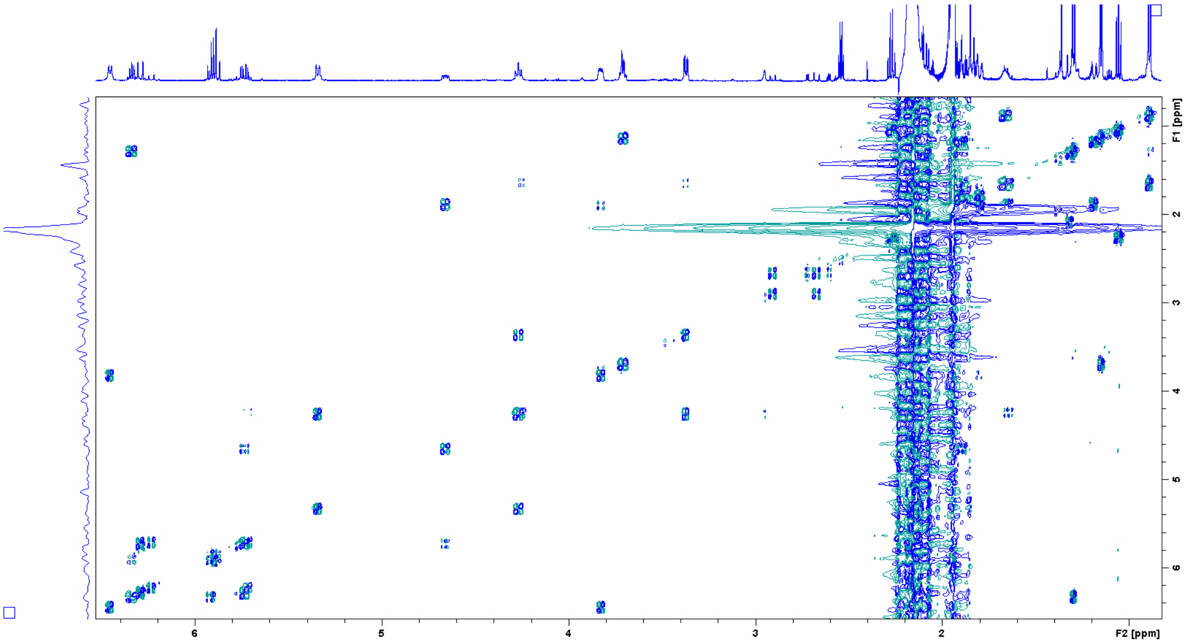

Supplementary Figure 45: COSY spectrum of spliceostatin M (21) from *Xanthomonas cannabis* in acetonitrile-*d*3.

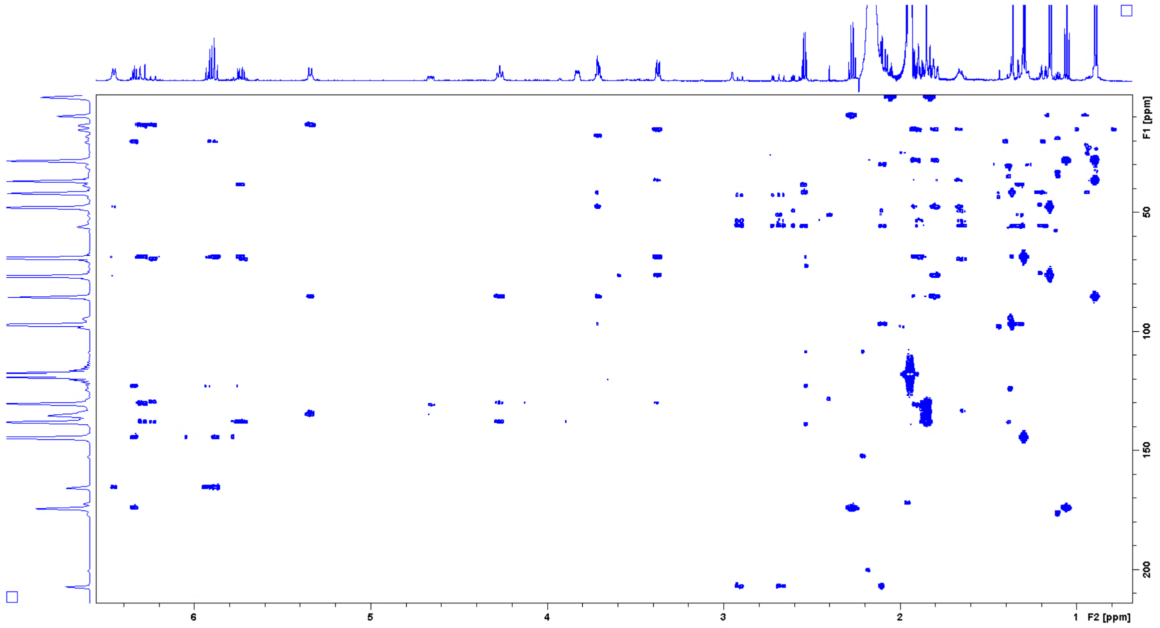

Supplementary Figure 46: HMBC spectrum of spliceostatin M (21) from *Xanthomonas cannabis* in acetonitrile-*d*3.

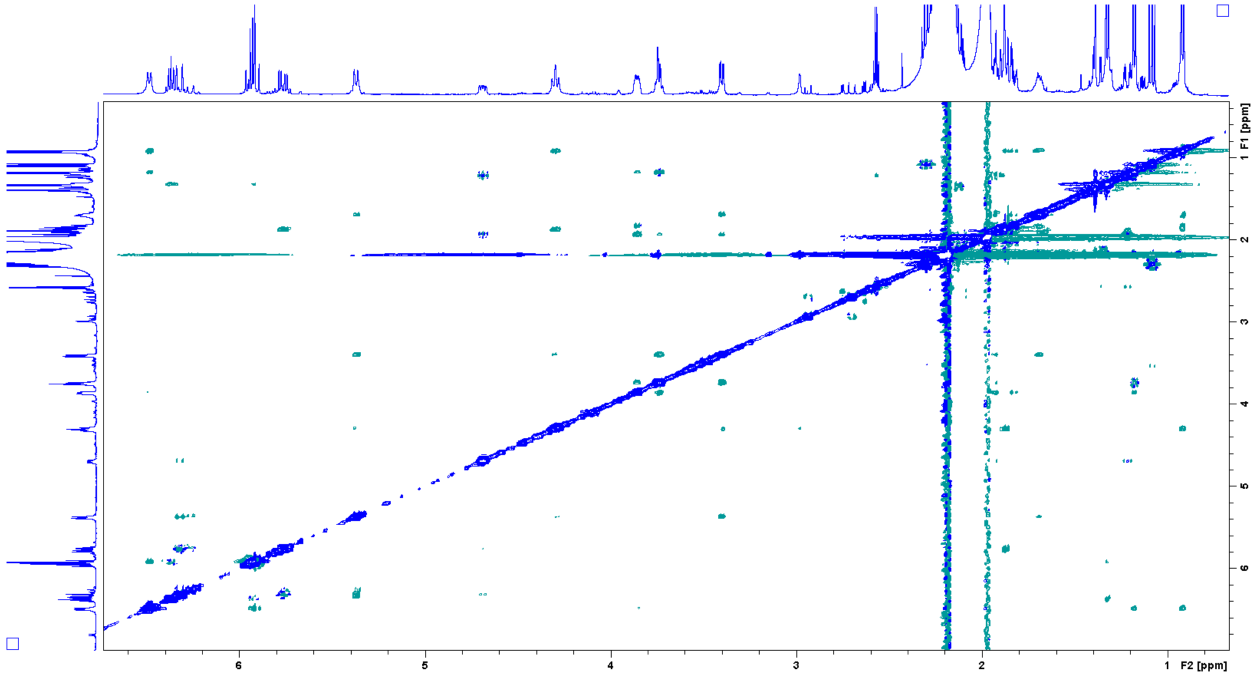

Supplementary Figure 47: NOESY spectrum of spliceostatin M (21) from *X. cannabis* in acetonitrile-*d*3.

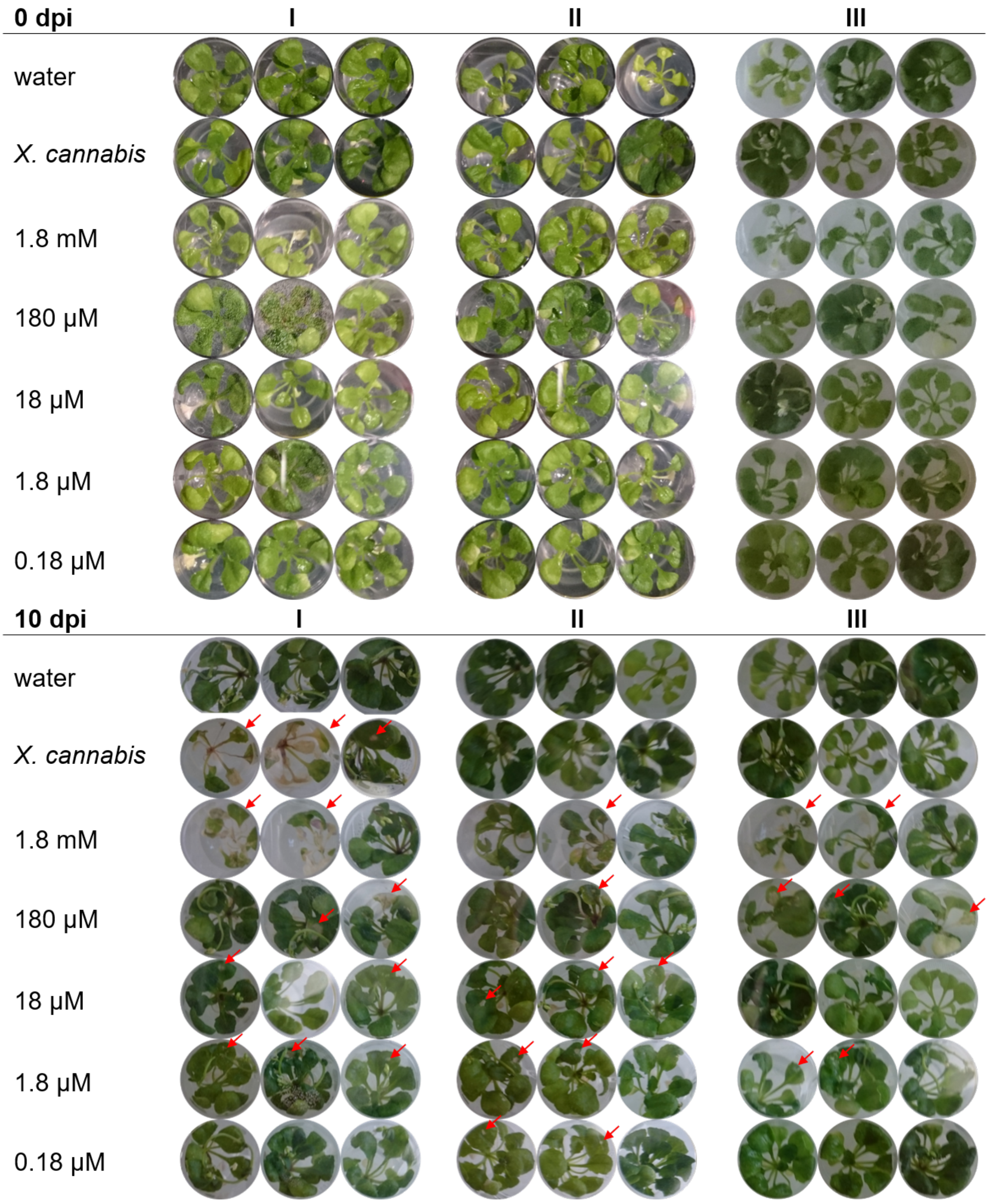

Supplementary Figure 48: Effect of *X. cannabis* and spliceostatin L (20) on *Arabidopsis thaliana*. 1 µL of different concentrations of **20** (from 1.8 mM to 180 nM) were spotted onto one leaf per plant. A cell suspension of *X. cannabis* (OD_{600} = 0.5) served as a positive control and water as a negative control. The experiment was performed in triplicates on three independent 24-well plates (**I-III**). Pictures of the 21 wells used per plate are shown. Top: plants directly post inoculation (0 dpi). Bottom: plants 10 days post inoculation (10 dpi). Red arrows: plants treated with various concentrations of **20** show white lesions on the treated leafs.

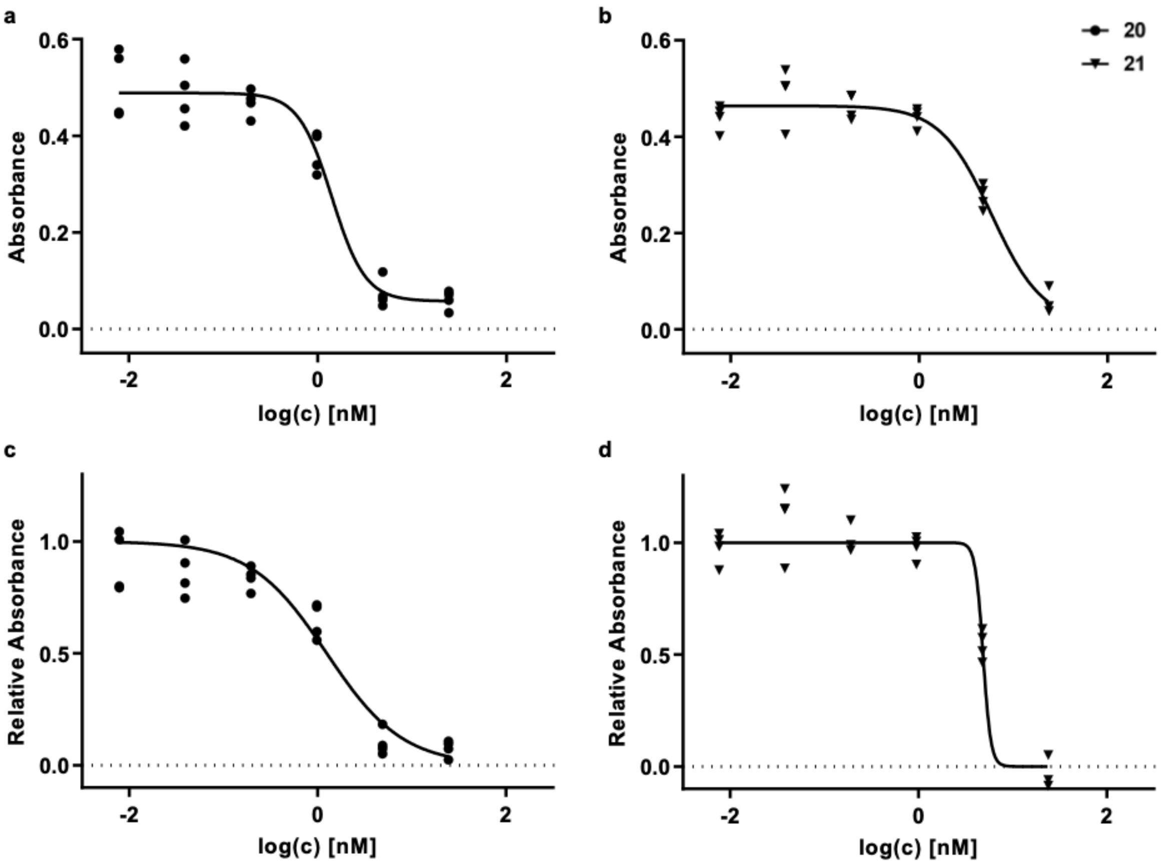

Supplementary Figure 49: HeLa cell assay for the determination of IC_{50} values of spliceostatin L (circle) and spliceostatin M (triangle). Absorbance was measured at 570 nm. A 4-parameter logistic model was used to fit the curve. **a** and **b**, Calculation of the relative IC_{50} values ($IC_{50,\,20}$ = 5.9 nM; $IC_{50,\,21}$ = 1.4 nM). **c** and **d**, calculation of the absolute IC_{50} values ($IC_{50,\,20}$ = 4.9 nM; $IC_{50,\,21}$ = 1.3 nM). Data were normalized by averaging the three lowest absorbance values measured at high spliceostatin concentrations and setting it as the bottom of the curve (bottom constrained to 0.0) and the three negative control values containing the least DMSO and setting it as the top of the curve (top constrained to 1.0). Four biological replicates were measured for each concentration.

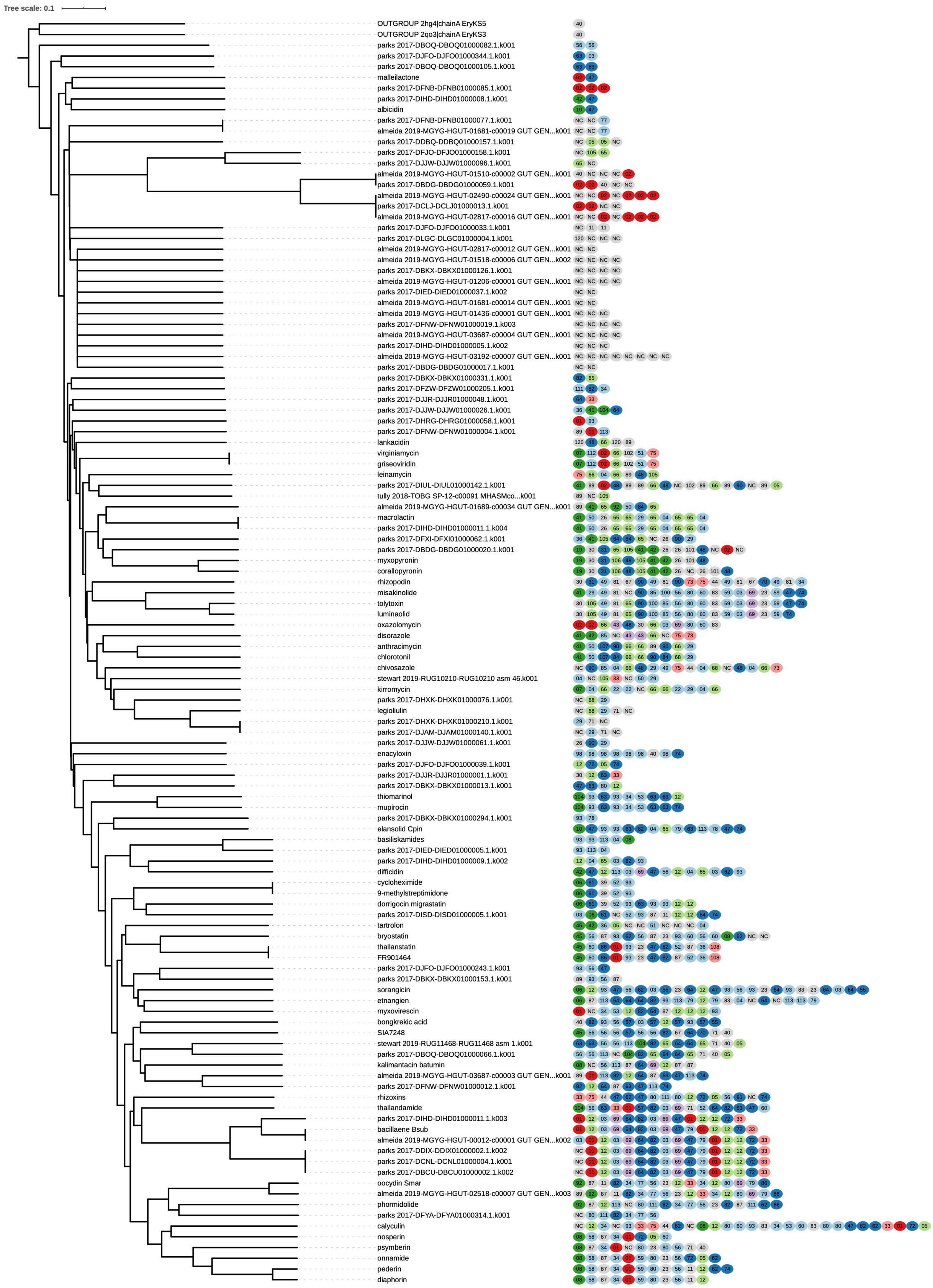

Supplementary Figure 50: Diversity of *trans*-AT PKS assembly lines in metagenome-assembled genomes. *Trans*PACT-generated dendrogram showing the diversity of *trans*-AT PKS assembly lines identified from 20,584 metagenome-assembled genomes. Taxonomic assignment was performed using GTDB-Tk. The DFYA cluster is assigned as an otherwise unspecified Opitutaceae bacterium (could not be assigned in more detail by GTDB-Tk); the DISD cluster is from *Magnetospirillum* sp. 002435715; the DFNW and HGUT-03687 clusters are both assigned to *Paenibacillus polymyxa*; the HGUT-02518 gene cluster, which is related to the oocydin BGC, is

assigned to *Serratia* sp. 001642805; the parks 2017 DIHD-DIHD01000009.1.k002, DIHD-DIHD01000011.1.k003 and DIHD-DIHD01000011.1.k004 gene clusters are from *Bacillus velezensis*; the HGUT-00012, DDIX, DBCU and DCNL ones, all related to the bacillaene BGC, are from *Bacillus subtilis*; the DIUL cluster is from an otherwise unspecified Acetobacteraceae bacterium (could not be assigned in more detail); the DBOQ cluster as well as the RUG11468 cluster are assigned to *Ruminococcus_E bromii_A*; the HGUT-01689 cluster is assigned to *Blautia producta*; the DFXI cluster is from *Anaerocolumna* sp. 002434335; the DBDG clusters are from an otherwise unspecified Lachnospiraceae bacterium (could not be assigned in more detail); the RUG10210 cluster is from *Ruminococcus flavefacien*s_*R*.

Supplementary Tables

Group	Color	Clade number	Clade description
Amino acids		1	amino_acids
Amino acids		2	amino_acids
Amino acids		121	amino_acids
bOH		3	bimod_bOH
bOH		4	bimod_bOH
bOH		22	aMe_bketo/bOH
bOH		29	b_OH/keto
bOH		34	non_elongating_hemiacetal/b_OH
bOH		36	non_elongating_reduced/bOH
bOH		49	non_elongating_b_OH
bOH		50	b_OH
bOH		51	b_OH
bOH		52	b_OH/eDB
bOH		53	mostly_a_OH_b_OH
bOH		56	b_L_OH
bOH		58	a_LMe_b_DOH
bOH		59	b_DOH
bOH		60	non_elongating_b_OH
bOH		77	aOH_bOH
bOH		78	b_L_OH
bOH		79	non_elongating_b_L_OH
bOH		80	aMe_b_L_OH
bOH		85	aMe_bOH
bOH		93	aMe_red/bOH/bketo
bOH		98	b_OH/eDB(enacyloxins)
bOH		99	aMe_bOH
bOH		100	aMe_b_L_OH
bOH		111	b_DOH
bOH		112	b_DOH
bOH		113	a_Me_b_OH
bOH		115	b_DOH
bOH		116	b_LOH
bOH		117	b_OH
bOH		122	b_DOH
bOH		124	b_DOH
bOH		128	b_OH
Double bonds		5	shifted_double_bonds
Double bonds		12	reduced/shifted_double_bonds
Double bonds		65	double_bonds
Double bonds		66	double_bonds
Double bonds		68	double_bonds
Double bonds		105	reduced/shifted double_bonds
Double bonds		106	shifted_double_bonds
eDB		31	eDB
eDB		47	aMe_eDB
eDB		48	aMe_eDB
eDB		55	non_elongating_DB
eDB		57	eDB
eDB		61	non_elongating_DB_before_branching
eDB		62	eDB
eDB		63	mainly_eDB
eDB		64	eDB
eDB		70	eDB
eDB		72	non_elongating_aMeeDB
eDB		74	non_elongating_DB
eDB		82	b_MeeDB
eDB		84	aMe_z_shifted_DB
eDB		86	non_elongating_DB
eDB		90	red_a_Me/shifted DB_a_Me
eDB		97	bMeDB
eDB		107	eDB
eDB		110	non-elongating_zDB
Non-elongating		33	non_elongating
Non-elongating		73	non_elongating
Non-elongating		75	non_elongating_oxazole
Non-elongating		108	non_elongating_pyran/furan
Starter		6	unusual_Starter_AMT
Starter		7	Starter
Starter		8	GNAT_Starter
Starter		10	aromatic_Starter
Starter		19	methoxycarbonyl_Starter
Starter		41	acetyl_Starter
Starter		42	DB/DB_Starter
Starter		45	lactate_Starter
Starter		92	unusual_Starter
Starter		104	acetyl/aromatic Starter
Starter		125	aromatic starter
zDB		43	zDB
zDB		69	zDB
zDB		119	zDB
Other		11	oxygen_insertion
Other		23	pyran/furan
Other		26	reduced
Other		30	glycine
Other		38	mainly reduced
Other		39	vinylogous_chain_branching
Other		40	cis-AT PKS-like
Other		44	oxazole
Other		67	various
Other		71	b_keto
Other		81	b_D_OMe
Other		83	b_OMe
Other		87	exometh/red_bMe
Other		89	b_keto
Other		101	b_Me
Other		102	b_Me
Other		103	aMe_reduced
Other		109	non-elongating_aMe_reduced
Other		114	unknown
Other		120	various
Other		123	non-canonical pyran
Other		126	a_oxygenation
Other		127	beet
Other		NC	not_conserved

Supplementary Table 1: Color code and clade assignment used throughout this study.

Supplementary Table 2: Secimide biosynthetic genes, protein size, proposed function and closest homologs

protein	amino acids	proposed function	sequence similarity (protein, origin)	identity	accession number
**SecA**	337	α-ketoglutarate dependent dioxygenase	*Gemmatimonadetes bacterium*, MBJ67707.1	29%	AAY39347.1
**SecB**	81	ACP	*Desulfofaba hansenii* WP_100393531.1	49%	AAY39346.1
**SecC**	658	asparagine synthase	*Robbsia andropogonis* WP_024905973.1	51%	AAY39345.1
**SecD**	3231	PKS (KS, DH, KR, ACP, KS, B, ACP, KS)	*Burkholderia gladioli*, WP_103692967.1	44%	AAY39344.1
**SecE**	2393	PKS (KR, ACP, KS, ACP, KS, DH)	*Burkholderia gladioli*, WP_146128462.1	39%	AAY39343.1
**SecF**	2260	PKS (KR, MT, ACP, KS, KR, ACP)	*Bacillus halotolerans*; WP_059292739.1	39%	AAY39342.1
**SecG**	1055	PKS (AH, AT, ER)	*Brevibacillus* sp. NRRL NRS-603, WP_106782766.1	32%	AAY39341.1
**SecH**	311	3-oxoacyl-ACP synthase	*Leptospira alexanderi*, WP_078124957.1	25%	AAY39340.1
**SecI**	204	LysE type translocator	*Klebsiella pneumonia*, WP_131729069.1	38%	AAY39339.1
**SecJ**	518	phosphotransferase	*Pseudomonas fluorescens*, WP_003212136.1	94%	AAY39338.1
**SecK**	311	carboxyl methyltransferase (SAM dependent)	*Oxalobacteraceae bacterium*, HCN87942.1	59%	AAY39337.1
**SecL**	65	hypothetical protein	*Pseudomonas* sp. GM60, WP_085989737.1	68%	AAY39336.1
**SecM**	85	hypothetical protein	*Pseudomonas* sp. 286, WP_137984090.1	98%	AAY39335.1
**SecN**	380	endonuclease	*Pseudomonas* sp. 286, WP_137984089.1	97%	AAY39334.1
**SecO**	220	multi-drug efflux protein	*Pseudomonas* sp. 286, WP_122530064.1	99%	AAY39333.1
**SecP**	234	DUF 2076	*Pseudomonas* sp. ICMP 10191, WP_058417062.1	99%	AAY39332.1
**SecQ**	158	nuclease	*Pseudomonas* sp. NP10-3, WP_111522202.1	99%	AAY39331.1
**SecR**	204	DNA polymerase III	*Pseudomonas congelans*,	99%	AAY39330.1

			WP_096131746.1		
**SecS**	155	AsnC transcriptional regulator	*Pseudomonas* sp. 286, WP_122585766.1	90%	AAY39329.1
**SecT**	304	multidrug transporter	*Pseudomonas congelans*, WP_096131744.1	98%	AAY39328.1
**SecU**	442	DEAD/DEAH box helicase	*Pseudomonas congelans*, KPW85797.1	99%	AAY39327.1
**SecV**	424	NAD/FAD dependent dehydrogenase	*Pseudomonas congelans*, KPW85777.1	97%	AAY39326.1
**SecW**	305	histone deacetylase	*Pseudomonas congelans*, WP_054993128.1	98%	AAY39325.1
**SecX**	189	acetyltransferase (GNAT family)	*Pseudomonas congelans*, WP_096125255.1	97%	AAY39324.1
**SecY**	289	TE	*Pseudomonas congelans*, WP_032615532.1	98%	AAY39323.1

Supplementary Table 3: NMR chemical shifts of secimide and MTPA esters in $CDCl_3$

	secimide		secimide (*R*)-MTPA ester		secimide (*S*)-MTPA ester	
No.	δ_C	δ_H, mult.	δ_C	δ_H, mult.	δ_C	δ_H, mult.
1	41.0	1.41 (ddd, 2.7, 8.8, 14.1)	39.1	1.60 m	39.3	1.69 m
		1.68 (ddd, 4.9, 10.4, 14.1)		1.74 m		1.83 m
2	65.1	4.18 m	70.4	5.56 m	70.8	5.57 m
3	44.2	2.76 m	41.5	2.90 (dd, 6.1, 6.1)	41.3	2.84 m
		2.91 (dd, 2.1, 17.6)		3.21 (ddd, 3.7, 6.1, 17.1)		3.19 (ddd, 2.8, 6.0, 17.2)
4	201.7		197.2		197.0	
5	138.0		138.1		138.1	
6	141.3	6.65 (dd, 1.1, 9.2)	141.0	6.60 m	140.9	6.56 m
7	39.8	3.55 (qd, 7.2 , 9.2)	39.7	3.54 ovlp	39.7	3.53 ovlp
8	173.9		173.9		173.9	
9	11.5	1.83 (d, 1.1)	11.5	1.81 brs	11.4	1.78 brs
10	17.4	1.35 (d, 7.2)	17.4	1.34 (d, 7.0)	17.4	1.33 (d, 7.1)
11	52.5	3.73 s	52.4	3.73 s	52.4	3.73 s
1'	27.5	2.52 m	26.7	1.85 m	27.1	2.03 m
2'	37.2	2.34 (dd, 10.9, 10.9)	36.9	2.19 m	37.1	2.31 m
		2.81 m		2.71 m		2.80 m
3'	171.8		171.0		171.0	
5'	171.8		171.0		171.0	
6'	38.6	2.37 (dd, 10.7, 10.7)	38.1	2.16 m	38.2	2.24 m
		2.78 m		2.52 m		2.62 m

Supplementary Table 4: Gynuellalide biosynthetic genes, protein size, proposed function and closest homologs

protein	amino acids	proposed function	sequence similarity (protein, origin)	identity (%)	accession number
**GynA**	252	enoyl-CoA hydratase	*Leptolyngbya* sp. ISBN3-Nov-94-8 AMH40437.1	67%	WP_044617468.1
**GynB**	261	enoyl-CoA hydratase	*Leptolyngbya* sp. PCC 7375 WP_006512938.1	69%	WP_044617469.1
**GynC**	420	HMG CoA-synthase	*Leptolyngbya* sp. ISBN3-Nov-94-8 AMH40430.1	78%	WP_044617470.1
**GynD**	6819	PKS (DH-FkbM-FkbH-ACP-KS-CR-CR-ACP-ACP-ACP-KS-KR-ACP-KS-KR-MT-ACP-KS-KR)	*Dickeya dianthicola* WP_029729610.1	46%	WP_044617471.1
**GynE**	5532	PKS (ACP-KS-KR-MT-ACP-KS-KR-ACP-KS-ACP-ACP-ACP-ACP-KS-KR-ACP)	*Leptolyngbya* sp. PCC 7375 EKU96423.1	50%	WP_144407643.1
**GynF**	380	flavin-dependent oxidoreductase	*Dickeya dianthicola* WP_024106524.1	59%	WP_044617473.1
**GynG**	4258	PKS (KS-ACP-KS-KR-ACP-KS-DH-PS-KR-ACP-KS-ACP)	*Leptolyngbya* sp. ISBN3-Nov-94-8 AMH40423.1	47%	WP_052830282.1
**GynH**	6350	PKS (ACP-KS-CR-ACP-KS-KR-ACP-KS-DH-KR-ACP-ACP-KS-ACP-ACP-KS-ACP-C)	*Leptolyngbya sp. ISBN3-Nov-94-8* AMH40443.1	47%	WP_044617474.1
**GynI**	493	dioxygenase	*Zhongshania aliphaticivorans* WP_008246983.1	69%	WP_044617475.1
**GynJ**	809	peptidase	*Oceanicoccus sagamiensis* WP_085757908.1	52%	WP_082070718.1
**GynK**	412	cytochrome P450	*Parvibaculum* sp. MAN62859.1	37%	WP_044617477.1
**GynL**	79	ACP	*Alteromonadales bacterium* BS08 WP_075185092.1	56%	WP_044617478.1
**GynM**	409	KS	*Tahibacter aquaticus* WP_133818593.1	59%	WP_044617479.1
**GynN**	774	metalloprotease	*Thalassolituus* sp. C2-1 WP_145466512.1	66%	WP_044617480.1
**GynO**	178	hypothetical protein	*Reinekea marinisedimentorum* WP_132701842.1	30%	WP_044617481.1
**GynP**	339	FruR transcriptional regulator	*Proteobacteria bacterium* 228 WP_146074273.1	70%	WP_044617482.1

Supplementary Table 5: NMR chemical shifts of gynuellalide in DMSO-d_6

No.	δ_C	δ_H, mult.	No.	δ_C	δ_H, mult.
**1**	164.8		22	41.3	
**2**	117.1	5.79 brs	23	73.9	3.49 m
**3**	159.2		24	35.2	1.37 m
**4**	39.4	1.98 m			1.62 m
		2.22 ovlp	25	72.0	3.87 m
**5**	20.5	1.03 ovlp	26	41.6	1.45 m
		1.78 m	27	69.3	3.75 m
**6**	34.0	1.33 m	28	41.3	1.67 ovlp
		1.73 ovlp			1.72 ovlp
**7**	77.0	3.96 m	29	75.0	4.72 m
**8**	32.1	1.03 ovlp	30	33.3	2.62 ovlp
		2.24 m			3.12 (dddd, 2.5, 2.5, 7.6, 17.2)
**9**	43.6	2.67 (dd, 8.0, 9.0)	31	135.2	
**10**	130.1	5.04 (d, 9.0)	32	169.7	
**11**	132.1		33	18.4	2.14 brs
**12**	46.9	2.11 ovlp	34	102.4	4.87 (d, 3.9)
		2.36 ovlp	35	14.8	1.47 brs
**13**	75.0	4.72 m	36	16.5	1.67 brs
**14**	34.0	1.70 ovlp	37	18.5	0.70 s
		2.09 ovlp	38	18.0	0.83 s
**15**	73.2	5.20 (dd, 4.2, 4.2)	39	7.2	0.84 ovlp
**16**	84.0	3.70 (dd, 4.2, 8.8)	40	121.0	5.70 (brdd, 2.2, 2.2)
**17**	65.2	4.38 (ddd, 4.7, 8.8, 8.8)			6.02 (brdd, 2.7, 2.7)
**18**	129.1	5.17 (d, 8.8)	17-OH		4.45 (d, 4.7)
**19**	135.2		21-OH		3.85 (d, 4.9)
**20**	41.8	1.83 (dd, 10.3, 13.5)	23-OH		4.60 (d, 4.6)
		2.21 ovlp	25-OH		4.63 (d, 3.7)
**21**	72.0	3.51 ovlp	27-OH		4.57 (d, 4.8)
			34-OH		5.88 (d, 3.9)

Supplementary Table 6: Spliceostatin BGC from *Xanthomonas cannabis*. The BGC is split into three contigs (GenBank: JSZF01000xyz.1, the last three digits indicate the contig number, see rightmost column). The full-length, concatenated BGC sequence is accessible under accession number BK010647.

protein	amino acids	proposed function	sequence similarity (protein, origin)	identity (%)	accession number	contig (xyz)
**SxcA**	57	transposase	*Xanthomonas vasicola* pv. *zeae* HHZ25049.1	100%	KHL53185.1	085
**SxcB**	227	GNAT family N-acetyl transferase	*Agrobacterium tumefaciens* WP_012650002.1	49%	WP_081419623.1	085
**SxcC**	88	transposase	*Xanthomonas gardneri* WP_003474297.1	99%	KHL53184.1	085
**SxcD**	5.008	PKS (DH, KR, FkbH, ACP, KS, KR, ACP, KS, ACP, ACP, TE, KS, ACP, C)	*Burkholderia thailandensis* MSMB43 EIP85579.1	69%	KHL53186.1 KHL53337.1	085 & 096
**SxcE**	8.224	PKS (C, A, ACP, KS, DH, KR, MT, ACP, ACP, ACP, ER, KS, DH, PS, KR, ACP, ACP, KS, DH, KR, MT, ACP, KS)	*Burkholderia* sp. 2002721687 WP_006028556.1	71%	KHL53336.1	096
**SxcF**	4.844	PKS (DH, KR, ACP, KS, KR, ACP, KS, ECH, ECH, ACP, ACP, KS, OX)	*Burkholderia* sp. MSMB1589WGS WP_063534551.1	72%	KHL53335.1	096
**SxcG**	1.964	PKS (ACP, ACP, KS, DH, PS, ACP, TE)	*Pseudomonas* sp. 2663 ADH01490.1	64%	KHL53334.1 KHL53751.1	096 & 100
**SxcH**	305	AT	*Burkholderia* sp. MSMB1589WGS WP_063534553.1	77%	WP_052210040.1	100
**SxcI**	145	hypothetical protein	*Limnobacter* sp. NLD69161.1	42%	WP_158002175.1	100
**SxcJ**	419	3-hydroxy-3-methylglutaryl-ACP synthase	*Burkholderia thailandensis* ATF32179.1	88%	KHL53752.1	100
**SxcK**	261	enoyl-CoA hydratase	*Burkholderia* sp. BDU18 WP_060822450.1	88%	KHL53753.1	100
**SxcL**	79	ACP	*Burkholderia* sp. BDU18 WP_060822451.1	72%	KHL53754.1	100
**SxcM**	426	KS	*Burkholderia* sp. BDU18 WP_060822452.1	77%	KHL53755.1	100
**SxcN**	377	AT	*Burkholderia* sp. BDU18 WP_082754672.1		KHL53756.1	100
**SxcO**	325	AT	*Burkholderia humptydooensis* KST72253.1	68%	KHL53757.1	100

**SxcP**	238	4'-phosphopantetheinyl transferase	*Pseudomonas acidophila* WP_096716368.1	51%	WP_158002174.1	100

Supplementary Table 7: NMR chemical shifts of 20 in acetonitrile-d_3

No.	δ_H, mult, (J in Hz)	δ_C	COSY	HMBC
**1**	—	97.2	—	—
**2**	1.32; m, 2.09; m	41.8	—	1.32: 1, 3, 4, 18; 2.09: 1, 3, 17, 18
**3**	—	55.9	—	—
**4**	1.18; m, 1.88; m	38.6	5	1.18: 2, 3; 1.88: 5
**5**	4.66; dddd (1.1, 2.4, 6.3, 11.8)	68.8	4, 6	6, 7
**6**	5.73; dd (6.2, 15.8)	131.0	5, 7, weak 10	4, 5, 8
**7**	6.29; d (15.8)	135.3	6	5, 8, 9, 19
**8**	—	138.1	—	—
**9**	5.34; d (9.4)	130.3	10	7, 11, 19
**10**	4.27; m	69.0	9, 11	8, 9, 11,
**11**	3.37; dd (2.5, 8.5)	85.6	10, 12	10, 13, 15, 20
**12**	1.66 ; m	28.2	20	10, 13, 14, 20
**13**	1.80, 1.90; overlap	36.6	—	11, 12, 14, 15, 20
**14**	3.83; dddd (2.1, 2.1 , 4.3, 8.7)	47.8	NH, 13,15	—
**15**	3.71; dq (2.3, 6.5) overlap	76.7	16	11, 14, 16
**16**	1.15; d (6.6)	17.9	15	14, 15
**17**	1.36; s	30.0	—	1, 2, 3, 5
**18**	2.53; d (4.7); 2.55; d (4.7)	51.2	—	2, 3, 4
**19**	1.85; d (1.3)	13.3	9	7, 8, 9
**20**	0.89; d (7.3)	15.3	12	11, 12, 13
**1'**	—	165.8	—	—
**2'**	5.88; d (11.7)	123.3	3'	1', 3', 4', 5'
**3'**	5.91; dd (6.6, 11.7)	144.7	2', 4',	1', 4', 5
**4'**	6.32; dq (6.5, 6.5)	69.0	3', 5'	2', 3', 5', 1''
**5'**	1.30; d (6.6)	20.2	4'	3',4'
**1''**	—	177.0	—	—
**2''**	2.48; qq (7.0, 7.0)	34.7	3''/4''	1'', 3''
**3''**	1.10; d (6.9)	19.2	2''	1'', 2''
**4''**	1.10; d (6.9)	29.5	2''	1'', 2''
**NH**	6.45; d (8.3)	—	14	1', 14
**OH**	2.95: d (1.6)	—	10	9, 10, 11, 12
**OH**	3.71	—	—	1, 2, 17

Supplementary Table 8: NMR chemical shifts of spliceostatin M (21) in acetonitrile-d_3

No.	δ_H, mult, (J in Hz)	δ_c	COSY	HMBC
**1**	—	97.2	—	—
**2**	1.32; m, 2.11; m	41.8	—	1.32: 1, 3, 4, 18; 2.09: 1, 3, 17, 18
**3**	—	55.9	—	—
**4**	1.19; m, 1.90; m	38.6	5	1.18: 2, 3; 1.90: 5
**5**	4.66; dddd (1.2, 2.6, 6.3, 11.9)	68.9	4, 6	6, 7
**6**	5.74; dd (6.3, 15.8)	131.1	5, 7, weak 10	4, 5, 8
**7**	6.29; d (15.8)	135.3, 134.1	6	5, 8, 9, 19
**8**	—	138.1	—	—
**9**	5.34; d (9.7)	130.3	10	7, 11, 19
**10**	4.27; m	69.2	9, 11	8, 9, 11,
**11**	3.37; dd (2.6, 8.6)	85.5	10, 12	9, 10, 13, 15, 20
**12**	1.66 ; m	28.1	20	10, 13, 14, 20
**13**	1.80, 1.90; m	36.7	—	11, 12, 14, 15, 20
**14**	3.83; dddd (2.2, 2.2, 4.4, 8.8)	47.8	NH, 13,15	—
**15**	3.71; dq (2.2, 6.4)	76.7	16	11, 14, 16
**16**	1.15; d (6.4)	17.9	15	14, 15
**17**	1.36; s	30.0	—	1, 2, 3, 5
**18**	2.53; d (4.6); 2.55; d (4.6)	51.2	—	2, 3, 4
**19**	1.85; d (1.3)	13.4	9	7, 8, 9
**20**	0.89; d (7.3)	15.2	12	11, 12, 13
**1'**	—	165.8	—	—
**2'**	5.88; d (11.7)	123.4	3'	1', 3', 4', 5'
**3'**	5.92; dd (7.0, 11.7)	144.8	2', 4',	1', 4', 5'
**4'**	6.34; dq (6.7, 6.5)	69.0	3', 5'	2', 3', 5', 1"
**5'**	1.30; d (6.5)	20.3	4'	3',4'
**1"**	—	174.4	—	—
**2"**	2.27; q (7.6)	28.3	3"	1", 3"
**3"**	1.05; d (7.5)	9.4	2"	1", 2"
**NH**	6.45; d (8.6)	—	14	1', 14
**OH**	2.95; d (1.6)	—	10	9, 10, 11
**OH**	3.7	—	—	1, 2, 17

Supplementary Table 9: Primers used in this study

primer	sequence
4314-F1	CATGTTCGCTCGACCGGTATCAAAGC
4314-R1	GAAGCAGCTCCAGCCTACACAAACGTTGTCCCTTAGGTTTCTAGTGCCTTG
4314-F2	GGTCGACGGATCCCCGGAATGGGTGTCGTCATGAATAACTCCAATGAGATTGCTAAC
4314-R2	GGACTTTCCGTAGGTGTTTACCAATTGCAGG
FRT-KM-F	GTGTAGGCTGGAGCTGCTTC
FRT-KM-R	ATTCCGGGGATCCGTCGACC

References

1 Helfrich, E. J. N. & Piel, J. Biosynthesis of polyketides by *trans*-AT polyketide synthases. *Nat. Prod. Rep.* **33**, 231-316, (2016).

2 Piel, J. Biosynthesis of polyketides by *trans*-AT polyketide synthases. *Nat. Prod. Rep.* **27**, 996-1047, (2010).
