# Supplementary material for: Evolution of combinatorial diversity in trans-acyltransferase polyketide synthase assembly lines across bacteria
Source: Nat Commun. 2021 Mar 3;12:1422. doi: 10.1038/s41467-021-21163-x (PMC7930024; doi:10.1038/s41467-021-21163-x)
Supplement: Supplementary file 2 — Description of Additional Supplementary Files [file 41467_2021_21163_MOESM2_ESM.docx]

**Description of Additional Supplementary Files**

**Supplementary Dataset 1: Comparison of TransATor and transPACT outputs for functionally assigned trans-AT PKS BGCs that were not included in the training dataset.** Left column: actual polyketide structures and manual assignments of KS substrates based on biosynthetic hypotheses. Middle and right columns: KS substrate predictions of TransATor and transPACT, respectively, as well as the TransATor-generated structure of the polyketide core. Green: match; red, mismatch; orange: no assignment.

**Supplementary Dataset 2:** **Dendrogram representation of Figure 3A showing conserved module blocks shared between characterized *trans*-AT PKS and orphan biosynthetic gene clusters**. See Supplementary Table 2 for detailed number legend. Colors represent classes of phylogenetic clades. Red: amino acids; light blue: β-hydroxyl groups; light green: double bonds; dark blue: *E*-configured double bonds; light red: non-elongating KSs; dark green: starters; light purple: *Z*-configured double bonds; grey: others. Molecules linked to characterized biosynthetic gene clusters help identify families of *trans*-AT PKSs. Orange boxes indicate likely incomplete biosynthetic gene clusters as determined by their small distance to contig borders (<5 kb). An interactive representation of the dendrogram can be accessed here: [https://itol.embl.de/tree/474115015487031585082885#.](https://itol.embl.de/tree/474115015487031585082885)
