# Supplementary material for: Evolution of combinatorial diversity in trans-acyltransferase polyketide synthase assembly lines across bacteria
Source: Nat Commun. 2021 Mar 3;12:1422. doi: 10.1038/s41467-021-21163-x (PMC7930024; doi:10.1038/s41467-021-21163-x)
Supplement: Supplementary file 3 — Supplementary Dataset 1 [file 41467_2021_21163_MOESM3_ESM.pdf]

Compound	TransATor	TransPACT
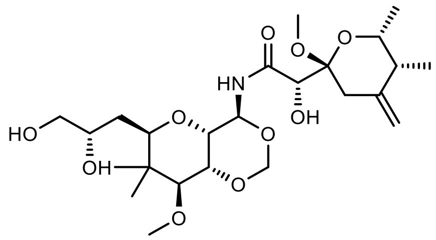<br><br>Mycalamide A	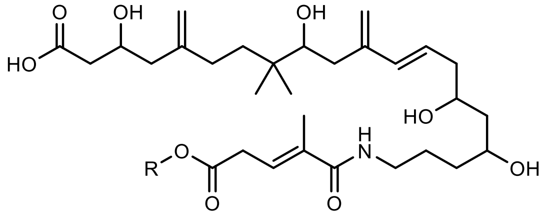<br><br>9/10	 <br>8/8; 2 not assigned
KS1: GNAT starter	KS1: GNAT starter	KS1: GNAT starter
KS2: α-Me β-OH	KS2: β-D-OH	KS2: not assigned
KS3: exomethyl/exoester	KS3: exomethyl/exoester	KS3: exomethyl/reduced β-Me
KS4: non-elongating (hemiacetale)	KS4: non-elongating (hemiacetale)	KS4: non-elongating (hemiacetale/β-OH)
KS5: amino acid (Gly)	KS5: amino acid (Gly)	KS5: amino acids
KS6: β-D-OH	KS6: β-D-OH	KS6: not assigned
KS7: α-(di)Me β-OH	KS7: α-(di)Me β-OH	KS7: α-Me β-L-OH
KS8: pyran	KS8: pyran, furan	KS8: pyran/furan
KS9: β-L-OH	KS9: β-L-OH	KS9: β-L-OH
KS10: oxygen insertion	KS10: oxidative rearrangement	KS10: oxygen insertion
KS11: unclear	KS11: reduced	KS11: reduced/shifted double bond
KS12: unclear	KS12: double bond (*E*-configured)	KS12: double bond (*E*-configured)

KS13: unclear	KS13: non-elongating (double bond)	KS13: non-elongating (double bond)
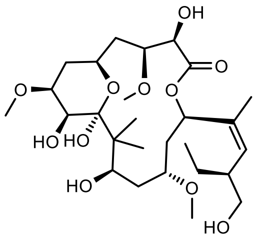<br>Peloruside A	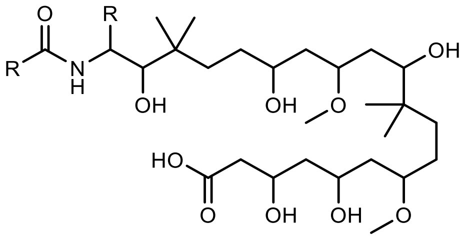<br>11/13	11/13
KS1: starter AL	KS1: amino acids	KS1: amino acids
KS2: α-Me β-OH	KS2: α-L-(di)Me β-OH	KS2: α-Me β-L-OH
KS3: non-elongating (α-Me β-AcO)	KS3: non-elongating	KS3: non-elongating (double bond)
KS4: non-elongating (α-Me double bond)	KS4: double bond (*E*-configured)	KS4: double bond (*E*-configured)
KS5: β-OH	KS5: β-D-OH	KS5: β-D-OH
KS6: non-elongating (β-OH)	KS6: non-elongating (β-D-OH)	KS6: non-elongating (β-OH)
KS7: β-OMe	KS7: β-OMe	KS7: β-OMe
KS8: α-(di)Me β-OH	KS8: α-L-(di)Me β-OH	KS8: α-Me β-L-OH
KS9: keto	KS9: completely reduced	KS9: reduced/shifted double bond
KS10: non-elongating (β-OH)	KS10: non-elongating (β-L-OH)	KS10: non-elongating (β-L-OH)

KS11: β-OMe<br>KS12: β-OH<br>KS13: non-elongating (β-OH)	KS11: β-OMe<br>KS12: β-D-OH<br>KS13: non-elongating (β-OH)	KS11: β-OMe<br>KS12: β-D-OH<br>KS13: non-elongating (hemiacetal/β-OH)
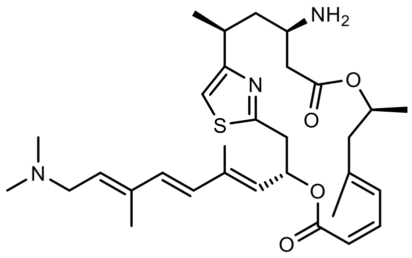<br>Pateamine A<br><br>KS1: amino acid<br>KS2: α-Me double bond<br>KS3: double bond<br>KS4: β-Me double bond<br>KS5: β-OH<br>KS6: non-elongating<br>KS7: thiazole<br>KS8: β-Me<br>KS9: β-NH_2<br>KS10: acetyl starter	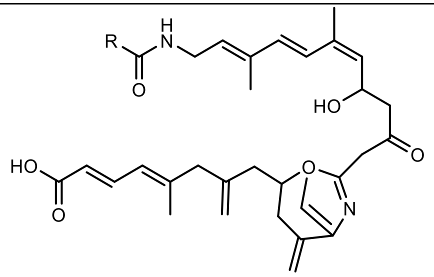<br>12/14<br><br>KS1: amino acid (Gly)<br>KS2: α-Me double bond<br>KS3: double bond (*E*-configured)<br>KS4: β-OMe or β-Me double bond<br>KS5: β-D-OH (some with α-L-Me)<br>KS6: non-elongating (oxazole/thiazole)<br>KS7: amino acids (oxa/thia)<br>KS8: β-OMe or β-Me double bond<br>KS9: pyran/furan rings<br>KS10: acetyl starter	9/12; 2 not assigned<br><br>KS1: amino acid (Gly)<br>KS2: α-Me double bond (*E*-configured)<br>KS3: double bond (*E*-configured)<br>KS4: β-Me double bond (*E*-configured)<br>KS5: not assigned<br>KS6: non-elongating (oxazole/thiazole)<br>KS7: oxazole/thiazole<br>KS8: exomethyl/reduced β-Me<br>KS9: β-OH/keto<br>KS10: acetyl starter

KS11: β-Me<br>KS12: β-Me double bond<br>KS13: non-elongating (β-Me double bond)<br>KS14: double bond	KS11: exomethyl/exoester<br>KS12: β-OMe or β-Me double bond<br>KS13: non-elongating (bimodule β-D-OH)<br>KS14: double bond (*Z*-configured)	KS11: not assigned<br>KS12: β-Me double bond (*E*-configured)<br>KS13: non-elongating (bimodule β-OH)<br>KS14: double bond (*Z*-configured)
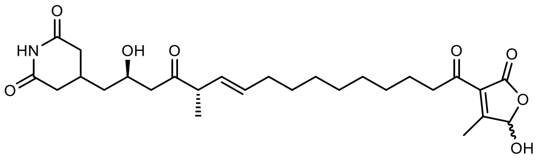<br><br>Gladiofungin A<br>KS1: starter AMT<br>KS2: double bond<br>KS3: glutarimide<br>KS4: β-OH<br>KS5: α-Me keto<br>KS6: α-Me double bond<br>KS7: double bond	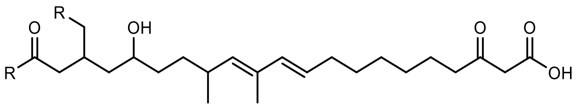<br><br>8/10<br>KS1: unusual starter (AMT/Succinate)<br>KS2: non-elongating (double bond)<br>KS3: vinylogous chain branching (including glutarimides)<br>KS4: β-D-OH<br>KS5: α-Me reduced/keto/D-OH<br>KS6: α-Me double bond<br>KS7: double bond (mostly *E*-configured)<br>KS8: completely reduced	7/9; 1 not assigned<br>KS1: unusual starter (AMT)<br>KS2: non-elongating (double bond before branching)<br>KS3: vinylogous chain branching (including glutarimides)<br>KS4: not assigned<br>KS5: α-Me reduced/β-keto/β-OH<br>KS6: α-Me double bond (*E*-configured)<br>KS7: double bond (*E*-configured)<br>KS8: reduced/shifted double bond

KS8: completely reduced<br>KS9: completely reduced<br>KS10: keto	KS9: completely reduced<br>KS10: completely reduced	KS9: reduced/shifted double bond<br>KS10: reduced/shifted double bond
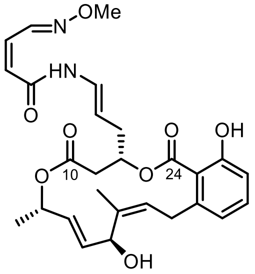<br>Lobatamide A<br><br>KS1: non-elongating (amino acid)<br>KS2: non-elongating (oxime)<br>KS3: methylated oxime<br>KS4: non-elongating (β-OH)<br>KS5: amino acids (Gly)<br>KS6: shifted double bond<br>KS7: β-OH<br>KS8: α-Me and oxygen insertion (BVMO)<br>KS9: double bond	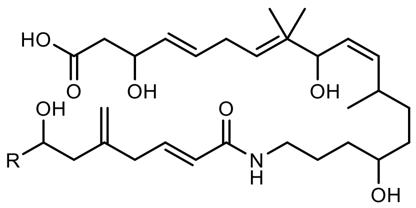<br>7/13<br><br>KS1: non-elongating (α-Me completely reduced or shifted double bond)<br>KS2: starters or β-OH<br>KS3: exomethyl/exoester<br>KS4: non-elongating (bimodule β-D-OH)<br>KS5: amino acids<br>KS6: completely reduced<br>KS7: β-L-OH<br>KS8: α-Me reduced/keto/D-OH<br>KS9: double bond (*E*-configured)	7/11; 2 not assigned<br><br>KS1: non-elongating<br>KS2: non-elongating (reduced/β-OH)<br>KS3: exomethyl/reduced β-Me<br>KS4: non-elongating (bimodule β-OH)<br>KS5: amino acids<br>KS6: reduced/shifted double bond<br>KS7: not assigned<br>KS8: α-Me reduced/β-keto/β-OH<br>KS9: double bond (*E*-configured)

KS10: α-Me β-OH<br>KS11: shifted double bond<br>KS12: β-keto (iterative)<br>KS13: β-OH	KS10: α-L-(di)Me β-OH<br>KS11: shifted double bond (some with α-Me)<br>KS12: β-keto or double bond<br>KS13: *cis*-AT PKS	KS10: α-Me β-L-OH<br>KS11: shifted double bond<br>KS12: β-keto<br>KS13: not assigned
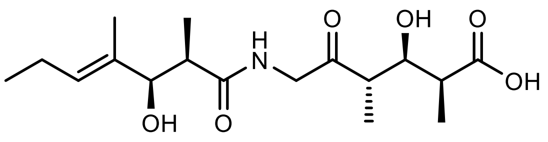<br>Alpiniamide A<br>KS1: amino acid (Gly)<br>KS2: α-Me keto<br>KS3: non-elongating (α-Me β-OH)	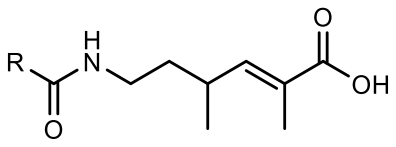<br>3/3<br>KS1: amino acids<br>KS2: α-Me reduced/keto/D-OH<br>KS3: non-elongating (α-Me completely reduced or double bond)	2/2; 1 not assigned<br>KS1: not assigned<br>KS2: α-Me reduced/β-keto/β-OH<br>KS3: non-elongating
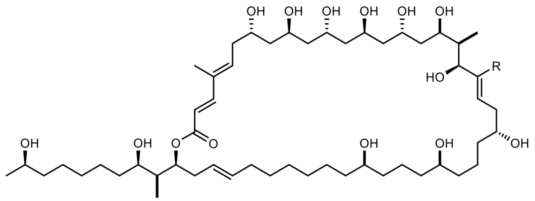<br>Lacunalides	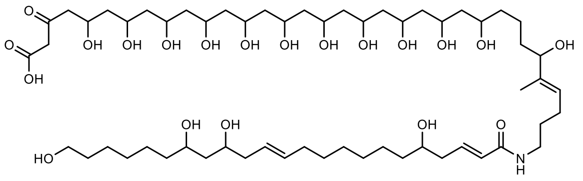<br>22/25	16/18; 7 not assigned

KS1: acetyl starter	KS1: various starters	KS1: acetyl/aromatic starter
KS2: β-OH	KS2: β-D-OH (some with α-L-Me)	KS2: β-OH/double bond (*E*-configure)
KS3: completely reduced	KS3: completely reduced	KS3: reduced/shifted double bond
KS4: completely reduced	KS4: completely reduced	KS4: reduced/shifted double bond
KS5: α-Me β-OH	KS5: β-D-OH (some with α-L-Me)	KS5: α-Me β-OH
KS6: β-OH	KS6: β-D-OH (some with α-L-Me)	KS6: α-Me β-OH
KS7: *E*-double bond	KS7: double bond (mostly *E*-configured)	KS7: mainly double bond (*E*-configured)
KS8: completely reduced	KS8: completely reduced	KS8: reduced/shifted double bond
KS9: completely reduced	KS9: completely reduced	KS9: reduced/shifted double bond
KS10: completely reduced	KS10: completely reduced	KS10: reduced/shifted double bond
KS11: β-OH	KS11: β-D-OH	KS11: β-OH/double bond (*E*-configure)
KS12: completely reduced	KS12: completely reduced	KS12: reduced/shifted double bond
KS13: β-OH	KS13: β-D-OH (some with α-L-Me)	KS13: not assigned
KS14: completely reduced	KS14: completely reduced	KS14: reduced/shifted double bond
KS15: β-OH	KS15: β-D-OH	KS15: not assigned
KS16: α-Me *E*-double bond	KS16: β-D-OH (some with α-L-Me)	KS16: not assigned
KS17: α-Me β-OH	KS17: β-D-OH (some with α-L-Me)	KS17: not assigned
KS18: β-OH	KS18: β-L-OH	KS18: β-L-OH
KS19: β-OH	KS19: β-D-OH (some with α-L-Me)	KS19: β-D-OH

KS20: β-OH<br>KS21: β-OH<br>KS22: β-OH<br>KS23: β-OH<br>KS24: α-Me *E*-double bond<br>KS25: non-elongating (α-Me *E*-double bond)	KS20: β-L-OH<br>KS21: β-D-OH<br>KS22: β-L-OH<br>KS23: β-D-OH (some with α-L-Me)<br>KS24: β-D-OH (some with α-L-Me)<br>KS25: β-D-OH	KS20: β-L-OH<br>KS21: not assigned<br>KS22: β-L-OH<br>KS23: not assigned<br>KS24: not assigned<br>KS25: non-elongating (reduced/β-OH)
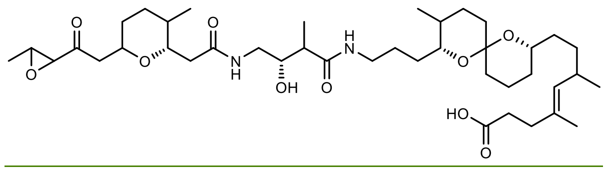<br>Lagriamide<br><br>KS1: GNAT starter<br>KS2: double bond<br>KS3: β-OH<br>KS4: double bond<br>KS5: α-Me reduced<br>KS6: non-elongating (β-OH)<br>KS7: amino acid (Gly)	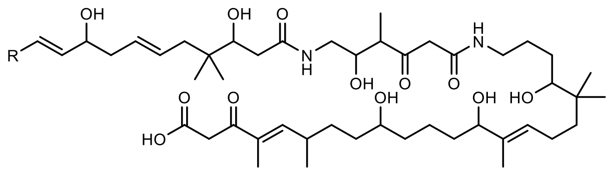<br>15/18<br><br>KS1: *cis*-AT PKS<br>KS2: double bond (mostly *E*-configured)<br>KS3: β-L-OH<br>KS4: double bond (mostly *E*-configured)<br>KS5: α-Me<br>KS6: non-elongating (double bond)<br>KS7: amino acids	12/15; 3 not assigned<br><br>KS1: *cis*-AT PKS-like<br>KS2: double bond (*E*-configured)<br>KS3: β-L-OH<br>KS4: double bond (*E*-configured)<br>KS5: α-Me reduced/β-keto/β-OH<br>KS6: non-elongating (double bond)<br>KS7: not assigned

KS8: α-Me β-OH<br>A: Gly<br>KS9: amino acid (Gly)<br>KS10: completely reduced<br>KS11: α-Me β-OH<br>KS12: completely reduced<br>KS13: β-OH<br>KS14: β-OH<br>KS15: completely reduced<br>KS16: β-OH<br>KS17: α-Me double bond<br>KS18: non-elongating (reduced)	KS8: α-L-Me β-OH<br><br>KS9: amino acids<br>KS10: completely reduced<br>KS11: α-L-(di)Me β-OH<br>KS12: completely reduced<br>KS13: α-Me double bond<br>KS14: β-D-OH<br>KS15: completely reduced<br>KS16: β-L-OH<br>KS17: α-Me reduced/keto/D-OH<br>KS18: α-Me double bond	KS8: α- Me β-L-OH<br><br>KS9: not assigned<br>KS10: reduced/shifted double bond<br>KS11: α-Me β-L-OH<br>KS12: reduced/shifted double bond<br>KS13: α-Me double bond (*E*-configured)<br>KS14: not assigned<br>KS15: reduced/shifted double bond<br>KS16: β-L-OH<br>KS17: α-Me reduced/β-keto/β-OH<br>KS18: α-Me double bond (*E*-configured)
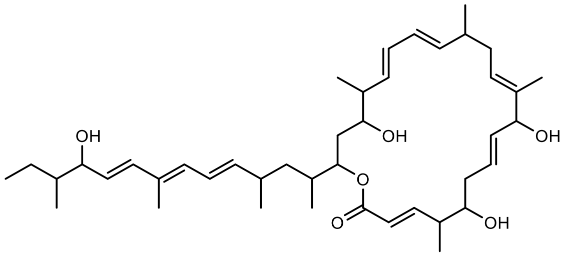<br>Macrobrevin<br><br>KS1: starter	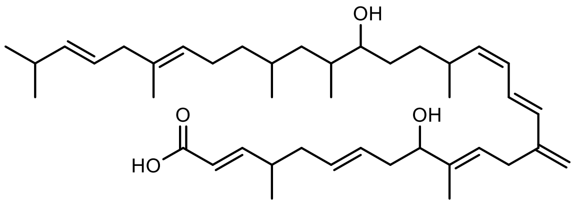<br>17/20<br><br>KS1: various starters	17/20<br><br>KS1: acetyl/aromatic starter

KS2: α-Me	KS2: α-Me reduced/keto/D-OH	KS2: α-Me reduced/β-keto/β-OH
KS3: β-OH	KS3: double bond (mostly *E*-configured)	KS3: mainly double bond (*E*-configured)
KS4: α-Me double bond	KS4: α-Me reduced/keto/D-OH	KS4: α-Me reduced/β-keto/β-OH
KS5: non-elongating (double bond)	KS5: non-elongating (mostly α-Me double bond)	KS5: non-elongating (α-Me double bond (*E*-configured))
KS6: shifted double bond	KS6: shifted double bond (some with α-Me)	KS6: shifted double bond
KS7: α-Me shifted double bond	KS7: α-Me reduced/keto/D-OH	KS7: α-Me reduced/β-keto/β-OH
KS8: α-Me	KS8: α-Me reduced/keto/D-OH	KS8: α-Me reduced/β-keto/β-OH
KS9: β-OH	KS9: β- D-OH (some with α-L-Me)	KS9: β-D-OH
KS10: α-Me β-OH	KS10: α-Me reduced/keto/D-OH	KS10: α-Me reduced/β-keto/β-OH
KS11: double bond	KS11: double bond (mostly *E*-configured)	KS11: mainly double bond (*E*-configured)
KS12: double bond	KS12: double bond (mostly *E*-configured)	KS12: mainly double bond (*E*-configured)
KS13: β-Me	KS13: *cis*-AT PKS	KS13: *cis*-AT PKS-like
KS14: β-Me	KS14: exomethyl/exoester	KS14: exomethyl/reduced β-Me
KS15: α-Me double bond	KS15: α-Me double bond (*E*-configured)	KS15: α-Me double bond (*E*-configured)
KS16: β-OH	KS16: β-L-OH	KS16: β-L-OH
KS17: non-elongating (β-OH)	KS17: non-elongating (bimodule β-D-OH)	KS17: non-elongating (bimodule β-OH)
KS18: shifted double bond	KS18: double bond (*Z*-configured)	KS18: double bond (*Z*-configured)
KS19: α-Me β-OH	KS19: α-Me reduced/keto/D-OH	KS19: α-Me reduced/β-keto/β-OH
KS20: non-elongating (double bond)	KS20: non-elongating (double bond)	KS20: non-elongating (double bond)

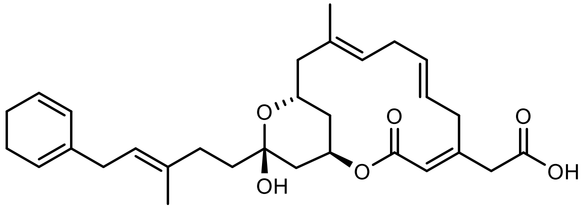	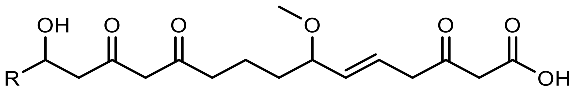	
Ripostatin	8/9	5/6; 3 not assigned
KS1: *cis*-AT PKS (aromatic starter)	KS1: *cis*-AT PKS	KS1: *cis*-AT PKS-like
KS2: *cis*-AT PKS	KS2: *cis*-AT PKS	KS2: not assigned
KS3: completely reduced	KS3: *cis*-AT PKS	KS3: not assigned
KS4: keto	KS4: β-D-OH or β-keto	KS4: β-OH/keto
KS5: β-OH	KS5: β-D-OH or β-keto	KS5: β-OH/keto
KS6: β-OH	KS6: various specificities	KS6: β-OH/keto
KS7: β-Me double bond	KS7: β-OMe or β-Me double bond	KS7: not assigned
KS8: completely reduced	KS8: bimodule (β-D-OH)	KS8: non-elongating (bimodule β-OH)
KS9: shifted double bond	KS9: shifted double bond (some with α-Me)	KS9: shifted double bond

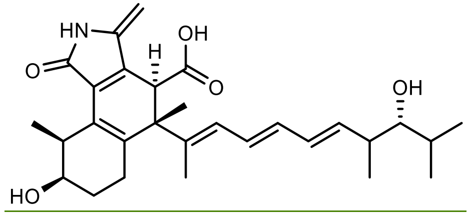	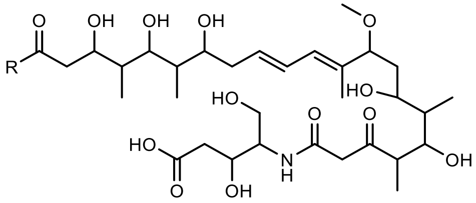	
Pyxipyrrolone A	6/9	5/9, 1 not assigned, 1 new functionality
KS1: starter	KS1: acetyl starter	KS1: acetyl starter
KS2: starter	KS2: β-D-OH or β-keto	KS2: β-OH/keto
KS3: skipped module	KS3: α-L-Me reduced or OH	KS3: reduced α-Me/shifted double bond α-Me
KS4: α-Me β-OH	KS4: α-L-Me reduced or OH	KS4: β-keto
KS5: unknown	KS5: β-D-OH	KS5: not assigned
KS6: double bond	KS6: double bond (*E*-configured)	KS6: double bond
KS7: α-Me double bond	KS7: α-Me double bond	KS7: α-Me double bond (*E*-configured)
KS8: β-branching	KS8: β-OMe or β-Me double bond	KS8: β-Me
KS9: double bond	KS9: α-L-Me reduced or OH	KS9: reduced α-Me/shifted double bond α-Me
KS10: α-Me keto	KS10: α-Me shifted double bond or OH	KS10: α-Me β-OH
KS11: amino acid (Ser)	KS11: amino acids	KS11: amino acids

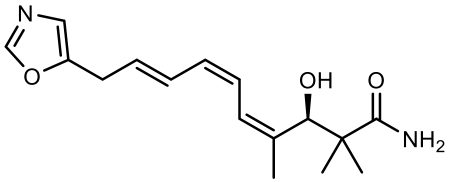<br>Phthoxazolin<br><br>KS1: Gly<br>KS2: keto<br>KS3: double bond<br>KS4: double bond<br>KS5: α-Me double bond<br>KS6: amino acid<br>KS7: double bond<br>KS8: non-elongating (β-OH)<br>KS9: double bond<br>KS10: non-elongating (double bond)	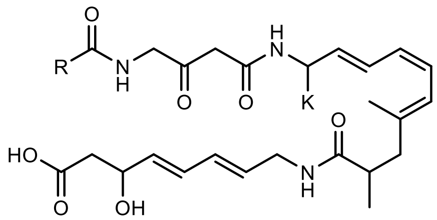<br>9/10<br><br>KS1: amino acids<br>KS2: amino acids<br>KS3: double bond<br>KS4: double bond<br>KS5: α-Me double bond<br>KS6: amino acids (Gly)<br>KS7: double bond (*E*-configured)<br>KS8: non-elongating (bimodule β-D-OH)<br>KS9: double bond (mostly *E*-configured)<br>KS10: non-elongating (double bond)	9/10<br><br>KS1: amino acids<br>KS2: amino acids<br>KS3: double bond<br>KS4: double bond (*Z*-configured)<br>KS5: α-Me double bond (*E*-configured)<br>KS6: amino acids (Gly)<br>KS7: double bond<br>KS8: non-elongating (bimodule β-OH)<br>KS9: double bond (*E*-configured)<br>KS10: non-elongating (double bond)
	Total: 127/154 (82.5%)	108/133 (81.2%)
