# Supplementary material for: Evolution of combinatorial diversity in trans-acyltransferase polyketide synthase assembly lines across bacteria
Source: Nat Commun. 2021 Mar 3;12:1422. doi: 10.1038/s41467-021-21163-x (PMC7930024; doi:10.1038/s41467-021-21163-x)
Supplement: Supplementary file 4 — Supplementary Dataset 2 [file 41467_2021_21163_MOESM4_ESM.pdf]

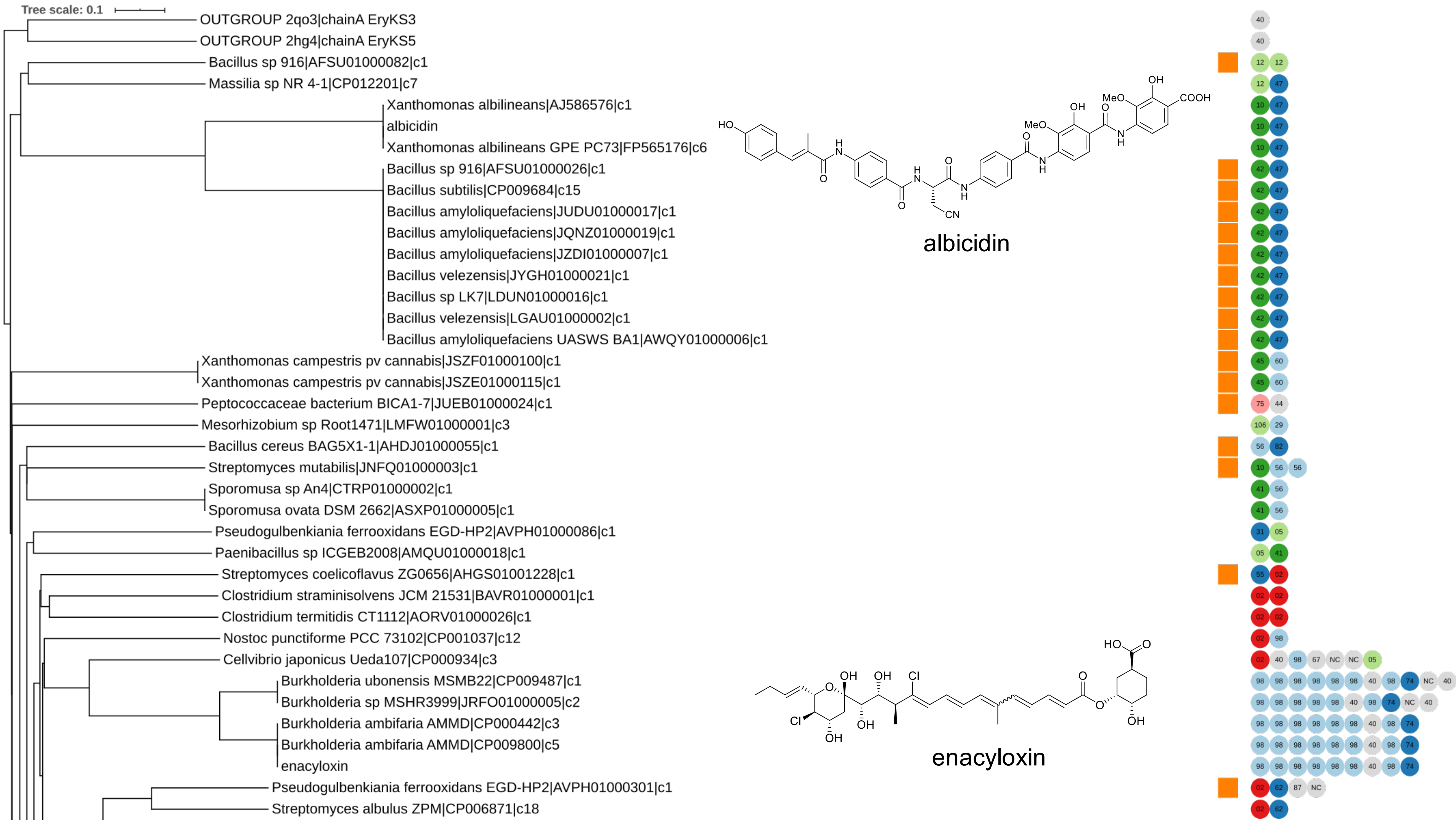
Tree scale: 0.1
OUTGROUP 2qo3|chainA EryKS3
OUTGROUP 2hg4|chainA EryKS5
Bacillus sp 916|AFSU01000082|c1
Massilia sp NR 4-1|CP012201|c7
Xanthomonas albilineans|AJ586576|c1
albicidin
Xanthomonas albilineans GPE PC73|FP565176|c6
Bacillus sp 916|AFSU01000026|c1
Bacillus subtilis|CP009684|c15
Bacillus amyloliquefaciens|JUDU01000017|c1
Bacillus amyloliquefaciens|JQNZ01000019|c1
Bacillus amyloliquefaciens|JZDI01000007|c1
Bacillus velezensis|JYGH01000021|c1
Bacillus sp LK7|LDUN01000016|c1
Bacillus velezensis|LGAU01000002|c1
Bacillus amyloliquefaciens UASWS BA1|AWQY01000006|c1
Xanthomonas campestris pv cannabis|JSZF01000100|c1
Xanthomonas campestris pv cannabis|JSZE01000115|c1
Peptococcaceae bacterium BICA1-7|JUEB01000024|c1
Mesorhizobium sp Root1471|LMFW01000001|c3
Bacillus cereus BAG5X1-1|AHDJ01000055|c1
Streptomyces mutabilis|JNFQ01000003|c1
Sporomusa sp An4|CTRP01000002|c1
Sporomusa ovata DSM 2662|ASXP01000005|c1
Pseudogulbenkiania ferrooxidans EGD-HP2|AVPH01000086|c1
Paenibacillus sp ICGEB2008|AMQU01000018|c1
Streptomyces coelicoflavus ZG0656|AHGS01001228|c1
Clostridium straminisolvens JCM 21531|BAVR01000001|c1
Clostridium termitidis CT1112|AORV01000026|c1
Nostoc punctiforme PCC 73102|CP001037|c12
Cellvibrio japonicus Ueda107|CP000934|c3
Burkholderia ubonensis MSMB22|CP009487|c1
Burkholderia sp MSHR3999|JRFO01000005|c2
Burkholderia ambifaria AMMD|CP000442|c3
Burkholderia ambifaria AMMD|CP009800|c5
enacyloxin
Pseudogulbenkiania ferrooxidans EGD-HP2|AVPH01000301|c1
Streptomyces albulus ZPM|CP006871|c18
albicidin
enacyloxin

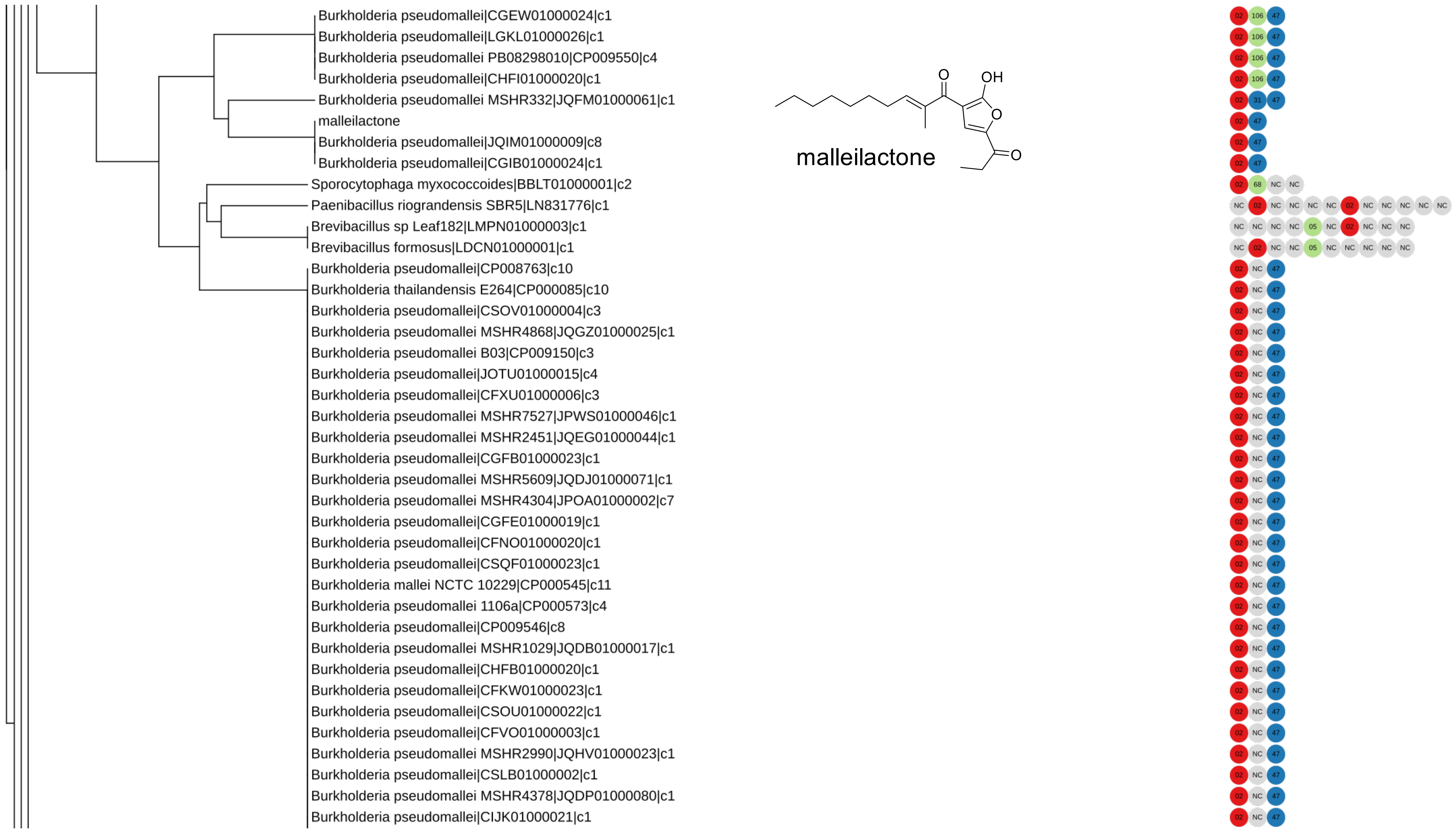
Burkholderia pseudomallei|CGEW01000024|c1
Burkholderia pseudomallei|LGKL01000026|c1
Burkholderia pseudomallei PB08298010|CP009550|c4
Burkholderia pseudomallei|CHFI01000020|c1
Burkholderia pseudomallei MSHR332|JQFM01000061|c1
malleilactone
Burkholderia pseudomallei|JQIM01000009|c8
Burkholderia pseudomallei|CGIB01000024|c1
Sporocytophaga myxococcoides|BBLT01000001|c2
Paenibacillus riograndensis SBR5|LN831776|c1
Brevibacillus sp Leaf182|LMPN01000003|c1
Brevibacillus formosus|LDCN01000001|c1
Burkholderia pseudomallei|CP008763|c10
Burkholderia thailandensis E264|CP000085|c10
Burkholderia pseudomallei|CSOV01000004|c3
Burkholderia pseudomallei MSHR4868|JQGZ01000025|c1
Burkholderia pseudomallei B03|CP009150|c3
Burkholderia pseudomallei|JOTU01000015|c4
Burkholderia pseudomallei|CFXU01000006|c3
Burkholderia pseudomallei MSHR7527|JPWS01000046|c1
Burkholderia pseudomallei MSHR2451|JQEG01000044|c1
Burkholderia pseudomallei|CGFB01000023|c1
Burkholderia pseudomallei MSHR5609|JQDJ01000071|c1
Burkholderia pseudomallei MSHR4304|JPOA01000002|c7
Burkholderia pseudomallei|CGFE01000019|c1
Burkholderia pseudomallei|CFNO01000020|c1
Burkholderia pseudomallei|CSQF01000023|c1
Burkholderia mallei NCTC 10229|CP000545|c11
Burkholderia pseudomallei 1106a|CP000573|c4
Burkholderia pseudomallei|CP009546|c8
Burkholderia pseudomallei MSHR1029|JQDB01000017|c1
Burkholderia pseudomallei|CHFB01000020|c1
Burkholderia pseudomallei|CFKW01000023|c1
Burkholderia pseudomallei|CSOU01000022|c1
Burkholderia pseudomallei|CFVO01000003|c1
Burkholderia pseudomallei MSHR2990|JQHV01000023|c1
Burkholderia pseudomallei|CSLB01000002|c1
Burkholderia pseudomallei MSHR4378|JQDP01000080|c1
Burkholderia pseudomallei|CIJK01000021|c1
O
OH
O
O
malleilactone

Burkholderia pseudomallei HBPUB10134a|CP008912|c11	02	NC	47
Burkholderia mallei|JPNX02000001|c6	02	NC	47
Burkholderia pseudomallei MSHR456|JQFN01000035|c1	02	NC	47
Burkholderia pseudomallei|CGFD01000016|c1	02	NC	47
Burkholderia pseudomallei MSHR5569|JQDL01000049|c1	02	NC	47
Burkholderia pseudomallei|CSKB01000018|c1	02	NC	47
Burkholderia pseudomallei|JPNP01000093|c1	02	NC	47
Burkholderia pseudomallei|CSKY01000020|c1	02	NC	47
Burkholderia thailandensis 34|CP010018|c6	02	NC	47
Burkholderia pseudomallei|CFWF01000023|c1	02	NC	47
Burkholderia pseudomallei|CSKO01000021|c1	02	NC	47
Burkholderia pseudomallei ABCPW 1|JQIJ01000004|c10	02	NC	47
Burkholderia oklahomensis C6786|CP009556|c1	02	NC	47
Burkholderia pseudomallei|JQIL01000005|c6	02	NC	47
Burkholderia pseudomallei TSV 43|JPQK01000002|c11	02	NC	47
Burkholderia pseudomallei|CP009156|c4	02	NC	47
Burkholderia pseudomallei|CSKR01000019|c1	02	NC	47
Burkholderia pseudomallei|CSPL01000022|c1	02	NC	47
Burkholderia pseudomallei BDU 2|JPVG01000006|c6	02	NC	47
Burkholderia mallei|LBFF01000094|c1	02	NC	47
Burkholderia pseudomallei|CSKL01000023|c1	02	NC	47
Burkholderia pseudomallei MSHR3951|JPVA01000004|c2	02	NC	47
Burkholderia pseudomallei|JOTW01000050|c5	02	NC	47
Burkholderia pseudomallei|CSOX01000022|c1	02	NC	47
Burkholderia pseudomallei TSV28|JQHU01000025|c1	02	NC	47
Burkholderia thailandensis Phuket 4W-1|AQQJ01000003|c7	02	NC	47
Burkholderia pseudomallei MSHR511|CP004024|c14	02	NC	47
Burkholderia pseudomallei|CFVN01000006|c1	02	NC	47
Burkholderia pseudomallei|CFWJ01000018|c1	02	NC	47
Burkholderia sp MSHR3999|JRFO01000005|c1	02	NC	47
Burkholderia pseudomallei|CGHG01000024|c1	02	NC	47
Burkholderia pseudomallei MSHR3965|CP009152|c5	02	NC	47
Burkholderia pseudomallei MSHR7334|JQDF01000080|c1	02	NC	47
Burkholderia pseudomallei|CFZI01000022|c1	02	NC	47
Burkholderia pseudomallei|CSKH01000018|c1	02	NC	47
Burkholderia pseudomallei|CGFC01000017|c1	02	NC	47
Burkholderia pseudomallei|CP008759|c4	02	NC	47
Burkholderia pseudomallei|CFWB01000029|c1	02	NC	47
Burkholderia pseudomallei TSV 48|CP009160|c2	02	NC	47
Burkholderia pseudomallei|CSKE01000003|c1	02	NC	47

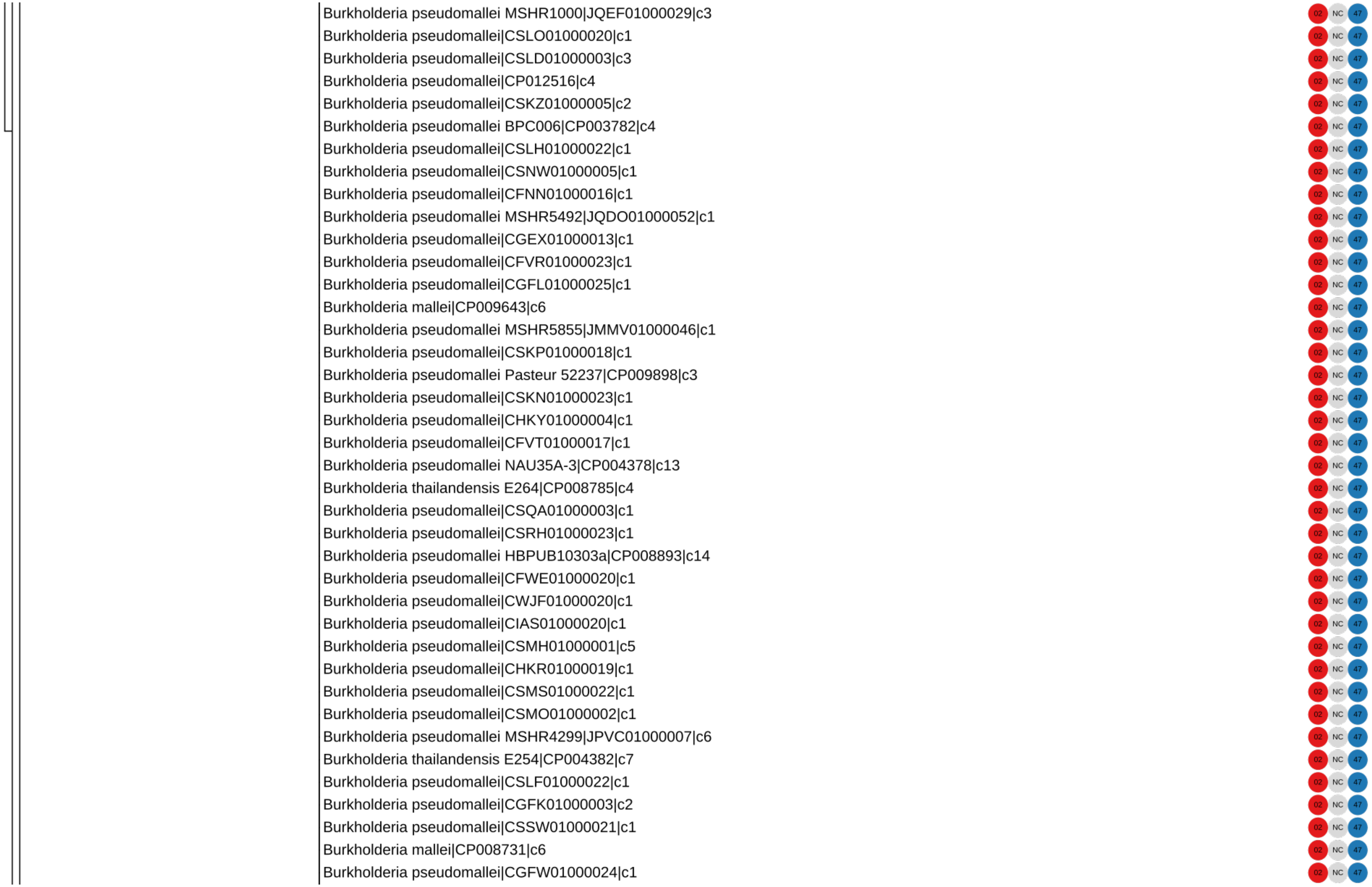
Burkholderia pseudomallei MSHR1000|JQEF01000029|c3 02 NC 47
Burkholderia pseudomallei|CSLO01000020|c1 02 NC 47
Burkholderia pseudomallei|CSLD01000003|c3 02 NC 47
Burkholderia pseudomallei|CP012516|c4 02 NC 47
Burkholderia pseudomallei|CSKZ01000005|c2 02 NC 47
Burkholderia pseudomallei BPC006|CP003782|c4 02 NC 47
Burkholderia pseudomallei|CSLH01000022|c1 02 NC 47
Burkholderia pseudomallei|CSNW01000005|c1 02 NC 47
Burkholderia pseudomallei|CFNN01000016|c1 02 NC 47
Burkholderia pseudomallei MSHR5492|JQDO01000052|c1 02 NC 47
Burkholderia pseudomallei|CGEX01000013|c1 02 NC 47
Burkholderia pseudomallei|CFVR01000023|c1 02 NC 47
Burkholderia pseudomallei|CGFL01000025|c1 02 NC 47
Burkholderia mallei|CP009643|c6 02 NC 47
Burkholderia pseudomallei MSHR5855|JMMV01000046|c1 02 NC 47
Burkholderia pseudomallei|CSKP01000018|c1 02 NC 47
Burkholderia pseudomallei Pasteur 52237|CP009898|c3 02 NC 47
Burkholderia pseudomallei|CSKN01000023|c1 02 NC 47
Burkholderia pseudomallei|CHKY01000004|c1 02 NC 47
Burkholderia pseudomallei|CFVT01000017|c1 02 NC 47
Burkholderia pseudomallei NAU35A-3|CP004378|c13 02 NC 47
Burkholderia thailandensis E264|CP008785|c4 02 NC 47
Burkholderia pseudomallei|CSQA01000003|c1 02 NC 47
Burkholderia pseudomallei|CSRH01000023|c1 02 NC 47
Burkholderia pseudomallei HBPUB10303a|CP008893|c14 02 NC 47
Burkholderia pseudomallei|CFWE01000020|c1 02 NC 47
Burkholderia pseudomallei|CWJF01000020|c1 02 NC 47
Burkholderia pseudomallei|CIAS01000020|c1 02 NC 47
Burkholderia pseudomallei|CSMH01000001|c5 02 NC 47
Burkholderia pseudomallei|CHKR01000019|c1 02 NC 47
Burkholderia pseudomallei|CSMS01000022|c1 02 NC 47
Burkholderia pseudomallei|CSMO01000002|c1 02 NC 47
Burkholderia pseudomallei MSHR4299|JPVC01000007|c6 02 NC 47
Burkholderia thailandensis E254|CP004382|c7 02 NC 47
Burkholderia pseudomallei|CSLF01000022|c1 02 NC 47
Burkholderia pseudomallei|CGFK01000003|c2 02 NC 47
Burkholderia pseudomallei|CSSW01000021|c1 02 NC 47
Burkholderia mallei|CP008731|c6 02 NC 47
Burkholderia pseudomallei|CGFW01000024|c1 02 NC 47

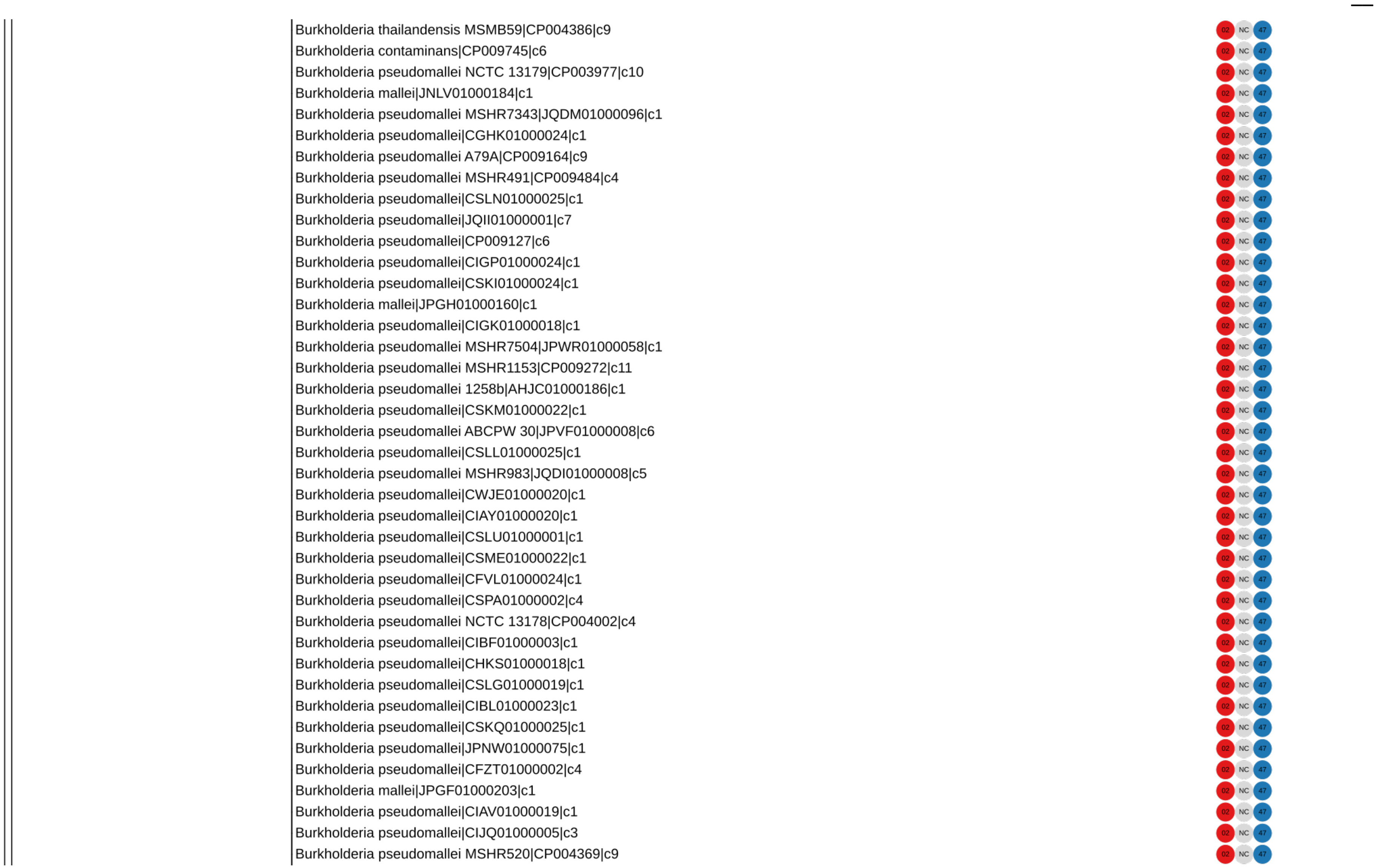
Burkholderia thailandensis MSMB59|CP004386|c9
Burkholderia contaminans|CP009745|c6
Burkholderia pseudomallei NCTC 13179|CP003977|c10
Burkholderia mallei|JNLV01000184|c1
Burkholderia pseudomallei MSHR7343|JQDM01000096|c1
Burkholderia pseudomallei|CGHK01000024|c1
Burkholderia pseudomallei A79A|CP009164|c9
Burkholderia pseudomallei MSHR491|CP009484|c4
Burkholderia pseudomallei|CSLN01000025|c1
Burkholderia pseudomallei|JQII01000001|c7
Burkholderia pseudomallei|CP009127|c6
Burkholderia pseudomallei|CIGP01000024|c1
Burkholderia pseudomallei|CSKI01000024|c1
Burkholderia mallei|JPGH01000160|c1
Burkholderia pseudomallei|CIGK01000018|c1
Burkholderia pseudomallei MSHR7504|JPWR01000058|c1
Burkholderia pseudomallei MSHR1153|CP009272|c11
Burkholderia pseudomallei 1258b|AHJC01000186|c1
Burkholderia pseudomallei|CSKM01000022|c1
Burkholderia pseudomallei ABCPW 30|JPVF01000008|c6
Burkholderia pseudomallei|CSLL01000025|c1
Burkholderia pseudomallei MSHR983|JQDI01000008|c5
Burkholderia pseudomallei|CWJE01000020|c1
Burkholderia pseudomallei|CIAY01000020|c1
Burkholderia pseudomallei|CSLU01000001|c1
Burkholderia pseudomallei|CSME01000022|c1
Burkholderia pseudomallei|CFVL01000024|c1
Burkholderia pseudomallei|CSPA01000002|c4
Burkholderia pseudomallei NCTC 13178|CP004002|c4
Burkholderia pseudomallei|CIBF01000003|c1
Burkholderia pseudomallei|CHKS01000018|c1
Burkholderia pseudomallei|CSLG01000019|c1
Burkholderia pseudomallei|CIBL01000023|c1
Burkholderia pseudomallei|CSKQ01000025|c1
Burkholderia pseudomallei|JPNW01000075|c1
Burkholderia pseudomallei|CFZT01000002|c4
Burkholderia mallei|JPGF01000203|c1
Burkholderia pseudomallei|CIAV01000019|c1
Burkholderia pseudomallei|CIJQ01000005|c3
Burkholderia pseudomallei MSHR520|CP004369|c9

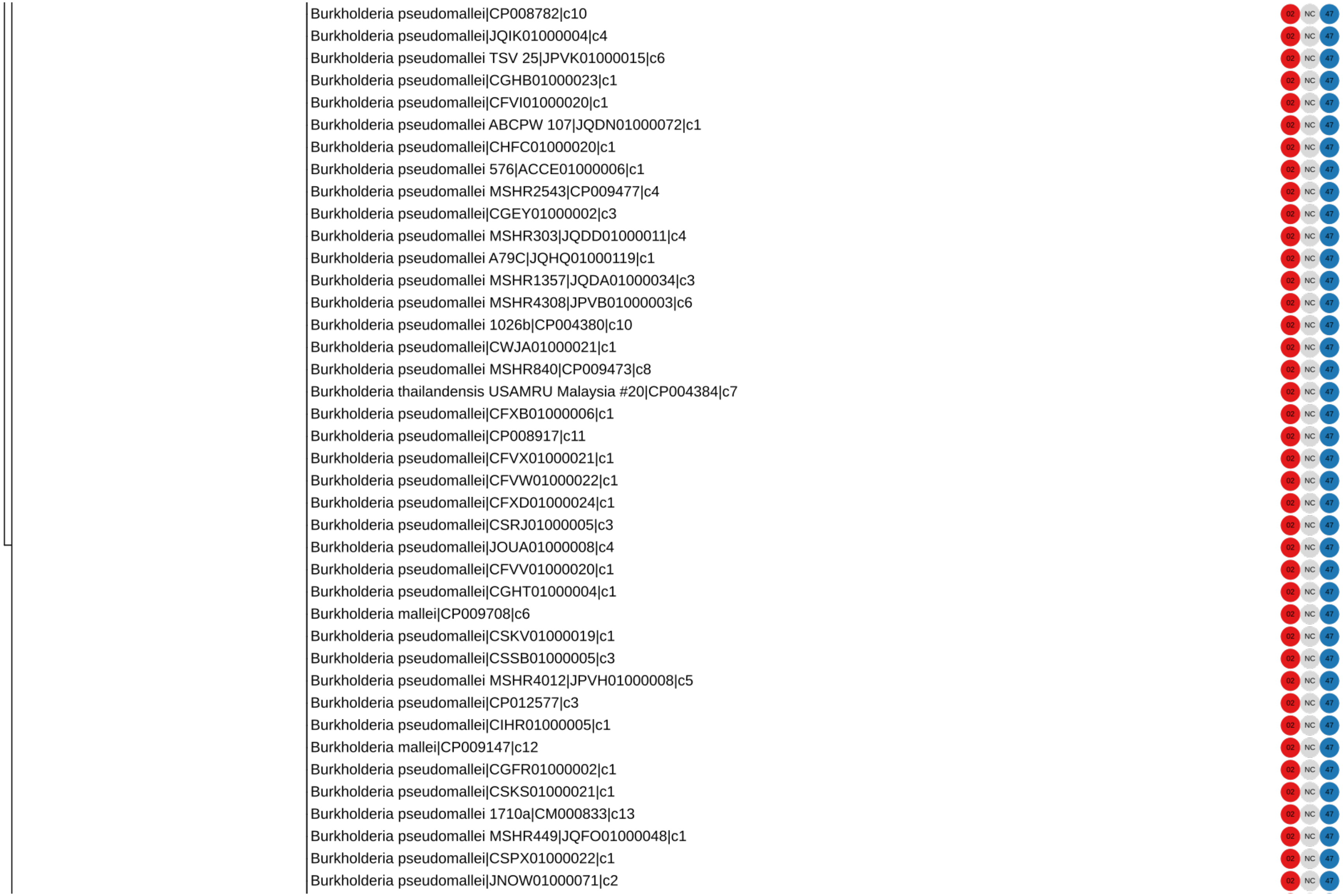
Burkholderia pseudomallei|CP008782|c10 02 NC 47
Burkholderia pseudomallei|JQIK01000004|c4 02 NC 47
Burkholderia pseudomallei TSV 25|JPVK01000015|c6 02 NC 47
Burkholderia pseudomallei|CGHB01000023|c1 02 NC 47
Burkholderia pseudomallei|CFVI01000020|c1 02 NC 47
Burkholderia pseudomallei ABCPW 107|JQDN01000072|c1 02 NC 47
Burkholderia pseudomallei|CHFC01000020|c1 02 NC 47
Burkholderia pseudomallei 576|ACCE01000006|c1 02 NC 47
Burkholderia pseudomallei MSHR2543|CP009477|c4 02 NC 47
Burkholderia pseudomallei|CGEY01000002|c3 02 NC 47
Burkholderia pseudomallei MSHR303|JQDD01000011|c4 02 NC 47
Burkholderia pseudomallei A79C|JQHQ01000119|c1 02 NC 47
Burkholderia pseudomallei MSHR1357|JQDA01000034|c3 02 NC 47
Burkholderia pseudomallei MSHR4308|JPVB01000003|c6 02 NC 47
Burkholderia pseudomallei 1026b|CP004380|c10 02 NC 47
Burkholderia pseudomallei|CWJA01000021|c1 02 NC 47
Burkholderia pseudomallei MSHR840|CP009473|c8 02 NC 47
Burkholderia thailandensis USAMRU Malaysia #20|CP004384|c7 02 NC 47
Burkholderia pseudomallei|CFXB01000006|c1 02 NC 47
Burkholderia pseudomallei|CP008917|c11 02 NC 47
Burkholderia pseudomallei|CFVX01000021|c1 02 NC 47
Burkholderia pseudomallei|CFVW01000022|c1 02 NC 47
Burkholderia pseudomallei|CFXD01000024|c1 02 NC 47
Burkholderia pseudomallei|CSRJ01000005|c3 02 NC 47
Burkholderia pseudomallei|JOUA01000008|c4 02 NC 47
Burkholderia pseudomallei|CFVV01000020|c1 02 NC 47
Burkholderia pseudomallei|CGHT01000004|c1 02 NC 47
Burkholderia mallei|CP009708|c6 02 NC 47
Burkholderia pseudomallei|CSKV01000019|c1 02 NC 47
Burkholderia pseudomallei|CSSB01000005|c3 02 NC 47
Burkholderia pseudomallei MSHR4012|JPVH01000008|c5 02 NC 47
Burkholderia pseudomallei|CP012577|c3 02 NC 47
Burkholderia pseudomallei|CIHR01000005|c1 02 NC 47
Burkholderia mallei|CP009147|c12 02 NC 47
Burkholderia pseudomallei|CGFR01000002|c1 02 NC 47
Burkholderia pseudomallei|CSKS01000021|c1 02 NC 47
Burkholderia pseudomallei 1710a|CM000833|c13 02 NC 47
Burkholderia pseudomallei MSHR449|JQFO01000048|c1 02 NC 47
Burkholderia pseudomallei|CSPX01000022|c1 02 NC 47
Burkholderia pseudomallei|JNOW01000071|c2 02 NC 47

Burkholderia pseudomallei MSHR733|JQEE01000021|c1	02	NC	47
Burkholderia pseudomallei|CIAW01000021|c1	02	NC	47
Burkholderia pseudomallei|CFVP01000023|c1	02	NC	47
Burkholderia pseudomallei|CSKG01000024|c1	02	NC	47
Burkholderia thailandensis 2002721723|CP004098|c11	02	NC	47
Burkholderia pseudomallei|CIJT01000024|c1	02	NC	47
Burkholderia pseudomallei|CFVZ01000021|c1	02	NC	47
Burkholderia pseudomallei|CSLE01000019|c1	02	NC	47
Burkholderia pseudomallei|CP008835|c10	02	NC	47
Burkholderia pseudomallei MSHR4300|JPQI01000001|c6	02	NC	47
Burkholderia pseudomallei TSV5|JQGY01000058|c1	02	NC	47
Burkholderia pseudomallei MSHR5855|CP008783|c5	02	NC	47
Burkholderia pseudomallei|CSNM01000023|c1	02	NC	47
Burkholderia pseudomallei|CFVY01000001|c3	02	NC	47
Burkholderia pseudomallei MSHR338|ATJY01000041|c1	02	NC	47
Burkholderia pseudomallei|CSKX01000018|c1	02	NC	47
Burkholderia pseudomallei MSHR4375|JPVI01000008|c1	02	NC	47
Burkholderia pseudomallei|CSSE01000023|c1	02	NC	47
Burkholderia pseudomallei MSHR1079|APNS01000004|c4	02	NC	47
Burkholderia pseudomallei|CIBE01000026|c1	02	NC	47
Burkholderia pseudomallei|CSNB01000002|c1	02	NC	47
Burkholderia pseudomallei|CSKU01000022|c1	02	NC	47
Burkholderia pseudomallei MSHR3458|JQOB01000006|c5	02	NC	47
Burkholderia pseudomallei|JOTT01000049|c3	02	NC	47
Burkholderia pseudomallei|CP008754|c10	02	NC	47
Burkholderia pseudomallei|CGFU01000024|c1	02	NC	47
Burkholderia pseudomallei|CSKD01000019|c1	02	NC	47
Burkholderia pseudomallei|CIBB01000002|c1	02	NC	47
Burkholderia pseudomallei|CHFD01000021|c1	02	NC	47
Burkholderia pseudomallei MSHR640|JQFP01000041|c2	02	NC	47
Burkholderia pseudomallei|JOTS01000016|c2	02	NC	47
Burkholderia pseudomallei|CSNO01000022|c1	02	NC	47
Burkholderia pseudomallei|CFZL01000023|c1	02	NC	47
Burkholderia pseudomallei MSHR684|JQDC01000005|c6	02	NC	47
Burkholderia pseudomallei|CSOW01000003|c3	02	NC	47
Burkholderia pseudomallei|CFWL01000024|c1	02	NC	47
Burkholderia mallei|CP009588|c11	02	NC	47
Burkholderia pseudomallei MSHR5608|JPWQ01000080|c1	02	NC	47
Burkholderia pseudomallei|JYBJ01000026|c3	02	NC	47

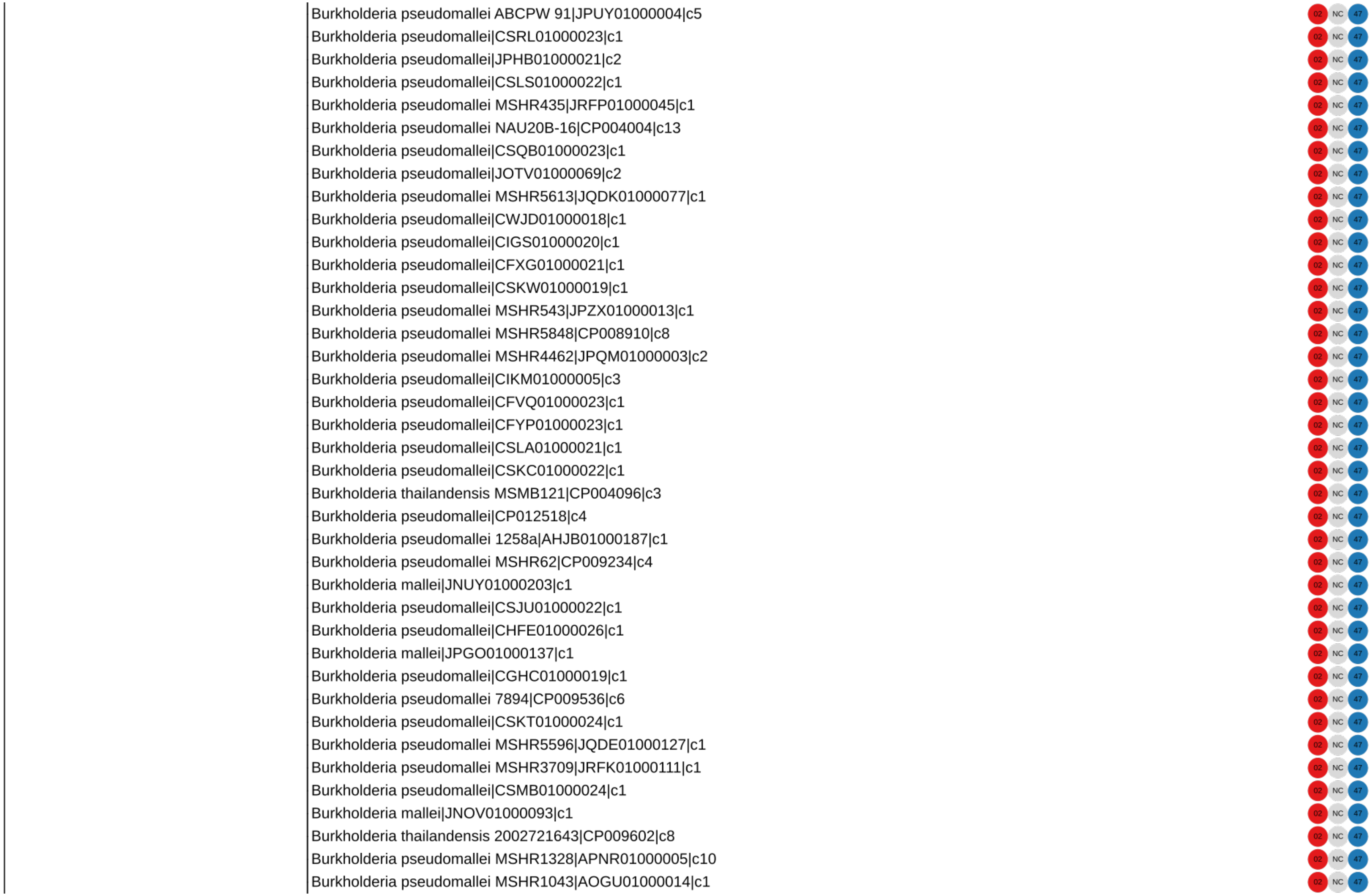
Burkholderia pseudomallei ABCPW 91|JPUY01000004|c5
Burkholderia pseudomallei|CSRL01000023|c1
Burkholderia pseudomallei|JPHB01000021|c2
Burkholderia pseudomallei|CSLS01000022|c1
Burkholderia pseudomallei MSHR435|JRFP01000045|c1
Burkholderia pseudomallei NAU20B-16|CP004004|c13
Burkholderia pseudomallei|CSQB01000023|c1
Burkholderia pseudomallei|JOTV01000069|c2
Burkholderia pseudomallei MSHR5613|JQDK01000077|c1
Burkholderia pseudomallei|CWJD01000018|c1
Burkholderia pseudomallei|CIGS01000020|c1
Burkholderia pseudomallei|CFXG01000021|c1
Burkholderia pseudomallei|CSKW01000019|c1
Burkholderia pseudomallei MSHR543|JPZX01000013|c1
Burkholderia pseudomallei MSHR5848|CP008910|c8
Burkholderia pseudomallei MSHR4462|JPQM01000003|c2
Burkholderia pseudomallei|CIKM01000005|c3
Burkholderia pseudomallei|CFVQ01000023|c1
Burkholderia pseudomallei|CFYP01000023|c1
Burkholderia pseudomallei|CSLA01000021|c1
Burkholderia pseudomallei|CSKC01000022|c1
Burkholderia thailandensis MSMB121|CP004096|c3
Burkholderia pseudomallei|CP012518|c4
Burkholderia pseudomallei 1258a|AHJB01000187|c1
Burkholderia pseudomallei MSHR62|CP009234|c4
Burkholderia mallei|JNUY01000203|c1
Burkholderia pseudomallei|CSJU01000022|c1
Burkholderia pseudomallei|CHFE01000026|c1
Burkholderia mallei|JPGO01000137|c1
Burkholderia pseudomallei|CGHC01000019|c1
Burkholderia pseudomallei 7894|CP009536|c6
Burkholderia pseudomallei|CSKT01000024|c1
Burkholderia pseudomallei MSHR5596|JQDE01000127|c1
Burkholderia pseudomallei MSHR3709|JRFK01000111|c1
Burkholderia pseudomallei|CSMB01000024|c1
Burkholderia mallei|JNOV01000093|c1
Burkholderia thailandensis 2002721643|CP009602|c8
Burkholderia pseudomallei MSHR1328|APNR01000005|c10
Burkholderia pseudomallei MSHR1043|AOGU01000014|c1

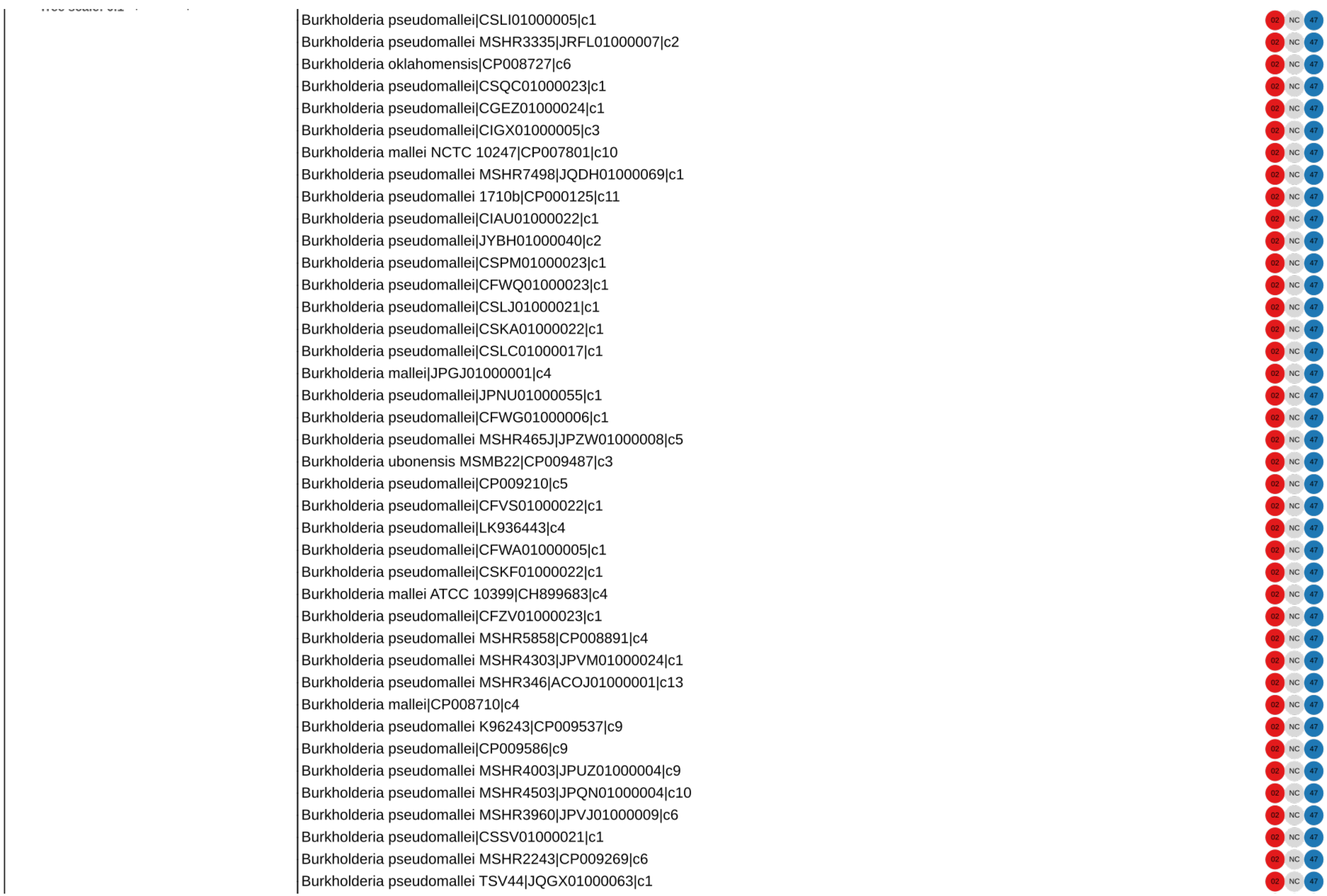
Burkholderia pseudomallei|CSLI01000005|c1 02 NC 47
Burkholderia pseudomallei MSHR3335|JRFL01000007|c2 02 NC 47
Burkholderia oklahomensis|CP008727|c6 02 NC 47
Burkholderia pseudomallei|CSQC01000023|c1 02 NC 47
Burkholderia pseudomallei|CGEZ01000024|c1 02 NC 47
Burkholderia pseudomallei|CIGX01000005|c3 02 NC 47
Burkholderia mallei NCTC 10247|CP007801|c10 02 NC 47
Burkholderia pseudomallei MSHR7498|JQDH01000069|c1 02 NC 47
Burkholderia pseudomallei 1710b|CP000125|c11 02 NC 47
Burkholderia pseudomallei|CIAU01000022|c1 02 NC 47
Burkholderia pseudomallei|JYBH01000040|c2 02 NC 47
Burkholderia pseudomallei|CSPM01000023|c1 02 NC 47
Burkholderia pseudomallei|CFWQ01000023|c1 02 NC 47
Burkholderia pseudomallei|CSLJ01000021|c1 02 NC 47
Burkholderia pseudomallei|CSKA01000022|c1 02 NC 47
Burkholderia pseudomallei|CSLC01000017|c1 02 NC 47
Burkholderia mallei|JPGJ01000001|c4 02 NC 47
Burkholderia pseudomallei|JPNU01000055|c1 02 NC 47
Burkholderia pseudomallei|CFWG01000006|c1 02 NC 47
Burkholderia pseudomallei MSHR465J|JPZW01000008|c5 02 NC 47
Burkholderia ubonensis MSMB22|CP009487|c3 02 NC 47
Burkholderia pseudomallei|CP009210|c5 02 NC 47
Burkholderia pseudomallei|CFVS01000022|c1 02 NC 47
Burkholderia pseudomallei|LK936443|c4 02 NC 47
Burkholderia pseudomallei|CFWA01000005|c1 02 NC 47
Burkholderia pseudomallei|CSKF01000022|c1 02 NC 47
Burkholderia mallei ATCC 10399|CH899683|c4 02 NC 47
Burkholderia pseudomallei|CFZV01000023|c1 02 NC 47
Burkholderia pseudomallei MSHR5858|CP008891|c4 02 NC 47
Burkholderia pseudomallei MSHR4303|JPVM01000024|c1 02 NC 47
Burkholderia pseudomallei MSHR346|ACOJ01000001|c13 02 NC 47
Burkholderia mallei|CP008710|c4 02 NC 47
Burkholderia pseudomallei K96243|CP009537|c9 02 NC 47
Burkholderia pseudomallei|CP009586|c9 02 NC 47
Burkholderia pseudomallei MSHR4003|JPUZ01000004|c9 02 NC 47
Burkholderia pseudomallei MSHR4503|JPQN01000004|c10 02 NC 47
Burkholderia pseudomallei MSHR3960|JPVJ01000009|c6 02 NC 47
Burkholderia pseudomallei|CSSV01000021|c1 02 NC 47
Burkholderia pseudomallei MSHR2243|CP009269|c6 02 NC 47
Burkholderia pseudomallei TSV44|JQGX01000063|c1 02 NC 47

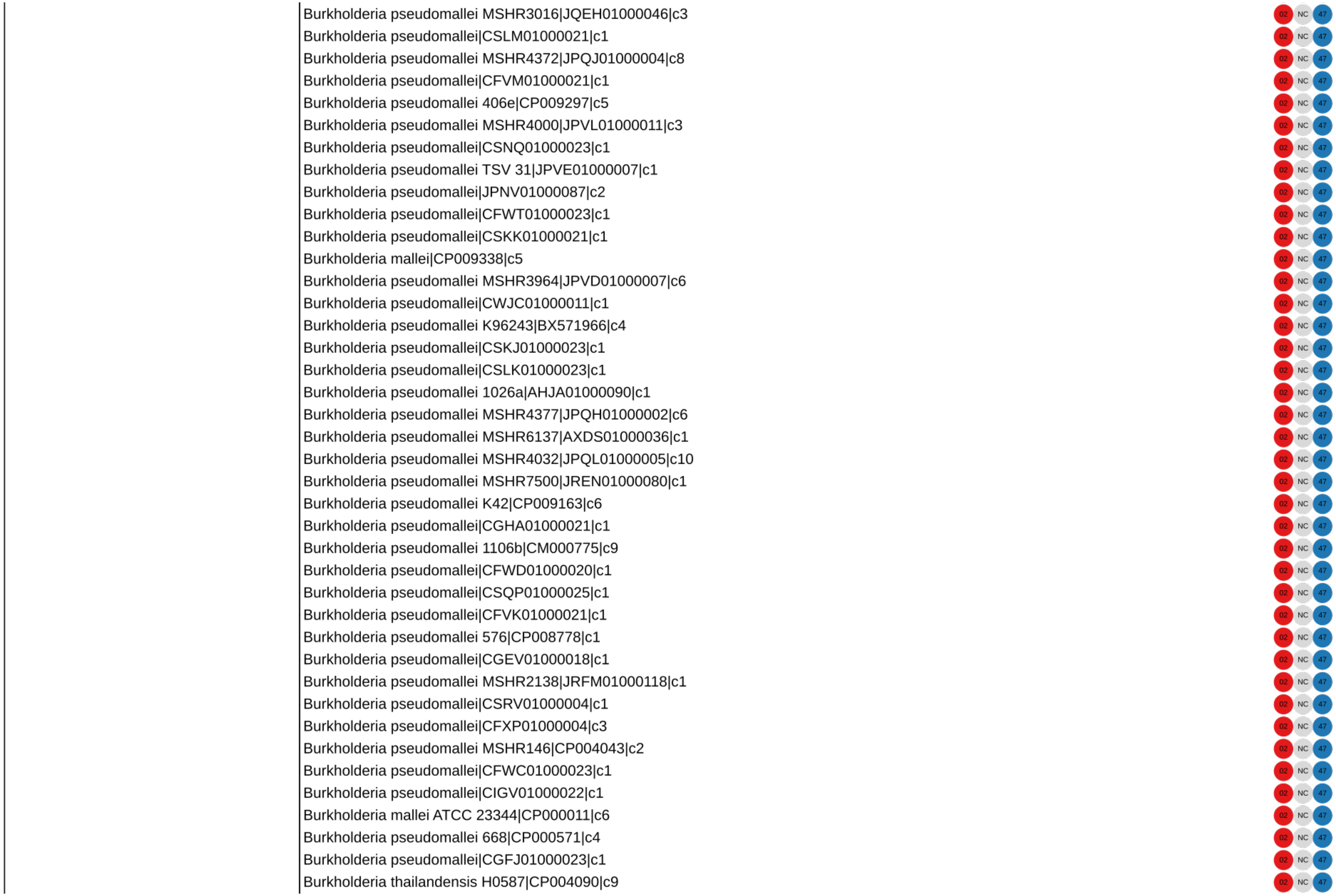
Burkholderia pseudomallei MSHR3016|JQEH01000046|c3
Burkholderia pseudomallei|CSLM01000021|c1
Burkholderia pseudomallei MSHR4372|JPQJ01000004|c8
Burkholderia pseudomallei|CFVM01000021|c1
Burkholderia pseudomallei 406e|CP009297|c5
Burkholderia pseudomallei MSHR4000|JPVL01000011|c3
Burkholderia pseudomallei|CSNQ01000023|c1
Burkholderia pseudomallei TSV 31|JPVE01000007|c1
Burkholderia pseudomallei|JPNV01000087|c2
Burkholderia pseudomallei|CFWT01000023|c1
Burkholderia pseudomallei|CSKK01000021|c1
Burkholderia mallei|CP009338|c5
Burkholderia pseudomallei MSHR3964|JPVD01000007|c6
Burkholderia pseudomallei|CWJC01000011|c1
Burkholderia pseudomallei K96243|BX571966|c4
Burkholderia pseudomallei|CSKJ01000023|c1
Burkholderia pseudomallei|CSLK01000023|c1
Burkholderia pseudomallei 1026a|AHJA01000090|c1
Burkholderia pseudomallei MSHR4377|JPQH01000002|c6
Burkholderia pseudomallei MSHR6137|AXDS01000036|c1
Burkholderia pseudomallei MSHR4032|JPQL01000005|c10
Burkholderia pseudomallei MSHR7500|JREN01000080|c1
Burkholderia pseudomallei K42|CP009163|c6
Burkholderia pseudomallei|CGHA01000021|c1
Burkholderia pseudomallei 1106b|CM000775|c9
Burkholderia pseudomallei|CFWD01000020|c1
Burkholderia pseudomallei|CSQP01000025|c1
Burkholderia pseudomallei|CFVK01000021|c1
Burkholderia pseudomallei 576|CP008778|c1
Burkholderia pseudomallei|CGEV01000018|c1
Burkholderia pseudomallei MSHR2138|JRFM01000118|c1
Burkholderia pseudomallei|CSRV01000004|c1
Burkholderia pseudomallei|CFXP01000004|c3
Burkholderia pseudomallei MSHR146|CP004043|c2
Burkholderia pseudomallei|CFWC01000023|c1
Burkholderia pseudomallei|CIGV01000022|c1
Burkholderia mallei ATCC 23344|CP000011|c6
Burkholderia pseudomallei 668|CP000571|c4
Burkholderia pseudomallei|CGFJ01000023|c1
Burkholderia thailandensis H0587|CP004090|c9
02 NC 47

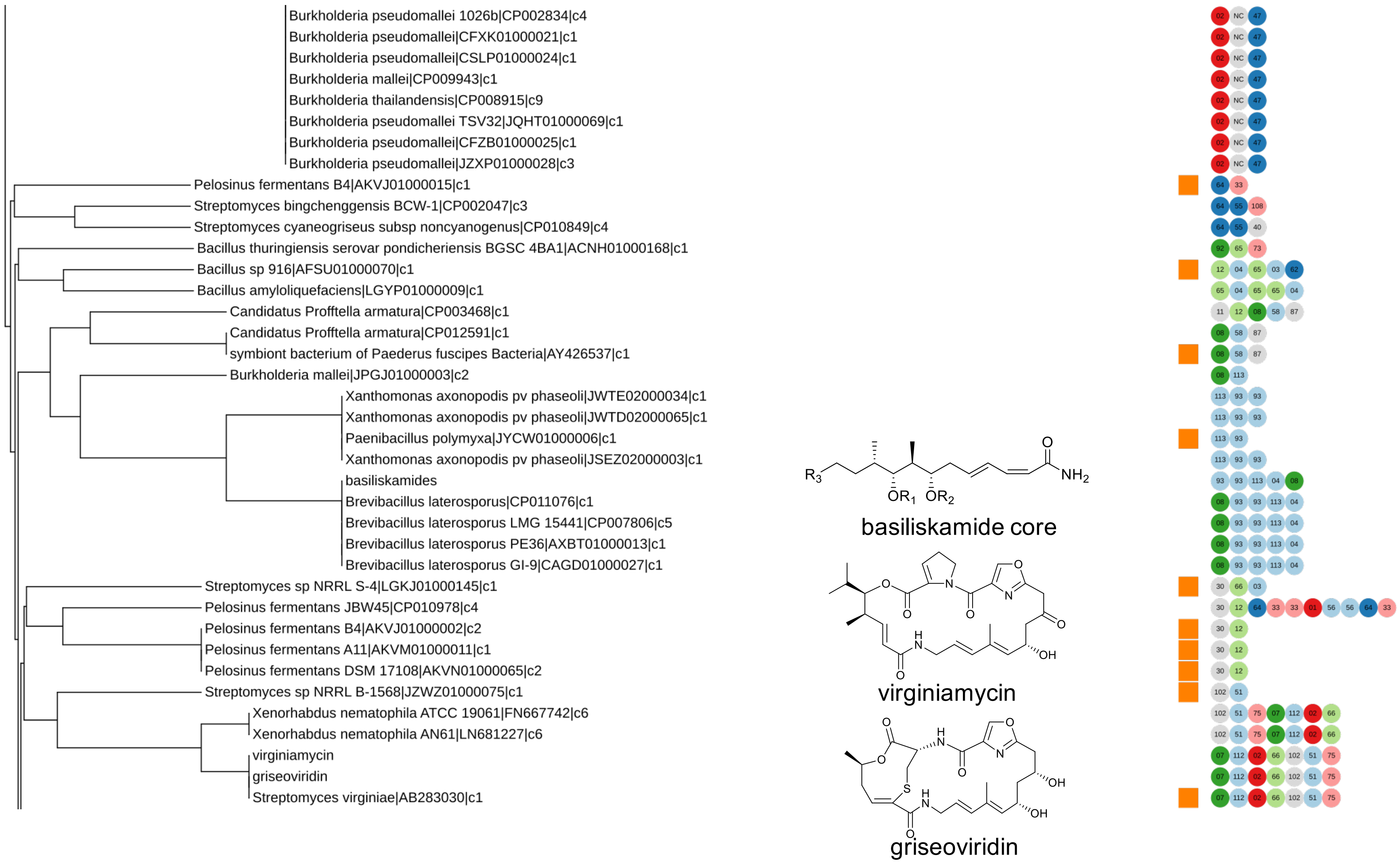
Burkholderia pseudomallei 1026b|CP002834|c4
Burkholderia pseudomallei|CFXK01000021|c1
Burkholderia pseudomallei|CSLP01000024|c1
Burkholderia mallei|CP009943|c1
Burkholderia thailandensis|CP008915|c9
Burkholderia pseudomallei TSV32|JQHT01000069|c1
Burkholderia pseudomallei|CFZB01000025|c1
Burkholderia pseudomallei|JZXP01000028|c3
Pelosinus fermentans B4|AKVJ01000015|c1
Streptomyces bingchenggensis BCW-1|CP002047|c3
Streptomyces cyaneogriseus subsp noncyanogenus|CP010849|c4
Bacillus thuringiensis serovar pondicheriensis BGSC 4BA1|ACNH01000168|c1
Bacillus sp 916|AFSU01000070|c1
Bacillus amyloliquefaciens|LGYP01000009|c1
Candidatus Profftella armatura|CP003468|c1
Candidatus Profftella armatura|CP012591|c1
symbiont bacterium of Paederus fuscipes Bacteria|AY426537|c1
Burkholderia mallei|JPGJ01000003|c2
Xanthomonas axonopodis pv phaseoli|JWTE02000034|c1
Xanthomonas axonopodis pv phaseoli|JWTD02000065|c1
Paenibacillus polymyxa|JYCW01000006|c1
Xanthomonas axonopodis pv phaseoli|JSEZ02000003|c1
basiliskamides
Brevibacillus laterosporus|CP011076|c1
Brevibacillus laterosporus LMG 15441|CP007806|c5
Brevibacillus laterosporus PE36|AXBT01000013|c1
Brevibacillus laterosporus GI-9|CAGD01000027|c1
Streptomyces sp NRRL S-4|LGKJ01000145|c1
Pelosinus fermentans JBW45|CP010978|c4
Pelosinus fermentans B4|AKVJ01000002|c2
Pelosinus fermentans A11|AKVM01000011|c1
Pelosinus fermentans DSM 17108|AKVN01000065|c2
Streptomyces sp NRRL B-1568|JZWZ01000075|c1
Xenorhabdus nematophila ATCC 19061|FN667742|c6
Xenorhabdus nematophila AN61|LN681227|c6
virginiamycin
griseoviridin
Streptomyces virginiae|AB283030|c1
basiliskamide core
virginiamycin
griseoviridin

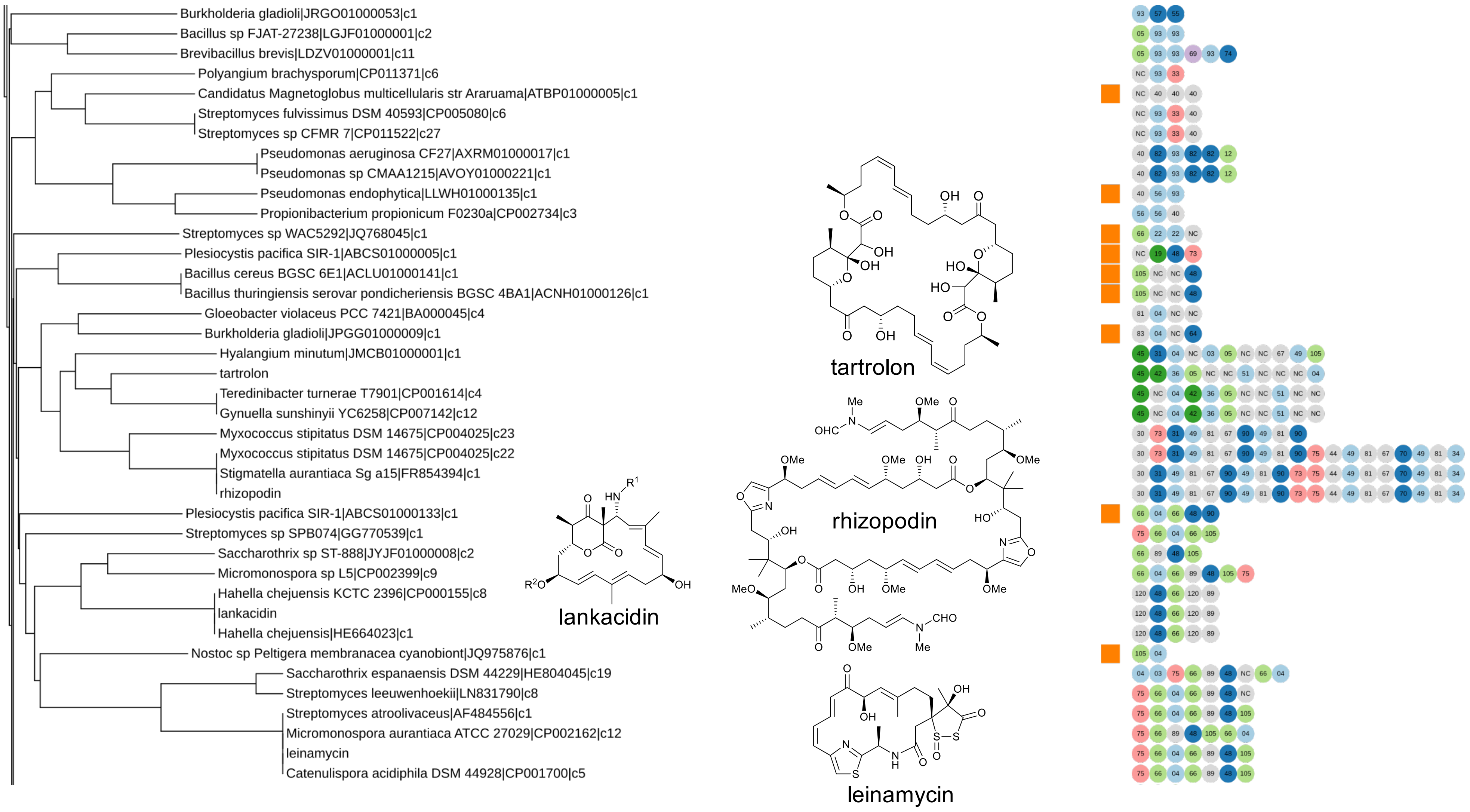

Burkholderia gladioli|JRGO01000053|c1
Bacillus sp FJAT-27238|LGJF01000001|c2
Brevibacillus brevis|LDZV01000001|c11
Polyangium brachysporum|CP011371|c6
Candidatus Magnetoglobus multicellularis str Araruama|ATBP01000005|c1
Streptomyces fulvissimus DSM 40593|CP005080|c6
Streptomyces sp CFMR 7|CP011522|c27
Pseudomonas aeruginosa CF27|AXRM01000017|c1
Pseudomonas sp CMAA1215|AVOY01000221|c1
Pseudomonas endophytica|LLWH01000135|c1
Propionibacterium propionicum F0230a|CP002734|c3
Streptomyces sp WAC5292|JQ768045|c1
Plesiocystis pacifica SIR-1|ABCS01000005|c1
Bacillus cereus BGSC 6E1|ACLU01000141|c1
Bacillus thuringiensis serovar pondicheriensis BGSC 4BA1|ACNH01000126|c1
Gloeobacter violaceus PCC 7421|BA000045|c4
Burkholderia gladioli|JPGG01000009|c1
Hyalangium minutum|JMCB01000001|c1
tartrolon
Teredinibacter turnerae T7901|CP001614|c4
Gynuella sunshinyii YC6258|CP007142|c12
Myxococcus stipitatus DSM 14675|CP004025|c23
Myxococcus stipitatus DSM 14675|CP004025|c22
Stigmatella aurantiaca Sg a15|FR854394|c1
rhizopodin
Plesiocystis pacifica SIR-1|ABCS01000133|c1
Streptomyces sp SPB074|GG770539|c1
Saccharothrix sp ST-888|JYJF01000008|c2
Micromonospora sp L5|CP002399|c9
Hahella chejuensis KCTC 2396|CP000155|c8
lankacidin
Hahella chejuensis|HE664023|c1
Nostoc sp Peltigera membranacea cyanobiont|JQ975876|c1
Saccharothrix espanaensis DSM 44229|HE804045|c19
Streptomyces leeuwenhoekii|LN831790|c8
Streptomyces atroolivaceus|AF484556|c1
Micromonospora aurantiaca ATCC 27029|CP002162|c12
leinamycin
Catenulispora acidiphila DSM 44928|CP001700|c5
tartrolon
rhizopodin
lankacidin
leinamycin

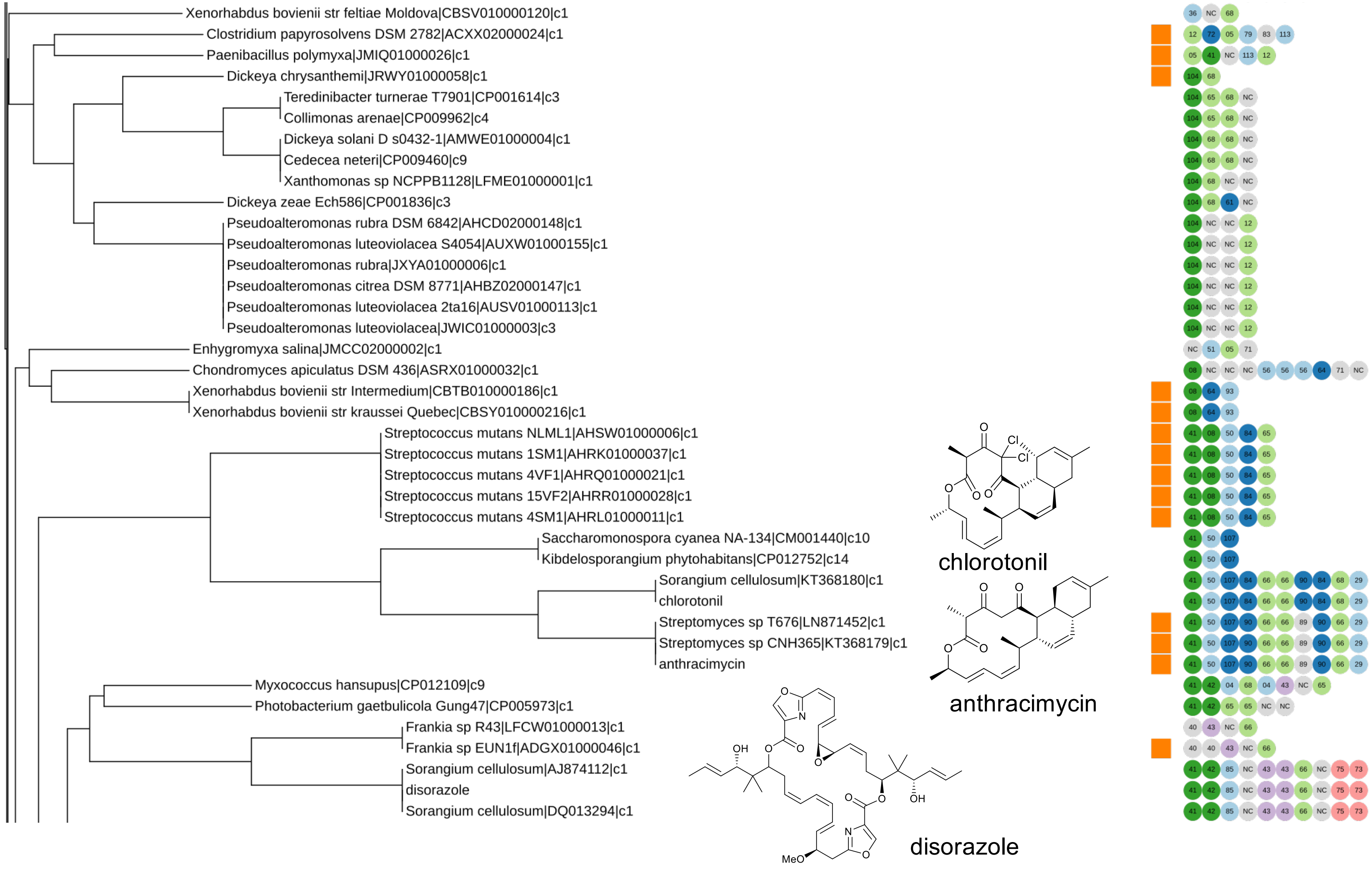
Xenorhabdus bovienii str feltiae Moldova|CBSV010000120|c1
Clostridium papyrosolvens DSM 2782|ACXX02000024|c1
Paenibacillus polymyxa|JMIQ01000026|c1
Dickeya chrysanthemi|JRWY01000058|c1
Teredinibacter turnerae T7901|CP001614|c3
Collimonas arenae|CP009962|c4
Dickeya solani D s0432-1|AMWE01000004|c1
Cedecea neteri|CP009460|c9
Xanthomonas sp NCPPB1128|LFME01000001|c1
Dickeya zeae Ech586|CP001836|c3
Pseudoalteromonas rubra DSM 6842|AHCD02000148|c1
Pseudoalteromonas luteoviolacea S4054|AUXW01000155|c1
Pseudoalteromonas rubra|JXYA01000006|c1
Pseudoalteromonas citrea DSM 8771|AHBZ02000147|c1
Pseudoalteromonas luteoviolacea 2ta16|AUSV01000113|c1
Pseudoalteromonas luteoviolacea|JWIC01000003|c3
Enhygromyxa salina|JMCC02000002|c1
Chondromyces apiculatus DSM 436|ASRX01000032|c1
Xenorhabdus bovienii str Intermedium|CBTB010000186|c1
Xenorhabdus bovienii str kraussei Quebec|CBSY010000216|c1
Streptococcus mutans NLML1|AHSW01000006|c1
Streptococcus mutans 1SM1|AHRK01000037|c1
Streptococcus mutans 4VF1|AHRQ01000021|c1
Streptococcus mutans 15VF2|AHRR01000028|c1
Streptococcus mutans 4SM1|AHRL01000011|c1
Saccharomonospora cyanea NA-134|CM001440|c10
Kibdelosporangium phytohabitans|CP012752|c14
Sorangium cellulosum|KT368180|c1
chlorotonil
Streptomyces sp T676|LN871452|c1
Streptomyces sp CNH365|KT368179|c1
anthracimycin
Myxococcus hansupus|CP012109|c9
Photobacterium gaetbulicola Gung47|CP005973|c1
Frankia sp R43|LFCW01000013|c1
Frankia sp EUN1f|ADGX01000046|c1
Sorangium cellulosum|AJ874112|c1
disorazole
Sorangium cellulosum|DQ013294|c1
chlorotonil
anthracimycin
disorazole

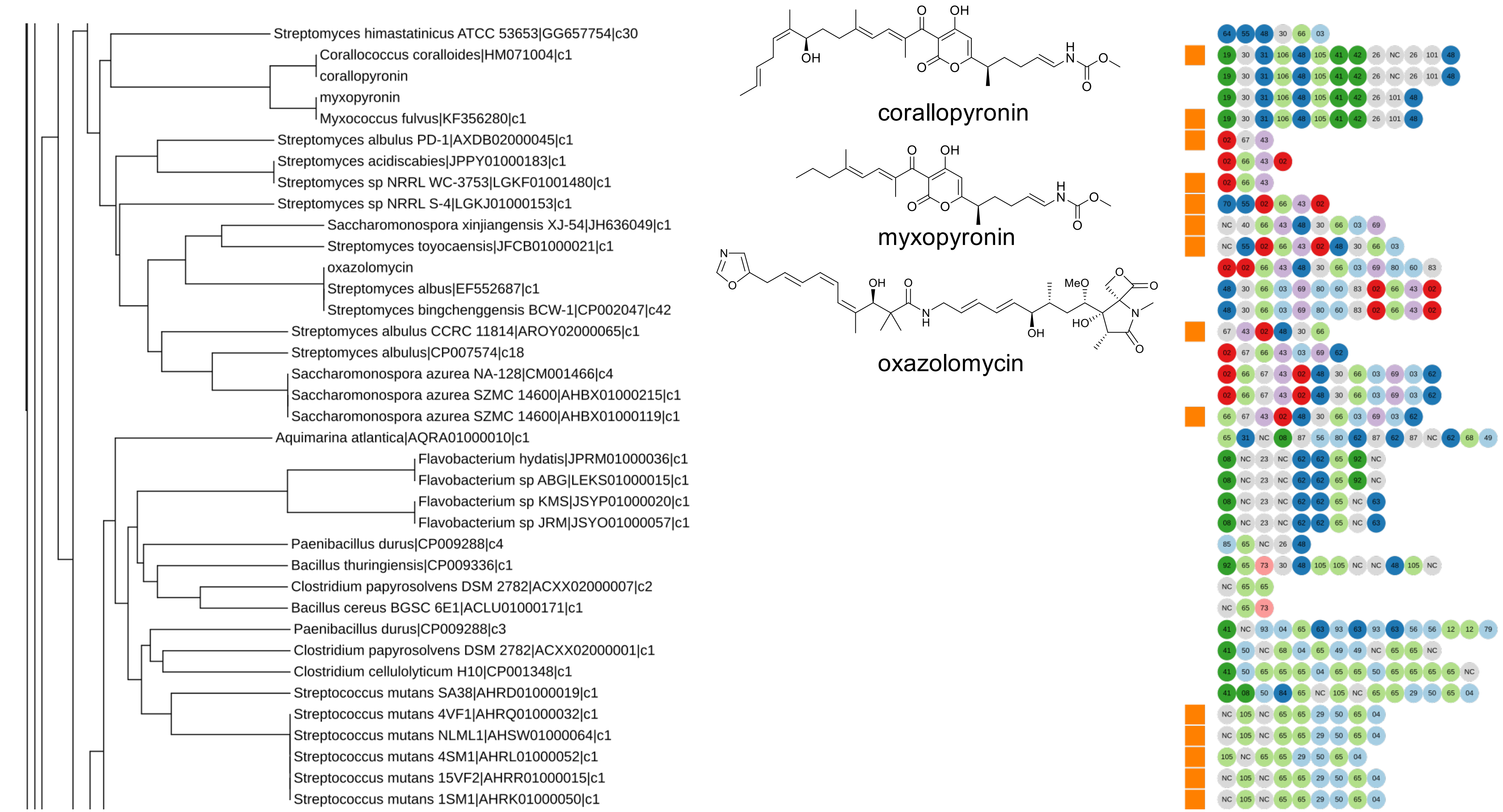
Streptomyces himastatinicus ATCC 53653|GG657754|c30
Corallococcus coralloides|HM071004|c1
corallopyronin
myxopyronin
Myxococcus fulvus|KF356280|c1
Streptomyces albulus PD-1|AXDB02000045|c1
Streptomyces acidiscabies|JPPY01000183|c1
Streptomyces sp NRRL WC-3753|LGKF01001480|c1
Streptomyces sp NRRL S-4|LGKJ01000153|c1
Saccharomonospora xinjiangensis XJ-54|JH636049|c1
Streptomyces toyocaensis|JFCB01000021|c1
oxazolomycin
Streptomyces albus|EF552687|c1
Streptomyces bingchenggensis BCW-1|CP002047|c42
Streptomyces albulus CCRC 11814|AROY02000065|c1
Streptomyces albulus|CP007574|c18
Saccharomonospora azurea NA-128|CM001466|c4
Saccharomonospora azurea SZMC 14600|AHBX01000215|c1
Saccharomonospora azurea SZMC 14600|AHBX01000119|c1
Aquimarina atlantica|AQRA01000010|c1
Flavobacterium hydatis|JPRM01000036|c1
Flavobacterium sp ABG|LEKS01000015|c1
Flavobacterium sp KMS|JSYP01000020|c1
Flavobacterium sp JRM|JSYO01000057|c1
Paenibacillus durus|CP009288|c4
Bacillus thuringiensis|CP009336|c1
Clostridium papyrosolvens DSM 2782|ACXX02000007|c2
Bacillus cereus BGSC 6E1|ACLU01000171|c1
Paenibacillus durus|CP009288|c3
Clostridium papyrosolvens DSM 2782|ACXX02000001|c1
Clostridium cellulolyticum H10|CP001348|c1
Streptococcus mutans SA38|AHRD01000019|c1
Streptococcus mutans 4VF1|AHRQ01000032|c1
Streptococcus mutans NLML1|AHSW01000064|c1
Streptococcus mutans 4SM1|AHRL01000052|c1
Streptococcus mutans 15VF2|AHRR01000015|c1
Streptococcus mutans 1SM1|AHRK01000050|c1
corallopyronin
myxopyronin
oxazolomycin

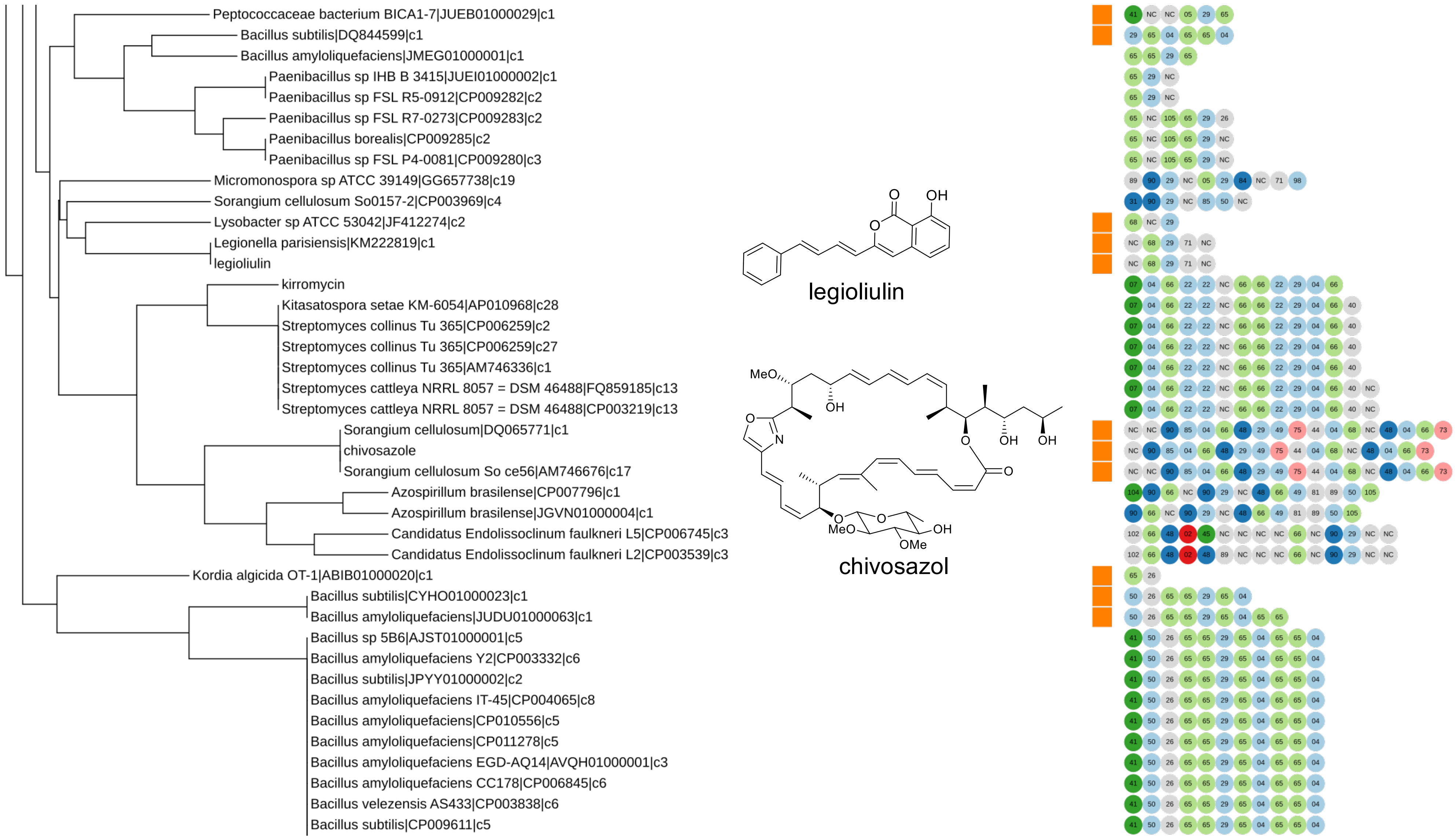

Peptococcaceae bacterium BICA1-7|JUEB01000029|c1
Bacillus subtilis|DQ844599|c1
Bacillus amyloliquefaciens|JMEG01000001|c1
Paenibacillus sp IHB B 3415|JUEI01000002|c1
Paenibacillus sp FSL R5-0912|CP009282|c2
Paenibacillus sp FSL R7-0273|CP009283|c2
Paenibacillus borealis|CP009285|c2
Paenibacillus sp FSL P4-0081|CP009280|c3
Micromonospora sp ATCC 39149|GG657738|c19
Sorangium cellulosum So0157-2|CP003969|c4
Lysobacter sp ATCC 53042|JF412274|c2
Legionella parisiensis|KM222819|c1
legioliulin
kirromycin
Kitasatospora setae KM-6054|AP010968|c28
Streptomyces collinus Tu 365|CP006259|c2
Streptomyces collinus Tu 365|CP006259|c27
Streptomyces collinus Tu 365|AM746336|c1
Streptomyces cattleya NRRL 8057 = DSM 46488|FQ859185|c13
Streptomyces cattleya NRRL 8057 = DSM 46488|CP003219|c13
Sorangium cellulosum|DQ065771|c1
chivosazole
Sorangium cellulosum So ce56|AM746676|c17
Azospirillum brasilense|CP007796|c1
Azospirillum brasilense|JGVN01000004|c1
Candidatus Endolissoclinum faulkneri L5|CP006745|c3
Candidatus Endolissoclinum faulkneri L2|CP003539|c3
Kordia algicida OT-1|ABIB01000020|c1
Bacillus subtilis|CYHO01000023|c1
Bacillus amyloliquefaciens|JUDU01000063|c1
Bacillus sp 5B6|AJST01000001|c5
Bacillus amyloliquefaciens Y2|CP003332|c6
Bacillus subtilis|JPYY01000002|c2
Bacillus amyloliquefaciens IT-45|CP004065|c8
Bacillus amyloliquefaciens|CP010556|c5
Bacillus amyloliquefaciens|CP011278|c5
Bacillus amyloliquefaciens EGD-AQ14|AVQH01000001|c3
Bacillus amyloliquefaciens CC178|CP006845|c6
Bacillus velezensis AS433|CP003838|c6
Bacillus subtilis|CP009611|c5
legioliulin
chivosazol

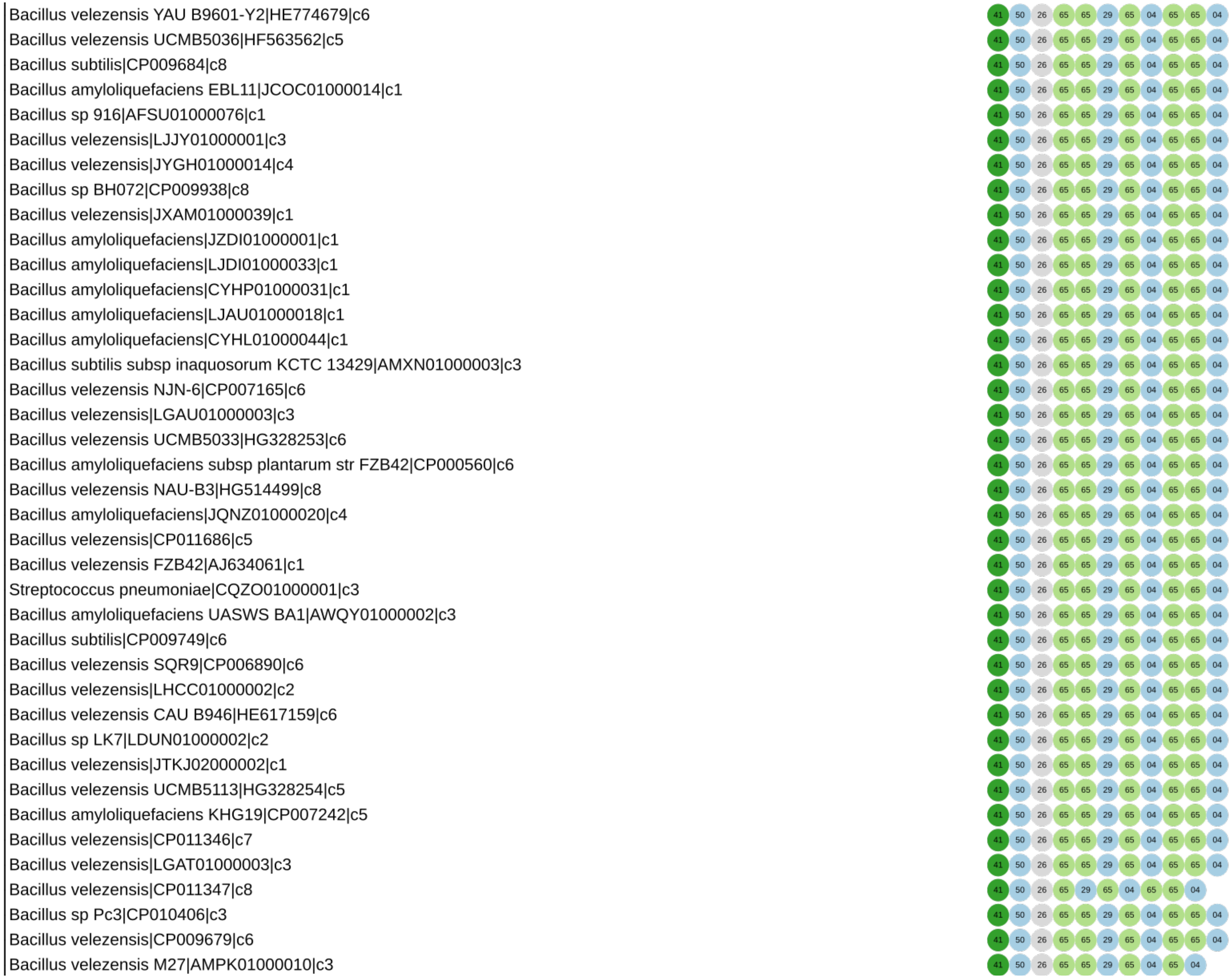
Bacillus velezensis YAU B9601-Y2|HE774679|c6
Bacillus velezensis UCMB5036|HF563562|c5
Bacillus subtilis|CP009684|c8
Bacillus amyloliquefaciens EBL11|JCOC01000014|c1
Bacillus sp 916|AFSU01000076|c1
Bacillus velezensis|LJJY01000001|c3
Bacillus velezensis|JYGH01000014|c4
Bacillus sp BH072|CP009938|c8
Bacillus velezensis|JXAM01000039|c1
Bacillus amyloliquefaciens|JZDI01000001|c1
Bacillus amyloliquefaciens|LJDI01000033|c1
Bacillus amyloliquefaciens|CYHP01000031|c1
Bacillus amyloliquefaciens|LJAU01000018|c1
Bacillus amyloliquefaciens|CYHL01000044|c1
Bacillus subtilis subsp inaquosorum KCTC 13429|AMXN01000003|c3
Bacillus velezensis NJN-6|CP007165|c6
Bacillus velezensis|LGAU01000003|c3
Bacillus velezensis UCMB5033|HG328253|c6
Bacillus amyloliquefaciens subsp plantarum str FZB42|CP000560|c6
Bacillus velezensis NAU-B3|HG514499|c8
Bacillus amyloliquefaciens|JQNZ01000020|c4
Bacillus velezensis|CP011686|c5
Bacillus velezensis FZB42|AJ634061|c1
Streptococcus pneumoniae|CQZO01000001|c3
Bacillus amyloliquefaciens UASWS BA1|AWQY01000002|c3
Bacillus subtilis|CP009749|c6
Bacillus velezensis SQR9|CP006890|c6
Bacillus velezensis|LHCC01000002|c2
Bacillus velezensis CAU B946|HE617159|c6
Bacillus sp LK7|LDUN01000002|c2
Bacillus velezensis|JTKJ02000002|c1
Bacillus velezensis UCMB5113|HG328254|c5
Bacillus amyloliquefaciens KHG19|CP007242|c5
Bacillus velezensis|CP011346|c7
Bacillus velezensis|LGAT01000003|c3
Bacillus velezensis|CP011347|c8
Bacillus sp Pc3|CP010406|c3
Bacillus velezensis|CP009679|c6
Bacillus velezensis M27|AMPK01000010|c3

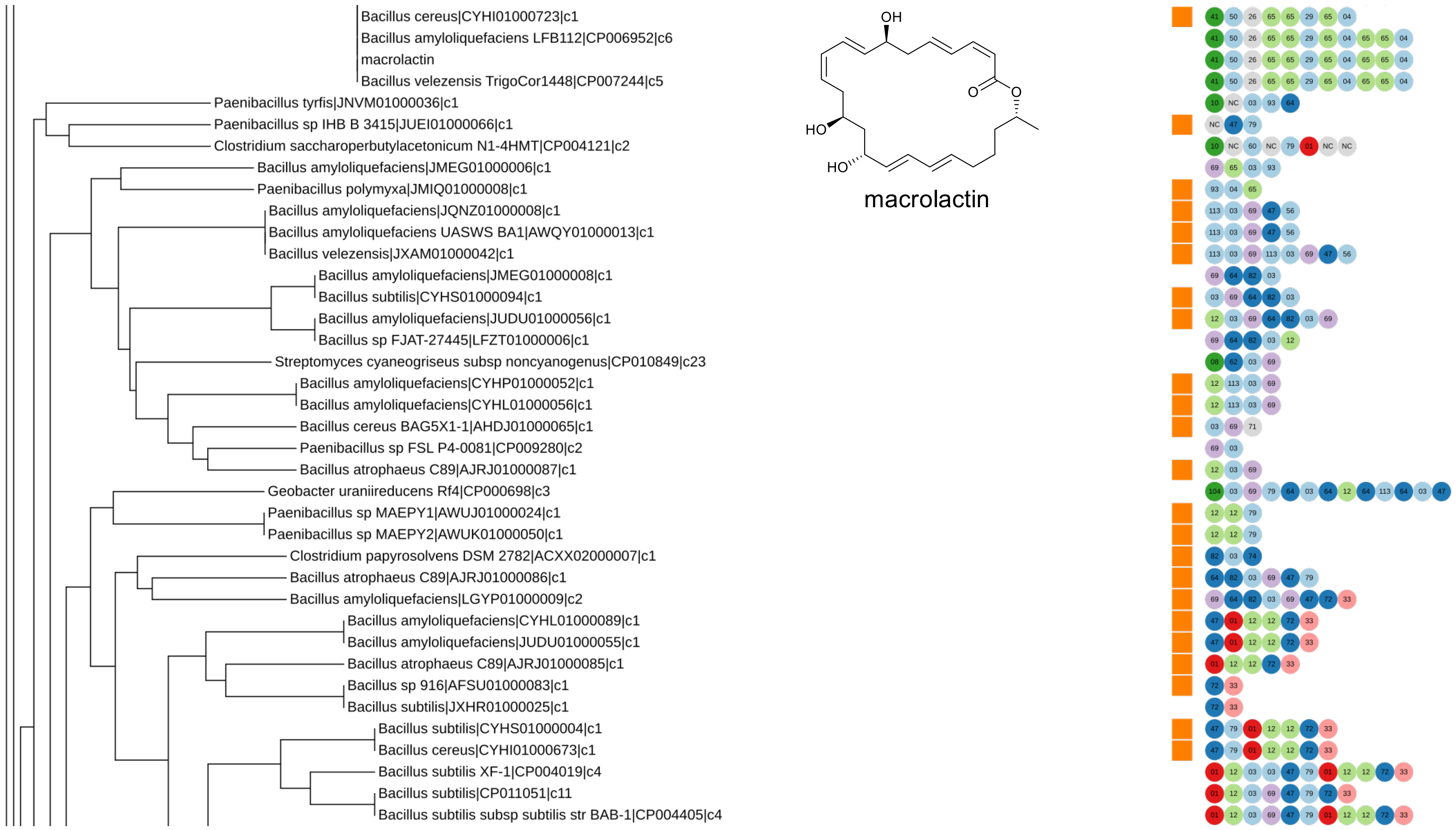
Bacillus cereus|CYHI01000723|c1
Bacillus amyloliquefaciens LFB112|CP006952|c6
macrolactin
Bacillus velezensis TrigoCor1448|CP007244|c5
Paenibacillus tyrfis|JNVM01000036|c1
Paenibacillus sp IHB B 3415|JUEI01000066|c1
Clostridium saccharoperbutylacetonicum N1-4HMT|CP004121|c2
Bacillus amyloliquefaciens|JMEG01000006|c1
Paenibacillus polymyxa|JMIQ01000008|c1
Bacillus amyloliquefaciens|JQNZ01000008|c1
Bacillus amyloliquefaciens UASWS BA1|AWQY01000013|c1
Bacillus velezensis|JXAM01000042|c1
Bacillus amyloliquefaciens|JMEG01000008|c1
Bacillus subtilis|CYHS01000094|c1
Bacillus amyloliquefaciens|JUDU01000056|c1
Bacillus sp FJAT-27445|LFZT01000006|c1
Streptomyces cyaneogriseus subsp noncyanogenus|CP010849|c23
Bacillus amyloliquefaciens|CYHP01000052|c1
Bacillus amyloliquefaciens|CYHL01000056|c1
Bacillus cereus BAG5X1-1|AHDJ01000065|c1
Paenibacillus sp FSL P4-0081|CP009280|c2
Bacillus atrophaeus C89|AJRJ01000087|c1
Geobacter uraniireducens Rf4|CP000698|c3
Paenibacillus sp MAEPY1|AWUJ01000024|c1
Paenibacillus sp MAEPY2|AWUK01000050|c1
Clostridium papyrosolvens DSM 2782|ACXX02000007|c1
Bacillus atrophaeus C89|AJRJ01000086|c1
Bacillus amyloliquefaciens|LGYP01000009|c2
Bacillus amyloliquefaciens|CYHL01000089|c1
Bacillus amyloliquefaciens|JUDU01000055|c1
Bacillus atrophaeus C89|AJRJ01000085|c1
Bacillus sp 916|AFSU01000083|c1
Bacillus subtilis|JXHR01000025|c1
Bacillus subtilis|CYHS01000004|c1
Bacillus cereus|CYHI01000673|c1
Bacillus subtilis XF-1|CP004019|c4
Bacillus subtilis|CP011051|c11
Bacillus subtilis subsp subtilis str BAB-1|CP004405|c4
OH
O
O
HO
HO
macrolactin

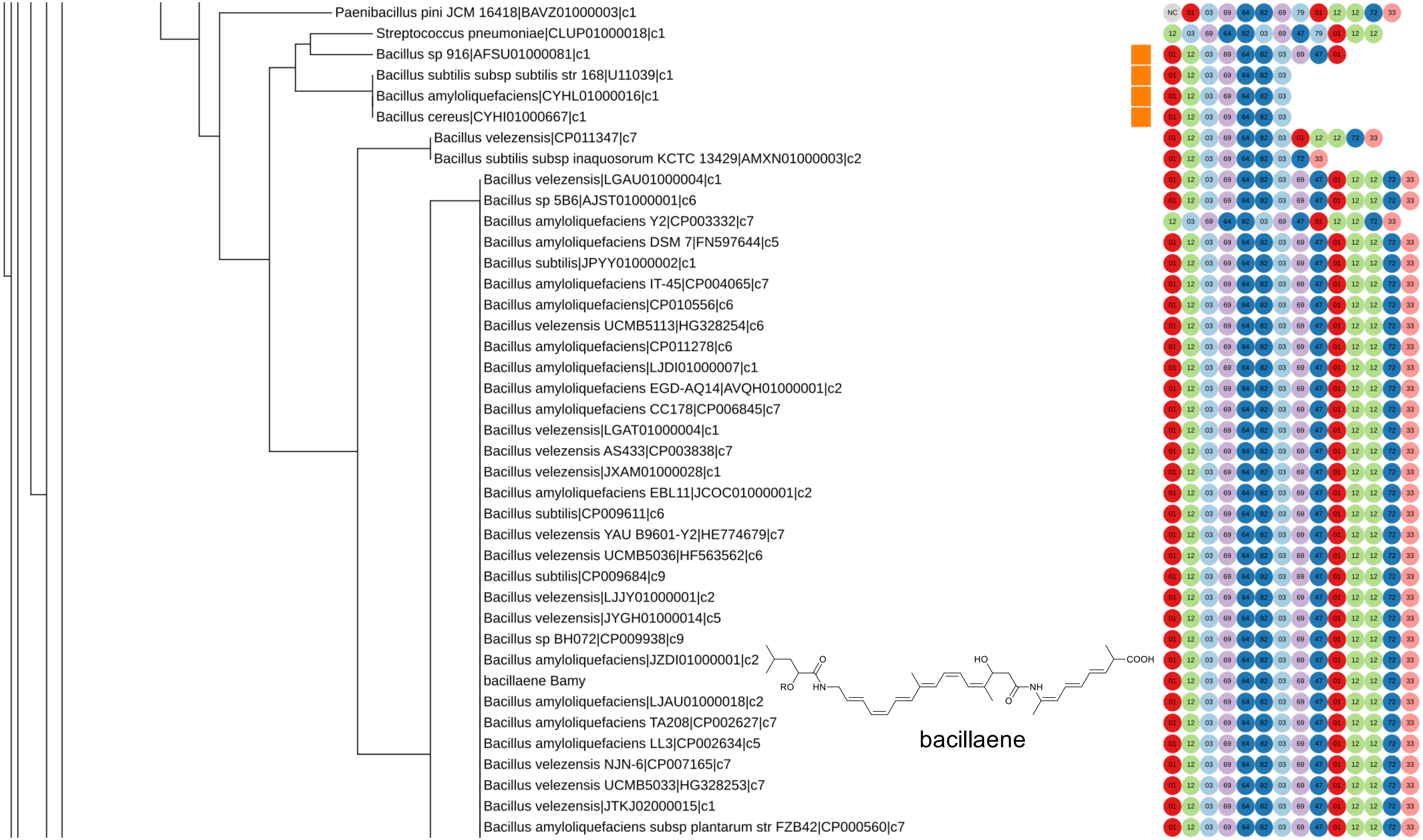
Paenibacillus pini JCM 16418|BAVZ01000003|c1
Streptococcus pneumoniae|CLUP01000018|c1
Bacillus sp 916|AFSU01000081|c1
Bacillus subtilis subsp subtilis str 168|U11039|c1
Bacillus amyloliquefaciens|CYHL01000016|c1
Bacillus cereus|CYHI01000667|c1
Bacillus velezensis|CP011347|c7
Bacillus subtilis subsp inaquosorum KCTC 13429|AMXN01000003|c2
Bacillus velezensis|LGAU01000004|c1
Bacillus sp 5B6|AJST01000001|c6
Bacillus amyloliquefaciens Y2|CP003332|c7
Bacillus amyloliquefaciens DSM 7|FN597644|c5
Bacillus subtilis|JPYY01000002|c1
Bacillus amyloliquefaciens IT-45|CP004065|c7
Bacillus amyloliquefaciens|CP010556|c6
Bacillus velezensis UCMB5113|HG328254|c6
Bacillus amyloliquefaciens|CP011278|c6
Bacillus amyloliquefaciens|LJDI01000007|c1
Bacillus amyloliquefaciens EGD-AQ14|AVQH01000001|c2
Bacillus amyloliquefaciens CC178|CP006845|c7
Bacillus velezensis|LGAT01000004|c1
Bacillus velezensis AS433|CP003838|c7
Bacillus velezensis|JXAM01000028|c1
Bacillus amyloliquefaciens EBL11|JCOC01000001|c2
Bacillus subtilis|CP009611|c6
Bacillus velezensis YAU B9601-Y2|HE774679|c7
Bacillus velezensis UCMB5036|HF563562|c6
Bacillus subtilis|CP009684|c9
Bacillus velezensis|LJJY01000001|c2
Bacillus velezensis|JYGH01000014|c5
Bacillus sp BH072|CP009938|c9
Bacillus amyloliquefaciens|JZDI01000001|c2
bacillaene Bamy
Bacillus amyloliquefaciens|LJAU01000018|c2
Bacillus amyloliquefaciens TA208|CP002627|c7
Bacillus amyloliquefaciens LL3|CP002634|c5
Bacillus velezensis NJN-6|CP007165|c7
Bacillus velezensis UCMB5033|HG328253|c7
Bacillus velezensis|JTKJ02000015|c1
Bacillus amyloliquefaciens subsp plantarum str FZB42|CP000560|c7
RO
HN
O
HO
NH
COOH
bacillaene

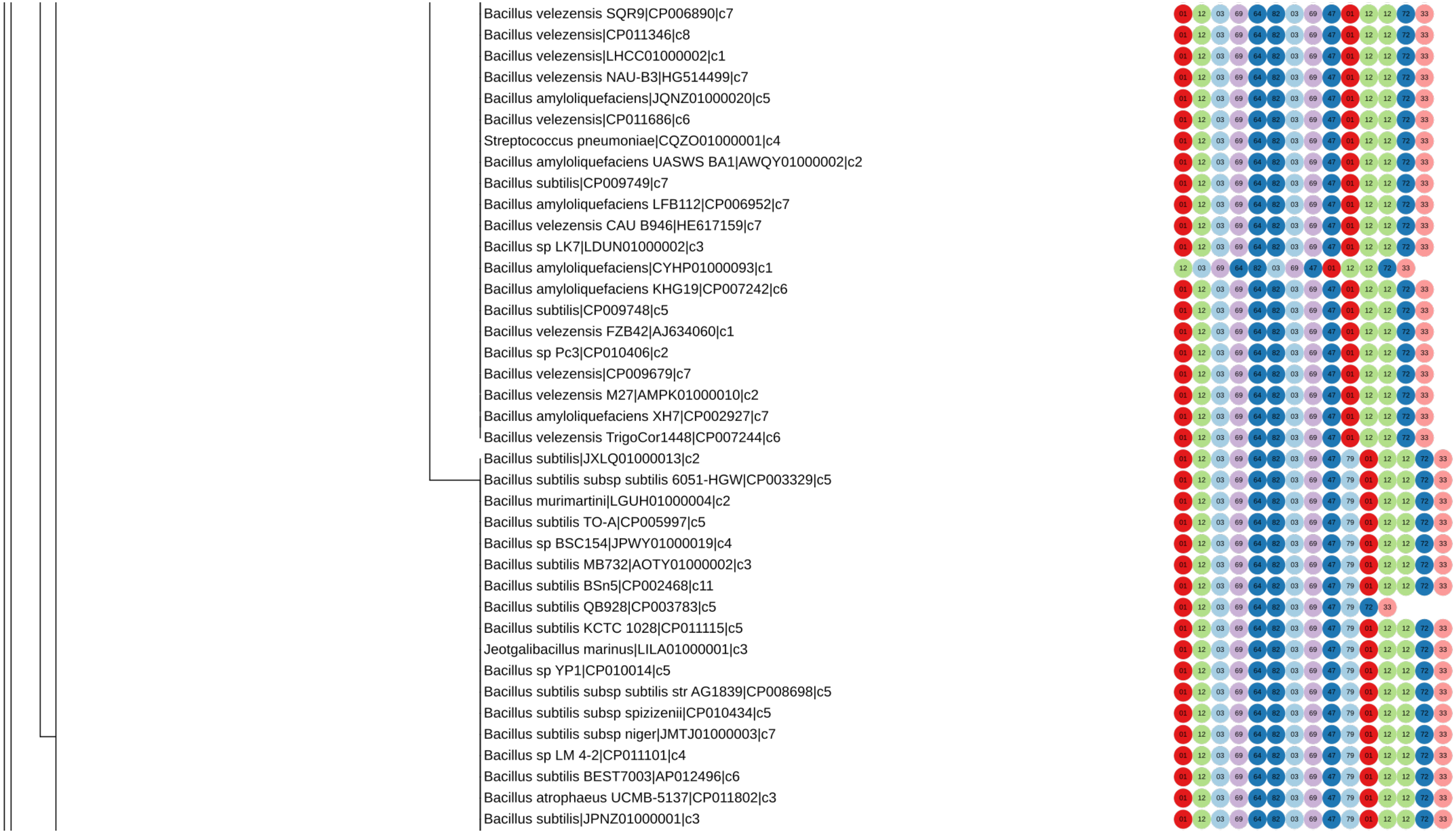
Bacillus velezensis SQR9|CP006890|c7
Bacillus velezensis|CP011346|c8
Bacillus velezensis|LHCC01000002|c1
Bacillus velezensis NAU-B3|HG514499|c7
Bacillus amyloliquefaciens|JQNZ01000020|c5
Bacillus velezensis|CP011686|c6
Streptococcus pneumoniae|CQZO01000001|c4
Bacillus amyloliquefaciens UASWS BA1|AWQY01000002|c2
Bacillus subtilis|CP009749|c7
Bacillus amyloliquefaciens LFB112|CP006952|c7
Bacillus velezensis CAU B946|HE617159|c7
Bacillus sp LK7|LDUN01000002|c3
Bacillus amyloliquefaciens|CYHP01000093|c1
Bacillus amyloliquefaciens KHG19|CP007242|c6
Bacillus subtilis|CP009748|c5
Bacillus velezensis FZB42|AJ634060|c1
Bacillus sp Pc3|CP010406|c2
Bacillus velezensis|CP009679|c7
Bacillus velezensis M27|AMPK01000010|c2
Bacillus amyloliquefaciens XH7|CP002927|c7
Bacillus velezensis TrigoCor1448|CP007244|c6
Bacillus subtilis|JXLQ01000013|c2
Bacillus subtilis subsp subtilis 6051-HGW|CP003329|c5
Bacillus murimartini|LGUH01000004|c2
Bacillus subtilis TO-A|CP005997|c5
Bacillus sp BSC154|JPWY01000019|c4
Bacillus subtilis MB732|AOTY01000002|c3
Bacillus subtilis BSn5|CP002468|c11
Bacillus subtilis QB928|CP003783|c5
Bacillus subtilis KCTC 1028|CP011115|c5
Jeotgalibacillus marinus|LILA01000001|c3
Bacillus sp YP1|CP010014|c5
Bacillus subtilis subsp subtilis str AG1839|CP008698|c5
Bacillus subtilis subsp spizizenii|CP010434|c5
Bacillus subtilis subsp niger|JMTJ01000003|c7
Bacillus sp LM 4-2|CP011101|c4
Bacillus subtilis BEST7003|AP012496|c6
Bacillus atrophaeus UCMB-5137|CP011802|c3
Bacillus subtilis|JPNZ01000001|c3

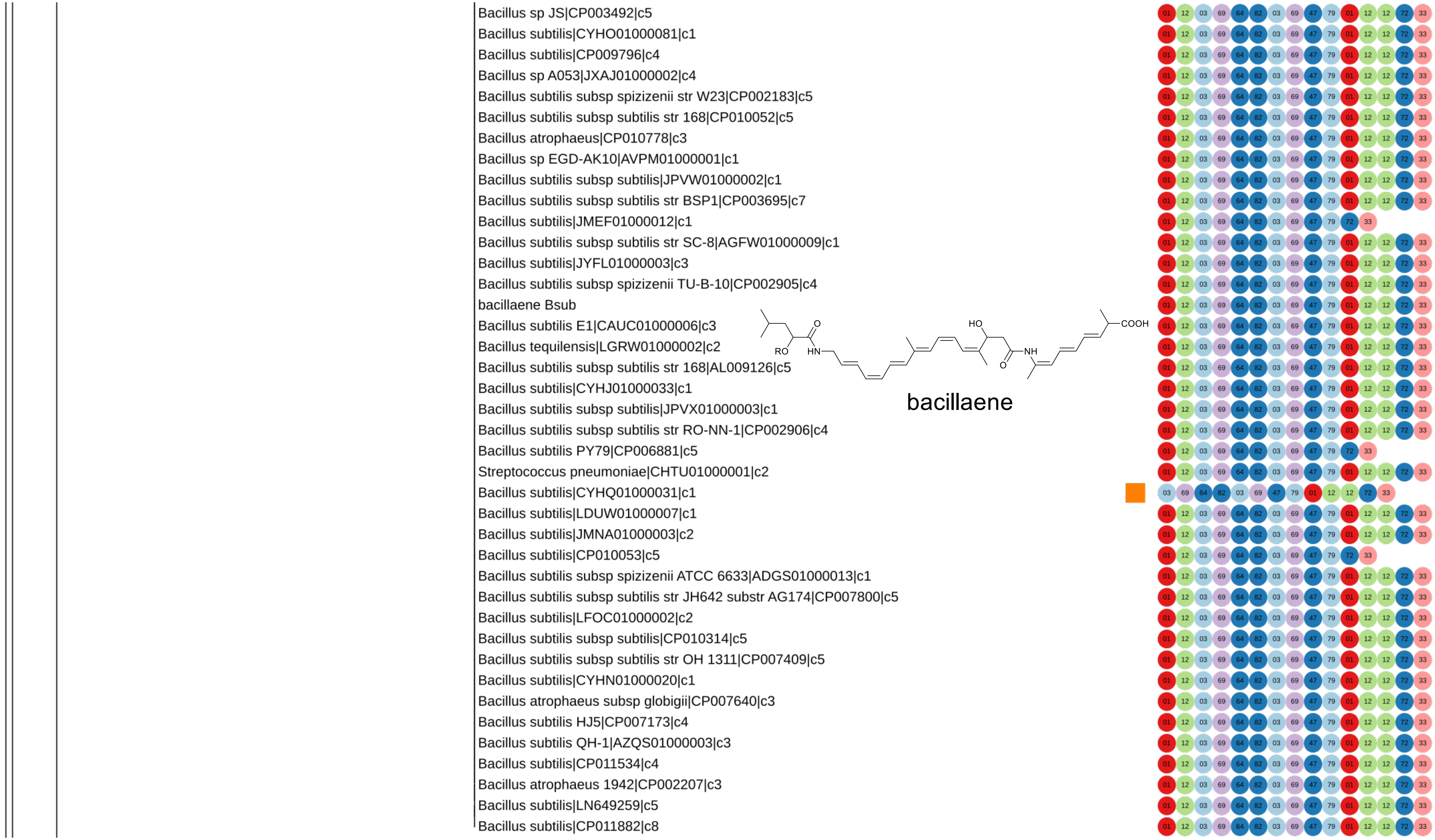
Bacillus sp JS|CP003492|c5
Bacillus subtilis|CYHO01000081|c1
Bacillus subtilis|CP009796|c4
Bacillus sp A053|JXAJ01000002|c4
Bacillus subtilis subsp spizizenii str W23|CP002183|c5
Bacillus subtilis subsp subtilis str 168|CP010052|c5
Bacillus atrophaeus|CP010778|c3
Bacillus sp EGD-AK10|AVPM01000001|c1
Bacillus subtilis subsp subtilis|JPVW01000002|c1
Bacillus subtilis subsp subtilis str BSP1|CP003695|c7
Bacillus subtilis|JMEF01000012|c1
Bacillus subtilis subsp subtilis str SC-8|AGFW01000009|c1
Bacillus subtilis|JYFL01000003|c3
Bacillus subtilis subsp spizizenii TU-B-10|CP002905|c4
bacillaene Bsub
Bacillus subtilis E1|CAUC01000006|c3
Bacillus tequilensis|LGRW01000002|c2
Bacillus subtilis subsp subtilis str 168|AL009126|c5
Bacillus subtilis|CYHJ01000033|c1
Bacillus subtilis subsp subtilis|JPVX01000003|c1
Bacillus subtilis subsp subtilis str RO-NN-1|CP002906|c4
Bacillus subtilis PY79|CP006881|c5
Streptococcus pneumoniae|CHTU01000001|c2
Bacillus subtilis|CYHQ01000031|c1
Bacillus subtilis|LDUW01000007|c1
Bacillus subtilis|JMNA01000003|c2
Bacillus subtilis|CP010053|c5
Bacillus subtilis subsp spizizenii ATCC 6633|ADGS01000013|c1
Bacillus subtilis subsp subtilis str JH642 substr AG174|CP007800|c5
Bacillus subtilis|LFOC01000002|c2
Bacillus subtilis subsp subtilis|CP010314|c5
Bacillus subtilis subsp subtilis str OH 1311|CP007409|c5
Bacillus subtilis|CYHN01000020|c1
Bacillus atrophaeus subsp globigii|CP007640|c3
Bacillus subtilis HJ5|CP007173|c4
Bacillus subtilis QH-1|AZQS01000003|c3
Bacillus subtilis|CP011534|c4
Bacillus atrophaeus 1942|CP002207|c3
Bacillus subtilis|LN649259|c5
Bacillus subtilis|CP011882|c8
RO
HN
O
HO
NH
O
COOH
bacillaene

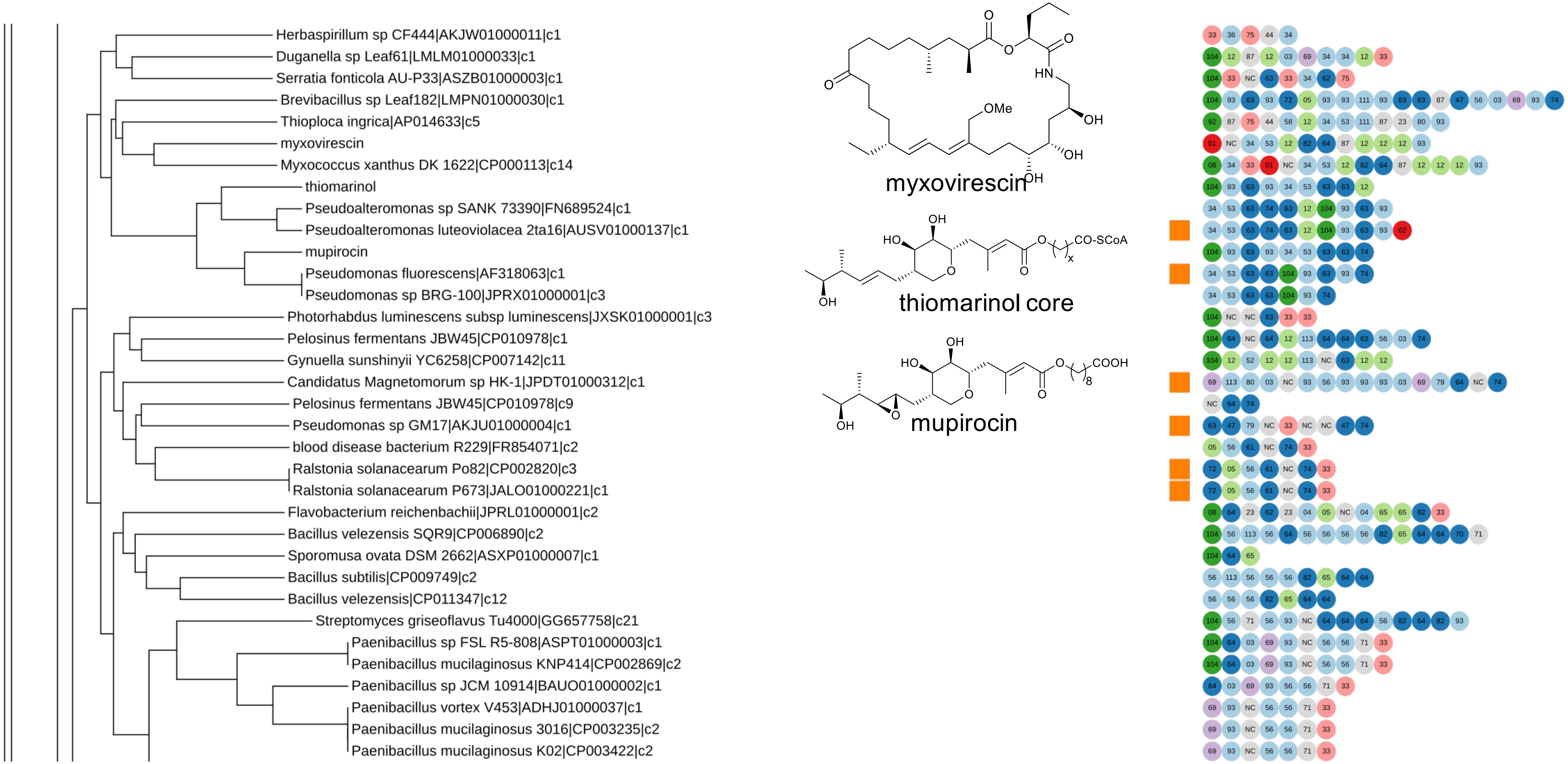
Herbaspirillum sp CF444|AKJW01000011|c1
Duganella sp Leaf61|LMLM01000033|c1
Serratia fonticola AU-P33|ASZB01000003|c1
Brevibacillus sp Leaf182|LMPN01000030|c1
Thioploca ingrica|AP014633|c5
myxovirescin
Myxococcus xanthus DK 1622|CP000113|c14
thiomarinol
Pseudoalteromonas sp SANK 73390|FN689524|c1
Pseudoalteromonas luteoviolacea 2ta16|AUSV01000137|c1
mupirocin
Pseudomonas fluorescens|AF318063|c1
Pseudomonas sp BRG-100|JPRX01000001|c3
Photorhabdus luminescens subsp luminescens|JXSK01000001|c3
Pelosinus fermentans JBW45|CP010978|c1
Gynuella sunshinyii YC6258|CP007142|c11
Candidatus Magnetomorum sp HK-1|JPDT01000312|c1
Pelosinus fermentans JBW45|CP010978|c9
Pseudomonas sp GM17|AKJU01000004|c1
blood disease bacterium R229|FR854071|c2
Ralstonia solanacearum Po82|CP002820|c3
Ralstonia solanacearum P673|JALO01000221|c1
Flavobacterium reichenbachii|JPRL01000001|c2
Bacillus velezensis SQR9|CP006890|c2
Sporomusa ovata DSM 2662|ASXP01000007|c1
Bacillus subtilis|CP009749|c2
Bacillus velezensis|CP011347|c12
Streptomyces griseoflavus Tu4000|GG657758|c21
Paenibacillus sp FSL R5-808|ASPT01000003|c1
Paenibacillus mucilaginosus KNP414|CP002869|c2
Paenibacillus sp JCM 10914|BAUO01000002|c1
Paenibacillus vortex V453|ADHJ01000037|c1
Paenibacillus mucilaginosus 3016|CP003235|c2
Paenibacillus mucilaginosus K02|CP003422|c2
myxovirescin
thiomarinol core
mupirocin

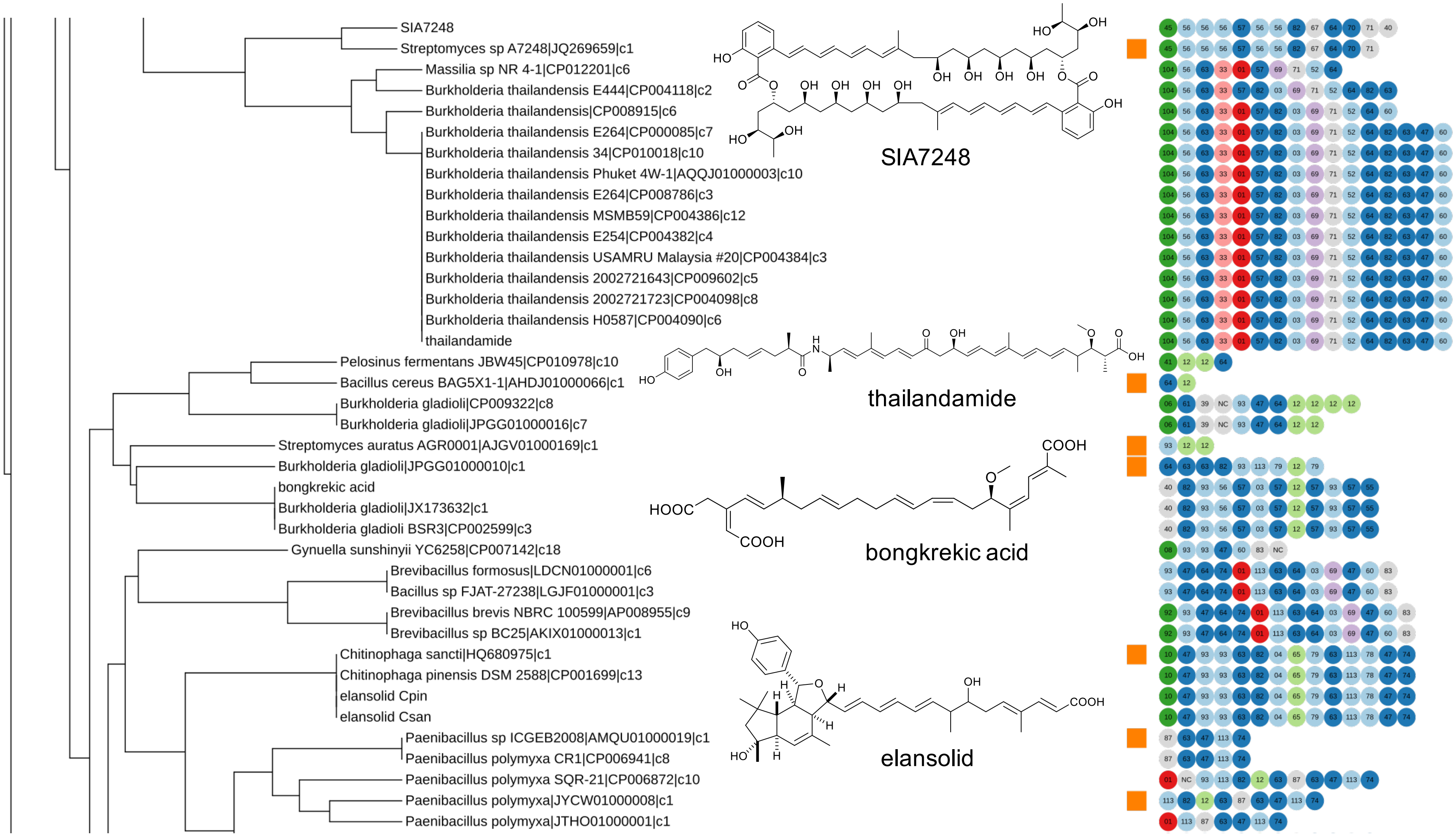

SIA7248
Streptomyces sp A7248|JQ269659|c1
Massilia sp NR 4-1|CP012201|c6
Burkholderia thailandensis E444|CP004118|c2
Burkholderia thailandensis|CP008915|c6
Burkholderia thailandensis E264|CP000085|c7
Burkholderia thailandensis 34|CP010018|c10
Burkholderia thailandensis Phuket 4W-1|AQQJ01000003|c10
Burkholderia thailandensis E264|CP008786|c3
Burkholderia thailandensis MSMB59|CP004386|c12
Burkholderia thailandensis E254|CP004382|c4
Burkholderia thailandensis USAMRU Malaysia #20|CP004384|c3
Burkholderia thailandensis 2002721643|CP009602|c5
Burkholderia thailandensis 2002721723|CP004098|c8
Burkholderia thailandensis H0587|CP004090|c6
thailandamide
Pelosinus fermentans JBW45|CP010978|c10
Bacillus cereus BAG5X1-1|AHDJ01000066|c1
Burkholderia gladioli|CP009322|c8
Burkholderia gladioli|JPGG01000016|c7
Streptomyces auratus AGR0001|AJGV01000169|c1
Burkholderia gladioli|JPGG01000010|c1
bongkrekic acid
Burkholderia gladioli|JX173632|c1
Burkholderia gladioli BSR3|CP002599|c3
Gynuella sunshinyii YC6258|CP007142|c18
Brevibacillus formosus|LDCN01000001|c6
Bacillus sp FJAT-27238|LGJF01000001|c3
Brevibacillus brevis NBRC 100599|AP008955|c9
Brevibacillus sp BC25|AKIX01000013|c1
Chitinophaga sancti|HQ680975|c1
Chitinophaga pinensis DSM 2588|CP001699|c13
elansolid Cpin
elansolid Csan
Paenibacillus sp ICGEB2008|AMQU01000019|c1
Paenibacillus polymyxa CR1|CP006941|c8
Paenibacillus polymyxa SQR-21|CP006872|c10
Paenibacillus polymyxa|JYCW01000008|c1
Paenibacillus polymyxa|JTHO01000001|c1
SIA7248
thailandamide
bongkrekic acid
elansolid

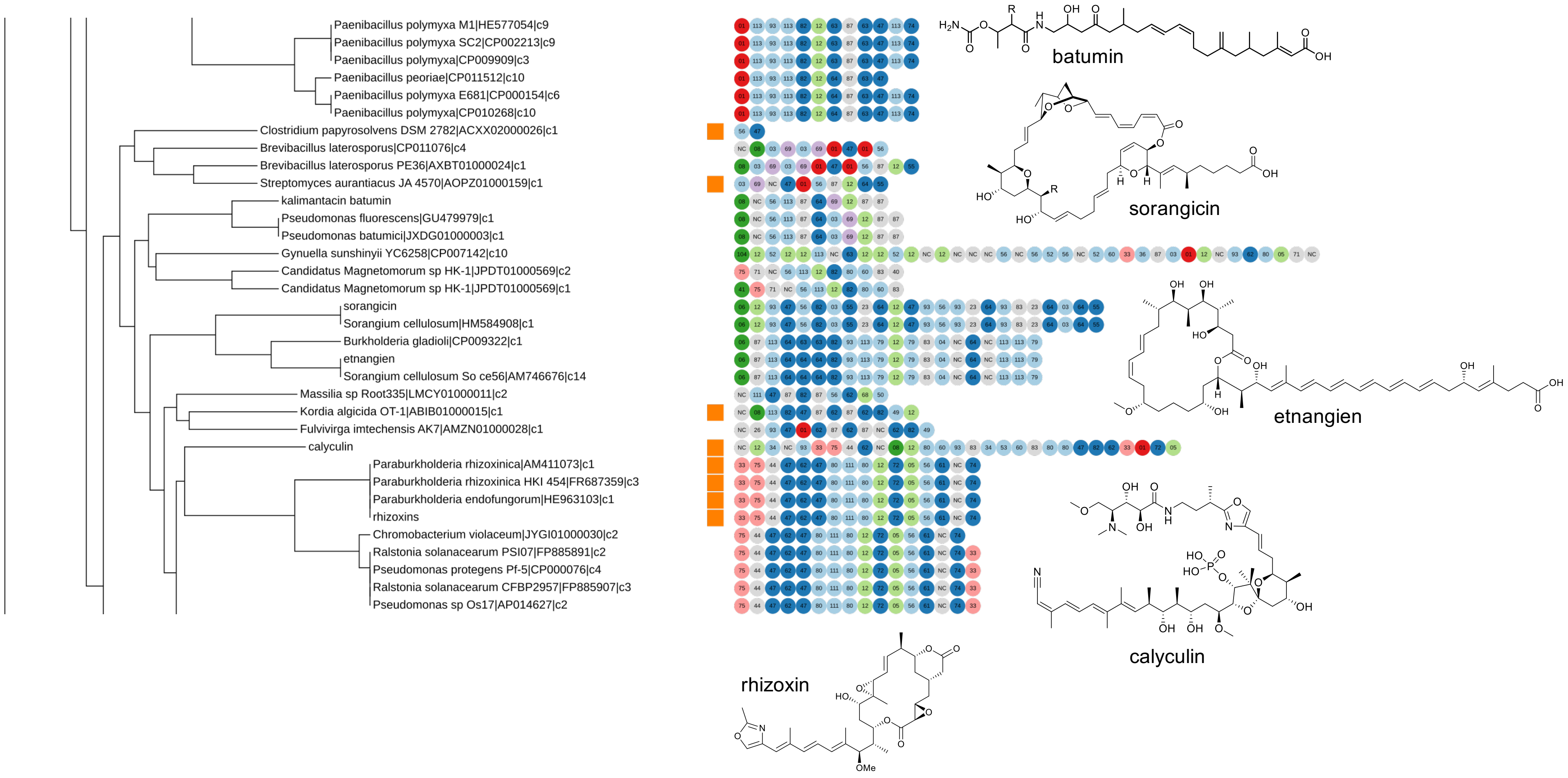
Paenibacillus polymyxa M1|HE577054|c9
Paenibacillus polymyxa SC2|CP002213|c9
Paenibacillus polymyxa|CP009909|c3
Paenibacillus peoriae|CP011512|c10
Paenibacillus polymyxa E681|CP000154|c6
Paenibacillus polymyxa|CP010268|c10
Clostridium papyrosolvens DSM 2782|ACXX02000026|c1
Brevibacillus laterosporus|CP011076|c4
Brevibacillus laterosporus PE36|AXBT01000024|c1
Streptomyces aurantiacus JA 4570|AOPZ01000159|c1
kalimantacin batumin
Pseudomonas fluorescens|GU479979|c1
Pseudomonas batumici|JXDG01000003|c1
Gynuella sunshinyii YC6258|CP007142|c10
Candidatus Magnetomorum sp HK-1|JPDT01000569|c2
Candidatus Magnetomorum sp HK-1|JPDT01000569|c1
sorangicin
Sorangium cellulosum|HM584908|c1
Burkholderia gladioli|CP009322|c1
etnangien
Sorangium cellulosum So ce56|AM746676|c14
Massilia sp Root335|LMCY01000011|c2
Kordia algicida OT-1|ABIB01000015|c1
Fulvivirga imtechensis AK7|AMZN01000028|c1
calyculin
Paraburkholderia rhizoxinica|AM411073|c1
Paraburkholderia rhizoxinica HKI 454|FR687359|c3
Paraburkholderia endofungorum|HE963103|c1
rhizoxins
Chromobacterium violaceum|JYGI01000030|c2
Ralstonia solanacearum PSI07|FP885891|c2
Pseudomonas protegens Pf-5|CP000076|c4
Ralstonia solanacearum CFBP2957|FP885907|c3
Pseudomonas sp Os17|AP014627|c2
batumin
sorangicin
etnangien
calyculin
rhizoxin

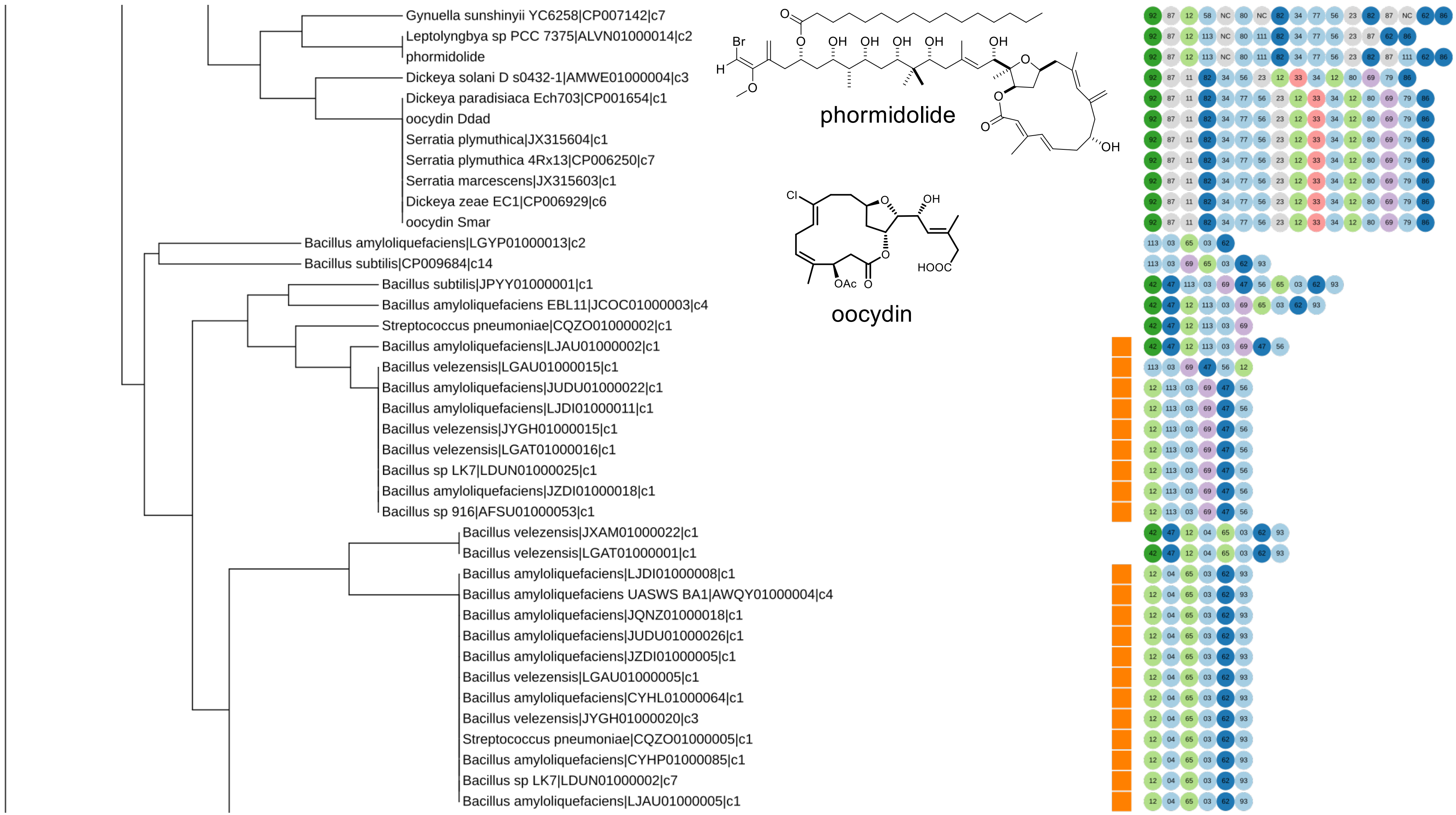
Gynuella sunshinyii YC6258|CP007142|c7
Leptolyngbya sp PCC 7375|ALVN01000014|c2
phormidolide
Dickeya solani D s0432-1|AMWE01000004|c3
Dickeya paradisiaca Ech703|CP001654|c1
oocydin Ddad
Serratia plymuthica|JX315604|c1
Serratia plymuthica 4Rx13|CP006250|c7
Serratia marcescens|JX315603|c1
Dickeya zeae EC1|CP006929|c6
oocydin Smar
Bacillus amyloliquefaciens|LGYP01000013|c2
Bacillus subtilis|CP009684|c14
Bacillus subtilis|JPYY01000001|c1
Bacillus amyloliquefaciens EBL11|JCOC01000003|c4
Streptococcus pneumoniae|CQZO01000002|c1
Bacillus amyloliquefaciens|LJAU01000002|c1
Bacillus velezensis|LGAU01000015|c1
Bacillus amyloliquefaciens|JUDU01000022|c1
Bacillus amyloliquefaciens|LJDI01000011|c1
Bacillus velezensis|JYGH01000015|c1
Bacillus velezensis|LGAT01000016|c1
Bacillus sp LK7|LDUN01000025|c1
Bacillus amyloliquefaciens|JZDI01000018|c1
Bacillus sp 916|AFSU01000053|c1
Bacillus velezensis|JXAM01000022|c1
Bacillus velezensis|LGAT01000001|c1
Bacillus amyloliquefaciens|LJDI01000008|c1
Bacillus amyloliquefaciens UASWS BA1|AWQY01000004|c4
Bacillus amyloliquefaciens|JQNZ01000018|c1
Bacillus amyloliquefaciens|JUDU01000026|c1
Bacillus amyloliquefaciens|JZDI01000005|c1
Bacillus velezensis|LGAU01000005|c1
Bacillus amyloliquefaciens|CYHL01000064|c1
Bacillus velezensis|JYGH01000020|c3
Streptococcus pneumoniae|CQZO01000005|c1
Bacillus amyloliquefaciens|CYHP01000085|c1
Bacillus sp LK7|LDUN01000002|c7
Bacillus amyloliquefaciens|LJAU01000005|c1
phormidolide
oocydin

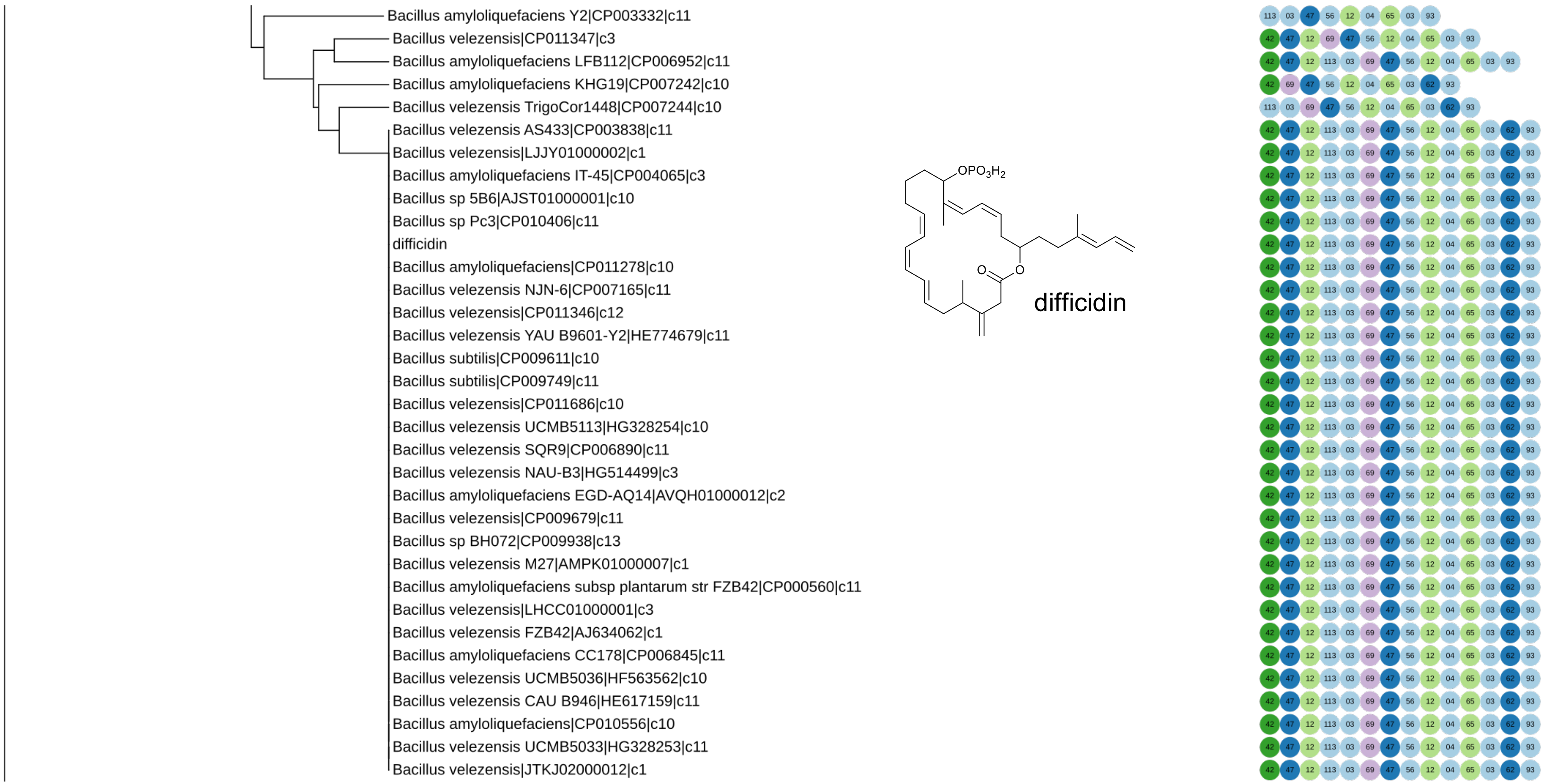
Bacillus amyloliquefaciens Y2|CP003332|c11
Bacillus velezensis|CP011347|c3
Bacillus amyloliquefaciens LFB112|CP006952|c11
Bacillus amyloliquefaciens KHG19|CP007242|c10
Bacillus velezensis TrigoCor1448|CP007244|c10
Bacillus velezensis AS433|CP003838|c11
Bacillus velezensis|LJJY01000002|c1
Bacillus amyloliquefaciens IT-45|CP004065|c3
Bacillus sp 5B6|AJST01000001|c10
Bacillus sp Pc3|CP010406|c11
difficidin
Bacillus amyloliquefaciens|CP011278|c10
Bacillus velezensis NJN-6|CP007165|c11
Bacillus velezensis|CP011346|c12
Bacillus velezensis YAU B9601-Y2|HE774679|c11
Bacillus subtilis|CP009611|c10
Bacillus subtilis|CP009749|c11
Bacillus velezensis|CP011686|c10
Bacillus velezensis UCMB5113|HG328254|c10
Bacillus velezensis SQR9|CP006890|c11
Bacillus velezensis NAU-B3|HG514499|c3
Bacillus amyloliquefaciens EGD-AQ14|AVQH01000012|c2
Bacillus velezensis|CP009679|c11
Bacillus sp BH072|CP009938|c13
Bacillus velezensis M27|AMPK01000007|c1
Bacillus amyloliquefaciens subsp plantarum str FZB42|CP000560|c11
Bacillus velezensis|LHCC01000001|c3
Bacillus velezensis FZB42|AJ634062|c1
Bacillus amyloliquefaciens CC178|CP006845|c11
Bacillus velezensis UCMB5036|HF563562|c10
Bacillus velezensis CAU B946|HE617159|c11
Bacillus amyloliquefaciens|CP010556|c10
Bacillus velezensis UCMB5033|HG328253|c11
Bacillus velezensis|JTKJ02000012|c1
OPO3H2
O
O
difficidin

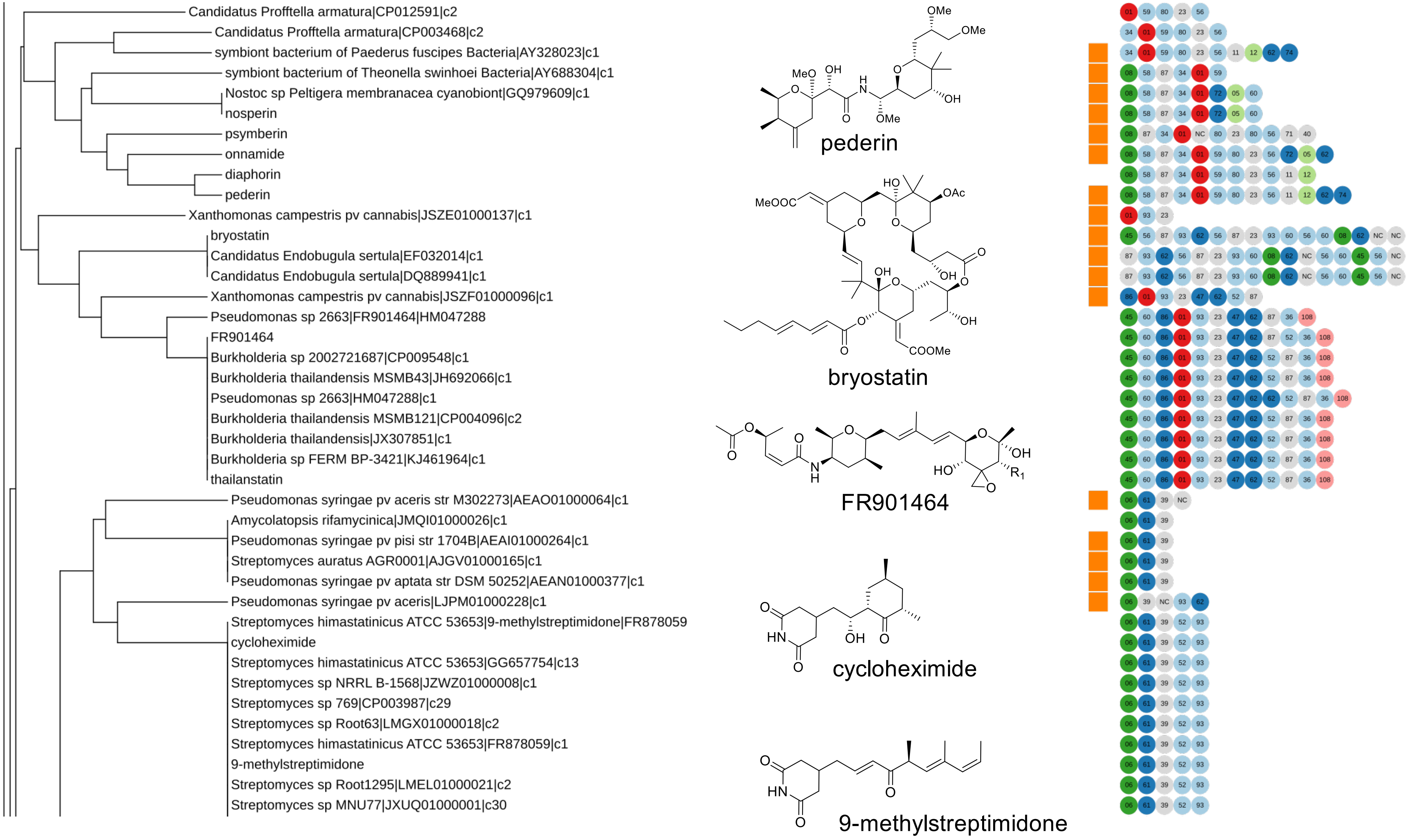
Candidatus Profftella armatura|CP012591|c2
Candidatus Profftella armatura|CP003468|c2
symbiont bacterium of Paederus fuscipes Bacteria|AY328023|c1
symbiont bacterium of Theonella swinhoei Bacteria|AY688304|c1
Nostoc sp Peltigera membranacea cyanobiont|GQ979609|c1
nosperin
psymberin
onnamide
diaphorin
pederin
Xanthomonas campestris pv cannabis|JSZE01000137|c1
bryostatin
Candidatus Endobugula sertula|EF032014|c1
Candidatus Endobugula sertula|DQ889941|c1
Xanthomonas campestris pv cannabis|JSZF01000096|c1
Pseudomonas sp 2663|FR901464|HM047288
FR901464
Burkholderia sp 2002721687|CP009548|c1
Burkholderia thailandensis MSMB43|JH692066|c1
Pseudomonas sp 2663|HM047288|c1
Burkholderia thailandensis MSMB121|CP004096|c2
Burkholderia thailandensis|JX307851|c1
Burkholderia sp FERM BP-3421|KJ461964|c1
thailanstatin
Pseudomonas syringae pv aceris str M302273|AEAO01000064|c1
Amycolatopsis rifamycinica|JMQI01000026|c1
Pseudomonas syringae pv pisi str 1704B|AEAI01000264|c1
Streptomyces auratus AGR0001|AJGV01000165|c1
Pseudomonas syringae pv aptata str DSM 50252|AEAN01000377|c1
Pseudomonas syringae pv aceris|LJPM01000228|c1
Streptomyces himastatinicus ATCC 53653|9-methylstreptimidone|FR878059
cycloheximide
Streptomyces himastatinicus ATCC 53653|GG657754|c13
Streptomyces sp NRRL B-1568|JZWZ01000008|c1
Streptomyces sp 769|CP003987|c29
Streptomyces sp Root63|LMGX01000018|c2
Streptomyces himastatinicus ATCC 53653|FR878059|c1
9-methylstreptimidone
Streptomyces sp Root1295|LMEL01000021|c2
Streptomyces sp MNU77|JXUQ01000001|c30
pederin
bryostatin
FR901464
cycloheximide
9-methylstreptimidone

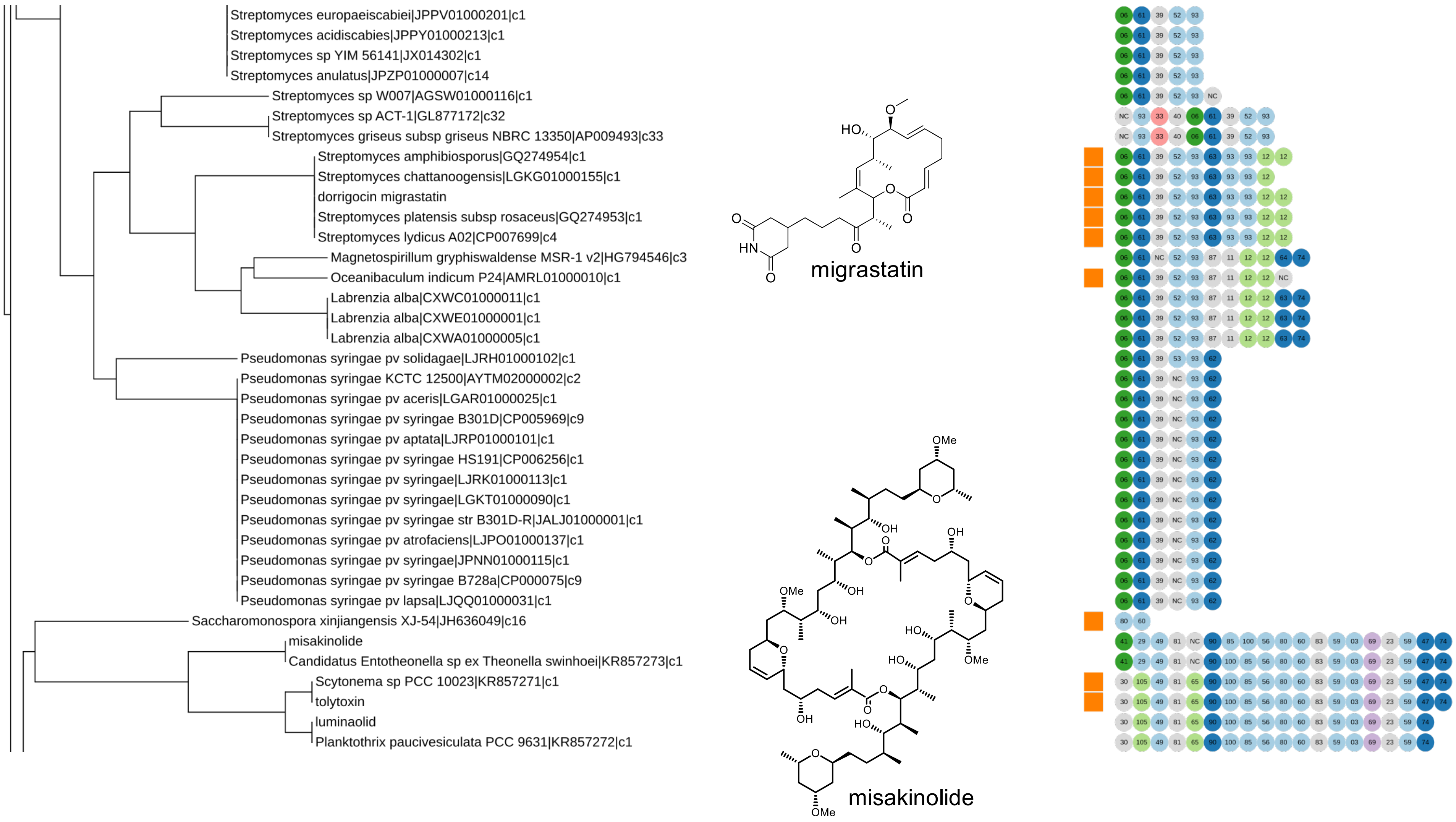
Streptomyces europaeiscabiei|JPPV01000201|c1
Streptomyces acidiscabies|JPPY01000213|c1
Streptomyces sp YIM 56141|JX014302|c1
Streptomyces anulatus|JPZP01000007|c14
Streptomyces sp W007|AGSW01000116|c1
Streptomyces sp ACT-1|GL877172|c32
Streptomyces griseus subsp griseus NBRC 13350|AP009493|c33
Streptomyces amphibiosporus|GQ274954|c1
Streptomyces chattanoogensis|LGKG01000155|c1
dorrigocin migrastatin
Streptomyces platensis subsp rosaceus|GQ274953|c1
Streptomyces lydicus A02|CP007699|c4
Magnetospirillum gryphiswaldense MSR-1 v2|HG794546|c3
Oceanibaculum indicum P24|AMRL01000010|c1
Labrenzia alba|CXWC01000011|c1
Labrenzia alba|CXWE01000001|c1
Labrenzia alba|CXWA01000005|c1
Pseudomonas syringae pv solidagae|LJRH01000102|c1
Pseudomonas syringae KCTC 12500|AYTM02000002|c2
Pseudomonas syringae pv aceris|LGAR01000025|c1
Pseudomonas syringae pv syringae B301D|CP005969|c9
Pseudomonas syringae pv aptata|LJRP01000101|c1
Pseudomonas syringae pv syringae HS191|CP006256|c1
Pseudomonas syringae pv syringae|LJRK01000113|c1
Pseudomonas syringae pv syringae|LGKT01000090|c1
Pseudomonas syringae pv syringae str B301D-R|JALJ01000001|c1
Pseudomonas syringae pv atrofaciens|LJPO01000137|c1
Pseudomonas syringae pv syringae|JPNN01000115|c1
Pseudomonas syringae pv syringae B728a|CP000075|c9
Pseudomonas syringae pv lapsa|LJQQ01000031|c1
Saccharomonospora xinjiangensis XJ-54|JH636049|c16
misakinolide
Candidatus Entotheonella sp ex Theonella swinhoei|KR857273|c1
Scytonema sp PCC 10023|KR857271|c1
tolytoxin
luminaolid
Planktothrix paucivesiculata PCC 9631|KR857272|c1
migrastatin
misakinolide

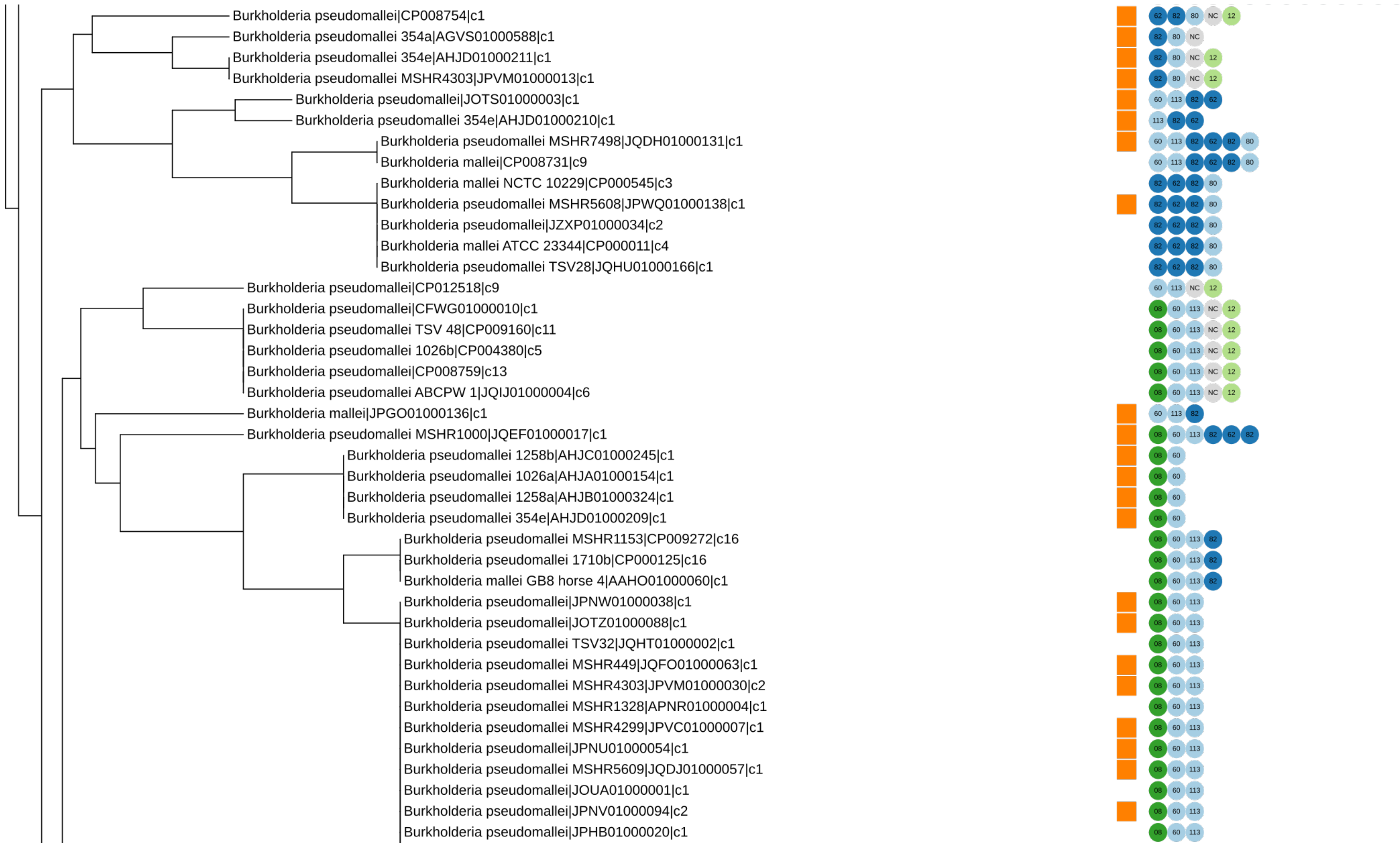
Burkholderia pseudomallei|CP008754|c1
Burkholderia pseudomallei 354a|AGVS01000588|c1
Burkholderia pseudomallei 354e|AHJD01000211|c1
Burkholderia pseudomallei MSHR4303|JPVM01000013|c1
Burkholderia pseudomallei|JOTS01000003|c1
Burkholderia pseudomallei 354e|AHJD01000210|c1
Burkholderia pseudomallei MSHR7498|JQDH01000131|c1
Burkholderia mallei|CP008731|c9
Burkholderia mallei NCTC 10229|CP000545|c3
Burkholderia pseudomallei MSHR5608|JPWQ01000138|c1
Burkholderia pseudomallei|JZXP01000034|c2
Burkholderia mallei ATCC 23344|CP000011|c4
Burkholderia pseudomallei TSV28|JQHU01000166|c1
Burkholderia pseudomallei|CP012518|c9
Burkholderia pseudomallei|CFWG01000010|c1
Burkholderia pseudomallei TSV 48|CP009160|c11
Burkholderia pseudomallei 1026b|CP004380|c5
Burkholderia pseudomallei|CP008759|c13
Burkholderia pseudomallei ABCPW 1|JQIJ01000004|c6
Burkholderia mallei|JPGO01000136|c1
Burkholderia pseudomallei MSHR1000|JQEF01000017|c1
Burkholderia pseudomallei 1258b|AHJC01000245|c1
Burkholderia pseudomallei 1026a|AHJA01000154|c1
Burkholderia pseudomallei 1258a|AHJB01000324|c1
Burkholderia pseudomallei 354e|AHJD01000209|c1
Burkholderia pseudomallei MSHR1153|CP009272|c16
Burkholderia pseudomallei 1710b|CP000125|c16
Burkholderia mallei GB8 horse 4|AAHO01000060|c1
Burkholderia pseudomallei|JPNW01000038|c1
Burkholderia pseudomallei|JOTZ01000088|c1
Burkholderia pseudomallei TSV32|JQHT01000002|c1
Burkholderia pseudomallei MSHR449|JQFO01000063|c1
Burkholderia pseudomallei MSHR4303|JPVM01000030|c2
Burkholderia pseudomallei MSHR1328|APNR01000004|c1
Burkholderia pseudomallei MSHR4299|JPVC01000007|c1
Burkholderia pseudomallei|JPNU01000054|c1
Burkholderia pseudomallei MSHR5609|JQDJ01000057|c1
Burkholderia pseudomallei|JOUA01000001|c1
Burkholderia pseudomallei|JPNV01000094|c2
Burkholderia pseudomallei|JPHB01000020|c1

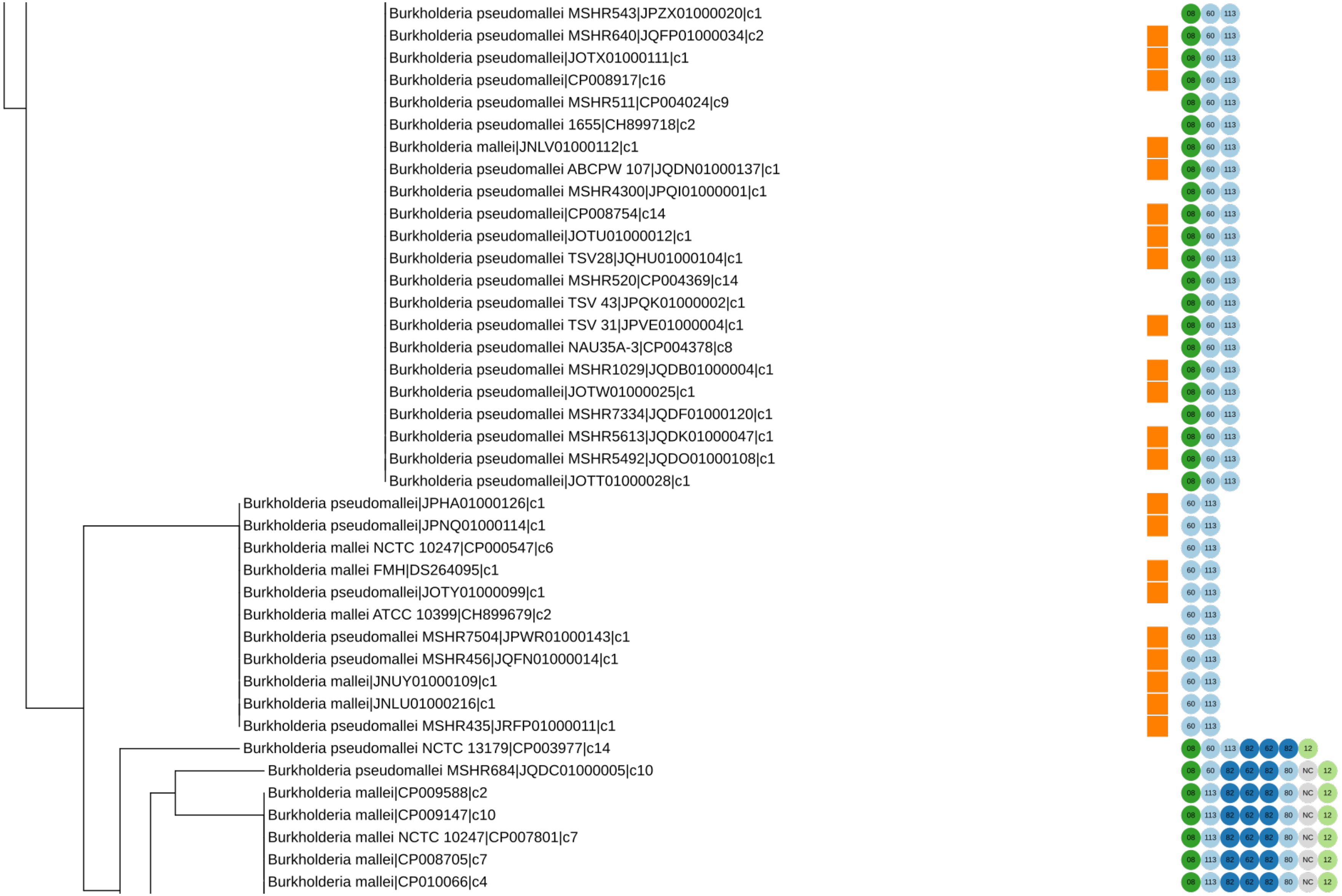
Burkholderia pseudomallei MSHR543|JPZX01000020|c1
Burkholderia pseudomallei MSHR640|JQFP01000034|c2
Burkholderia pseudomallei|JOTX01000111|c1
Burkholderia pseudomallei|CP008917|c16
Burkholderia pseudomallei MSHR511|CP004024|c9
Burkholderia pseudomallei 1655|CH899718|c2
Burkholderia mallei|JNLV01000112|c1
Burkholderia pseudomallei ABCPW 107|JQDN01000137|c1
Burkholderia pseudomallei MSHR4300|JPQI01000001|c1
Burkholderia pseudomallei|CP008754|c14
Burkholderia pseudomallei|JOTU01000012|c1
Burkholderia pseudomallei TSV28|JQHU01000104|c1
Burkholderia pseudomallei MSHR520|CP004369|c14
Burkholderia pseudomallei TSV 43|JPQK01000002|c1
Burkholderia pseudomallei TSV 31|JPVE01000004|c1
Burkholderia pseudomallei NAU35A-3|CP004378|c8
Burkholderia pseudomallei MSHR1029|JQDB01000004|c1
Burkholderia pseudomallei|JOTW01000025|c1
Burkholderia pseudomallei MSHR7334|JQDF01000120|c1
Burkholderia pseudomallei MSHR5613|JQDK01000047|c1
Burkholderia pseudomallei MSHR5492|JQDO01000108|c1
Burkholderia pseudomallei|JOTT01000028|c1
Burkholderia pseudomallei|JPHA01000126|c1
Burkholderia pseudomallei|JPNQ01000114|c1
Burkholderia mallei NCTC 10247|CP000547|c6
Burkholderia mallei FMH|DS264095|c1
Burkholderia pseudomallei|JOTY01000099|c1
Burkholderia mallei ATCC 10399|CH899679|c2
Burkholderia pseudomallei MSHR7504|JPWR01000143|c1
Burkholderia pseudomallei MSHR456|JQFN01000014|c1
Burkholderia mallei|JNUY01000109|c1
Burkholderia mallei|JNLU01000216|c1
Burkholderia pseudomallei MSHR435|JRFP01000011|c1
Burkholderia pseudomallei NCTC 13179|CP003977|c14
Burkholderia pseudomallei MSHR684|JQDC01000005|c10
Burkholderia mallei|CP009588|c2
Burkholderia mallei|CP009147|c10
Burkholderia mallei NCTC 10247|CP007801|c7
Burkholderia mallei|CP008705|c7
Burkholderia mallei|CP010066|c4

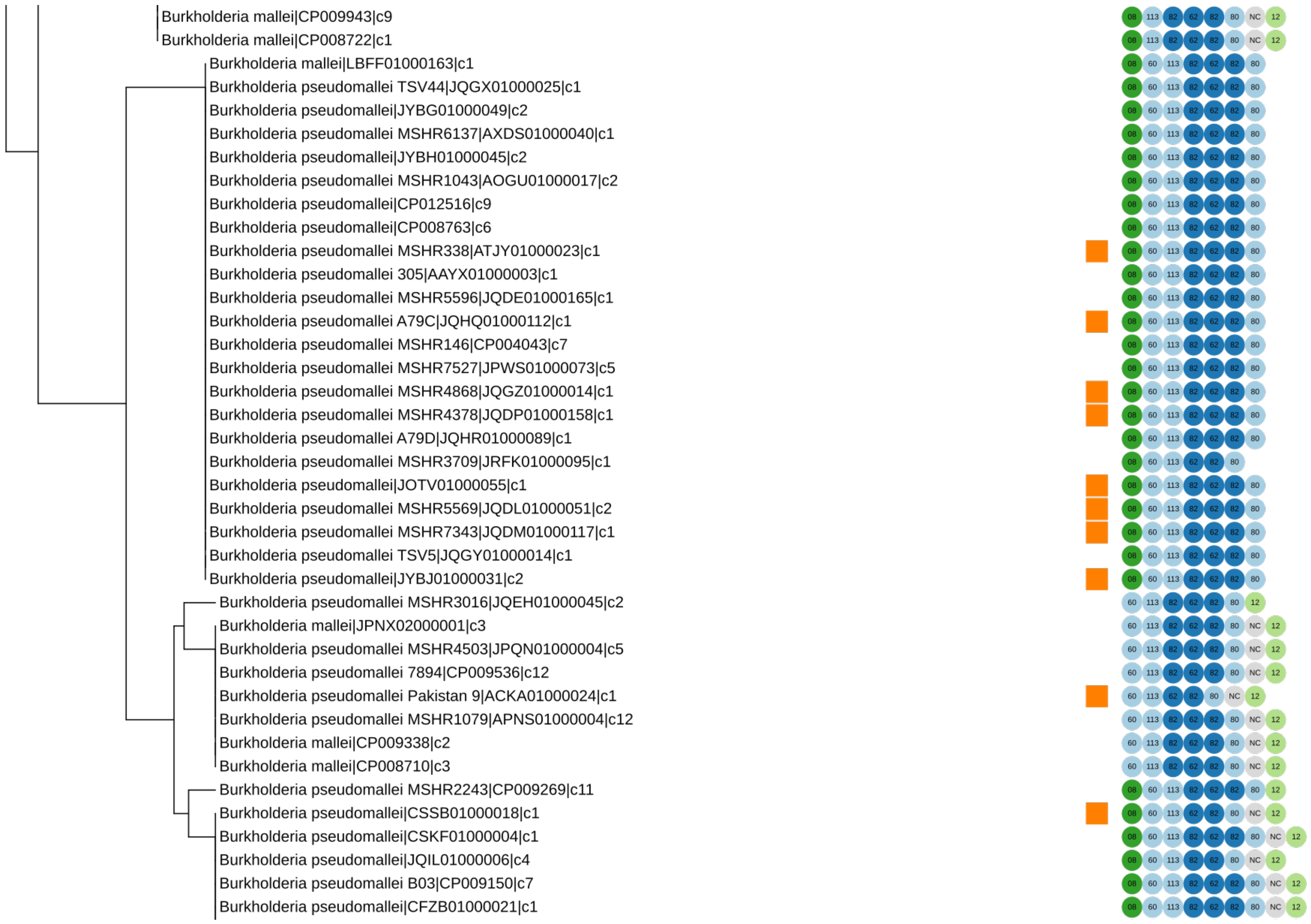
Burkholderia mallei|CP009943|c9
Burkholderia mallei|CP008722|c1
Burkholderia mallei|LBFF01000163|c1
Burkholderia pseudomallei TSV44|JQGX01000025|c1
Burkholderia pseudomallei|JYBG01000049|c2
Burkholderia pseudomallei MSHR6137|AXDS01000040|c1
Burkholderia pseudomallei|JYBH01000045|c2
Burkholderia pseudomallei MSHR1043|AOGU01000017|c2
Burkholderia pseudomallei|CP012516|c9
Burkholderia pseudomallei|CP008763|c6
Burkholderia pseudomallei MSHR338|ATJY01000023|c1
Burkholderia pseudomallei 305|AAYX01000003|c1
Burkholderia pseudomallei MSHR5596|JQDE01000165|c1
Burkholderia pseudomallei A79C|JQHQ01000112|c1
Burkholderia pseudomallei MSHR146|CP004043|c7
Burkholderia pseudomallei MSHR7527|JPWS01000073|c5
Burkholderia pseudomallei MSHR4868|JQGZ01000014|c1
Burkholderia pseudomallei MSHR4378|JQDP01000158|c1
Burkholderia pseudomallei A79D|JQHR01000089|c1
Burkholderia pseudomallei MSHR3709|JRFK01000095|c1
Burkholderia pseudomallei|JOTV01000055|c1
Burkholderia pseudomallei MSHR5569|JQDL01000051|c2
Burkholderia pseudomallei MSHR7343|JQDM01000117|c1
Burkholderia pseudomallei TSV5|JQGY01000014|c1
Burkholderia pseudomallei|JYBJ01000031|c2
Burkholderia pseudomallei MSHR3016|JQEH01000045|c2
Burkholderia mallei|JPNX02000001|c3
Burkholderia pseudomallei MSHR4503|JPQN01000004|c5
Burkholderia pseudomallei 7894|CP009536|c12
Burkholderia pseudomallei Pakistan 9|ACKA01000024|c1
Burkholderia pseudomallei MSHR1079|APNS01000004|c12
Burkholderia mallei|CP009338|c2
Burkholderia mallei|CP008710|c3
Burkholderia pseudomallei MSHR2243|CP009269|c11
Burkholderia pseudomallei|CSSB01000018|c1
Burkholderia pseudomallei|CSKF01000004|c1
Burkholderia pseudomallei|JQIL01000006|c4
Burkholderia pseudomallei B03|CP009150|c7
Burkholderia pseudomallei|CFZB01000021|c1

Burkholderia pseudomallei 576|ACCE01000001|c3	08	60	113	82	62	82	80	NC	12
Burkholderia pseudomallei|CSNB01000018|c1	08	60	113	82	62	82	80	NC	12
Burkholderia pseudomallei MSHR465J|JPZW01000007|c4	08	60	113	82	62	82	80	NC	12
Burkholderia pseudomallei|CSMS01000020|c1	08	60	113	82	62	82	80	NC	12
Burkholderia pseudomallei|CFXU01000017|c1	08	60	113	82	62	82	80	NC	12
Burkholderia pseudomallei|CGHT01000019|c1	08	60	113	82	62	82	80	NC	12
Burkholderia pseudomallei ABCPW 91|JPUY01000005|c4	08	60	113	82	62	82	80	NC	12
Burkholderia pseudomallei|CHFE01000004|c2	08	60	113	82	62	82	80	NC	12
Burkholderia pseudomallei|CSNM01000020|c1	08	60	113	82	62	82	80	NC	12
Burkholderia pseudomallei|CFXB01000018|c1	08	60	113	82	62	82	80	NC	12
Burkholderia pseudomallei|CSLL01000022|c1	08	60	113	82	62	82	80	NC	12
Burkholderia pseudomallei MSHR4462|JPQM01000003|c7	08	60	113	82	62	82	80	NC	12
Burkholderia pseudomallei 1106a|CP000573|c9	08	60	113	82	62	82	80	NC	12
Burkholderia pseudomallei|JYBK01000026|c1	08	60	113	82	62	82	80	NC	12
Burkholderia pseudomallei|CSLA01000023|c1	08	60	113	82	62	82	80	NC	12
Burkholderia pseudomallei|CSKJ01000001|c4	08	60	113	82	62	82	80	NC	12
Burkholderia pseudomallei|CSKV01000024|c1	08	60	113	82	62	82	80	NC	12
Burkholderia pseudomallei|CFVM01000011|c1	08	60	113	82	62	82	80	NC	12
Burkholderia pseudomallei 576|CP008778|c11	08	60	113	82	62	82	80	NC	12
Burkholderia pseudomallei|CSKG01000003|c2	08	60	113	82	62	82	80	NC	12
Burkholderia pseudomallei|CSLK01000020|c1	08	60	113	82	62	82	80	NC	12
Burkholderia pseudomallei|CFXG01000017|c1	08	60	113	82	62	82	80	NC	12
Burkholderia pseudomallei|CSLC01000019|c1	08	60	113	82	62	82	80	NC	12
Burkholderia pseudomallei MSHR4375|JPVI01000007|c4	08	60	113	82	62	82	80	NC	12
Burkholderia pseudomallei MSHR3965|CP009152|c9	08	60	113	82	62	82	80	NC	12
Burkholderia pseudomallei|CFVI01000002|c4	08	60	113	82	62	82	80	NC	12
Burkholderia pseudomallei BDU 2|JPVG01000009|c5	08	60	113	82	62	82	80	NC	12
Burkholderia pseudomallei|CFWJ01000023|c1	08	60	113	82	62	82	80	NC	12
Burkholderia pseudomallei|CIAW01000001|c4	08	60	113	82	62	82	80	NC	12
Burkholderia pseudomallei|CIKM01000012|c2	08	60	113	82	62	82	80	NC	12
Burkholderia pseudomallei|CP008782|c5	08	60	113	82	62	82	80	NC	12
Burkholderia pseudomallei|CFXD01000019|c1	08	60	113	82	62	82	80	NC	12
Burkholderia pseudomallei|CSQC01000019|c1	08	60	113	82	62	82	80	NC	12
Burkholderia pseudomallei|CIGK01000007|c1	08	60	113	82	62	82	80	NC	12
Burkholderia pseudomallei BPC006|CP003782|c9	08	60	113	82	62	82	80	NC	12
Burkholderia pseudomallei|CSLD01000008|c2	08	60	113	82	62	82	80	NC	12
Burkholderia pseudomallei|CIBE01000022|c1	08	60	113	82	62	82	80	NC	12
Burkholderia pseudomallei|CIGV01000018|c1	08	60	113	82	62	82	80	NC	12
Burkholderia pseudomallei|CGFJ01000020|c1	08	60	113	82	62	82	80	NC	12

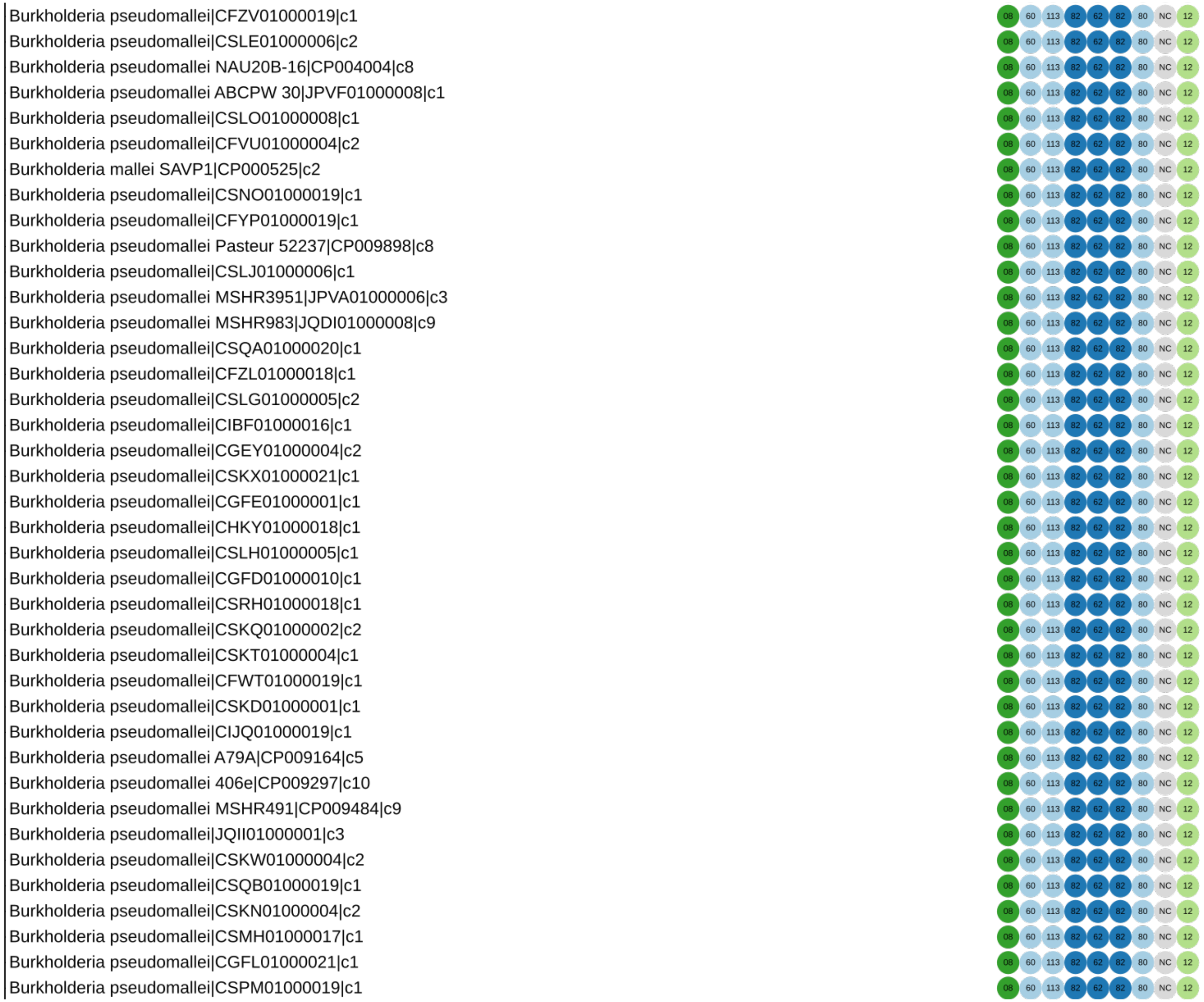
Burkholderia pseudomallei|CFZV01000019|c1
Burkholderia pseudomallei|CSLE01000006|c2
Burkholderia pseudomallei NAU20B-16|CP004004|c8
Burkholderia pseudomallei ABCPW 30|JPVF01000008|c1
Burkholderia pseudomallei|CSLO01000008|c1
Burkholderia pseudomallei|CFVU01000004|c2
Burkholderia mallei SAVP1|CP000525|c2
Burkholderia pseudomallei|CSNO01000019|c1
Burkholderia pseudomallei|CFYP01000019|c1
Burkholderia pseudomallei Pasteur 52237|CP009898|c8
Burkholderia pseudomallei|CSLJ01000006|c1
Burkholderia pseudomallei MSHR3951|JPVA01000006|c3
Burkholderia pseudomallei MSHR983|JQDI01000008|c9
Burkholderia pseudomallei|CSQA01000020|c1
Burkholderia pseudomallei|CFZL01000018|c1
Burkholderia pseudomallei|CSLG01000005|c2
Burkholderia pseudomallei|CIBF01000016|c1
Burkholderia pseudomallei|CGEY01000004|c2
Burkholderia pseudomallei|CSKX01000021|c1
Burkholderia pseudomallei|CGFE01000001|c1
Burkholderia pseudomallei|CHKY01000018|c1
Burkholderia pseudomallei|CSLH01000005|c1
Burkholderia pseudomallei|CGFD01000010|c1
Burkholderia pseudomallei|CSRH01000018|c1
Burkholderia pseudomallei|CSKQ01000002|c2
Burkholderia pseudomallei|CSKT01000004|c1
Burkholderia pseudomallei|CFWT01000019|c1
Burkholderia pseudomallei|CSKD01000001|c1
Burkholderia pseudomallei|CIJQ01000019|c1
Burkholderia pseudomallei A79A|CP009164|c5
Burkholderia pseudomallei 406e|CP009297|c10
Burkholderia pseudomallei MSHR491|CP009484|c9
Burkholderia pseudomallei|JQII01000001|c3
Burkholderia pseudomallei|CSKW01000004|c2
Burkholderia pseudomallei|CSQB01000019|c1
Burkholderia pseudomallei|CSKN01000004|c2
Burkholderia pseudomallei|CSMH01000017|c1
Burkholderia pseudomallei|CGFL01000021|c1
Burkholderia pseudomallei|CSPM01000019|c1

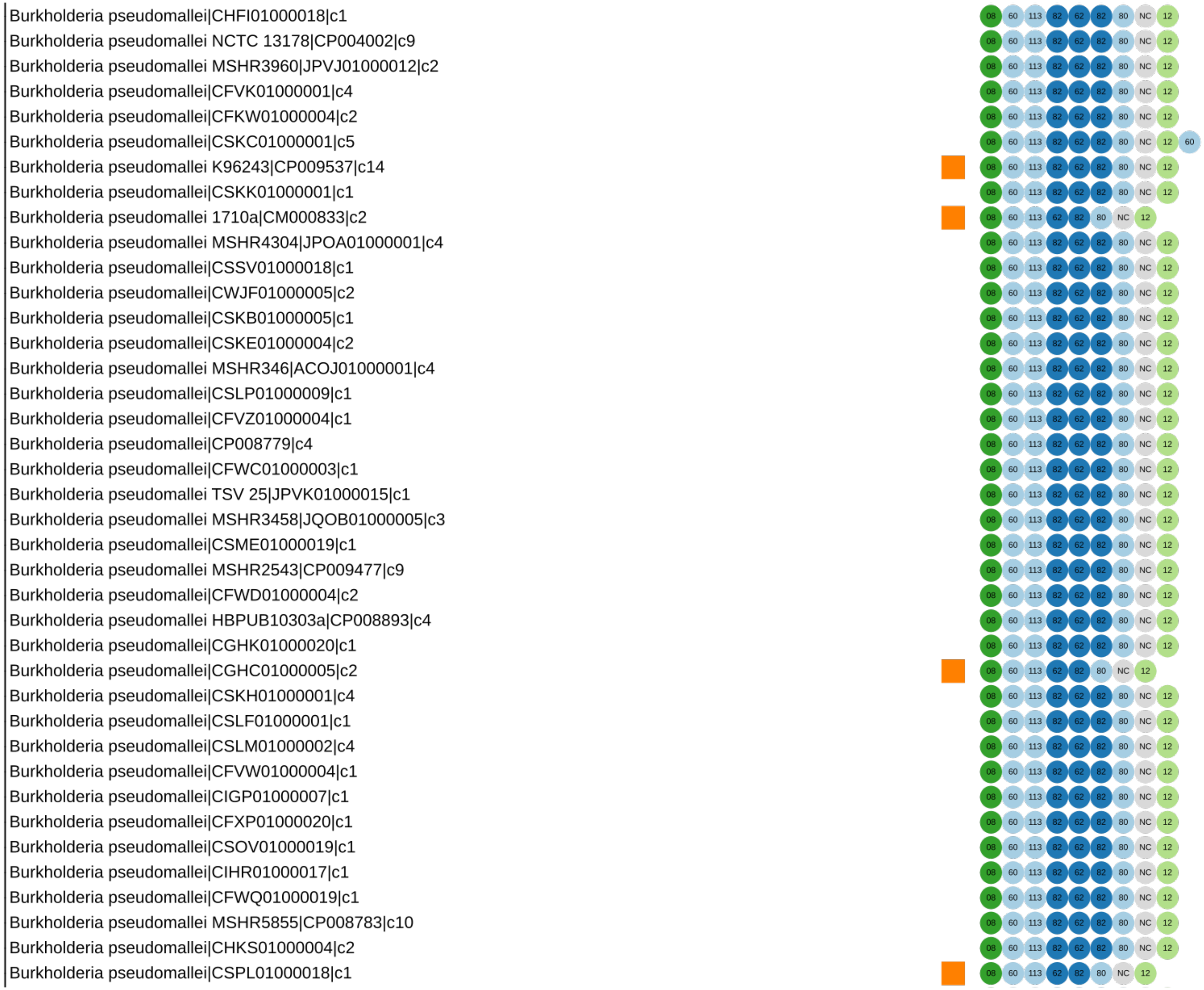
Burkholderia pseudomallei|CHFI01000018|c1
Burkholderia pseudomallei NCTC 13178|CP004002|c9
Burkholderia pseudomallei MSHR3960|JPVJ01000012|c2
Burkholderia pseudomallei|CFVK01000001|c4
Burkholderia pseudomallei|CFKW01000004|c2
Burkholderia pseudomallei|CSKC01000001|c5
Burkholderia pseudomallei K96243|CP009537|c14
Burkholderia pseudomallei|CSKK01000001|c1
Burkholderia pseudomallei 1710a|CM000833|c2
Burkholderia pseudomallei MSHR4304|JPOA01000001|c4
Burkholderia pseudomallei|CSSV01000018|c1
Burkholderia pseudomallei|CWJF01000005|c2
Burkholderia pseudomallei|CSKB01000005|c1
Burkholderia pseudomallei|CSKE01000004|c2
Burkholderia pseudomallei MSHR346|ACOJ01000001|c4
Burkholderia pseudomallei|CSLP01000009|c1
Burkholderia pseudomallei|CFVZ01000004|c1
Burkholderia pseudomallei|CP008779|c4
Burkholderia pseudomallei|CFWC01000003|c1
Burkholderia pseudomallei TSV 25|JPVK01000015|c1
Burkholderia pseudomallei MSHR3458|JQOB01000005|c3
Burkholderia pseudomallei|CSME01000019|c1
Burkholderia pseudomallei MSHR2543|CP009477|c9
Burkholderia pseudomallei|CFWD01000004|c2
Burkholderia pseudomallei HBPUB10303a|CP008893|c4
Burkholderia pseudomallei|CGHK01000020|c1
Burkholderia pseudomallei|CGHC01000005|c2
Burkholderia pseudomallei|CSKH01000001|c4
Burkholderia pseudomallei|CSLF01000001|c1
Burkholderia pseudomallei|CSLM01000002|c4
Burkholderia pseudomallei|CFVW01000004|c1
Burkholderia pseudomallei|CIGP01000007|c1
Burkholderia pseudomallei|CFXP01000020|c1
Burkholderia pseudomallei|CSOV01000019|c1
Burkholderia pseudomallei|CIHR01000017|c1
Burkholderia pseudomallei|CFWQ01000019|c1
Burkholderia pseudomallei MSHR5855|CP008783|c10
Burkholderia pseudomallei|CHKS01000004|c2
Burkholderia pseudomallei|CSPL01000018|c1

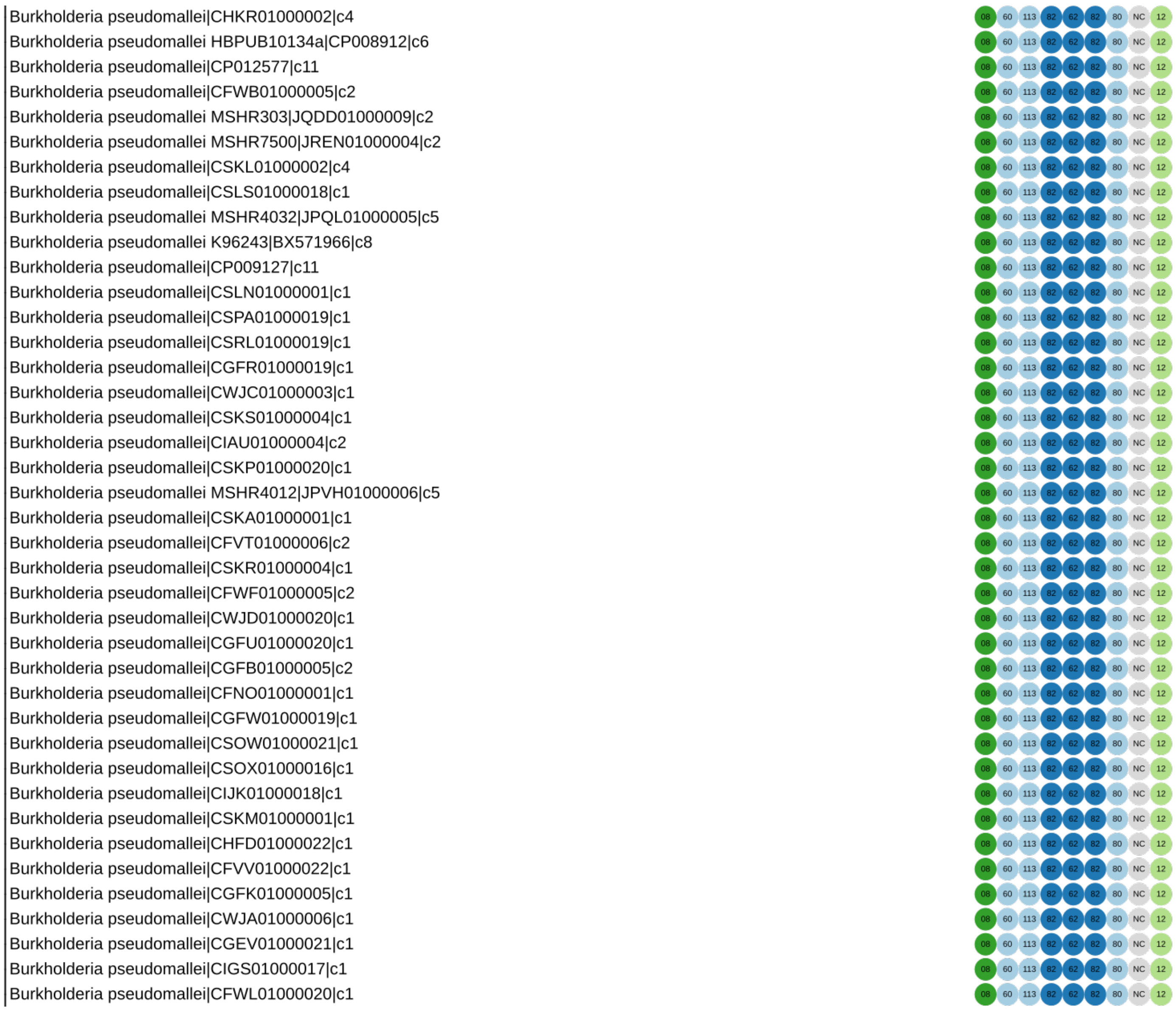
Burkholderia pseudomallei|CHKR01000002|c4 08 60 113 82 62 82 80 NC 12
Burkholderia pseudomallei HBPUB10134a|CP008912|c6 08 60 113 82 62 82 80 NC 12
Burkholderia pseudomallei|CP012577|c11 08 60 113 82 62 82 80 NC 12
Burkholderia pseudomallei|CFWB01000005|c2 08 60 113 82 62 82 80 NC 12
Burkholderia pseudomallei MSHR303|JQDD01000009|c2 08 60 113 82 62 82 80 NC 12
Burkholderia pseudomallei MSHR7500|JREN01000004|c2 08 60 113 82 62 82 80 NC 12
Burkholderia pseudomallei|CSKL01000002|c4 08 60 113 82 62 82 80 NC 12
Burkholderia pseudomallei|CSLS01000018|c1 08 60 113 82 62 82 80 NC 12
Burkholderia pseudomallei MSHR4032|JPQL01000005|c5 08 60 113 82 62 82 80 NC 12
Burkholderia pseudomallei K96243|BX571966|c8 08 60 113 82 62 82 80 NC 12
Burkholderia pseudomallei|CP009127|c11 08 60 113 82 62 82 80 NC 12
Burkholderia pseudomallei|CSLN01000001|c1 08 60 113 82 62 82 80 NC 12
Burkholderia pseudomallei|CSPA01000019|c1 08 60 113 82 62 82 80 NC 12
Burkholderia pseudomallei|CSRL01000019|c1 08 60 113 82 62 82 80 NC 12
Burkholderia pseudomallei|CGFR01000019|c1 08 60 113 82 62 82 80 NC 12
Burkholderia pseudomallei|CWJC01000003|c1 08 60 113 82 62 82 80 NC 12
Burkholderia pseudomallei|CSKS01000004|c1 08 60 113 82 62 82 80 NC 12
Burkholderia pseudomallei|CIAU01000004|c2 08 60 113 82 62 82 80 NC 12
Burkholderia pseudomallei|CSKP01000020|c1 08 60 113 82 62 82 80 NC 12
Burkholderia pseudomallei MSHR4012|JPVH01000006|c5 08 60 113 82 62 82 80 NC 12
Burkholderia pseudomallei|CSKA01000001|c1 08 60 113 82 62 82 80 NC 12
Burkholderia pseudomallei|CFVT01000006|c2 08 60 113 82 62 82 80 NC 12
Burkholderia pseudomallei|CSKR01000004|c1 08 60 113 82 62 82 80 NC 12
Burkholderia pseudomallei|CFWF01000005|c2 08 60 113 82 62 82 80 NC 12
Burkholderia pseudomallei|CWJD01000020|c1 08 60 113 82 62 82 80 NC 12
Burkholderia pseudomallei|CGFU01000020|c1 08 60 113 82 62 82 80 NC 12
Burkholderia pseudomallei|CGFB01000005|c2 08 60 113 82 62 82 80 NC 12
Burkholderia pseudomallei|CFNO01000001|c1 08 60 113 82 62 82 80 NC 12
Burkholderia pseudomallei|CGFW01000019|c1 08 60 113 82 62 82 80 NC 12
Burkholderia pseudomallei|CSOW01000021|c1 08 60 113 82 62 82 80 NC 12
Burkholderia pseudomallei|CSOX01000016|c1 08 60 113 82 62 82 80 NC 12
Burkholderia pseudomallei|CIJK01000018|c1 08 60 113 82 62 82 80 NC 12
Burkholderia pseudomallei|CSKM01000001|c1 08 60 113 82 62 82 80 NC 12
Burkholderia pseudomallei|CHFD01000022|c1 08 60 113 82 62 82 80 NC 12
Burkholderia pseudomallei|CFVV01000022|c1 08 60 113 82 62 82 80 NC 12
Burkholderia pseudomallei|CGFK01000005|c1 08 60 113 82 62 82 80 NC 12
Burkholderia pseudomallei|CWJA01000006|c1 08 60 113 82 62 82 80 NC 12
Burkholderia pseudomallei|CGEV01000021|c1 08 60 113 82 62 82 80 NC 12
Burkholderia pseudomallei|CIGS01000017|c1 08 60 113 82 62 82 80 NC 12
Burkholderia pseudomallei|CFWL01000020|c1 08 60 113 82 62 82 80 NC 12

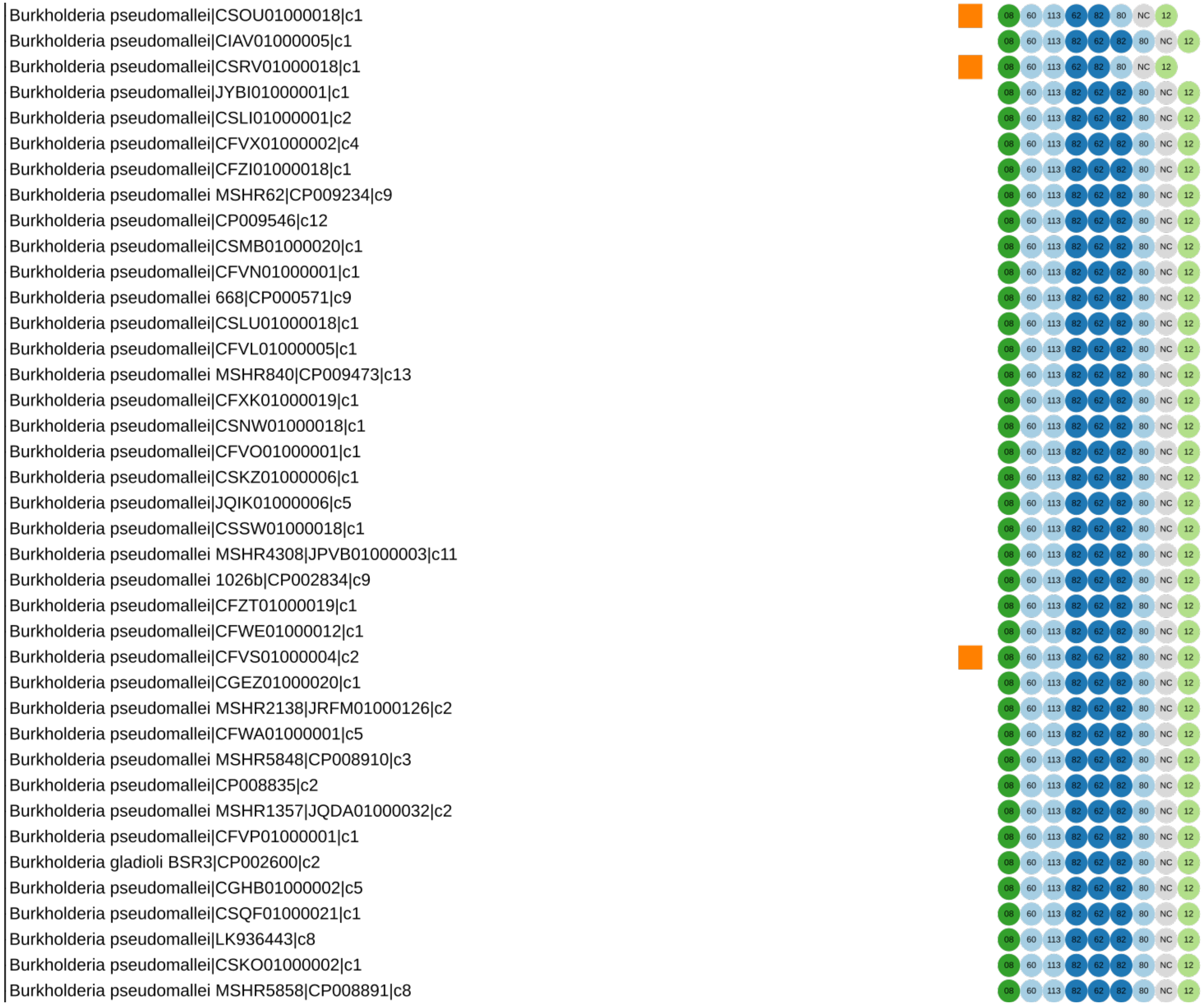
Burkholderia pseudomallei|CSOU01000018|c1
Burkholderia pseudomallei|CIAV01000005|c1
Burkholderia pseudomallei|CSRV01000018|c1
Burkholderia pseudomallei|JYBI01000001|c1
Burkholderia pseudomallei|CSLI01000001|c2
Burkholderia pseudomallei|CFVX01000002|c4
Burkholderia pseudomallei|CFZI01000018|c1
Burkholderia pseudomallei MSHR62|CP009234|c9
Burkholderia pseudomallei|CP009546|c12
Burkholderia pseudomallei|CSMB01000020|c1
Burkholderia pseudomallei|CFVN01000001|c1
Burkholderia pseudomallei 668|CP000571|c9
Burkholderia pseudomallei|CSLU01000018|c1
Burkholderia pseudomallei|CFVL01000005|c1
Burkholderia pseudomallei MSHR840|CP009473|c13
Burkholderia pseudomallei|CFXK01000019|c1
Burkholderia pseudomallei|CSNW01000018|c1
Burkholderia pseudomallei|CFVO01000001|c1
Burkholderia pseudomallei|CSKZ01000006|c1
Burkholderia pseudomallei|JQIK01000006|c5
Burkholderia pseudomallei|CSSW01000018|c1
Burkholderia pseudomallei MSHR4308|JPVB01000003|c11
Burkholderia pseudomallei 1026b|CP002834|c9
Burkholderia pseudomallei|CFZT01000019|c1
Burkholderia pseudomallei|CFWE01000012|c1
Burkholderia pseudomallei|CFVS01000004|c2
Burkholderia pseudomallei|CGEZ01000020|c1
Burkholderia pseudomallei MSHR2138|JRFM01000126|c2
Burkholderia pseudomallei|CFWA01000001|c5
Burkholderia pseudomallei MSHR5848|CP008910|c3
Burkholderia pseudomallei|CP008835|c2
Burkholderia pseudomallei MSHR1357|JQDA01000032|c2
Burkholderia pseudomallei|CFVP01000001|c1
Burkholderia gladioli BSR3|CP002600|c2
Burkholderia pseudomallei|CGHB01000002|c5
Burkholderia pseudomallei|CSQF01000021|c1
Burkholderia pseudomallei|LK936443|c8
Burkholderia pseudomallei|CSKO01000002|c1
Burkholderia pseudomallei MSHR5858|CP008891|c8

Burkholderia pseudomallei|CSKU01000004|c1	08	60	113	82	62	82	80	NC	12
Burkholderia pseudomallei|CIGX01000017|c1	08	60	113	82	62	82	80	NC	12
Burkholderia pseudomallei|CP009586|c4	08	60	113	82	62	82	80	NC	12
Burkholderia pseudomallei MSHR4003|JPUZ01000004|c4	08	60	113	82	62	82	80	NC	12
Burkholderia pseudomallei|CP009210|c10	08	60	113	82	62	82	80	NC	12
Burkholderia pseudomallei|CFNN01000002|c1	08	60	113	82	62	82	80	NC	12
Burkholderia pseudomallei|CFVQ01000005|c1	08	60	113	82	62	82	80	NC	12
Burkholderia pseudomallei MSHR4372|JPQJ01000004|c3	08	60	113	82	62	82	80	NC	12
Burkholderia pseudomallei MSHR3335|JRFL01000009|c4	08	60	113	82	62	82	80	NC	12
Burkholderia pseudomallei|CP010974|c8	08	60	113	82	62	82	80	NC	12
Burkholderia pseudomallei MSHR2990|JQHV01000041|c2	08	60	113	82	62	82	80	NC	12
Burkholderia pseudomallei|CSRJ01000019|c1	08	60	113	82	62	82	80	NC	12
Burkholderia pseudomallei|LGKL01000032|c4	08	60	113	82	62	82	80	NC	12
Burkholderia pseudomallei|CSSE01000019|c1	08	60	113	82	62	82	80	NC	12
Burkholderia pseudomallei MSHR4377|JPQH01000001|c3	08	60	113	82	62	82	80	NC	12
Burkholderia pseudomallei|CSKI01000005|c1	08	60	113	82	62	82	80	NC	12
Burkholderia pseudomallei PB08298010|CP009550|c9	08	60	113	82	62	82	80	NC	12
Burkholderia pseudomallei|CGHG01000001|c1	08	60	113	82	62	82	80	NC	12
Burkholderia pseudomallei|CIBL01000018|c1	08	60	113	82	62	82	80	NC	12
Burkholderia pseudomallei|CFVR01000005|c2	08	60	113	82	62	82	80	NC	12
Burkholderia pseudomallei MSHR3964|JPVD01000007|c11	08	60	113	82	62	82	80	NC	12
Burkholderia pseudomallei|CGEX01000006|c1	08	60	113	82	62	82	80	NC	12
Burkholderia pseudomallei|CHFB01000005|c1	08	60	113	82	62	82	80	NC	12
Burkholderia pseudomallei|CGFC01000003|c3	08	60	113	82	62	82	80	NC	12
Burkholderia pseudomallei|CHFC01000005|c2	08	60	113	82	62	82	80	NC	12
Burkholderia pseudomallei|CIJT01000019|c1	08	60	113	82	62	82	80	NC	12
Burkholderia pseudomallei K42|CP009163|c1	08	60	113	82	62	82	80	NC	12
Burkholderia pseudomallei MSHR4000|JPVL01000019|c3	08	60	113	82	62	82	80	NC	12
Burkholderia pseudomallei|CSQP01000020|c1	08	60	113	82	62	82	80	NC	12
Burkholderia pseudomallei 1106b|CM000775|c6	08	60	113	82	62	82	80	NC	12
Burkholderia pseudomallei MSHR2451|JQEG01000036|c1	08	60	113	82	62	82	80	NC	12
Burkholderia pseudomallei|JQIM01000009|c3	08	60	113	82	62	82	80	NC	12
Burkholderia pseudomallei|CSMO01000021|c1	08	60	113	82	62	82	80	NC	12
Burkholderia pseudomallei|CIAS01000006|c1	08	60	113	82	62	82	80	NC	12
Burkholderia pseudomallei MSHR332|JQFM01000068|c1	08	60	113	82	62	82	80	NC	12
Burkholderia pseudomallei|CIBB01000020|c1	08	60	113	82	62	82	80	NC	12
Burkholderia pseudomallei|CWJE01000004|c1	08	60	113	82	62	82	80	NC	12
Burkholderia pseudomallei|CFVY01000008|c1	08	60	113	82	62	82	80	NC	12
Burkholderia pseudomallei MSHR5855|JMMV01000071|c4	08	60	113	82	62	82	80	NC	12

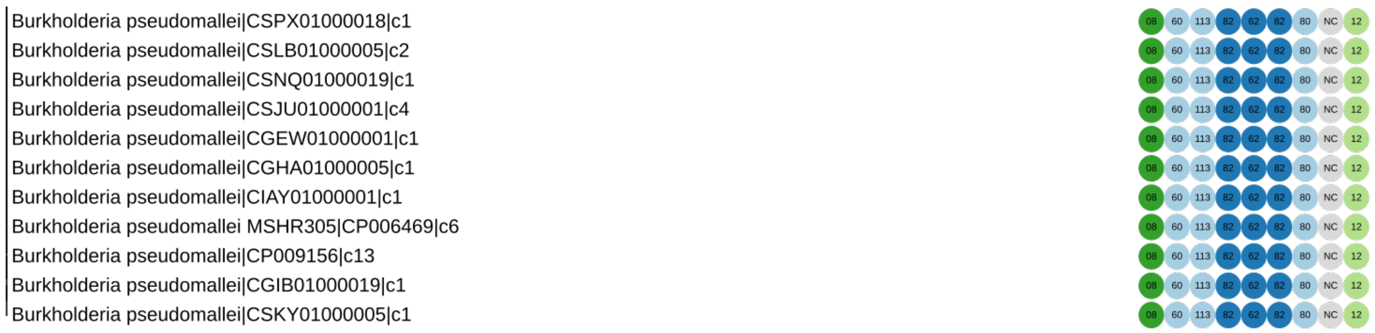
Burkholderia pseudomallei|CSPX01000018|c1
Burkholderia pseudomallei|CSLB01000005|c2
Burkholderia pseudomallei|CSNQ01000019|c1
Burkholderia pseudomallei|CSJU01000001|c4
Burkholderia pseudomallei|CGEW01000001|c1
Burkholderia pseudomallei|CGHA01000005|c1
Burkholderia pseudomallei|CIAY01000001|c1
Burkholderia pseudomallei MSHR305|CP006469|c6
Burkholderia pseudomallei|CP009156|c13
Burkholderia pseudomallei|CGIB01000019|c1
Burkholderia pseudomallei|CSKY01000005|c1
08 60 113 82 62 82 80 NC 12
