# Supplementary material for: Evolution of combinatorial diversity in trans-acyltransferase polyketide synthase assembly lines across bacteria
Source: Nat Commun. 2021 Mar 3;12:1422. doi: 10.1038/s41467-021-21163-x (PMC7930024; doi:10.1038/s41467-021-21163-x)
Supplement: Supplementary file 5 — Reporting Summary [file 41467_2021_21163_MOESM5_ESM.pdf]

nature research

Corresponding author(s): Jörn Piel & Marnix Medema

Last updated by author(s): November 3rd, 2020

Reporting Summary

Nature Research wishes to improve the reproducibility of the work that we publish. This form provides structure for consistency and transparency in reporting. For further information on Nature Research policies, see our Editorial Policies and the Editorial Policy Checklist.

Statistics

For all statistical analyses, confirm that the following items are present in the figure legend, table legend, main text, or Methods section.

n/a	Confirmed	
☐	☒	The exact sample size (*n*) for each experimental group/condition, given as a discrete number and unit of measurement
☐	☒	A statement on whether measurements were taken from distinct samples or whether the same sample was measured repeatedly
☒	☐	The statistical test(s) used AND whether they are one- or two-sided<br>*Only common tests should be described solely by name; describe more complex techniques in the Methods section.*
☒	☐	A description of all covariates tested
☒	☐	A description of any assumptions or corrections, such as tests of normality and adjustment for multiple comparisons
☐	☒	A full description of the statistical parameters including central tendency (e.g. means) or other basic estimates (e.g. regression coefficient) AND variation (e.g. standard deviation) or associated estimates of uncertainty (e.g. confidence intervals)
☒	☐	For null hypothesis testing, the test statistic (e.g. *F*, *t*, *r*) with confidence intervals, effect sizes, degrees of freedom and *P* value noted<br>*Give P values as exact values whenever suitable.*
☒	☐	For Bayesian analysis, information on the choice of priors and Markov chain Monte Carlo settings
☒	☐	For hierarchical and complex designs, identification of the appropriate level for tests and full reporting of outcomes
☒	☐	Estimates of effect sizes (e.g. Cohen's *d*, Pearson's *r*), indicating how they were calculated

Our web collection on statistics for biologists contains articles on many of the points above.

Software and code

Policy information about availability of computer code

Data collection	MS acquisition: Xcalibur 4.1 (Thermo), Chromeleon Xpress version 7.2 (Thermo); NMR acquisition: TopSpin version 3.5.7 (Bruker); HPLC: Open Lab CDS version 2.2 (Agilent)
Data analysis	HMMer 3.1b2, pplacer 1.1.alpha19-1, antiSMASH 5.0, Perl 5.26.2, Python 3.6.3 & 2.7.13, RAxML 8.2.11, Cytoscape 3.3.0, Diamond 0.9.35, TopSpin 3.5.7, MNova 14.1.2, GraphPad Prism 7.0.3, Xcalibur, 4.1 GNPS,<br>Our own software, transPACT is available open-source at https://github.com/chevrm/transPACT

For manuscripts utilizing custom algorithms or software that are central to the research but not yet described in published literature, software must be made available to editors and reviewers. We strongly encourage code deposition in a community repository (e.g. GitHub). See the Nature Research guidelines for submitting code & software for further information.

Data

Policy information about availability of data

All manuscripts must include a data availability statement. This statement should provide the following information, where applicable:

- Accession codes, unique identifiers, or web links for publicly available datasets
- A list of figures that have associated raw data
- A description of any restrictions on data availability

GenBank: The spliceostatin gene cluster from Xanthomonas cannabis was deposited in GenBank under accession number BK010647. MIBiG: The spliceostatin, gynuellalide, and secimide BGCs were deposited in MIBiG under accession numbers BGC0002059, BGC0001835, and BGC0002060.

Field-specific reporting

Please select the one below that is the best fit for your research. If you are not sure, read the appropriate sections before making your selection.

☒ Life sciences ☐ Behavioural & social sciences ☐ Ecological, evolutionary & environmental sciences

For a reference copy of the document with all sections, see nature.com/documents/nr-reporting-summary-flat.pdf

Life sciences study design

All studies must disclose on these points even when the disclosure is negative.

Sample size	No sample size was precalculated; rather, all publicly available genomic and metabolomic data was used for the data analysis, thus maximizing the amount of input data.
Data exclusions	No data was excluded.
Replication	For the analytical chemistry and genomics, we performed no differential analysis between samples/conditions; hence, no replicates were required. Plant experiments were performed in triplicates with individual plants as indicated in Suppl Fig 49. In the HeLa cell assay, four biological replicates per sample were used (see details in Suppl Fig 50). Samples showed coherent phenotypes across the replicates, as can be observed in our supplementary data.
Randomization	Randomization was not relevant for our study, as we did not perform group comparisons.
Blinding	No blinding was performed in our study, as no group comparisons were performed and there was no specific reason to expect bias, as MS/MS and NMR are unbiased techniques.

Reporting for specific materials, systems and methods

We require information from authors about some types of materials, experimental systems and methods used in many studies. Here, indicate whether each material, system or method listed is relevant to your study. If you are not sure if a list item applies to your research, read the appropriate section before selecting a response.

Materials & experimental systems

n/a	Involved in the study	
☒	☐	Antibodies
☐	☒	Eukaryotic cell lines
☒	☐	Palaeontology and archaeology
☒	☐	Animals and other organisms
☒	☐	Human research participants
☒	☐	Clinical data
☒	☐	Dual use research of concern

Methods

n/a	Involved in the study	
☒	☐	ChIP-seq
☒	☐	Flow cytometry
☒	☐	MRI-based neuroimaging

Eukaryotic cell lines

Policy information about cell lines

Cell line source(s)	HeLa cell ine, ATCC
Authentication	Cell lines were not authenticated as they were used to test cytotoxic effects of compounds.
Mycoplasma contamination	Cell lines were not tested for mycoplasma contamination.
Commonly misidentified lines (See ICLAC register)	No commonly misidentified cell lines were used in the study.
